# Adamantane-type clusters: compounds with a ubiquitous architecture but a wide variety of compositions and unexpected materials properties

**DOI:** 10.1039/d4sc01136h

**Published:** 2024-05-02

**Authors:** Niklas Rinn, Irán Rojas-León, Benjamin Peerless, Saravanan Gowrisankar, Ferdinand Ziese, Nils W. Rosemann, Wolf-Christian Pilgrim, Simone Sanna, Peter R. Schreiner, Stefanie Dehnen

**Affiliations:** a Institute of Nanotechnology, Karlsruhe Institute of Technology Herrmann-von-Helmholtz-Platz 1 76344 Eggenstein-Leopoldshafen Germany; b Light Technology Institute, Karlsruhe Institute of Technology Engesserstr. 13 76131 Karlsruhe Germany; c Fachbereich Chemie and Wissenschaftliches Zentrum für Materialwissenschaften, Philipps University Marburg Hans-Meerwein-Straße 4 35043 Marburg Germany; d Department of Chemistry, Justus Liebig University Giessen Heinrich-Buff-Ring 17 35392 Giessen Germany; e Center for Materials Research, Justus Liebig University Giessen Germany

## Abstract

The research into adamantane-type compounds has gained momentum in recent years, yielding remarkable new applications for this class of materials. In particular, organic adamantane derivatives (AdR_4_) or inorganic adamantane-type compounds of the general formula [(RT)_4_E_6_] (R: organic substituent; T: group 14 atom C, Si, Ge, Sn; E: chalcogenide atom S, Se, Te, or CH_2_) were shown to exhibit strong nonlinear optical (NLO) properties, either second-harmonic generation (SHG) or an unprecedented type of highly-directed white-light generation (WLG) – depending on their respective crystalline or amorphous nature. The (missing) crystallinity, as well as the maximum wavelengths of the optical transitions, are controlled by the clusters' elemental composition and by the nature of the organic groups R. Very recently, it has been additionally shown that cluster cores with increased inhomogeneity, like the one in compounds [RSi{CH_2_Sn(E)R′}_3_], not only affect the chemical properties, such as increased robustness and reversible melting behaviour, but that such ‘cluster glasses’ form a conceptually new basis for their use in light conversion devices. These findings are likely only the tip of the iceberg, as beside elemental combinations including group 14 and group 16 elements, many more adamantane-type clusters (on the one hand) and related architectures representing extensions of adamantane-type clusters (on the other hand) are known, but have not yet been addressed in terms of their opto-electronic properties. In this review, we therefore present a survey of all known classes of adanmantane-type compounds and their respective synthetic access as well as their optical properties, if reported.

## Abstract 2

Adamantane-type clusters exhibit a huge diversity of chemical compositions, structural details, and resulting properties.

## Graphical abstract

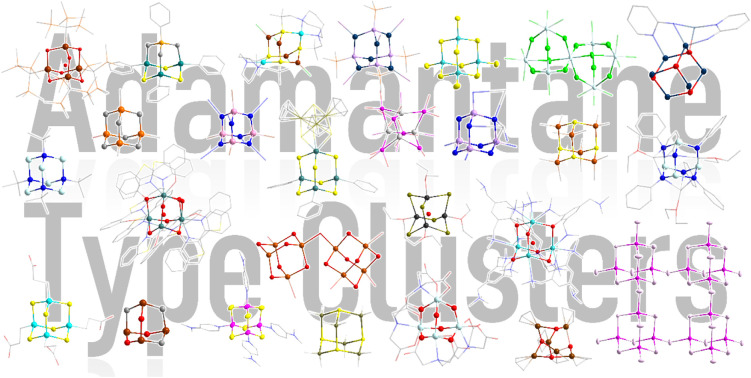

## 1. Introduction

Diamond, in its cubic modification, is the hardest solid on Earth, which is due to the unique structure and bonding with four strong bonds directing in a perfectly tetrahedral manner to four neighbors in a three-dimensional network of face-centered cubic (*F*3̄*m*) symmetry.^1^ It is therefore reasonable that the heavier congeners, Si, Ge, and α-Sn also adopt this structure. However, not only those, but also isoelectronic binary or multinary solids follow this structural concept, as the same overall electron count allows for a corresponding electronic structure of the material. The most well-known examples are 1 : 1 combinations of atoms of groups 13 and 15 or 12 and 16 such as GaAs or ZnS, but more complex compositions, like CuFeS_2_, can also be derived from the cubic diamond network by replacement of the atomic sites in a tetragonal superstructure. Moreover, there are also “filled” versions, like the Zintl phase NaTl with two intertwining diamond networks of covalently bonded Tl atoms and non-bonding Na^+^ cations, or crystobalite-type SiO_2_ with O atoms bridging between the Si atoms that are arranged in a diamond-type pattern. Naturally, the chemical and physical properties of the materials vary as a consequence of the different elemental combinations and corresponding changes in bond strengths and electronic structures. This is extensively taken advantage of in technical applications – starting with the electrical insulator and heat conductor diamond, *via* all kinds of semiconductor applications of the heavier homologues and the binary analogs, to more specific applications of the more complex compounds.

However, the structure and bonding concept of diamond, which is overwhelmingly successful in solid state compounds, is not restricted to the three-dimensional extension. On the contrary, molecular fragments of these structures are even more diverse. The parent structural fragment of diamond is adamantane (derived from the greek adamas for diamond). The adamantane-type topology (or adamantane-type scaffold) is based on a core structure with ten atoms, four of which represent the bridgehead atoms, and six of which occupy the briding positions. It has a sum formula of C_10_H_16_ (or (CH)_4_(CH_2_)_6_) and was first proposed in 1924 by Decker, who investigated the compound under the name “decaterpene,” which would later be recognized as adamantane.^2^ However, due to its exceedingly low natural abundance (0.0004%),^3^ it took another decade until adamantane was identified in crude oil in Hodonin, Czechoslovakia in 1933. The adamantane-type scaffold, just like its parent solid state structures, is found in a multitude of compounds scattered throughout the periodic table. Innumerous admantane derivatives have been realized – either by replacing H with other atoms or molecules, or by isoelectronic replacement of some or all of the C atoms or CH_2_ units – like in the related solids with diamond-type structures. A very prominent derivative of the admantane molecule is urotropine, N_4_(CH_2_)_6_, a condensation product of ammonia and formaldehyde, in which the C–H bridgehead units are replaced with isoelectronic N atoms. There are also purely inorganic analogs. One of the first purely inorganic adamantane-type molecules, and maybe the most prominent example, is phosphorous pentaoxide that consists of binary molecules of P_4_O_10_, the structure of which was suggested in the late 19th century.^4^ Inorganic cluster cores of the type {Q_4_E_6_} are obtained when replacing the bridgehead C atoms (position Q) with atoms of another group 14 element and the CH_2_ groups (position E) with atoms of a group 16 element. Saturation of the bridgehead atoms requires a substituent to form either binary anions [Q_4_E_10_]^4−^ (Q = Si, Ge, Sn) or hybrid clusters of the type [(RQ)_4_E_6_], with Q = group 14 element Si, Ge, or Sn and R = organic or organometallic group substituent.

While the first observation of these molecules was unintended and caused excitement for the beautiful structure, the development is now in the direction of the compounds' intriguing chemical and physical properties. With regard to the effects of the substitution of elements on these features, the same rules apply to molecules as to solids, which enables fine-tuning across a broad spectrum. To make use of these properties, however, it is necessary to know all about the synthetic approaches and their respective modifications, and develop them further. In this review, we therefore aim at giving a comprehensive overview of the various synthesis pathways of compounds with a molecular adamantane-type structure across the periodic table, and discuss methods for the functionalization of the organic adamantane. To keep in scope, we have decided to limit the organic synthesis to tetrasubstituted adamantanes. Based on this, we will further elaborate on the optical (nonlinear) properties and structural features of the different compounds in the solid state.

## 2. Variety of compositions and syntheses

### 2.1 Inorganic and hybrid compounds

Inorganic and hybrid compounds featuring adamantane-type architectures are formed with elements from nearly all groups across the periodic table. In this section, we will discuss their synthetic access and elaborate on prevalent methods for the formation of molecules with specific elemental combinations. This will be discussed for each combination of groups from the periodic table using the Q/E nomenclature introduced above, with Q representing the atom(s) featuring three bonds within the adamantane-type structure, and E representing the atoms or groups bridging between two of the former positions. Some methods are commonly used for all elements and will be briefly discussed first with one example given for each; for easier tracking, a letter will be assigned to those procedures, to be referred to later in the course of this article.

#### Method A

**Scheme 1:**
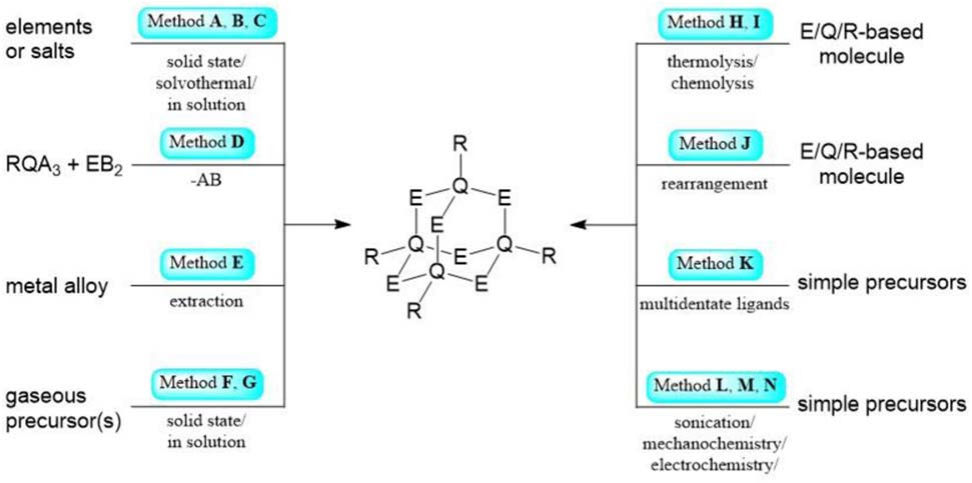
Simplified representation of the synthetic Methods A–N for the formation of adamantane-type clusters.

One common synthesis method is a solid-state reaction starting directly from the elements or from binary salts. It is a simple way to realize uncomplicated adamantane molecules, but it also requires high temperatures, which prohibits the use of some precursors (Scheme 1, top left).

Example:^253^
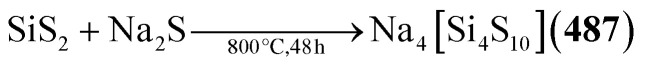


#### Method B

Similar to Method A, solvothermal reactions are commonly used to generate adamantane-like structures. In those reactions, a solvent, elements or binary precursors (as well as some additives, if applicable) are reacted in a closed vessel at elevated temperatures. Compared to Method A, these oftentimes use lower temperatures, and milder conditions allow for the use of more diverse precursors (Scheme 1, top left).

Example:^65^
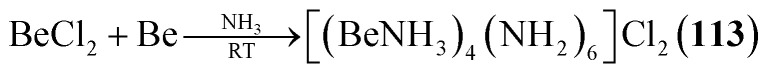


#### Method C

As in A or B, simple salts or elements are reacted, but this time, the reaction takes place in solution at temperatures below their respective boiling points in open vessels. The very mild conditions allow for the addition of additives or catalysts and for more thermally unstable adamantanes to be realized (Scheme 1, top left).

Example:^253^
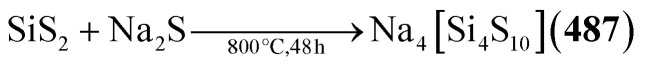


#### Method D

If the precursor used for the Q component contains three leaving groups and those for E contain two, a condensation reaction can yield adamantane compounds with each bond of the scaffold formally formed by one condensation event. This mostly occurs for metal (pseudo)halides reacted with alkaline metal salts of E anions, H_*x*_E, or silyl derivatives of E. Such syntheses are normally carried out in solution at mild temperatures. The formation of the condensation side product can be the driving force in the reaction (Scheme 1, left upper center).

Example:^304^
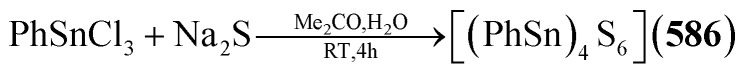


#### Method E

Some purely inorganic molecules can be obtained by first creating a solid phase—by melting the corresponding elements or binary salts—and subsequently extracting this phase with an appropriate solvent. This sometimes takes place in the presence of a sequestering agent, like a crown ether or a cryptand, or other additives. Common solvents for this method are ethane-1,2-diamine or THF (Scheme 1, left lower center).

Example:^282^
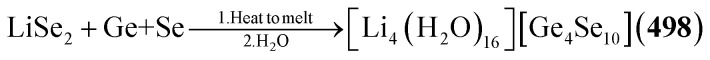


#### Method F

Gaseous reactants like H_2_S, H_2_, O_2_ or PH_3_ can be introduced to solid reaction partners at high temperatures to occupy the E position during construction of the adamantane scaffold (Scheme 1, bottom left).

Example:^304^
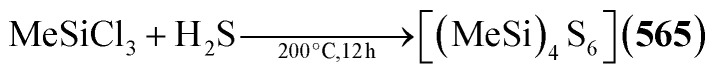


#### Method G

Similar to F, but in the liquid phase, hence these reactions often do not require high temperatures (Scheme 1, bottom left).

Example:^78,79^
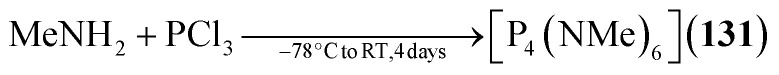


#### Method H

Thermal decomposition of a precursor can lead to the formation of simple adamantanes, sometimes in the presence of a catalyst or additive (Scheme 1, top right).

Example:^62,63^
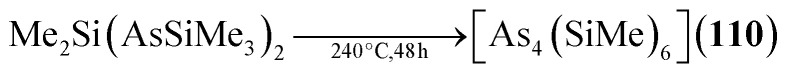


#### Method I

Chemically induced decomposition by hydrolysis or acidic decomposition of a precursor can afford adamantane-type clusters, especially for oxide and hydroxide species. From a mechanistic viewpoint, this is often similar to Method D, but may happen unintentionally under ambient conditions (Scheme 1, top right).

Example:^116^
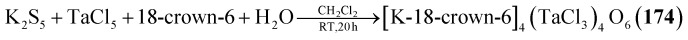


#### Method J

In some cases, rearrangement of molecules or other cluster architectures to the adamantane-type scaffold induced by heat, catalysts, or other reactants were reported (Scheme 1, right upper center).

Example:^28^
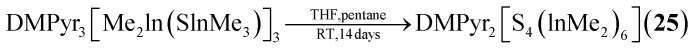


#### Method K

Multidentate organic ligands, mostly with oxygen or nitrogen sites, or preformed cluster fragments can be used as templates to fill the E position in adamantane-type compounds (Scheme 1, right lower center).

Example:^30^
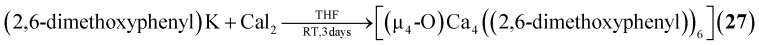


#### Method L

Reactions towards adamantane-type clusters can be induced by sonication (Scheme 1, bottom right).

Example:^29^
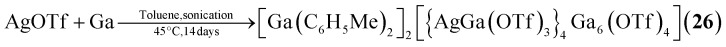


#### Method M

Mechanochemical reactions can prompt isomerisation to the desired adamantane-type molecules (Scheme 1, bottom right).

Example:^86^
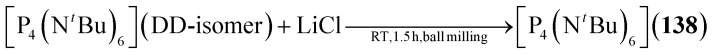


#### Method N

Electrochemical methods can form adamantane-type clusters from appropriate electrodes and electrolytes (Scheme 1, bottom right).

Example:^194^
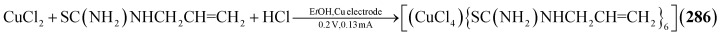


#### Method O

**Scheme 2:**
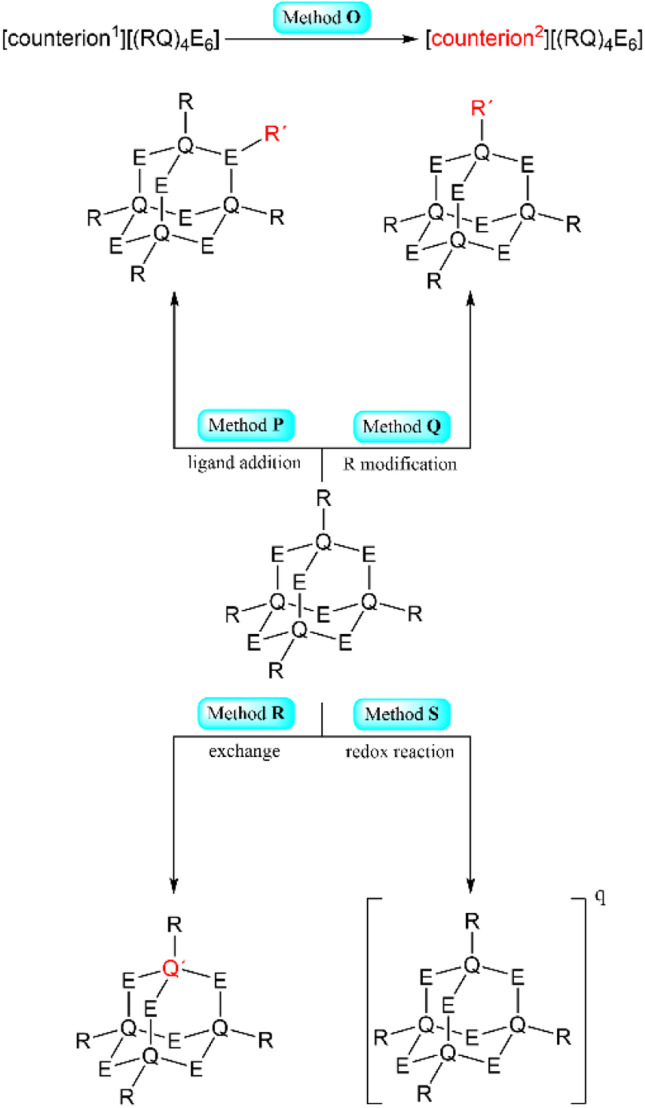
Simplified representation of the synthetic Methods O–S for the formation of adamantane-type clusters by modification of an adamantane-type cluster compound.

For ionic clusters, new compounds can be generated by exchanging the counter ion to introduce new functionalities or templating counter ions (Scheme 2, top).

Example:^293^
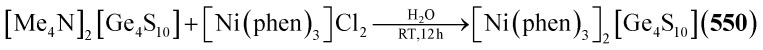


#### Method P

In a few cases, ligands can be added to an existing adamantane core in a position that did not previously form bonds outside the cluster scaffold (Scheme 2, top left).

Example:^345–347^
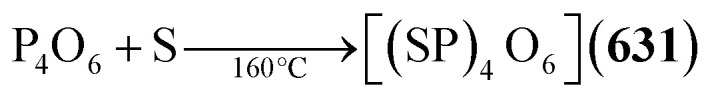


#### Method Q

Clusters with (organic) ligands can be expanded by modification of the ligand, by formal ligand exchange, or by ligand abstraction to afford new compounds (Scheme 2, top right).

Example:^512^
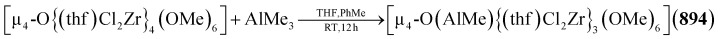


#### Method R

Reactions of adamantane-type or other clusters to replace atoms in their inorganic core, sometimes combined with a rearrangement of the architecture to the adamantane scaffold. This includes exchange reactions in Q and E positions between adamantanes. This method can also be used to create larger clusters with ternary inorganic cores of other architectures, especially when an anion source is additionally provided (Scheme 2, bottom left).

Example:^131^
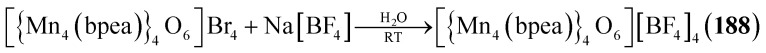


#### Method S

Chemical reduction or oxidation of an adamantane-type cluster can, in some cases, be done under retainment of its structural motif (Scheme 2, bottom right).

Example:^131^
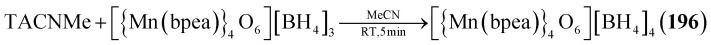


#### Method T

**Scheme 3:**
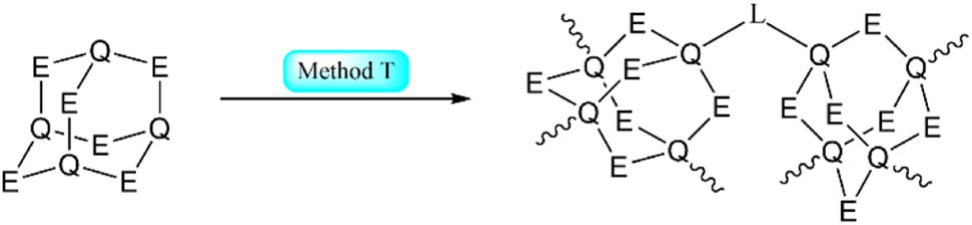
Simplified representation of the synthetic Method T to generate a polymeric compound from adamantane-type clusters.

A method for the generation of extended structures is the linkage of inorganic adamantane-type clusters using transition metal compounds or other linkers in solution. Sometimes, this is combined with an ion exchange and some additives (Scheme 3).

Example:^533^
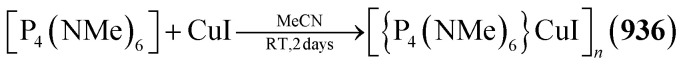


#### Method U

There are a couple of unique approaches, which are not outlined in this overview.

In the following, we will dicuss all different families of adamantane-type compounds in groups sorted by their elemental combination. This will be done in order of the group of the atoms in the E position, starting with hydride clusters and moving along to halide species. The only main groups that do not occur in the E position are groups 2 and 18.

Being rather uncommon, examples with transition metal atoms in the Q positions will be discussed last. In some of the final subsection, we will give an overview of clusters comprising elements from different groups in their scaffold, as well as extended and polymeric species.

All cluster examples, along with their simplified synthesis/reaction methods, are given in tables at the end of each section (Tables 1–23); for the sake of readability, the respective synthesis methods are not always referred to in the main text though. If the reaction temperature is not specified in the table, the reaction was carried out at ambient temperature. Similarly, reactions without specified durations occur instantaneously. Purification times and methods are not included for purifications that occur in additional, subsequent steps.

**Table 1:** Adamantane-type compounds with hydrogen or group 1 atoms in the E position^a^

Compound	Reagents/conditions	Method
[(MgIDipp)_2_(MgHMDS)_2_H_6_] (1)	IDipp, [Mg{N(SiMe_3_)_2_}_2_], PhSiH_3_/hexane, 60 °C, 3 h	C^11^
[(CaTACNMe)_4_H_6_][B(C_6_H_3_-3,5-Me_2_)] (2)	H_2_ (1 bar), [(Me_3_TACNMe)Ca(CH_2_Ph)(thf)_*x*_][B(C_6_H_3_-3,5-Me_2_)]/THF, 70 °C, 6 h	G^12^
[AsPh_4_]_2_[Re_4_(CO)_12_H_6_] (3)	Re_2_(CO)_10_, NaBH_4_, (C_6_H_5_)_4_AsCl/THF, EtOH	C^13^
[Me_3_BnN]_2_[Re_4_(CO)_12_H_6_] (4)	Re_2_(CO)_10_, KOH, [Me_3_BnN]Cl/MeOH, 65 °C, prolonged heating	H^14^
[(Cp*Zr)_4_H_6_] (5)	[(μ-H)(μ_3_-H)(Cp*ZrCl)]_4_, Na in Hg/Et_2_O, 1 month	J^5^
[(ZnIDipp)_2_(ZnHMDS)_2_H_6_] (6)	Zn(HMDS)_2_, IDipp, DMAB/cyclohexane, RT, 30 min	C^15^
[Ir_4_(IMe)_7_(CO)H_10_][BF_4_]_2_ (7)	[Ir(cod)(IMe)_2_][BF_4_], KOH, Na[Bar^F^]/glycerol, H_2_O, 120 °C, 24 h	J^16^
[Ir_4_(IMe)_7_(CO)H_10_][BAr^F^]_2_ (8)	[Ir_4_(IMe)_7_(CO)H_10_][BF_4_]_2_ (7), Na[Bar^F^]/dichlormethane, 2 h	O^16^
[Ir_4_(IMe)_8_H_10_][BAr^F^]_2_ (9)	[Ir(cod)(IMe)_2_] [BF_4_], KOH, NaBar^F^/glycerol, H_2_O, 120 °C, 24 h	J^17^
[{Me_2_P(BH_3_)CHSiMe_2_OLi}_4_Li_4_(Et_2_O)_2.75_(thf)_1.25_] (10)	1. Me_3_P(BH_3_), *n*-BuLi/THF, 2 h	J^18^
	2. (Me_2_SiO)_3_/Et_2_O, 2 h	

a IDipp = 1,3-bis(2,6-diisopropylphenyl)imidazole-2-ylidene, HMDS = 1,1,1,3,3,3-hexamethyldisilazide, TACNMe = 1,4,7-trimethyl-1,4,7-triazacyclononane, Bn = benzyl, Cp* = pentamethylcyclopentadienyl, DMAP = dimethylamine borane, BAr^F^ = [B[3,5-(CF_3_)_2_C_6_H_3_].

We will illustrate examples of molecular structures of all cluster types that were obtained in single-crystalline form. For crystallographic details, we refer to the original literature.

#### 2.1.1 Q/H and Q/group 1 adamantane-type clusters

A number of hydride clusters with (transition) metals have been realized, which most often comprise a central metal tetrahedron with direct metal–metal bonds. They are formally constructed by coordinating all edges of this central element by hydrides. They can be seen as one point in a continuum of related compounds featuring fewer hydrogen atoms or additional (bridging) ones, respectively. Although, those will not be further discussed except for some examples.^5–10^ Apart from that, there is one species with lithium coordinated by oxygen atoms.

**Fig. 1:**
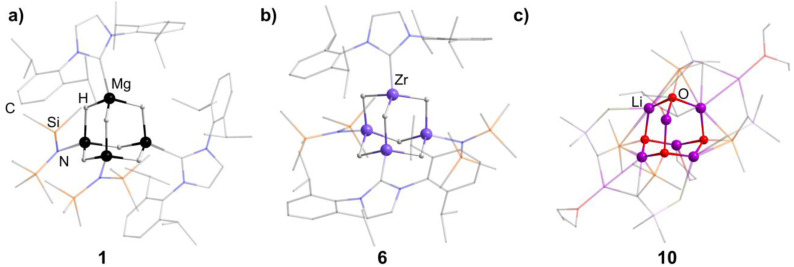
Examples of adamantane-type compounds with hydrogen or group 1 atoms in the E position: [(MgIDipp)_2_(MgHMDS)_2_H_6_] (1, left (a)), [(ZnIDipp)_2_(ZnHMDS)_2_H_6_] (6, middle (b)) and [{Me_2_P(BH_3_)CHSiMe_2_OLi}_4_Li_4_(Et_2_O)_2.75_(thf)_1.25_] (10, right (c)). Hydrogen atoms in the ligands are omitted for clarity.

An N-heterocyclic carbene can coordinate to [Mg(HMDS)_2_] (HMDS = 1,1,1,3,3,3-hexamethyldisilazide) and in turn be reacted with PhSiH_3_, resulting in the adamantane-type cluster [(MgIDipp)_2_(MgHMDS)_2_H_6_] (1, IDipp = 1,3-bis(2,6-diisopropylphenyl)imidazole-2-ylidene, Fig. 1), where the magnesium atoms carry either an IDipp or N(SiMe_3_)_2_ ligand.^11^ A calcium congener [(CaTACNMe)_4_H_6_][B(C_6_H_3_-3,5-Me_2_)] (2, TACNMe = 1,4,7-trimethyl-1,4,7-triazacyclononane) is obtained from an *in situ*-formed complex [(TACNMe)Ca(CH_2_Ph)(thf)_*x*_][B(C_6_H_3_-3,5-Me_2_)] after treatment with H_2_ gas under elimination of toluene, with all Ca atoms carrying the same tridentate ligand.^12^

The first transition metal cluster anion in this group, [{(CO)_3_Re}_4_H_6_]^2−^ (in 3 and 4) was formed from [Re_2_(CO)_10_], either by reaction with Na[BH_4_]^13^ or by prolonged heating under basic conditions in methanol as one of multiple products.^14^

The adamantane-type compound [(Cp*Zr)_4_H_6_] (5, Cp* = pentamethylcyclopentadienyl) was found as the final piece in a row of tetrahedral compounds with fewer hydrides by reduction of [(μ-H)(μ_3_-H)(Cp*ZrCl)]_4_ with Na amalgam.^5^ This led to a mixed-valence Zr^II^/Zr^III^ situation in the cluster core.

An analog to the aforementioned [(MgIDipp)_2_(MgHMDS)_2_H_6_] cluster was realized with zinc in [(ZnIDipp)_2_(ZnHMDS)_2_H_6_] (6, Fig. 1).^15^ The synthesis strategy runs in parallel as well, with Zn(HMDS)_2_ as the metal precursor and dimethylamine borane as a hydride source.

A number of iridium hydride clusters (7–9) could be obtained upon dehydrogenation reactions catalyzed by [Ir(Ime)_2_(cod)][BF_4_] (cod = 1,5-cyclooctadiene) of glycerol.^16,17^ This results in the formation of the hydride as well as CO ligands at the metal center in some cases.

A {Li_4_O_6_} adamantane-like core can be observed in the larger complex [{Me_2_P(BH_3_)CHSiMe_2_OLi}_4_Li_4_(Et_2_O)_2.75_(thf)_1.25_] (10, Fig. 1). It is formed as the tetramer of the *in situ* generated linear molecule Me_2_P(BH)CH(Li)Si(Me_2_)OLi coordinated by additional solvent molecules.^18^

#### 2.1.2 Q/group 13 adamantane-type clusters

Adamantane-type clusters with group 13 atoms in the E position are known for groups 14 to 16, with a few unique examples in each group and without a unifying synthetic route. Additionally, there is also an example with a {Ag_4_Ga_10_} adamantane-type scaffold. A brief description of the formation conditions for all of them is given in the following paragraphs.

**Fig. 2:**
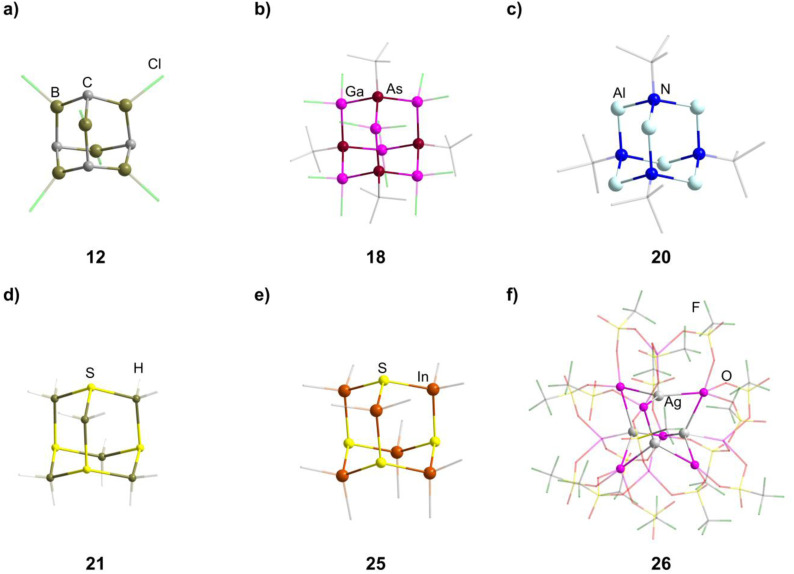
Examples of adamantane-type compounds with group 13 atoms in the E position: [(CH)_4_(BCl)_6_] (12, top left (a)), [Li(thf)_4_]_2_[(^*t*^BuAs)_4_(GaCl_2_)_6_] (18, top center (b)), Li_2_[(^*t*^BuN)_4_(AlH_2_)_6_] (20, top right (c)), Na_2_[S_4_(BH_2_)_6_] (21, bottom left (d)), DMPyr_2_[S_4_(InMe_2_)_6_] (25, bottom center (e)) and [Ga(C_6_H_5_Me)_2_]_2_[{AgGa(OTf)_3_}_4_Ga_6_(OTf)_4_] (26, bottom right (f)). Hydrogen atoms in the organic ligands and counterions, if present, are omitted for clarity.

Different approaches for the formation of the few known carbon/group 13 adamantane-type compounds have been showcased in the literature. The boron congeners [(RC)_4_(R′B)_6_] (11–13, Fig. 2) can be synthesized at higher temperatures by pyrolysis of BMe_3_ or (Cl_2_B)_2_CH_2_,^19–21^ or by a solid state reaction of HC(BEt_2_)_3_ and BEt_3_ in the presence of AlEt_3_.^22^ At room temperature, the rearrangement of (BEt)_3_(CMe)_2_ to [(CMe)_4_(BEt)_6_] (14) was observed, induced by elemental potassium and I_2_.^23^

A unique synthetic approach, featuring R_2_GaH and alkenes HC≡CR′, leads to the formation of carbagallane adamantane-type structures [(R′C)_4_(RGa)_6_] (15–17).^24^ It involves the intermediate formation of dialkyl(alkenyl)gallium compounds, which react with additional R_2_GaH to form the clusters under elimination of GaR_3_.

Three dianionic group 15 congeners exist. An As/Ga compound [Li(thf)_4_]_2_[(^*t*^BuAs)_4_(GaCl_2_)_6_] (18, ^*t*^Bu = tertiary butyl, Fig. 2) is isolated by a simple condensation reaction of InCl_3_ and Li_2_As^*t*^Bu at low temperatures,^25^ while the compounds Li_2_[(RN)_4_(AlH_2_)_6_] (19–20, R = Me, ^*t*^Bu, Fig. 2) are formed by condensation of Li[AlH_4_] and [RNH_3_]Cl.^26^

The sulfur containing Na_2_[S_4_(BH_2_)_6_] (21, Fig. 2) adamantane-type cluster is obtained by a stepwise condensation reaction of THF·BH_3_, and Na[BH_4_] with H_2_S under elimination of H_2_.^27^ In the reaction, an intermediate species (BH_3_)S(B_2_H_5_) is formed, which reacts with additional H_2_S to give the final product. The Se congener is formed *via* a different species with elemental Se and Na[BH_4_]. This leads to Na_2_[H_3_BSe–SeBH_3_] which, under the influence of elevated temperature and BH_3_, reforms Na_2_[Se_4_(BH_2_)_6_] (22). Both the sulfur and selenium homologs undergo a cation exchange to the Cs compounds (23–24) with CsBr. The only other example of a group 16-based adamantane in this category is DMPyr_2_[S_4_(Me_2_In)_6_] (25, Fig. 2), which is a decomposition side product of the six membered ring DMPyr_3_[Me_2_In(SInMe_3_)]_3_, which could not yet be synthesized in a pure form.^28^

The single example featuring a transition metal [Ga(C_6_H_5_Me)_2_]_2_[{AgGa(OTf)_3_}_4_Ga_6_(OTf)_4_] (26, OTf = O_3_SCF_3_, Fig. 2) comprises bridging triflate ligands between the gallium atoms, with the terminal gallium moieties connecting to three, and the atoms in the E position to four, ligands.^29^ It is formed by silver triflate reacting with elemental gallium after ultrasonic activation.

**Table 2:** Adamantane-type compounds with group 13 atoms in the E position^a^

Compound	Reagents/conditions	Method
[(CH)_4_(BMe)_6_] (11)	BMe_3_/450 °C, 40 min	H^19,20^
[(CH)_4_(BCl)_6_] (12)	(Cl_2_B)_2_CH_2_/450 °C to RT, 12 h	H^21^
[(CH)_4_(BEt)_6_] (13)	HC(BEt_2_)_3_, BEt_2_, AlEt_3_/150 °C	A^22^
[(CMe)_4_(BEt)_6_] (14)	(BEt)_3_(CMe)_2_, I_2_, K/THF	J^23^
[(EtC)_4_(GaEt)_6_] (15)	Et_2_GaH, HC≡CEt/−196 °C to RT, 4 h	U^24^
[(^*n*^BuC)_4_(GaEt)_6_] (16)	Et_2_GaH, HC≡C^*n*^Bu/4 h	U^24^
[(EtC)_4_(GaMe)_6_] (17)	Me_2_GaH, HC≡CEt/−196 °C to RT, 4 h	U^24^
[Li(thf)_4_]_2_[(^*t*^BuAs)_4_(GaCl_2_)_6_] (18)	Li_2_As^*t*^Bu, GaCl_3_/Et_2_O, −78 °C to RT, 3 days	C^25^
Li_2_[(RN)_4_(AlH_2_)_6_] (19–20, R = Me, ^*t*^Bu)	Li[AlH_4_], [RNH_3_]Cl,/Et_2_O, 4 weeks	C^26^
Na_2_[S_4_(BH_2_)_6_] (21)	THF·BH_3_, Na[BH_4_], H_2_S/0 °C	C^27^
Na_2_[Se_4_(BH_2_)_6_] (22)	1. Se, Na[BH_4_]/diglyme, 0 °C to 110 °C, 8 h	B^27^
	2. THF·BH_3_/diglyme	
Cs_2_[S_4_(BH_2_)_6_] (23)	Na_2_[S_4_(BH_2_)_6_] (21), CsBr/H_2_O	O^27^
Cs_2_[Se_4_(BH_2_)_6_] (24)	Na_2_[Se_4_(BH_2_)_6_] (22), CsBr/H_2_O	O^27^
DMPyr_2_[S_4_(InMe_2_)_6_] (25)	DMPyr_3_[Me_2_In(SInMe_3_)]_3_/THF, pentane, 14 days	J^28^
[Ga(C_6_H_5_Me)_2_]_2_[{AgGa(OTf)_3_}_4_Ga_6_(OTf)_4_] (26)	AgOTf, Ga/toluene, 45 °C, 1.5 h (ultrasonic activation)	L^29^

a ^*n*^Bu = normal butyl, ^*t*^Bu = tertiary butyl, diglyme = bis(2-methoxyethyl) ether, DMPyr = 1,1-dimethylpyrrolidinium, OTf = O_3_SCF_3_

#### 2.1.3 Q/group 14 adamantane-type clusters

An extensive family of silaadamantanes obtained from exchanging some or all carbon positions in organic adamantanes with silicon form the largest group in this section. Targeted ligand substitution has been extensively studied in their case. There are also two publications of Si_4_E_6_ compounds with Ge and Sn in the E position. In combination with P or As, neutral adamantanes of the type [(E^15^)_4_(E^14^R_2_)_6_] form a small subset. But at first, we will discuss clusters with metal atoms in the Q position, with an example from group 2, 8 and 10.

##### 2.1.3.1 Group 2/group 14 adamantane-type clusters

**Fig. 3:**
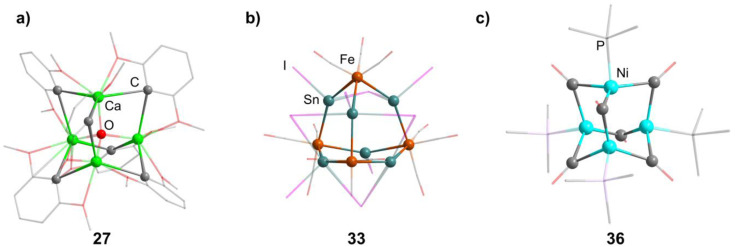
Examples of adamantane-type compounds with group 2 and transition metals in the Q position and group 14 atoms in the E position: [(μ_4_-O)Ca_4_(2,6-dimethoxyphenyl)_6_] (27, left (a)), [BMIm]_6_[S][{Fe(CO)_3_}_4_Sn_6_I_10_]_2_ (33, center (b)) and [(NiPMe_3_)_4_(CO)_6_] (36 right (c)). Hydrogen atoms and counterions, if present, are omitted for clarity.

This unique group 2/14 adamantane-type, [(μ_4_-O)Ca_4_(2,6-dimethoxyphenyl)_6_] (27, Fig. 3), which is formed around a central oxygen atom, uses the tridentate dimethoxyphenyl group as a templating ligand.^30^ These ligands bridge the Ca sites both by a carbon atom in the E position, as well as by coordination with their oxygen atoms. The origin of the central μ_4_-O atom could not be determined and might stem either from H_2_O or O_2_ impurities during the inert gas protected reaction, or decomposition of the solvent/ligand.

##### 2.1.3.2 Group 8/14 adamantane-type clusters

In two studies, Fe clusters have been characterized. In the cluster family [(Fe)_4_(aryl)_6_(thf)_*x*_] (28–31), the carbon atoms of aryls are found in the E position. These clusters are prepared by reacting Fe(acac)_3_ (acac = acetylacetonate) with the aryl Grignard reagent (aryl)MgBr.^31^

In the other study, reactions of SnI_4_ and Fe(CO)_5_ in ionic liquids lead to Fe/Sn compounds. [BMIm]_2_[{Fe(CO)_3_}_4_Sn_6_I_10_] (32, BMIm = 1-butyl-3-methyl-imidazolium) or [BMIm]_6_[S][{Fe(CO)_3_}_4_Sn_6_I_10_]_2_ (33, Fig. 3) depending on the counterion in the ionic liquid.^32^ They each feature different Sn coordination sites. In 32, three Sn atoms carry two iodo ligands, one is connected to only one iodine and the final two carry one terminal iodine and one bridging μ-I connecting them to each other. The second cluster comprises three tin atoms carrying two iodine ligands, while the other three only connect to one terminal iodide each and are connected *via* a μ_3_-I bridge.

##### 2.1.3.3 Group 8/14 adamantane-type clusters

Group 10 clusters with group fourteen elements in the E position are known for combinations with Ni and Pd.

The first family of such compounds with the general compositon [(NiPR_3_)_4_(CO)_6_] (34–37, Fig. 3) comprise CO bridged Ni tetrahedra with terminal phosphine ligands.^33–35^ They are generally prepared by reacting a Ni complex with the desired phosphine and CO gas, if the original complex does not contain such ligands already. These results could be transferred to palladium in the case of [(PdP^*n*^Bu_3_)_4_(CO)_6_] (38).^36^

**Table 3:** Adamantane-type compounds with group 2 and transition metals in the Q position and group 14 atoms in E position^a^

Compound	Reagents/conditions	Method
[(μ_4_-O)Ca_4_(2,6-dimethoxyphenyl)_6_] (27)	(2,6-Dimethoxyphenyl)K, CaI_2_/THF, 3 days	K^30^
[Fe_4_(Ph)_6_(THF)_4_] (28)	Fe(acac)_3_, PhMgBr/THF, −30 °C, 25 min	C^31^
[Fe_4_(*p*-tolyl)_6_(THF)_4_] (29)	Fe(acac)_3_, *p*-tolylMgBr/THF, −30 °C, 25 min	C^31^
[Fe_4_(*p*-tolyl)_6_(THF)_3_] (30)	Fe(acac)_3_, *p*-tolylMgBr/THF, −30 °C, 25 min	C^31^
[Fe_4_(4-F-Ph)_6_(THF)_4_] (31)	Fe(acac)_3_, 4-F-PhMgBr/THF, −30 °C, 25 min	C^31^
[BMIm]_2_[{Fe(CO)_3_}_4_Sn_6_I_10_] (32)	SnI_4_, Fe(CO)_5_, [BMIm][NTf_2_]/130 °C, 4 days	B^32^
[BMIm]_6_[S][{Fe(CO)_3_}_4_Sn_6_I_10_]_2_ (33)	SnI_4_, NH_4_I, Fe(CO)_5_, [BMIm][OTf]/130 °C, 4 days	B^32^
[{NiP(CH_2_CH_2_CN)_3_}_4_(CO)_6_] (34)	Tris-(2-cyanoethyl)phosphine, Ni(CO)_4_/MeOH, 70 °C, 24 h	B^33^
[(NiPMe_3_)_4_}[BF_4_][(NiPMe_3_)_4_(CO)_6_] (35)	Ni(COMe)Cl(PMe_3_)_2_, PMe_3_, Tl[BF_4_]/CH_2_Cl_2_, RT, 30 min	C^34^
[(NiPMe_3_)_4_(CO)_6_] (36)	Bis(cod)nickel, PMe_3_, CO/toluene, RT, 6 h	F^35^
[(NiP^*n*^Bu_3_)_4_(CO)_6_] (37)	Bis(cod)nickel, P^*n*^Bu_3_, CO/toluene, RT, 6 h	F^35^
[(PdP^*n*^Bu_3_)_4_(CO)_6_] (38)	Pd_4_(CO)_5_(PBu_3_^*n*^)_4_, CH_3_COOH/EtOH, pentane, RT, 2 days or Pd(OAc)_2_, CH_3_COOH, CO, PBu_3_^*n*^/dioxane, Me_2_CO, 5 days	J/F^36^

a cod = 1,5-cyclooctadiene, OAc = acetate, acac = acetylacetonate, BMIm = 1-butyl-3-methyl-imidazolium, NTf_2_ = bistrifluoridomethansulfonimide.

##### 2.1.3.4 Group 14/14 adamantane-type clusters

**Fig. 4:**
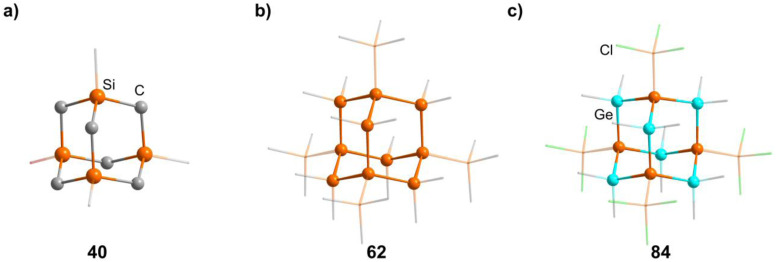
Examples of adamantane-type compounds with group 14 elements in the Q position and group 14 atoms in the E position: [(SiMe)_4_(CH_2_)_6_] (40, left (a)), [(SiSiMe_3_)_4_(SiMe_2_)_6_] (62, center (b)) and [(SiSiCl_3_)_4_(GeMe_2_)_6_] (84, right (c)). Hydrogen atoms are omitted for clarity.

A family of tetrasilaadamantanes of the composition [(RSi)_4_(CH_2_)_6_] has been investigated, with the first examples being obtained in high temperature reactions of either SiMe_4_ to form [(SiMe)_4_(CH_2_)_6_] or SiCl_4_ and Me_3_SiCl in the presence of AlCl_3_ to yield [(SiCl)_4_(CH_2_)_6_] (39).^37–40^ In subsequent work, access to such compounds was made at considerably lower temperatures and in higher yields. [(SiMe)_4_(CH_2_)_6_] (40, Fig. 4) could be obtained from an AlBr_3_ induced rearrangement of (Me_2_SiCH_2_)_3_ at 100 °C, which could then in turn be reacted with Cl_2_ and I_2_ to form 39 or be treated with Li[AlH_4_] to form the hydrogen terminated [(SiH)_4_(CH_2_)_6_] (41).^39,41,42^*Via* both of these routes, tetrasilaadamantanes with mixed methyl and halide positions can be isolated as well.^41–43^ These clusters described so far are used as the basis for ligand exchange reactions at the Si sites (Method Q, leading to 42–55), often by exchanging the halides found in various positions.^38,44–46^ Asides from derivatization on the silicon atom, the CH_2_ moiety can also be a target for lithiation to give stepwise addition of longer C/Si chains (56–60).^47^ Lastly, it was also shown that the ligand of a single Si site can be abstracted to obtain a charged cluster cation [(SiMe)_3_Si(CH_2_)_6_][CHB_11_Cl_11_] (61) by reacting the carbocation [Ph_3_C]^+^ with [(SiMe)_3_SiH(CH_2_)_6_] (49).^48^

Realizing the first purely Si based adamantanes took a 9 step synthesis, the last one being a rearrangement of a tricyclic compound Si_14_Me_24_ to [(SiMe)_4_(SiMe_2_)_6_] (62, Fig. 4) reminiscent of a synthesis route to organic adamantanes by Schleyer (see section 2.2).^49^ In recent times, the topic has been reinvestigated, resulting in a streamlined gram scale synthesis method, and strategies for a site selective functionalization, which can lead to one or more methyl groups being substituted at the Q position (63–81).^50^

While the pure silaadamantanes were not obtainable from simple building blocks, compounds with mixed Ge/Si sites were isolated by a mixture of Me_2_GeCl_2_, Si_2_Cl_6_ and [^*n*^Bu_4_N]Cl, leading to [(SiSiCl_3_)_4_(GeMe_2_)_6−*x*_(SiCl_2_)_*x*_] (82–84, *x* = 0–2, Fig. 4), with the amount of Ge incorporated rising with the use of higher amounts of [^*n*^Bu_4_N]Cl.^51^ In follow up investigations, site selective methylation at the Q position of these compounds was realized using the Grignard reagent MeMgBr (85–87).^52^ It was also possible to obtain the corresponding stannasilaadamantanes [(SiSiCl_3_)_4_(SnMe_2_)_6−*x*_(SiCl_2_)_*x*_] ((88–89, *x* = 1–2) by substituting the Ge component for the higher homolog Me_2_SnCl_2_ in the reaction.

**Table 4:** Adamantane-type compounds with group 14 elements in the Q position and group 14 atoms in the E position^a^

Compound	Reagents/conditions	Method
[(SiCl)_4_(CH_2_)_6_] (39)	SiCl_4_, Me_3_SiCl, AlCl_3_/500 °C or [(SiMe)_4_(CH_2_)_6_], Cl_2_, I_2_/CCl_4_	B/Q^37,39,42^
[(SiMe)_4_(CH_2_)_6_] (40)	SiMe_4_/700 °C or (Me_2_SiCH_2_)_3_, AlBr_3_/100 °C	A^38,40,41^
[(SiH)_4_(CH_2_)_6_] (41)	Li[AlH_4_], [(SiMe)_4_(CH_2_)_6_] (40)	Q^39^
[(SiMe)_3_SiBr(CH_2_)_6_] (42)	(Me_2_SiCH_2_)_3_, AlBr_3_/100 °C	J^41,43^
[(SiMe)_2_(SiBr)_2_(CH_2_)_6_] (43)	(Me_2_SiCH_2_)_3_, AlBr_3_/100 °C	J^41^
[(SiMe)_3_SiCl(CH_2_)_6_] (44)	(Me_2_SiCH_2_)_3_, AlCl_3_/100 °C	J^41^
[(SiMe)_2_(SiCl)_2_(CH_2_)_6_] (45)	(Me_2_SiCH_2_)_3_, AlCl_3_/100 °C	J^41^
[SiMe(SiCl)_3_(CH_2_)_6_] (46)	[(SiMe)_4_(CH_2_)_6_] (40), Cl_2_, I_2_/CCl_4_	Q^42^
[(SiMe)_3_SiOH(CH_2_)_6_] (47)	[(SiMe)_3_SiCl(CH_2_)_6_] (44), [NBu_4_]Cl, KOH/2-methyl-2-butanol, H_2_O, 80 °C, 30 min	Q^44^
[(SiMe)_3_SiOCH_2_CH_2_NMe_2_(CH_2_)_6_] (48)	[(SiMe)_3_SiCl(CH_2_)_6_] (44), HOCH_2_CH_2_NMe_2_, *n*-BuLi/hexane, 69 °C, 9 h	Q^44^
[(SiMe)_3_SiH(CH_2_)_6_] (49)	[(SiMe)_3_SiBr(CH_2_)_6_] (42), Li[AlH_4_]/Et_2_O, 35 °C, 4 days	Q^44^
[(SiMe)_3_SiNEt_2_(CH_2_)_6_] (50)	[(SiMe)_3_SiCl(CH_2_)_6_] (44), LiNEt_2_/hexane, 24 h	Q^44^
[(SiMe)_3_SiPh(CH_2_)_6_] (51)	[(SiMe)_3_SiCl(CH_2_)_6_] (44), LiPh/Et_2_O	Q^45^
[(SiMe)_3_SiOMe(CH_2_)_6_] (52)	[(SiMe)_3_SiBr(CH_2_)_6_] (42), NaOMe,/MeOH	Q^45^
[(SiMe)_3_SiF(CH_2_)_6_] (53)	[(SiMe)_3_SiBr(CH_2_)_6_] (42), c-C_6_H_11_NH_3_F,/CHCl_3_	Q^45^
[(SiMe)_3_SiOTf(CH_2_)_6_] (54)	[(SiMe)_4_(CH_2_)_6_] (40), ICl, AgOTf/CH_2_Cl_2_, 1 day	Q^46^
[(SiOTf)_2_(SiMe)_2_(CH_2_)_6_] (55)	[(SiMe)_3_SiOTf(CH_2_)_6_] (54), ICl, AgOTf/CH_2_Cl_2_, 24 h	Q^46^
[(SiMe)_4_(CH_2_)_5_CHSiMe_2_Ph] (56)	[(SiMe)_4_(CH_2_)_6_] (40), ClSiMe_2_Ph, *n*-BuLi, KOCMe_3_/THF, 0 °C, 10 h	Q^47^
[(SiMe)_4_(CH_2_)_5_CHSiMe_2_CH_2_SiMe_2_Ph] (57)	[(SiMe)_4_(CH_2_)_5_CHSiMe_2_Ph] (56), Br_2_, LiCH_2_SiMe_2_Ph/	Q^47^
[(SiMe)_4_(CH_2_)_5_CHSiMe_2_CH_2_SiMe_2_CH_2_SiMe_3_] (58)	[(SiMe)_4_(CH_2_)_6_] (40), Me_3_SiCH_2_SiMe_2_CH_2_SiMe_2_Br, *n*-BuLi, TMEDA/hexane, 40 °C, 5 h	Q^47^
[(SiMe)_3_(CH_2_)_5_CH(SiMe_2_CH_2_SiMe_2_CH_2_)Si] (59)	[(SiMe)_4_(CH_2_)_5_CHSiMe_2_CH_2_SiMe_2_CH_2_SiMe_3_] (59), AlBr_3_/30 °C, 20 h	Q^47^
[(SiBr)(SiMe)_2_(CH_2_)_5_CH(SiMe_2_CH_2_SiMe_2_CH_2_)Si] (60)	[(SiMe)_4_(CH_2_)_5_CHSiMe_2_CH_2_SiMe_2_CH_2_SiMe_3_] (59), AlBr_3_/30 °C, 20 h	Q^47^
[(SiMe)_3_Si(CH_2_)_6_][CHB_11_Cl_11_] (61)	[Ph_3_C][CHB_11_Cl_11_], [(SiMe)_3_SiH(CH_2_)_6_] (49)/PhBr	Q^48^
[(SiSiMe_3_)_4_(SiMe_2_)_6_] (62)	Si_14_Me_24_, [CPh_3_][B(C_6_F_5_)_4_]/Toluene, 48 h	J^49,50^
[(SiSiMe_3_)_4_(SiMe_2_)_5_(SiMeCl)] (63)	Si_14_Me_24_, AlCl_3_, MeI, Me_3_SiCl/C_6_H_6_, 48 h	J^50^
[(SiSiMe_3_)_4_(SiMe_2_)_5_(SiMeBr)] (64)	Si_14_Me_24_, AlBr_3_, MeI, Me_3_SiBr/C_6_H_6_, 17 days	J^50^
[(SiSiMe_2_Cl)(SiSiMe_3_)_3_(SiMe_2_)_6_] (65)	1. [(SiSiMe_3_)_4_(SiMe_2_)_6_] (62), KOCMe_3_, 18-crown-6/toluene, 16 h	Q^50^
	2. Me_2_SiCl_2_/1 h	
[(SiSiMe_2_Ph)(SiSiMe_3_)_3_(SiMe_2_)_6_] (66)	1. [(SiSiMe_3_)_4_(SiMe_2_)_6_] (62), KOCMe_3_, 18-crown-6/toluene, 16 h	Q^50^
	2. Me_2_PhSiCl/3 h	
[(SiSiPh_3_)(SiSiMe_3_)_3_(SiMe_2_)_6_] (67)	1. [(SiSiMe_3_)_4_(SiMe_2_)_6_] (62), KOCMe_3_, 18-crown-6/toluene, 16 h	Q^50^
	2. Ph_3_SiCl/3 h	
[(SiSnMe_3_)(SiSiMe_3_)_3_(SiMe_2_)_6_] (68)	1. [(SiSiMe_3_)_4_(SiMe_2_)_6_] (62), KOCMe_3_, 18-crown-6/toluene, 16 h	Q^50^
	2. Me_3_SnCl/3 h	
[(SiGeMe_3_)(SiSiMe_3_)_3_(SiMe_2_)_6_] (69)	1. [(SiSiMe_3_)_4_(SiMe_2_)_6_] (62), KOCMe_3_, 18-crown-6/toluene, 16 h	Q^50^
	2. Me_3_GeCl/3 h	
[(SiH)(SiSiMe_3_)_3_(SiMe_2_)_6_] (70)	1. [(SiSiMe_3_)_4_(SiMe_2_)_6_] (62), KOCMe_3_, 18-crown-6/toluene, 16 h	Q^50^
	2. HCl/3 h	
[{SiP(NET_2_)_2_}(SiSiMe_3_)_3_(SiMe_2_)_6_] (71)	1. [(SiSiMe_3_)_4_(SiMe_2_)_6_] (62), KOCMe_3_, 18-crown-6/toluene, 16 h	Q^50^
	2. P(NET_2_)_2_Cl/3 h	
[(SiCH_2_SMe)(SiSiMe_3_)_3_(SiMe_2_)_6_] (72)	1. [(SiSiMe_3_)_4_(SiMe_2_)_6_] (62), KOCMe_3_, 18-crown-6/toluene, 16 h	Q^50^
	2. ClCH_2_SMe/3 h	
[(SiMe)(SiSiMe_3_)_3_(SiMe_2_)_6_] (73)	1. [(SiSiMe_3_)_4_(SiMe_2_)_6_] (62), KOCMe_3_, 18-crown-6/toluene, 16 h	Q^50^
	2. Methyl-*p*-toluenesulfonate/3 h	
[(SiBr)(SiSiMe_3_)_3_(SiMe_2_)_6_] (74)	1. [(SiSiMe_3_)_4_(SiMe_2_)_6_] (62), KOCMe_3_, 18-crown-6/toluene, 16 h	Q^50^
	2. 1,2-Dibromoethane/3 h	
[(SiCl)(SiSiMe_3_)_3_(SiMe_2_)_6_] (75)	1. [(SiSiMe_3_)_4_(SiMe_2_)_6_] (62), KOCMe_3_, 18-crown-6/toluene, 16 h	Q^50^
	2. PCl_3_/−78 °C, 3 h	
[(SiCH_2_SMe)_2_(SiSiMe_3_)_2_(SiMe_2_)_6_] (76)	1. [(SiSiMe_3_)_4_(SiMe_2_)_6_] (62), KOCMe_3_, 18-crown-6/toluene, 16 h	Q^50^
	2. ClCH_2_SMe/3 h	
[(SiCH_2_SMe)_3_(SiSiMe_3_)(SiMe_2_)_6_] (77)	1. [(SiSiMe_3_)_4_(SiMe_2_)_6_] (62), KOCMe_3_, 18-crown-6/toluene, 16 h	Q^50^
	2. ClCH_2_SMe/3 h	
[(SiCH_2_SMe)_4_(SiMe_2_)_6_] (78)	1. [(SiSiMe_3_)_4_(SiMe_2_)_6_] (62), KOCMe_3_, 18-crown-6/toluene, 16 h	Q^50^
	2. ClCH_2_SMe/3 h	
[(SiMe)(Si^i^Pr)(SiSiMe_3_)_2_(SiMe_2_)_6_] (79)	1. [(SiMe)(SiSiMe_3_)_3_(SiMe_2_)_6_] (62), KOCMe_3_, 18-crown-6/toluene, 16 h	Q^50^
	2.Chlorotriisopropylsilane/3 h	
[(SiMe)(Si^i^Pr)(SiCH_2_SMe) (SiSiMe_3_)(SiMe_2_)_6_] (80)	1. [(SiMe)(Si^i^Pr)(SiSiMe_3_)_2_(SiMe_2_)_6_] (79), KOCMe_3_, 18-crown-6/toluene, 16 h	Q^50^
	2.ClCH_2_SMe/3 h	
[(SiMe)(Si^i^Pr)(SiCH_2_SMe)(SiBr)(SiMe_2_)_6_] (81)	1. [(SiMe)(Si^i^Pr)(SiCH_2_SMe) (SiSiMe_3_)(SiMe_2_)_6_] (80), KOCMe_3_, 18-crown-6/toluene, 16 h	Q^50^
	2. 1,2-Dibromoethane/3 h	
[(SiSiCl_3_)_4_(GeMe_2_)_4_(SiCl_2_)_2_] (82)	Me_2_GeCl_2_, Si_2_Cl_6_, [Bu_4_N]Cl/CH_2_Cl_2_, 13 days	C^51^
[(SiSiCl_3_)_4_(GeMe_2_)_5_(SiCl)] (83)	Me_2_GeCl_2_, Si_2_Cl_6_, [Bu_4_N]Cl/CH_2_Cl_2_, 19 days	C^51^
[(SiSiCl_3_)_4_(GeMe_2_)_6_] (84)	Me_2_GeCl_2_, Si_2_Cl_6_, [Bu_4_N]Cl/CH_2_Cl_2_, 60 °C, 6 days	C^51^
[(SiSiMe_3_)_4_(GeMe_2_)_6_] (85)	[(SiSiCl_3_)_4_(GeMe_2_)_6_] (85), MeMgBr/Et_2_O, 60 °C, 1 day	Q^52^
[(SiSiMe_3_)_4_(GeMe_2_)_4_(GeMe_2_)_2_] (86)	[(SiSiCl_3_)_4_(GeMe_2_)_4_(GeMe_2_)_2_] (86), MeMgBr/Et_2_O, 60 °C, 1 day	Q^52^
[(SiSiMe_3_)_4_(GeMe_2_)_5_(GeMe_2_)] (87)	[(SiSiCl_3_)_4_(GeMe_2_)_5_(GeMe_2_)] (87), MeMgBr/THF, Et_2_O, 1 day	Q^52^
[(SiSiCl_3_)_4_(SnMe_2_)_4_(SiCl_2_)_2_] (88)	Me_2_SnCl_2_, Si_2_Cl_6_, [Bu_4_N]Cl/CH_2_Cl_2_, 3 days	Q^52^
[(SiSiCl_3_)_4_(SnMe_2_)_5_(SiCl_2_)] (89)	[(SiSiCl_3_)_4_(SnMe_2_)_4_(SiCl_2_)_2_] (88), [Bu_4_N]Cl/CH_2_Cl_2_, 60 °C, 1 day	Q^52^

a TMEDA = tetramethylethylenediamine, ^i^Pr = isopropyl.

##### 2.1.3.5 Group 15/14 adamantane-type clusters

[P_4_(SiR_2_)_6_] (90–95) adamantane-type clusters with different ligands R are formed by adding Cl_2_SiR_2_ to a solution of Na, K and P_4_.^53–56^ A route to mixtures of such compounds with a heterogeneous ligand sphere 
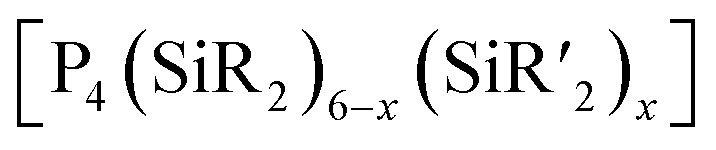
 (90–92, 96–105) is by the thermolysis of 
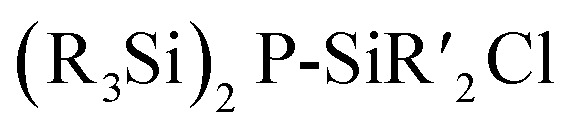
.^57^

**Fig. 5:**
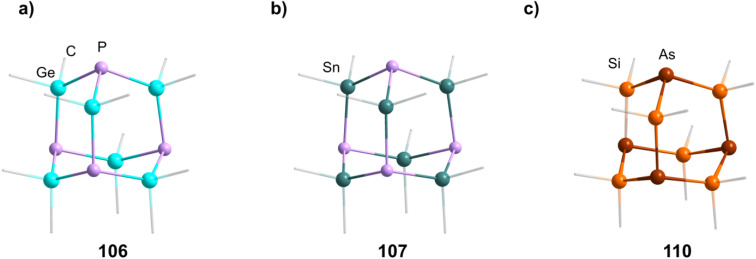
Examples of adamantane-type compounds with group 15 elements in the Q position and group 14 atoms in the E position: [P_4_(GeMe_2_)_6_] (106, left (a)), [P_4_(SnMe_2_)_6_] (107, center (b)) and [As_4_(SiMe_2_)_6_] (110, right (c)). Hydrogen atoms are omitted for clarity.

The germanium compound [P_4_(GeMe_2_)_6_] (106, Fig. 5) is obtained by a Hg catalyzed reaction of Cl_2_GeMe_2_,^58,59^ while the heaviest congeners [P_4_(SnR_2_)_6_] (107–109) were first suggested to be detected as a side product in the condensation reaction of PH_3_ and R_2_SnCl_2_.^60^ The first larger yield synthesis and crystallographic investigation of 107 (Fig. 5) was carried out after an unexpected rearrangement of P(SnMe_3_)_3_ catalyzed by [(ZnCl)_2_Fe(CO)_4_(THF)_2_] was observed.^61^

The analogous [As_4_(SiMe_2_)_6_] (110, Fig. 5) is only found as a side product in the thermolysis of Me_2_Si(AsSiMe_3_)_2_.^62,63^

**Table 5:** Adamantane-type compounds with group 15 elements in the Q position and group 14 atoms in the E position^a^

Compound	Reagents/conditions	Method
[P_4_(SiR_2_)_6_] (90–95, R_2_ = Me_2_, MeEt, Et_2_, MePh, (Me)(C_2_H_3_), MeH)	1. P_4_, K, Na,/DME, −78 °C	D^53–56^
	2. Cl_2_SiR_2_/DME, 24 h	
[P_4_(SiMe_2_)_6−*x*_(SiEt_2_)_*x*_] (90, 96–100, 92, *x* = 0–6)	(Me_3_Si)_2_P–SiEt_2_Cl/300 °C	H^57^
[P_4_(SiMe_2_)_6−*x*_(SiMeEt)_*x*_] (90, 101–105, 91, *x* = 0–6)	(Me_3_Si)_2_P–SiMeEtCl/300 °C	H^57^
[P_4_(GeMe_2_)_6_] (106)	Me_2_Ge(PH_2_)_2_, Hg/100 °C, 24 h	H^58,59^
[P_4_(SnR_2_)_6_] (107–109, R = Me, ^*n*^Bu, Ph)	R_2_SnCl_2_, PH_3_/or P(SnMe_3_)_3_, [(ZnCl)_2_Fe(CO)_4_(thf)_2_]/THF, 4 days	D/J^60,61^
[As_4_(SiMe_2_)_6_] (110)	Me_2_Si(AsSiMe_3_)_2_/240 °C, 48 h	H^62,63^

a DME = 1,2-dimethoxyethane.

#### 2.1.4 Q/group 15 adamantane-type clusters

Compounds with group 15 atoms in the E position are much rarer than those of the neighboring groups. They are spread around the periodic table with examples known in combination with the elements of groups 2, 8 and 11–15, of which the group 15/15 combination is the most common, comprising nearly half of all known species. Adamantane like scaffolds are only found for the lowest homologues, with NR_2_, NR, PR_2_ or PR making up the bulk of the known groups in the E position. Approaches to obtain those compounds are very diverse, with no unifying method between the different groups.

##### 2.1.4.1 Group 2/15 adamantane-type clusters

**Fig. 6:**
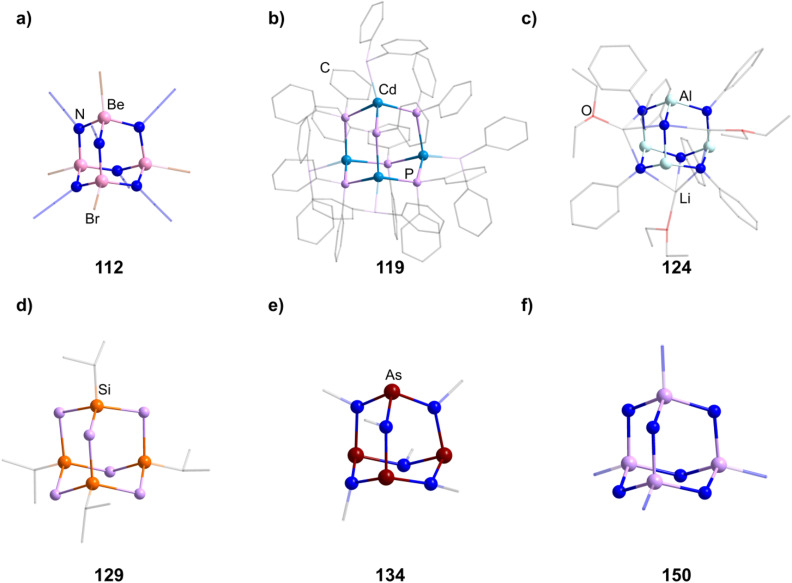
Examples of adamantane-type compounds with group 15 atoms in E position: (Ph_4_P)_2_[(Be_4_Br)_4_(N_3_)_6_] (112, top left (a)), [Li(thf)_4_]_2_[(CdPPh_2_)_4_(PPh_2_)_6_] (119, top center (b)), [Li(OEt_2_)_3_][(HAl)_4_(NPh)_6_{Li(OEt_2_)}_3_] (124, top right (c)), [(^i^PrSi)_4_(PH)_6_] (129, bottom left (d)), [As_4_(NMe)_6_] (134,bottom center (e)) and Na_10_[P_4_(NH)_6_N_4_](NH_2_)_6_(NH_3_)_0.5_ (150, bottom right (f)). Hydrogen atoms and counterions, if present, are omitted for clarity.

Two studies have investigated Be/N adamantane-type clusters. One publication found the anionic azide compounds [(BeX)_4_(N_3_)_6_] (111–112, Fig. 6) by reactions of Me_3_SiN_3_ with (Ph_4_P)_2_[Be_2_X_6_].^64^ The other investigated the formation of amido adamantanes [(BeNH_3_)_4_(NH_2_)_6_]^2+^ (in compounds 113–118) in liquid ammonia from elemental Be with varying counterions.^65^

##### 2.1.4.2 Transition metal/15 adamantane-type clusters

There are only a few examples of group 15 containing adamantanes with transition metals. Two of them can be formed by the addition of Ph_2_PH to a metal salt in the presence of ^*n*^BuLi to yield [Li(thf)_4_]_2_[(CdPPh_2_)_4_(PPh_2_)_6_] (119, Fig. 6) or [Li(thf)_4_]_2_[Cu_4_(PPh_2_)_6_] (120) depending on the element used.^66,67^ Two neutral iron clusters with a [Fe_4_]^6+^ core, comprising iron centers in a formal oxidation state of +1.5 were investigated.^68,69^ One could be obtained with a phosphide ligand, [Fe_4_(P^i^Pr_2_)_6_] (121), and the other with a ketimide ligand, [Fe_4_(N=CPh_2_)_6_] (122). Both are prepared in the same way as the Cd and Cu adamantanes by reacting the lithiated ligand with a metal salt.

##### 2.1.4.3 Group 13/15 adamantane-type clusters

[(NMe_2_)_2_AlCl]_2_ can dimerize under elimination of NMe_3_ to form [(AlCl)_4_(NMe_2_)_4_(NMe)_2_] (123), with higher yields achieved in the presence of B_2_(NMe_2_)_4_.^70,71^ An anionic derivative [(HAl)_4_(NPh)_6_{Li(OEt_2_)}_3_]^−^ (in 124, Fig. 6) featuring a Li capped adamantane is prepared by the combination of PhN(H)Li and AlH_3_.^72^

The only known Al/P adamantane compound, [(Ar^Me6^Al)_4_(PH_2_)_4_(PH)_2_] (125), is isolated after the reaction of PH_3_ with [Ar^Me6^AlH_2_]_2_.^73^ An example for Ga in the Q position [(PhGa)_4_(NH^i^Bu)_4_(N^i^Bu)_2_] (126) can be synthesized from [PhGa(NMe_2_)_2_]_2_ and H_2_N^i^Bu.^74^

##### 2.1.4.4 Group 14/15 adamantane-type clusters

Group 14/15 adamantanes have been investigated for E = P. [(PhSi)_4_(PPh)_6_] (127) and its germanium congener (128) are obtainable by a simple condensation reaction of PhQCl_3_ and K_2_PPh.^75^ The same principle can be used for the synthesis of [(^i^PrSi)_4_(PH)_6_] (129, Fig. 6).^76^ Another synthesis method, utilizing preformed fragments [Li(tmeda)]_2_[C_6_H_4_(PSiMe_3_)_2_-1,2] with Si^*t*^BuCl_3_, leads to the formation of the asymmetrical [(C_6_H_4_{P(Si^*t*^BuP)1,2})_3_(Si^*t*^Bu)] (130).^77^

##### 2.1.4.5 Group 15/15 adamantane-type clusters

Compounds of the type [P_4_(NR)_6_] form the vast majority of clusters within this group combination. They are mostly obtained by condensation reactions of PCl_3_ with RNH_2_ (131–133),^78–81^ a synthesis strategy which also works when substituting PCl_3_ for AsCl_3_ to form the lesser investigated congeners [As_4_(NR)_6_] (134–136, Fig. 6).^82,83^ Notably, another method of achieving an adamantane-type topology is a reaction starting from a precursor featuring a P_2_N_2_ four membered ring, ClP(N^i^Pr)_2_PN^i^PrSiMe_3_, which dimerizes when heated to form the so called double decker-type cluster [P_4_(N^i^Pr)_6_], an isomer to the adamantane-type architecture consisting of two four membered rings bridged by two bridging atoms.^84,85^ This cluster will in turn rearrange to the adamantane compound (137); an isomerization that also plays a major role in the chemistry of group 14/16 adamantane-type structures and for one Mn/O cluster. The same rearrangement from the double decker was required to form [P_4_(N^*t*^Bu)_6_] (138), albeit that ball milling was needed instead of higher temperatures to prompt the rearrangement.^86^

These compounds can be used as precursors in ligand addition reactions to the pnictogen. The first one investigated was the addition of MeI resulting in [P_3_(PMe)(NMe)_6_Me]I (139).^78,79,87^ Adding S or O atoms in the form of Me_3_NO or elemental sulfur leads to [(OP)_4_(NR)_6_] (140) or [(SP)_4_(NR)_6_] (141) respectively. The addition of sulfur can be carried out stepwise to achieve the desired degree of sulfurization (142–145).^80,88–93^ The addition of transition metal moieties was also realized by reactions with [Ni(CO)_4_] to 131 and 134, resulting in adamantanes with terminal Ni(CO)_3_ groups (146 and 147).^94^ The ligand sphere on the phosphorous atom can also be expanded stepwise by introducing a SiMe_3_ group in [(PNSiMe_3_)_4_(NMe)_6_] (148), which can subsequently be exchanged for PPh_3_ (149).^95^ Lastly, purely inorganic and anionic clusters were obtained by the rearrangement of P_3_N_5_ with addition of alkaline metal NH_2_ salts to yield cluster cores [(PN)_4_N_6_] (150–151, Fig. 6) with different degrees of protonation.^96,97^

**Table 6:** Adamantane-type compounds with group 15 atoms in E position^a^

Compound	Reagents/conditions	Method
(Ph_4_P)_2_[(BeCl)_4_(N_3_)_6_] (111)	Me_3_SiN_3_, (Ph_4_P)_2_[Be_2_Cl_6_]/CH_2_Cl_2_, 2 days	C^64^
(Ph_4_P)_2_[(Be_4_Br)_4_(N_3_)_6_] (112)	Me_3_SiN_3_, (Ph_4_P)_2_[Be_2_Br_6_]/CH_2_Br_2_, 2 days	C^64^
[(BeNH_3_)_4_(NH_2_)_6_]Cl_2_ (113)	BeCl_2_, Be/NH_3_, 2 days	B^65^
[(BeNH_3_)_4_(NH_2_)_6_]Br_2_ (114)	BeBr_2_, Be/NH_3_, 2 days	B^65^
[(BeNH_3_)_4_(NH_2_)_6_]I_2_ (115)	NH_4_I, Be/NH_3_, 29 days	B^65^
[(BeNH_3_)_4_(NH_2_)_6_](CN)_2_ (116)	Me_3_SiCN, Be/NH_3_, 2 days	B^65^
[(BeNH_3_)_4_(NH_2_)_6_](SCN)_2_ (117)	NH_4_SCN, Be/NH_3_, 4 days	B^65^
[(BeNH_3_)_4_(NH_2_)_6_](N_3_)_2_ (118)	Me_3_SiN_3_, Be/NH_3_, 4 days	B^65^
[Li(thf)_4_]_2_[(CdPPh_2_)_4_(PPh_2_)_6_] (119)	^*n*^BuLi, Ph_2_PH, [Cd{N(SiMe_3_)_2_}_2_]/THF, 80 °C to RT, 12 h	C^66^
[Li(thf)_4_]_2_[Cu_4_(PPh_2_)_6_] (120)	^*n*^BuLi, Ph_2_PH, CuCN/toluene, −78 °C	C^67^
[Fe_4_(P^i^Pr_2_)_6_] (121)	[FeBr_2_(thf)_2_], ^i^Pr_2_PLi/DME, RT	C^68^
[Fe_4_(N=CPh_2_)_6_] (122)	FeBr_2_, LiN=CPh_2_, Zn/THF, −25 °C to RT, 18 h	C^69^
[(AlCl)_4_(NMe_2_)_4_(NMe)_2_] (123)	(NMe_2_)_2_AlCl, B_2_(NMe_2_)_4_/240 °C, 10 h	B^70,71^
[Li(OEt_2_)_3_][(HAl)_4_(NPh)_6_{Li(OEt_2_)}_3_] (124)	PhN(H)Li, H_3_Al·N(Me)C_5_H_8_/Et_2_O	D^72^
[(Ar^Me6^Al)_4_(PH_2_)_4_(PH)_2_] (125)	(Ar^Me6^AlH_2_)_2_, PH_3_/toluene, 80 psi, 24 h	G^73^
[(PhGa)_4_(NH^i^Bu)_4_(N^i^Bu)_2_] (126)	[PhGa(NMe_2_)_2_]_2_, H_2_N^i^Bu/2 h	C^74^
[(PhSi)_4_(PPh)_6_] (127)	PhSiCl_3_, K_2_PPh/C_6_H_6_, Et_2_O, DME, 10 h	D^75^
[(PhGe)_4_(PPh)_6_] (128)	PhGeCl_3_, K_2_PPh/C_6_H_6_, Et_2_O, DME, 10 h	D^75^
[(^i^PrSi)_4_(PH)_6_] (129)	Li[Al(PH_2_)_4_], ^i^PrSiCl_3_/1,2-DME, −30 °C, 3 h	C^76^
[(C_6_H_4_{P(Si^*t*^BuP)1,2})_3_(Si^*t*^Bu)] (130)	[Li(tmeda)]_2_[C_6_H_4_(PSiMe_3_)_2_-1,2], Si^*t*^BuCl_3_/THF, −78 °C	J^77^
[P_4_(NMe)_6_] (131)	MeNH_2_, PCl_3_/−78 °C to RT, 4 days	G^78,79^
[P_4_(NEt)_6_] (132)	PCl_3_, EtNH_2_/−60 °C to 150 °C	G^80,81^
[P_4_(NBn)_6_] (133)	PCl_3_, ^*n*^BuLi, BnNH_2_ NEt_3_/THF, −60 °C to RT, 5 days	D^81^
[As_4_(NMe)_6_] (134)	AsCl_3_, MeNH_2_/C_6_H_6_, 0 °C, 1 h	G^82,83^
[As_4_(N^i^Pr)_6_] (135)	AsCl_3_, ^i^PrNH_2_/pentane, 1 h	D^82^
[As_4_(N^*n*^Bu)_6_] (136)	AsCl_3_, ^*n*^BuNH_2_/C_6_H_6_, 60 °C, 30 min	D^82^
[P_4_(N^i^Pr)_6_] (137)	1. ClP(N^i^Pr)_2_PN^i^PrSiMe_3_/MeCN, 82 °C, 15 h	K^84,85^
	2. 158 °C, 3 days	
[P_4_(N^*t*^Bu)_6_] (138)	[P_4_(N^*t*^Bu)_6_] (double decker isomer), LiCl/ball milling, 90 min	M^86^
[P_3_(PMe)(NMe)_6_Me]I (139)	[P_4_(NMe)_6_] (131), MeI/0 °C	P^78,79,87^
[(SP)_4_(NEt)_6_] (140)	[P_4_(NEt)_6_] (132), S/toluene, 95 °C, 9 h	P^80^
[(OP)_4_(NMe)_6_] (141)	[P_4_(NMe)_6_] (131), Me_3_NO/C_6_H_6_, 12 h	P^90,91^
[P_*n*_(SP)_4−*n*_(NMe)_6_] ((142–145, *n* = 1–4)	[P_4_(NMe)_6_] (131), S or [P_4_(NMe)_6_] (131), S/CS_2_, −20 °C, 12 h	P^88–93^
[{(CO)_3_NiP}_4_(NMe)_2_] (146)	[P_4_(NMe)_6_] (131), [Ni(CO)_4_]/3 h	P^94^
[{(CO)_3_NiAs}_4_(NMe)_2_] (147)	[As_4_(NMe)_6_] (134), [Ni(CO)_4_]/CHCl_3_, 3 h, 5 min	P^94^
[(PNSiMe_3_)_4_(NMe)_6_] (148)	[P_4_(NMe)_6_] (131), Me_3_SiN_3_/toluene, 100 °C, 12 weeks	P^95^
[(PNPPh_3_)_4_(NMe)_6_] (149)	[(PNSiMe_3_)_4_(NMe)_6_] (148), Ph_3_PBr_2_/MeCN, 55 °C, 3 days	P^95^
Na_10_[P_4_(NH)_6_N_4_](NH_2_)_6_(NH_3_)_0.5_ (150)	P_3_N_5_, NaNH_2_/600 °C, 5 days	A^96^
Rb_8_[(PNH)_4_N_6_](NH_2_)_2_ (151)	P_3_N_5_, RbNH_2_/400 C, 5 days	A^97^

a Ar^Me6^ = C_6_H_3_-2,6(C_6_H_2_-2,4,6-Me_3_)_2_.

#### 2.1.5 Q/group 16 adamantane-type clusters

A group 16 element is the most common atom in the E position of inorganic adamantane-type structures. Examples are known for all groups 2–15 (with the notable exception of monomeric group 10 adamantanes) as well as lanthanides. Most often, the chemistry of the oxo-adamantanes is quite different from its higher congeners, stemming from the unique properties of the elements in the second period. Groups 2 and 4–6 nearly exclusively feature compounds with O atoms in the E position, while the reverse case is observed in the groups 11, 12 and 14, which mainly comprise S, Se and Te. There are a few reoccurring structural motifs and synthetic approaches, especially for clusters with the heavier elements S, Se and Te. One family of chalcogenolate compounds [(QER)_4_(ER)_6_]^*q*^ with differing charges q can often be isolated from simple transition metal salts (groups 7–9 and 11–12) and deprotonated chalcogenols, either through *in situ* deprotonation or by using metal salts. A variant comprising halides X [(QX)_4_(ER)_6_]^*q*^ or other ligands in the X position is sometimes achievable by the choice of the correct precursor salt or counterion, as well as by exchanging a chalcogenolate in this terminal position.

Chalcogenide adamantane-type clusters of the general composition [(QR_0–3_)_4_E_6_]^*q*^ are found in a large family of compounds of the groups 13 and 14 as well as a single example with Ru. They are obtainable by condensation reactions using a metal (pseudo)halide and a chalcogenide source such as alkaline metal chalcogenides, H_2_E or (SiMe_3_)_2_E.

An additional family of purely inorganic adamantane-type clusters [Q_4_E_10_] is found for the groups 13–15. They are mostly accessible from the elements and simple salts by Methods A–C or by extracting alloys in accordance with Method E.

##### 2.1.5.1 Group 2/16 adamantane-type clusters

**Fig. 7:**
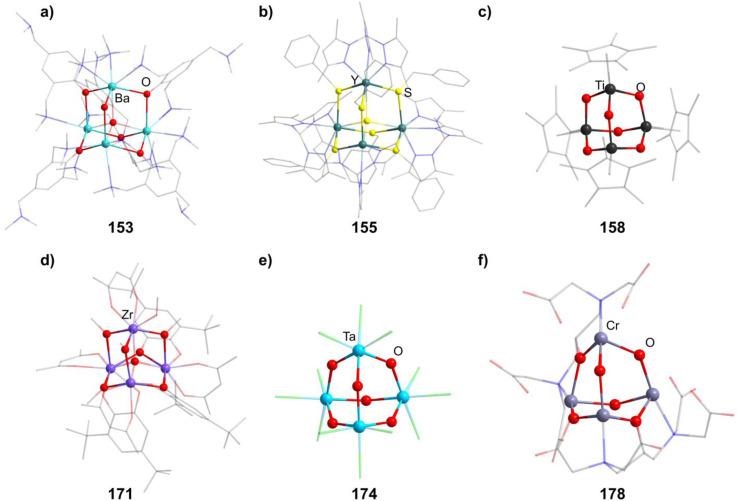
Examples of adamantane-type compounds with group 2–6 elements in Q position and group 16 atoms in the E position: [(μ_4_-O)Ba_4_(μ-OC_6_H_2_(CH_2_NMe_2_)_3_-2,4,6)_6_] (153, top left (a)), [(μ_4_-S)(Tp^Me2^Y)_4_(SBn)_6_] (155, top center (b)), [(TiCp*)_4_O_6_] (158, top right (c)), [(μ_4_-O){Zr(acac)}_4_{Zr(OMe)(acac)}(DBcat)_3_(OMe)_3_] (171, bottom left (d)), [K-18-crown-6]_4_[(TaCl_3_)_4_O_6_] (174,bottom center (e)) and (enH_2_)[Cr_4_(OH)_4_(hpdta)_2_] (178, bottom right (f)). Hydrogen atoms and counterions, if present, are omitted for clarity.

In group 2, a Be hydroxide cluster Na_2_[(BeOH)_4_(OH)_6_] (152) is reported to form from BeSO_4_ in basic aqueous solution.^98^ Two further oxygen centered species are obtainable with Ba. One, [(μ_4_-O)Ba_4_(μ-OC_6_H_2_(CH_2_NMe_2_)_3_-2,4,6)_6_] (153, Fig. 7), is formed with a tridentate ligand, which both delivers the oxygen in the E position and coordinates to the two closest barium atoms *via* nitrogen atoms.^99^ The other is obtained from a Ba dimer [Ba{N(SiMe_3_)_2_}_2_]_2_ assembling around (mes)_2_BOH to form [(μ_4_-O)Ba_4_{OB(mes)_2_}_6_] (147).^100^

##### 2.1.5.2 Group 3/16 adamantane-type clusters

[(μ_4_-S)(Tp^Me2^Y)_4_(SBn)_6_] (154, Fig. 7) is a unique compound in two ways, as it is both the only group 3 and S centered compound in this review.^101^ It is created by adding the Y complex [Tp^Me2^YBn_2_(thf)] with a tridentate ligand to elemental sulfur, which creates the cluster in a redox reaction.

##### 2.1.5.3 Group 4/16 adamantane-type clusters

All but one literature known compound in this category feature a Ti^IV^_4_O_6_ core. The first two examples were cationic in nature, [(TiL_3_)_4_O_6_]^4+^, with each Ti exhibiting three bonds to neutral ligands L. For [{Ti(TACN)}_4_O_6_]Br_4_ (156, TACN = 1,4,7-triazacyclononane), this was achieved by hydrolysis of TiO(acac)_2_ in the presence of TACN and NaBr,^102^ while the second example [{Ti(dmso)_3_}_4_O_6_]Cl_4_ (157) was generated in a solution of TiCl_4_, Na_2_S_4_ and PPh_3_ in DMSO under partial decomposition of the solvent to yield the required oxygen atoms.^103^

A larger family of neutral compounds contains derivatives of cyclopentadienyl at the Ti centers [(TiCp^R^)_4_O_6_] (158–164, 158 in Fig. 7), mainly obtained through hydrolysis of various Ti cyclopentadienyl complexes or through reactions with other O sources.^104–108^

More complex neutral clusters are isolated when the Ti_4_O_6_ is formally extended by additional M/O fragments. This could be observed for [Ti_4_(dmae)_6_(OH)(O)_6_Cu_6_(benzoate)_9_] (165, dmae = *N*,*N*-dimethylaminoethanolate) and its methyl derivative (166).^109^ They form from the respective hydrated Cu benzoates and Ti(dmae)_4_ in toluene and feature different coordination modes of the Cu/O fragments.

Two isostructural compounds [{Ti(thf)}_4_O_6_M_2_(TFA)_8_(thf)_2_] (167–168, M = Fe, Cd; TFA = trifluoroacetic acid) show a symmetric buildup, with the M centers being connected to opposing oxygen atoms in E position and *via* four TFA groups each to the neighboring Ti centers.^110,111^ They are obtained from [Fe_3_O(OAc)_6_(H_2_O)_3_]NO_3_ (OAc = acetate) or [(OAc)_2_Cd(H_2_O)_2_], and after addition of a Ti complex and TFA in THF.

A highly charged anion [Ti_4_O_6_(Hcit)_3_(cit)]^9−^ (in 169, H_4_cit = citric acid) is crystallized from a reactive solution of citric acid and [Ti{^i^PrO)_4_] in a H_2_O/THF mix. The addition of [Co(NH_3_)_6_]Cl_3_ yields the cobaltate salt, which can be converted to the Na analog (170) by ion exchange chromatography.^112^

[(μ_4_-O){Zr(acac)}_4_{Zr(OMe)(acac)}(DBcat)_3_(OMe)_3_] (171, acac = acetylacetonate, H_2_DBcat = 3,5-di-*tert*-butylcatechol, Fig. 7), hydrolytically obtained from [Zr_2_(acac)_4_(DBcat)_2_], is a singular Zr example in this group in which half of the E positions are occupied by methoxy groups and half of them by DBcat groups, which also coordinate to one Zr center each.^113^

##### 2.1.5.4 Group 5/16 adamantane-type clusters

There are only three unrelated examples of different group 5 oxides in this group.

The vanadium species [(VCp*)_4_O_6_] (172) stems from a rearrangement of the trimeric species [Cp*V(O)(μ-O)]_3_ after addition of PMe_2_Ph.^114^

The cluster compound [{HBO-3,5-(^*t*^Bu)_2_NbCl}_4_O_6_] (173, HBO = 2-(2′-Hydroxyphenyl)benzoxazole) is the simple hydrolysis product of [HBO-3,5-(^*t*^Bu)_2_NbCl_4_].^115^

Using a water containing sample of 18-crown-6 in a reaction of TaCl_5_ and K_2_S_5_ generates the heaviest congener [K-18-crown-6]_4_[(TaCl_3_)_4_O_6_] (174, Fig. 7) with an anionic cluster scaffold.^116^

##### 2.1.5.5 Group 6/16 adamantane-type clusters

Two cationic hydroxo clusters of the type [(CrR)_4_(OH)_6_]^*q*+^ (in 175–177) can be obtained by hydrolysis of Cr precursor complexes.^117,118^ In the case of a combination of CrCl_3_ and the pentadentate ligand hpdta (H_5_hpdta = hydroxypropanediaminotetraacetic acid), a compound with the cationic cluster [Cr_4_(μ-OH)_4_(hpdta)_2_]^2+^ (in 178, Fig. 7) was isolated, in which two of the oxygen atoms in E position stem from the hpdta ligands.^119^

The only known Mo congener [{MoO(IPAP)}_4_O_6_] (179, HIPAP = *N*-(*tert*-butyl)-3-((3,5-di-*tert*-butyl-2-hydroxybenzylidene)amino)-propanamide) is formed as a side product during the reduction of the complex [Mo(O)_2_(IPAP)_2_] using PPh_3_ and could only be isolated in trace amounts.^120^

Two structurally related oxo clusters of tungsten, [(W(O)(tdmap)}_4_O_6_] (180, tdmap = OC(CH2NMe_2_)_3_) and [{(W(O)(S-Phoz)}_4_O_6_] (181, S-Phoz = 2-(4′,4′-dimethyloxazoline-2′-yl)thiophenolate), are known in the literature.^121,122^ The first from a reaction of [W(O)(O^i^Pr)_4_] with Htdmap in the presence of water and the second by rearrangement of the complex [W(CO)(C_2_Me_2_)(S-Phoz)_2_] after oxidation using pyridine-*N*-oxide.

One sulfide containing adamantane-type cluster [(WPMe_2_Ph)_4_S_6_] (182) exists, which rearranges from the tetranuclear [W_4_(μ_3_-S)_2_(μ-S)_4_Cl_2_(PMe_2_Ph)_6_] after reduction with a Na/Hg amalgam in low yields.^123^

**Table 7:** Adamantane-type compounds with group 2–6 elements in the Q-position and group 16 atoms in the E-position^a^

Compound	Reagents/conditions	Method
Na_2_[(BeOH)_4_(OH)_6_] (152)	BeSO_4_, Ba(OH)_2_, NaOH/H_2_O, pH 13.2, 18 h	C^98^
[(μ_4_-O)Ba_4_(μ-OC_6_H_2_(CH_2_NMe_2_)_3_–2,4,6)_6_] (153)	K[(OC_6_H_2_(CH_2_NMe_2_)_3_-2,4,6), BaI_2_/toluene	K^99^
[(μ_4_-O)Ba_4_{OB(mes)_2_}_6_] (154)	(mes)_2_BOH, [Ba{N(SiMe_3_)_2_}_2_]_2_	C^100^
[(μ_4_-S)(Tp^Me2^Y)_4_(SBn)_6_] (155)	S, [Tp^Me2^YBn_2_(thf)]/THF, RT, 18 h	C^101^
[{Ti(TACN)}_4_O_6_]Br_4_ (156)	TiO(acac)_2_, 9aneN_3_, NaBr/Me_2_CO, H_2_O, 50 °C, 30 min	I^102^
[{Ti(dmso)_3_}_4_O_6_]Cl_4_ (157)	Na_2_S_4_, PPh_4_, TiCl_4_/DMSO, RT	C^103^
[(TiCp*)_4_O_6_] (158)	Cp*TiCl_3_, NH_4_OH/toluene, RT, 3 days or Cp*Ti(OMe)_3_/H_2_O, RT	I^104,105^
[(TiCp^*x*Ph^)_4_O_6_] (159)	Cp^*x*Ph^Ti(OME)_3_/Me_2_CO, H_2_O, 100 °C, 30 min	I^106^
[{Ti(η^5^-C_5_Me_4_SiMe_2_NHNMe_2_)}_4_O_6_] (160)	[(η^5^-C_5_Me_4_)SiMe_2_(NNMe_2_)]Ti(NMe)_2_/H_2_O, toluene, RT, 5 h	I^107^
[{Ti(OHF)}_4_O_6_] (161)	[(OHF)Ti(OMe)_3_]/Me_2_CO, H_2_O 56 °C	I^106^
[{Ti(η^5^-C_5_Me_4_SiMe_3_)}_4_O_6_] (162)	(η^5^-C_5_Me_4_SiMe_3_)_2_Ti(O)/pentane, RT, 2 weeks	J^108^
[{Ti(η^5^-C_5_Me_4_SiMe_2_Ph)}_4_O_6_] (163)	(η^5^-C_5_Me_4_SiMe_2_Ph)_2_Ti(O)/pentane, RT, 2 weeks	J^108^
[{Ti(η^5^-C_5_Me_4_^i^Pr)}_4_O] (164)	(η^5^-C_5_Me_4_^i^Pr)_2_Ti(O), Na_2_O_2_/THF, RT, overnight	I^108^
[Ti_4_(dmae)_6_(OH)(O)_6_Cu_6_(benzoate)_9_] (165)	Cu(benzoate)_2_·2H_2_O, Ti(dmae)_4_/toluene, RT, 2 h	C^109^
[Ti_4_(dmae)_6_(OH)(O)_6_Cu_6_(2-methylbenzoate)_9_] (166)	Cu(2-methylbenzoate)_2_·2H_2_O, Ti(dmae)_4_/toluene, RT, 2 h	C^109^
[{Ti(thf)}_4_O_6_Fe_2_(TFA)_8_(thf)_2_] (167)	[Fe_3_O(OAc)_6_(H_2_O)_3_]NO_3_, [(EtOEtO)_4_Ti], TFA/THF, RT, 1 h	J^110^
[{Ti(thf)}_4_O_6_Cd_2_(TFA)_8_(thf)_2_] (168)	[(OAC)_2_Cd(H_2_O)_2_], [Ti{^i^PrO)_4_], TFA/THF, RT, 4 h	C^111^
[Co(NH_3_)_6_]_3_[Ti_4_O_6_(Hcit)_3_(cit)] (169)	[Ti{^i^PrO)_4_], H_4_cit, [Co(NH_3_)_6_]Cl_3_/THF, H_2_O, 90 °C 1 h	I^112^
Na_9_[Ti_4_O_6_(Hcit)_3_(cit)] (170)	[Co(NH_3_)_6_]_3_[Ti_4_O_6_(Hcit)_3_(cit)] (169)/ion exchange chromatography	O^112^
[(μ_4_-O){Zr(acac)}_4_{Zr(OMe)(acac)}(DBcat)_3_(OMe)_3_] (171)	[Zr_2_(acac)_4_(DBcat)_2_]/CH_2_Cl_2_, MeOH, H_2_O, RT	I^113^
[(VCp*)_4_O_6_] (172)	[Cp*V(O)(μ-O)]_3_, PMe_2_Ph/THF	J^114^
[{HBO-3,5-(^*t*^Bu)_2_NbCl}_4_O_6_] (173)	HBO-3,5-(^*t*^Bu)_2_NbCl_4_, H_2_O/Toluene, THF, RT, 12 h	I^115^
[K-18-crown-6]_4_[(TaCl_3_)_4_O_6_] (174)	K_2_S_5_, TaCl_5_, 18-crown-6, H_2_O/CH_2_Cl_2_, RT, 20 h	I^116^
[(Cp*Cr)_4_(OH)_6_][Cp*Cr(CO)_3_] (175)	[(Cp*)_2_Cr_2_(CO)_4_]/H_2_O, toluene, 111 °C, 24 h	I^117^
[{(Cp*)Cr}_4_(OH)_6_][BF_4_]_2_ (176)	[(Cp*Cr)_4_(OH)_6_][Cp*Cr(CO)_3_] (175), H[BF_4_]	J^117^
[{Cr(tach)}_4_(OH)_6_](ClO_4_)_*n*_(CF_3_SO_3_)_6–*n*_ (177)	[Cr(tach)(CF_3_SO_3_)_3_], NaOH,/H_2_O	I^118^
(enH_2_)[Cr_4_(OH)_4_(hpdta)_2_] (178)	H_5_hpdta, en, CrCl_3_/H_2_O, 85 °C, 24 h	K^119^
[{MoO(IPAP)}_4_O_6_] (179)	1. HIPAP, [MoO_2_Br_2_(DMSO)_2_], NEt_3_, PMe_3_/MeOH, RT, 18 h	I^120^
	2. PMe_3_/toluene, RT, 18 h	
[{W(O)(tdmap)}_4_O_6_] (180)	[W(O)(OPr^i^)_4_], Htdmap/toluene, H_2_O, ^i^PrOH, reflux, 24 h	I^121^
[{(W(O)(S-Phoz)}_4_O_6_] (181)	[W(CO)(C_2_Me_2_)(S-Phoz)_2_], pyridine-*N*-Oxide/CH_2_Cl_2_, RT, 24 h	J^124^
[(WPMe_2_Ph)_4_S_6_] (182)	[W_4_(μ_3_-S)_2_(μ-S)_4_Cl_2_(PMe_2_Ph)_6_], Na(Hg)/THF, 8 h	J^123^

a mes = 2,4,6-Me_3_-C_6_H_2_, Tp^Me2^ = tri(3,5 dimethylpyrazolyl)borate), TACN = 1,4,7-triazacyclononane, DMSO = dimethyl sulfoxide, Cp^*x*Ph^ = C_5_Me_4_Ph, OHF = 1,2,3,4,5,6,7,8-octahydrofluorenyl, dmae = *N*,*N*-dimethylaminoethanolate, TFA = trifluoacetic acid, H_4_cit = citric acid, H_2_DBcat = 3,5-di-*tert*-butylcatechol, HBO = 2-(2′-hydroxyphenyl)benzoxazole, tach = 1,3,5-triaminocyclohexane, en = ethylendiamine, H_5_hpdta = hydroxypropanediaminotetraacetic acid, HIPAP = *N*-(*tert*-butyl)-3-((3,5-di-*tert*-butyl-2-hydroxybenzylidene)amino)-propanamide, tdmap = OC(CH2NMe_2_)_3_, S-Phoz = 2-(4′,4′-dimethyloxazoline-2′-yl)thiophenolate.

##### 2.1.5.6 Group 7/16 adamantane-type clusters

All known adamantane compounds with an elemental combination of groups 7/16 are Mn clusters in the oxidation state IV, either with oxygen or thiolates in the E position. The oxides are mainly available *via* hydrolysis and can be derivatized by ligand or ion exchange.

{Mn(TACN)}_4_O_6_]^4+^ (in 183–185) is the first example of such an adamantane-type structure synthesized by addition of simple Mn^II^ salts to TACN in the presence of water and air to oxidize the metal centers.^125–130^

The related adamantane [{Mn(bpea)}_4_O_6_](ClO_4_)_4_ (186, bpea = *N*,*N*-bis(2-pyridylmethyl)ethylamine) also comprises of an *N*,*N*,*N*-tridentate ligand and cannot be obtained by air oxidation, but requires a comproportionation of two Mn compounds Mn(ClO_4_)_2_ and [^*n*^Bu_4_N][MnO_4_] and bpea.^131^ Addition of [^*n*^Bu_4_N]Br yields the bromide salt [{Mn(bpea)}_4_O_6_]Br 187, which can subsequently be treated with alkaline metal salts for anion exchange (188–192). Methylated bpea can also be used during the synthesis to form derivatives (193–194). The same study also investigated the single electron reduction of the compounds under retention of the adamantane-type scaffold, either by electrochemistry or *via* TACNMe as a reducing agent (195).

[Mn_4_O_6_(bpea)_4_](ClO_4_)_4_ can also be used as a basis for ligand exchange using other tridentate ligands (196–200).^129^ In the case of the charged *N*-substituted iminodicarboxylate ligands, used as their ammonium salts, only partial substitiution products in the form of [{Mn(R-ida)}_2_{Mn(bpea)}_2_O_6_] (201–206, R-ida = *N*-(R)iminodiacetate) could be isolated as stable compounds.

By a reaction of tame·3HOTf (tame = *tert*-amyl methyl ether), Mn(OTf)_2_ and Et_3_N in MeCN and under exposure to athmosperic O_2_, the mixed oxo/hydroxo species [{Mn(tame)}_4_O_5_(OH)](OTf)_5_ (207) was obtained, which could be completely deprotonated by additional Et_3_N, leading to 208.^130^ Protonation of [{Mn(TACN)}_4_O_6_]^4+^ to the corresponding [{Mn(tame)}_4_O_5_(OH)]^5+^ (in 209) by HClO_4_ was also proven to work.

**Fig. 8:**
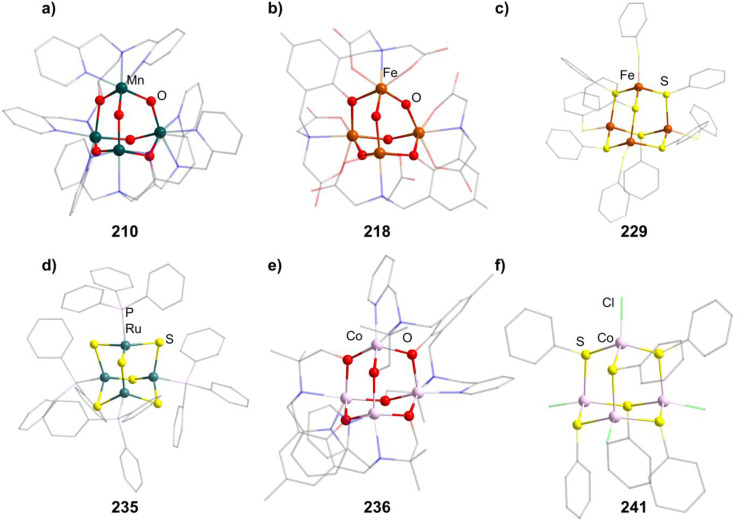
Examples of adamantane-type compounds with group 7–9 elements in the Q-position and group 16 atoms in the E-position: [Mn_4_O_4_(tphpn)_2_](CF_3_SO_3_)_2_(ClO_4_)_3_ (210, top left (a)), (HPy)_3_[{Fe_2_(HPhXCG)}_2_O(OH)_3_] (218, top center (b)), [Et_4_N]_2_[(FeSPh)_4_(SPh)_6_] (229, top right (c)), [(RuPPh_3_)_4_S_6_] (235, bottom left (d)), [Co_4_(HMPM)_2_](ClO_4_)_2_ (236,bottom center (e)) and [Et_4_N]_2_[{Co(Cl)}_4_(SPh)_6_] (241, bottom right (f)). Hydrogen atoms and counterions, if present, are omitted for clarity.

The last literature-known oxide cluster [Mn_4_O_4_(tphpn)_2_](OTf)_2_(ClO_4_)_3_ (210, Htphpn = *N*,*N*,*N*′,*N*′-tetra-(2-methylpyridyl)-2-hydroxypropanediamine, Fig. 8) features a Mn^III^/Mn^IV^ mixed valency situation and a pentadentate ligand bridging two Mn moieties by coordination with its N sites as well as the O atom in the E position between the two metal centers.^132^ It is prepared by a reductively induced isomerization of the double decker type compound [{Mn_2_(μ-O)_2_(tphpn)}_2_].

Thiolate complexes with Mn exhibiting adamantane-type structure have also been studied. In the most simple case, dianionic [(MnSPh)_4_(SPh)_6_]^2−^ clusters (in 211–212) are isolated after conversion of MnCl_2_ with NaSPh and an appropriate ammonium countercation.^133^ Unlike the oxygen species, they contain Mn^II^ sites in their inorganic core.

In those compounds, all Mn atoms also carry a thiolate ligand which can formally be substituted by halides by either using [Et_4_N]Br during the synthesis to form the brominated 213,^134^ or through a rearrangement by adding MnCl_2_ to [(Me_4_N)_2_{Mn_2_(S^i^Pr)_6_}], leading to [Me_4_N]_2_((MnCl)_4_(S^i^Pr)_6_] (214).^135^ The last method can also yield the corresponding selonlate [Me_4_N]_2_[(MnBr)_4_(Se^i^Pr)_6_] (215) when using MnBr_2_ and [Me_4_N]_2_[Mn_2_(Se^i^Pr^i^)_6_] instead.

In [{Mn(BMAP)}_3_(MnCl)_3_]Cl (216, H_2_BMAP = 2-[bis(2-mercaptoethyl)aminomethyl-pyridine), the BMAP ligands coordinate to three of the Mn centers by their N atoms and also carry two thiols each, which make up the atoms in the E position.^136^ The last Mn atom is saturated by a chlorine atom. It forms when adding H_2_BMAP to MnCl_2_.

##### 2.1.5.7 Group 8/16 adamantane-type clusters

All but one known compounds in this cluster family are iron compounds, which mainly form oxo/hydroxo compounds with polydentate ligands and Fe^III^ centers, but also Fe^II^ thiolate complexes common for most transition metal groups.

A family of oxo/hydroxo clusters comprising heptadentate ligands of the type [{Fe_2_(L)}_2_O_4−*n*_(OH)_*n*_]^*q*^ (in 217–221, *n* = 2, 3, Fig. 8) is obtainable from mostly basic conditions by providing the desired ligand and simple iron salts.^137–141^ The ligands in those systems bridge two Fe atoms by providing an O atom in the E position between them and coordinating *via* three Lewis basic sites to both of them. The charge of the resulting clusters depends on the charge of the ligand and the O/OH ratio. For [Fe_4_(N-Et-HPTB)_2_O_4_][BF_4_]_2_ (222), obtained from bubbling O_2_ through a solution of [Fe_2_(N-Et-HPTB)(dmf)_4_][BF_4_]_3_, all of the four E atoms not part of the organic ligand are oxo ligands.^142^

There is a distinctly different arrangement of bridging ligands found in the hydroxo cluster [{Fe(^*t*^BuOH)}_4_(dppoe)_4_(OH)_6_][PF_6_]_2_Cl_4_ (223, dppoe = 1,2-bis(diphenylphosphine oxide)ethane), in which the neutral ligands are not part of the adamantane architechture.^143^ It was unintentionally found to be the main product in a reaction of [(Cp)(dppe)FeCl] (dppe = 1,2-bis(diphenylphosphino)ethane) with the carborane [*closo*-1,12-C_2_B_11_H_10_(CN)_2_] while in contact to air, oxidizing both the dppe and iron atoms.

Clusters with the non bridging tridentate ligands TACN, [{Fe(TACN)}_4_O_2_(OH)_4_]X_4_ (224–225, X = I, CIO_4_), do also not comprise oxygen atoms from the ligand in their scaffold and were first obtained after the hydrolysis of [(TACN)_2_Fe_2_(acac)_2_(O)](ClO_4_)_2_ under addition of NaX,^144^ although examples of [{Fe(TACN)}_4_O_4−*n*_(OH)_*n*_]^*q*^ (in 226–227, *n* = 2, 3) with different halide counterions could later be synthesized directly from [(TACN)FeCl_3_] with a sodium halide in basic solution.^145,146^

Thiolate clusters of the form [(FeSR)_4_(SR)_6_]^2−^ (in 228–230, Fig. 8) and [(FeX)_4_(SR)_6_]^2−^ (231–232, X = Cl, Br) both exist. The first type is generated by converting FeCl_2_ using thiosulfates^147–149^ and the second by adding the preformed thiol complex [Ph_4_P]_2_[Fe(SPh)_4_] to FeX_2_.^150^ [Et_4_N]_2_[(FeBr)_4_(SBn)_6_](233) can also be prepared by the first method.^151^

[{Fe(BMAP)}_3_(FeCl)_3_]Cl (234) is isostructural to the Mn congener 209 and prepared accordingly.^136^

[(RuPPh_3_)_4_S_6_] (235, Fig. 8) is a singular example, as it is a pure sulfide cluster and the only Ru compound.^152^ It can be formed in reactions of a sulfide source like (SiMe_3_)_2_S or NaSH with PPh_3_ and a Ru^II^ complex like RuCl_2_(DMSO)_4_ resulting in H_2_ or (SiMe_3_)_2_ as reduced side products.

##### 2.1.5.8 Group 9/16 adamantane-type clusters

There are only a few adamantane-type structures comprising cobalt which are known in the literature.

One, [Co_4_(HMPM)_2_](ClO_4_)_2_ (236, H_3_HMPM = 2,6-bis[{{(1-hydroxy-2-methylpropan-2-yl)(pyridine-2-ylmethyl)}amino}methyl]-4-methylphenol, Fig. 8), is formed with two heptadentate ligands, which encompass the six oxygen atoms in the E position and coordinate terminally to the Co^II^ moieties with two N atoms per metal center.^153^ It is formed by combining the deprotonated H_3_HMPM ligand and Co(ClO_4_)_2_.

All other Co adamantane-type clusters are thiolates with a Co^III^ core. Clusters of the form [{Co(SPh)}_4_(SR)_6_]^2−^ (in 237–239) are obtained from the thiolates and common cobalt and ammonium salts.^149,154^ The terminal thiolates can be formally exchanged by halides, as seen in the compound [^*t*^Bu_4_N]_2_[{Co(Cl)}_4_(SPh)_6_] (240) formed from [^*t*^Bu_4_N][CoCl_3_(PPh_3_)] reacting with PhSSiMe_3_ and [Et_4_N]_2_[{Co(Cl)}_4_(SPh)_6_] (241, Fig. 8), which in turn forms in a solution of Na, PhSH, CoCl_2_ and [Et_4_N]Cl.^155,156^

A heterogenous substitution pattern is observed for [{Co(Cl)}_2_(CoPPh_3_)_2_(SPh)_6_] (242) and [{Co(Cl)}_2_(CoPOPh_3_)(CoPPh_3_)(SPh)_6_] (243), which could both be isolated as the products of the addition of PhSSiMe_3_ to the complex [CoCl_2_(PPh_3_)_2_], in the presence of O_2_ in the second case.^155^

**Table 8:** Adamantane-type compounds with group 7–9 elements in the Q-position and group 16 atoms in the E-position^a^

Compound	Reagents/conditions	Method
[{Mn(TACN)}_4_O_6_]Br_3.5_OH_0.5_ (183)	TACN, MnCl_2_, NaBr, O_2_/H_2_O	S^125,126^
[{Mn(TACN)}_4_O_6_](ClO_4_)_4_ (184)	Mn(NO_3_)_2_, Na_2_C_2_O_4_, TACN, NaClO_4_, NaOH, O_2_/MeOH, H_2_O, 60 °C, 3 h or TACN, [Mn_4_O_6_(bpea)_4_](ClO_4_)_4_ (186)/MeCN, RT, 1 h	S^127–129^
[{Mn(TACN)}_4_O_6_](OTf)_4_ (185)	Mn(OTf)_2_, TACN, O_2_/MeCN	S^130^
[{Mn(bpea)}_4_O_6_](ClO_4_)_4_ (186)	Mn(ClO_4_)_2_·6H_2_O, [^*n*^Bu_4_N][MnO_4_],/MeCN, RT, 1 h	C^131^
[{Mn(bpea)}_4_O_6_]Br_4_ (187)	[^*n*^Bu_4_N][Br], [Mn_4_O_6_(bpea)_4_](ClO_4_)_4_ (186)/MeCN, RT, 24 h	O^131^
[{Mn(bpea)}_4_O_6_]X_4_ ((188–192, X = [BF_4_], OTf, [PF_6_], SCN, 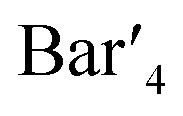 )	[{Mn(bpea)}_4_O_6_)_4_]Br_4_ (187), NaX or KX/H_2_O, RT	O^131^
[{Mn(4,4′-Me_2_bpea)}_4_O_6_](ClO_4_)_4_ (193)	Mn(4,4′-Me_2_bpma)_2_·6H_2_O, [*n*-Bu_4_N][MnO_4_],/MeCN, RT, 1 h	C^131^
[{Mn(5,5′-Me_2_bpea)}_4_O_6_](ClO_4_)_4_ (184)	Mn(5,5′-Me_2_bpma)_2_·6H_2_O, [*n*-Bu_4_N][MnO_4_],/MeCN, RT, 1 h	C^131^
[{Mn(bpea)}_4_O_6_](ClO_4_)_3_ (195)	[Mn_4_O_6_(bpea)_4_](ClO_4_)_4_, [^*n*^Bu_4_N]ClO_4_/MeCN, THF, electrolysis (−0.1 V), 25 min	N^131^
[{Mn(bpea)}_4_O_6_](X)_3_ (196–200, X = [BF_4_], OTf, [PF_6_], SCN, 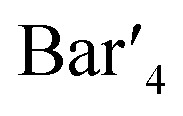 )	TACNMe, [Mn_4_O_6_(bpea)_4_](X)_4_ (188–192)/MeCN, 5 min	S^131^
[{Mn(dien)}_2_{Mn(bpea)}_2_O_6_](ClO_4_)_4_ (201)	[Mn_4_O_6_(bpea)_4_](ClO_4_)_4_ (186), dien/MeCN, RT, 3 h	Q^129^
[{Mn(Medien)}_4_](ClO_4_)_4_ (202)	[Mn_4_O_6_(bpea)_4_](ClO_4_)_4_ (186), medien/MeCN, RT, 45 min	Q^129^
[{Mn(R-ida)}_2_{Mn(bpea)}_2_O_6_] (203–206, R = Me, Bn, ^*t*^Bu, ^*C*^Pe)	[Mn_4_O_6_(bpea)_4_](ClO_4_)_4_ (186), [^*t*^Bu_4_N]_2_[R-ida]/MeCN, RT, 30 min	Q^129^
[{Mn(tame)}_4_O_5_(OH)](OTf)_5_ (207)	tame·3HOTf, Mn(OTf)_2_·MeCN, Et_3_N, O_2_/MeCN, RT, 36 h	S^130^
[{Mn(tame)}_4_O_6_](OTf)_4_ (208)	[{Mn(tame)}_4_O_5_(OH)](OTf)_5_[Mn_4_O_6_(bpea)_4_](ClO_4_)_4_ (207), NEt_3_/MeCN	Q^130^
[{Mn(tame)}_4_O_5_(OH)](OTf)_5_ (209)	[{Mn(TACN)}_4_O_6_](OTf)_4_ (185), HClO_4_/MeCN	P^130^
[Mn_4_O_4_(tphpn)_2_](CF_3_SO_3_)_2_(ClO_4_)_3_ (210)	[{Mn_2_(μ-O)_2_(tphpn)}_2_], [Mn((HB(3,5-Me_2_pz)_3_)_2_](ClO_4_)_2_/MeCN, RT, 10 min	S/K^132^
[Et_4_N]_2_[(MnSPh)_4_(SPh)_6_] (211)	MnCl_2_·4H_2_O, PhSNa, Et_4_NCl·H_2_O/MeOH, RT, 40 min	C^133^
[Me_4_N]_2_[(MnSPh)_4_(SPh)_6_] (212)	MnCl_2_·4H_2_O, PhSNa, Me_4_NCl/MeOH, RT, 40 min	C^133^
[Et_4_N]_2_[(MnBr)_4_(SPh)_6_] (213)	MnBr_2_, NaSPh, [Et_4_N]Br/MeCN, RT, 2 h	C^134^
[Me_4_N]_2_((MnCl)_4_(S^i^Pr)_6_] (214)	[Me_4_N]_2_(Mn_2_(S^i^Pr)_6_], MnCl_2_/MeCN, 35 °C, 5 h	J^135^
[Me_4_N]_2_((MnBr)_4_(Se^i^Pr)_6_] (215)	[Me_4_N]_2_(Mn_2_(Se^i^Pr)_6_], MnBr_2_/MeCN, RT, 12 h	J^135^
[{Mn(BMAP)}_3_(MnCl)_3_]Cl (216)	H_2_BMAP, MnCl_2_/MeOH, 60 °C, 5 min	K^136^
[^*n*^Bu_4_N]_4_[{Fe_2_(HXMeCG)}_2_O_2_(OH)_2_] (217)	FeCl_3_, [^*n*^Bu_4_N]Cl, NaOH, H_5_HMeXCG/H_2_O	K^137^
(HPy)_3_[{Fe_2_(HPhXCG)}_2_O(OH)_3_] (218)	Na_3_H_2_HPhXCG, Py, Fe(NO_3_)_3_/MeOH, RT, 1 month	K^138^
(enH_2_)_1.5_[Fe_4_O(OH)_3_(hpdta)_2_] (219)	H_5_hpdta, Fe(NO_3_)_3_, en, dma/H_2_O, 3 days	K^139^
[{Fe_2_(bpbp)}_2_O_2_(OH)_2_](ClO_4_)_4_ (220)	Hbpbp, Fe(ClO_4_)_3_/THF, H_2_O, RT, 2 days	I^140^
[(Fe_2_{(TACN)CH_2_}_2_CHOH)O(OH)]_2_[PF_6_]_4_ (221)	{(TACN)CH_2_}_2_CHOH, FeCl_3_, NaOAc, NEt_3_, K[PF_6_]/^i^PrOH, 24–36 h	K^141^
[Fe_4_(N-Et-HPTB)_2_O_4_][BF_4_]_2_ (222)	[Fe_2_(N-Et-HPTB)(dmf)_4_][BF_4_]_3_, O_2_/DMF	K^142^
[{Fe(^*t*^BuOH)}_4_(dppoe)_4_(OH)_6_][PF_6_]_2_Cl_4_ (223)	[*closo*-1,12-C_2_B_11_H_10_(CN)_2_], [(Cp)(dppe)FeCl], [NH]_4_[PF_6_], ^*t*^BuOH/THF, 66 °C, 18 h	J^143^
[{Fe(TACN)}_4_O_2_(OH)_4_]X_4_ (224–225, X = I, CIO_4_)	NaX, [(TACN)_2_Fe_2_(acac)_2_(O)](ClO_4_)_2_/Me_2_CO, H_2_O, 2 weeks	I^144^
[{Fe(TACN)}_4_O(OH)_5_](I)_4_I_3_ (226)	[(TACN)FeCl_3_], KI/H_2_O, Py, 72 h	D^145^
[{Fe(TACN)}_4_O_2_(OH)]Br_4_ (227)	[(TACN)FeCl_3_], NaBr/H_2_O, 25 °C, pH = 10.28	D^146^
[R_4_N]_2_[(FeSPh)_4_(SPh)_6_] (228–229, R = Me, Et)	FeCl_2_, NaSPh, [R_4_N]Cl/MeOH	C^147,148^
[Me_4_N]_2_[(FeSEt)_4_(SEt)_6_] (230)	FeCl_2_, NaSEt, [Me_4_N]Br/MeOH, 2 h	C^149^
[Ph_4_P]_2_[(FeCl)_4_(SPh)_6_] (231)	FeCl_2_, [Ph_4_P]_2_[Fe(SPh)_4_]/MeCN, 30 min	C^150^
[Ph_4_P]_2_[(FeBr)_4_(SPh)_6_] (232)	FeBr_2_, [Ph_4_P]_2_[Fe(SPh)_4_]/MeCN, 30 min	C^150^
[Et_4_N]_2_[(FeBr)_4_(SBn)_6_] (233)	FeCl_2_, NaSBn, [Et_4_N]Br/MeCN	C^151^
[{Fe(BMAP)}_3_(FeCl)_3_]Cl (234)	H_2_BMAP, FeCl_2_·4H_2_O/MeOH, 60 °C, 5 min	K^136^
[(RuPPh_3_)_4_S_6_] (235)	RuCl_2_(DMSO)_4_, PPH_3_, (SiMe_3_)_2_S/THF, −50 °C	C^152^
[Co_4_(HMPM)_2_](ClO_4_)_2_ (236)	Co(ClO_4_)_2_, H_3_HMPM, Et_3_N/MeOH, RT	K^153^
[Me_4_N]_2_[{Co(SPh)}_4_(SPh)_6_] (237)	PhSH, Et_3_N, Co(NO_3_)_2_, [Me_4_N]Cl/EtOH	C^154^
[Cy_2_NH_2_]_2_[{Co(SPh)}_4_(SPh)_6_] (238)	PhSH, Cy_2_NH, Co(NO_3_)_2_, [Me_4_N]Cl/EtOH	C^154^
[Et_4_N]_2_[{Co(SEt)}_4_(SEt)_6_] (239)	NaSEt, CoCl_2_, [Et_4_N]Cl/MeCN	C^149^
[^*t*^Bu_4_N]_2_[{Co(Cl)}_4_(SPh)_6_] (240)	[^*t*^Bu_4_N][CoCl_3_(PPh_3_)], PhSSiMe_3_/toluene, 3 h	C^155^
[Et_4_N]_2_[{Co(Cl)}_4_(SPh)_6_] (241)	Na, PhSH, CoCl_2_, [Et_4_N]Cl/MeOH, RT	C^156^
[{Co(Cl)}_2_(CoPPh_3_)_2_(SPh)_6_] (242)	[CoCl_2_(PPh_3_)_2_], PhSSiMe_3_/THF, 3 h	C^155^
[{Co(Cl)}_2_(CoPPh_3_)(CoPOPh_3_)(SPh)_6_] (243)	[CoCl_2_(PPh_3_)_2_], PhSSiMe_3_, O_2_/THF, 3 h	C^155^

a bpea = *N*,*N*-bis(2-pyridylmethyl)ethylamine, 
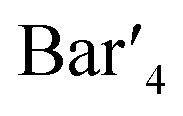
 = [3,5-(CF_3_)_2_C_6_H_3_]_4_B]^−^, dien = diethylenetriamine, medien = *N*′-methyldiethylenetriamine, R-ida = *N*-(R)iminodiacetate, ^*C*^Pe = cyclopentane, tame = *tert*-amyl methyl ether, Htphpn = *N*,*N*,*N*′,*N*′-tetra-(2-methylpyridyl)-2-hydroxypropanediamine, pz = pyrazolyl, H_2_BMAP = 2-[bis(2-mercaptoethyl)aminomethyl]pyridine, Py = pyridine, H_5_HMeXCG = *N*,*N*′-(2-hydroxy-5-methyl-1,3-xylylene)bis(*N*-(carboxymethyl)glycine), H_5_HPhXCG = *N*,*N*′-(2-hydroxy-5-phenyl-1,3-xylylene)bis(*N*-(carboxymethyl)glycine), dma = *N*,*N*-dimethylacetamid, Hbpbp = 2,6-bis((*N*,*N*′-bis-(2-picolyl)amino)methyl)-4-*tert*-butylphenol, {(TACN)CH_2_}_2_CHOH = 1,3-bis(1,4,7-triaza-1-cyclononyl)-2-hydroxypropane, N-Et-HPTB = *N*,*N*,*N*′,*N*′-tetrakis(2-(1-ethylbenzimidazolyl))-2-hydroxy-1,3-diaminopropane, dppoe = 1,2-bis(diphenylphosphine oxide)ethane, dppe = 1,2-bis(diphenylphosphino)ethane, H_3_HMPM = 2,6-bis[{{(1-hydroxy-2-methylpropan-2-yl)(pyridine-2-ylmethyl)}amino}methyl]-4-methylphenol.

##### 2.1.5.9 Group 11/16 adamantane-type clusters

The second largest family of compounds with group 16 elements in the E position is the 11/16 combination. Most of them exist for the elemental combination Cu and S, although some Ag examples and clusters with different chalcogenides are known.

Thiolate containing adamantane-type cluster anions of the general composition [Cu_4_(SR)_6_]^2−^ (in 244–255) have been extensively studied, and can be obtained by reacting a copper salt with the desired thiolate or by using a monomeric precursor complex already containing the SR species in most cases.^157–169^ In some cases, this involves a reduction of the copper atoms from Cu^II^ to Cu^I^.

Different synthetic approaches have also been showcased. An interesting alternative synthesis route features the inversion of Q and E positions during the transformation of the S/Cu adamantane-type structure [(NEt_4_]_4_[(SPh)_4_(CuBr)_6_] (847, see section 2.1.7) to the desired [Et_4_N]_2_[Cu_4_(SPh)_6_] (256) by addition of [Et_4_N]SPh in DMF.^170^

**Fig. 9:**
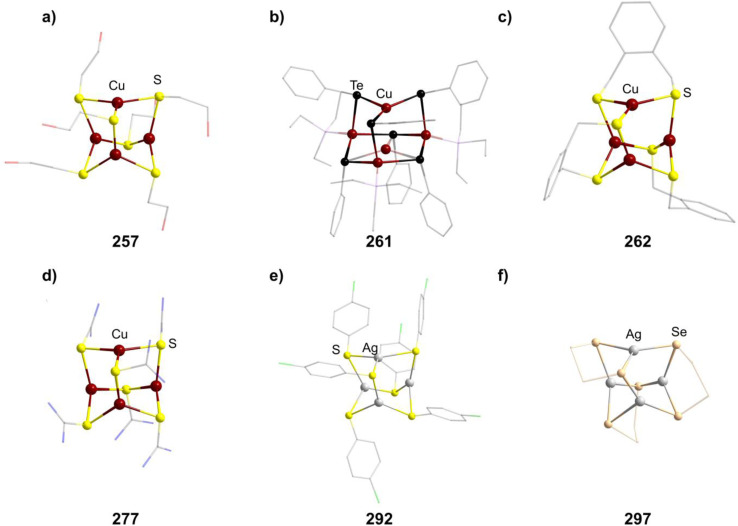
Examples of adamantane-type compounds with group 11 in the Q-position and group 16 atoms in the E-position: [(^*n*^Bu)_4_N]_2_[Cu_4_(SCH_2_CH_2_OH)_6_] (257, top left (a)), [Et_3_PPh][μ_3_-Cu(CuPEt_3_)_3_Cu(TePh)_6_] (261, top center (b)), [Ph_4_P]_2_[Cu_4_{*o*-(SCH_2_)_2_C_6_H_4_}_3_] (262, top right (c)), [Cu_4_{SC(NH_2_)_2_}_6_](SO_4_)_2_ (277, bottom left (d)), [Et_4_N]_2_[Ag_4_(SC_6_H_4_-*p*-Cl)_6_] (292, bottom center (e)) and [^*n*^Pr_4_N]_2_[Ag_4_(Se_4_)_3_] (297, bottom right (f)). Hydrogen atoms and counterions, if present, are omitted for clarity.

The polymer (CuSCH_2_CH_2_OH)_*n*_ decomposes and dissolves in basic aqueous solutions to give the adamantane-type [(^*n*^Bu)_4_N]_2_[Cu_4_(SCH_2_CH_2_OH)_6_] (257, Fig. 9).^171^

An electrochemical synthesis route to the thiolate cluster [Cu(BIK)_2_]_2_[Cu_4_{S(*o*-tolyl)}_6_] (258, BIK = bis(2-methyl-imidazole-2-yl)ketone) is also feasible using a Cu anode in an electrolyte solution of BIK, the thiol HS(*o*-tolyl) and [^*n*^Bu_4_N]ClO_4_ in MeCN.^172^

Analogous reactions can also generate the selenium congener [Me_4_N]_2_[Cu_4_(SePh)_6_] (259),^173^ while the only known Te congener [^*t*^Bu_3_PH]_2_[Cu_4_(TePh)_6_] (260) has been obtained from a rearrangement of the cluster [(^*t*^Bu_3_P)_3_(CuTePh)_4_].^174^

There is however another tellurium containing adamantane-type structure formally derived from this example. Unlike many other adamantanes discussed here containing a μ_4_-atom in the center, this one features a μ_3_-Cu atom. One six membered (CuPEt_3_)_3_Te_3_ ring of the adamantane-type scaffold in [Et_3_PPh][μ_3_-Cu(CuPEt_3_)_3_Cu(TePh)_6_] (261, Fig. 9) coordinates an additional Cu atom in its center opposite to a naked Cu atom in the Q position, leading to a more planar arrangement of the six membered ring.^175^ Isolation was possible if Te(Ph)SiMe_3_ was used as a tellurolate source in a solution with CuCl and PEt_3_.

A related family of adamantane-type ions [Cu_4_(SRS)_3_]^2−^ (in 262–274, Fig. 9) comprises bridging bis-thiolates in the E position. This leads to two different copper sites: three copper moieties are coordinated by two sulfur atoms of the same bis-thiolate and one from another, while the last Cu atom is coordinated by three different ligands.

Their synthesis normally follows the same patterns as has been discussed for the monothiolates,^176–181^ although two examples can be found that form by rearrangement of other copper thiolate compounds.^179,182,183^

A purely inorganic S_4_ bridge in place of a bis-thiolate could also be observed in the compound [Ph_4_P]_2_[Cu_4_(S_4_)_3_] (275), prepared from a reaction of elemental sulfur, H_2_S and Cu(OAc)_2_, thus involving a reduction of the copper atoms.^184^

Utilizing neutral thiones in place of thiolates results in the formation of cationic adamantanes of the type [Cu_4_(SCR_2_)_6_]^4+^ (in 276–280, Fig. 9).^185–189^ This is achieved by addition of the thione to simple copper salts, mostly nitrates or sulfates, in common solvents. Depending on the concentrations and additives used, additional thione ligands can also coordinate to one or multiple Cu sites in the cluster, expanding their coordination number from three to four (281–283).^185,190,191^ When choosing CuI as a precursor, such an addition of iodide is observed on all copper atoms, resulting in neutral clusters [(CuI)_4_(SR_2_)_6_] (284–285).^192,193^ A Cl homolog [(CuCl)_4_{SC(NH_2_)NHCH_2_CH=CH_2_}_6_] (286) is observed in an electrochemical reaction at copper electrodes in an electrolyte of CuCl_2_, HCl and SC(NH_2_)NHCH_2_CH=CH_2_ in ethanol.^194^

Using linked phosphine sulfides or selenides (EPPh_2_)_2_N^−^ (E = S, Se) results in cluster cations [Cu_4_{(EPPh_2_)_2_N}_3_]^+^ (in 287–290) with the same architecture as described for linked thiolates.^195–198^

[Cu_4_(O_3_N_4_)_2_](ClO_4_)_2_ (291, H_3_O_3_N_4_ = 1-Me-4-OH-3,4-bis(CH_2_N(CH_2_C_5_H_4_N)(CMe_2_CH_2_OH)–C_6_H_2_) represents the only example of a Cu^II^ as well as a Cu/O cluster compound. The two ligands deliver three oxygen atoms in the E position and additionally coordinate to two copper atoms each *via* four N moieties, resulting in a heptadentate coordination.^199^ Despite the differences, the reaction pathway is similar to the thiolate route as the ligand is deprotonated before reaction with a simple copper salt.

While fewer examples for silver exist, they can generally be seen as the simple heavier congeners of known Cu compounds. [Et_4_N]_2_[Ag_4_(SC_6_H_4_-*p*-Cl)_6_] (292, Fig. 9) results from transferring the chemistry of simple Cu thiolates to silver,^166^ while [Ph_4_P]_2_[Ag_4_{*o*-(SCH_2_)_2_C_6_H_4_}_3_] (293) and [^*n*^Bu_4_N]_2_[Ag_4_(FcSe_2_)_3_] (294, Fc = ferrocenyl) can be isolated when using a bis-thiolate or bis-selenide respectively.^200,201^

Another silver thiolate could be found as the anion in an intercluster compound [Et_4_N][Br@Ag_8_(2-TBI)_12_(SO_4_)_2_][Ag_4_(2-TBI)_6_(SO_4_)_3_]_2_ (295, 2-TBI = 2-thiobenzimidadzol) together with an octomeric cluster, in which it is additionally coordinated by three sulfate ions. While the reactants are similar to those used in other reactions leading to thiolate adamantanes, solvothermal conditions and ultrasonic activation are used in this case.^202^

The only Te homolog in this compound family is found in [Ph_4_P]_2_[Ag_4_(C_4_H_3_STe)_6_] (296). The ligand of this cluster is made by addition of elemental Te to thiophene in the presence of ^*n*^BuLi.^203^

An oligoselenide-containing cluster [^*n*^Pr_4_N]_2_[Ag_4_(Se_4_)_3_] (297, Fig. 9) in analogy to the sulfide congener could also be obtained after using Na_2_Se_5_ as the selenide source.^204^

The nitrogen bridged phophine selenide [Ag_4_{(SePPh_2_)_2_N}_3_](OTf) (298) is another example of a silver compound that can be prepared according to the synthesis used for its copper homolog.^205^

Lastly, a second selenone [(AgPPh_3_)_4_(Mbis)_3_](OTf)_4_ (299, Mbis = 1,1′-methylenebis(3-methylimidazoline-2-selone)) unique to the chemistry with silver results from the addition of Mbis to [Ag(OTf)(PPh_3_)], which leads to an adamantane featuring PPh_3_ terminal ligands at the silver positions.^206^

**Table 9:** Adamantane-type compounds with group 11 in the Q-position and group 16 atoms in the E-position^a^

Compound	Reagents/conditions	Method
[Me_4_N]_2_[Cu_4_(SPh)_6_] (244)	Cu(NO_3_), PhSH, ^*n*^Bu_3_N, [Me_4_N]Cl/EtOH, 75 °C	C^157–159^
[Ph_4_P]_2_[Cu_4_(SPh)_6_] (245)	[Ph_4_P]_2_[Cu(SPh)_3_], [Cu(MeCN)_4_]ClO_4_/MeCN, 82 °C, 5 min	J^160,161^
[Li(diglyme)_2_]_2_[Cu_4_(SPh)_6_] (246)	CuN(SiMe_3_)_2_, LiN(SiMe_3_)_2_, HSPh/diglyme, 110 °C, 10 min	C^162^
[Li(dme)_3_]_2_[Cu_4_(SPh)_6_] (247)	CuN(SiMe_3_)_2_, LiN(SiMe_3_)_2_, HSPh/DME, 84 °C, 10 min	C^162^
[Li(15-crown-5)thf]_2_[Cu_4_(SPh)_6_] (248)	CuN(SiMe_3_)_2_, LiN(SiMe_3_)_2_, HSPh, 15-crown-5/THF, slight heat, 5 min	C^162^
[Me_4_N]_2_[Cu_4_(SMe)_6_] (249)	[Me_4_N][CuCl_2_], NaSMe/EtOH, MeCN, 75 °C, 90 min	C^159,163^
[^*n*^Pr_4_N]_2_[Cu_4_(SMe)_6_] (250)	Cu_2_O, [^*n*^Pr_4_N]Br, NaOMe/(CH_2_OH)_2_, MeOH, MeCN, 55 °C, 1 h	C^164^
[Ph_4_P]_2_[Cu_4_(SEt)_6_] (251)	Cu_2_O, EtSH, [Ph_4_P]Br, NaOMe/(CH_2_OH)_2_, 55 °C	C^165^
[Et_4_N]_2_[Cu_4_(SC_6_H_4_-*p*-Cl)_6_] (252)	Cu(NO_3_)_2_, HSC_6_H_4_-*p*-Cl, ^*n*^Bu_3_N, [Et_4_N]Cl/EtOH, MeOH, MeCN, 50 °C to 4 °C, 18 h	C^166^
[Et_4_N]_2_[Cu_4_{S(*o*-^*t*^BuC_6_H_4_)}_6_] (253)	CuCl, HS(*o*-^*t*^BuC_6_H_4_, NaH, [Et_4_N]Cl/DMF	C^167^
[Et_4_N]_2_[Cu_4_(S^i^Pr)_6_] (254)	CuCl, HS^i^Pr, NaH, [Et_4_N]Cl/THF, 24 h	C^168^
[K(Me_2_phen)_3_]_2_[Cu_4_(SBn)_6_] (255)	CuCl, KSBn, Me_2_phen/THF	C^169^
[Et_4_N]_2_[Cu_4_(SPh)_6_] (256)	[(NEt_4_]_4_[(SPh)_4_(CuBr)_6_] (847), HSPh, Et_3_N/DMF, 15 min	J^170^
[(^*n*^Bu)_4_N]_2_[Cu_4_(SCH_2_CH_2_OH)_6_] (257)	(CuSCH_2_CH_2_OH)_*n*_, [(^*n*^Bu)_4_N]OH/H_2_O	I^171^
[Cu(BIK)_2_]_2_[Cu_4_{S(*o*-tolyl)}_6_] (258)	BIK, HS(*o*-tolyl), Cu anode, [^*n*^Bu_4_N]ClO_4_/MeCN, electrolysis	N^172^
[Me_4_N]_2_[Cu_4_(SePh)_6_] (259)	CuCl, PhSeH, Et_3_N, [Me_4_N]Cl/DMF, MeOH	C^173^
[^*t*^Bu_3_PH]_2_[Cu_4_(TePh)_6_] (260)	[(^*t*^Bu_3_P)_3_(CuTePh)_4_], Me_3_SiTePh, Me_3_GaOEt_2_/THF	J^174^
[Et_3_PPh][μ_3_-Cu(CuPEt_3_)_3_Cu(TePh)_6_] (261)	PEt_3_, CuCl, Te(Ph)SiMe_3_/Pentane, RT, 18 h	C^175^
[Ph_4_P]_2_[Cu_4_{*o*-(SCH_2_)_2_C_6_H_4_}_3_] (262)	Cu(NO_3_)_2_, *o*-(HSCH_2_)_2_C_6_H_4_, NEt_3_, [Ph_4_P]Br/EtOH, 5 h	C^176^
[Ph_4_P]_2_[Cu_4_(SCH_2_CH_2_S)_3_] (263)	CuCl, HSCH_2_CH_2_SH, NEt_3_, [Ph_4_P]Br/MeCN, 5 h	C^177,178^
[(Me_3_P)_4_Cu]_2_[Cu_4_(SCH_2_CH_2_S)_3_] (264)	[CuSCH_2_CH_2_SCu], PMe_3_/PhMe, 90 °C, 1.5 h	C^179^
[Ph_4_P]_2_[Cu_4_{S(CH_2_)_3_S}_3_] (265)	HS(CH_2_)_3_SH, Cu_2_O, [Ph_4_P]Br, NaOMe/(CH_2_OH)_2_, MeOH, 55 °C, 1 h	C^178^
[Me_4_N]_2_[Cu_4_{S(CH_2_)_3_S}_3_] (266)	HS(CH_2_)_3_SH, Cu_2_O, [Me_4_N]Cl, NaOMe/MeCN, MeOH, 50 °C, 1 h	C^178^
[Et_4_N]_2_[Cu_4_{S(CH_2_)_3_S}_3_] (267)	HS(CH_2_)_3_SH, Cu_2_O, [Et_4_N]Br, NaOMe/MeCN, MeOH, 50 °C, 45 min	C^178^
[Et_4_N]_2_[Cu_4_(SCH_2_CH_2_S)_3_] (268)	HSCH_2_CH_2_SH, Cu_2_O, [Et_4_N]OH/MeCN, MeOH, 50 °C	C^178^
[Me_3_NCH_2_Ph]_2_[Cu_4_(SCH_2_CH_2_S)_3_] (269)	HSCH_2_CH_2_SH, Cu_2_O, [Me_3_NCH_2_Ph]Cl, NaOMe/glycerol, MeOH, 45 °C	C^178^
[Me_4_N]_2_[Cu_4_(C_8_H_6_S_8_)_3_] (270)	[Cu(MeCN)_4_][PF_6_], C_8_H_8_S_8_, [Me_4_N]OH/THF, Me_2_CO, MeOH, 3 days	C^180^
[Ph_4_P]_2_[Cu_4_(tpdt)_3_] (271)	CuCl_2_, 5,6-thieno[2,3-*d*]-1,3-dithiol-2-one, KOMe, [Ph_4_P]Br/MeOH, 1 h	C^181^
[Ph_4_P]_2_[Cu_4_(α-tpdt)_3_] (272)	CuCl_2_, thieno[3,4-*d*]-1,3-dithiol-2-thione, KOMe, [Ph_4_P]Br/MeOH, 1 h	C^181^
[(Me_3_P)_4_Cu][Cu_4_(SCH_2_CH_2_S)_3_(CuPPh_3_)] (273)	[(Me_3_P)_4_Cu]_2_[Cu_4_(SCH_2_CH_2_S)_3_]/THF	J^179^
K[Ph_4_P][Cu_4_(^*t*^Bu_2_DED)_3_] (274)	K_4_[Cu_8_(^*t*^Bu_2_DED)_6_], [Ph_4_P]Cl, S/Me_2_CO, EtOH	J^182,183^
[Ph_4_P]_2_[Cu_4_(S_4_)_3_] (275)	S, H_2_S, Cu(MeCO_2_)_2_, [Ph_4_P]Br, NH_3_/MeCN	C^184^
[Cu_4_{SC(NH_2_)_2_}_6_](NO_3_)_4_ (276)	CuNO_3_, SC(NH_2_)_2_ HNO_3_/H_2_O	C^185^
[Cu_4_{SC(NH_2_)_2_}_6_](SO_4_)_2_ (277)	CuSO_4_, SC(NH_2_)_2_, HOAc/H_2_O, 80°	C^186^
[Cu_4_{SC(NH_2_)_2_}_6_](HSO_4_)_2_SO_4_ (278)	CuSO_4_, SC(NH_2_)_2_, H_2_SO_4_/H_2_O, 80 °C	C^186,187^
[Cu_4_(H_4_pymtH)_6_](ClO_4_)_4_ (279)	[Cu(C_2_H_4_)ClO_4_], H_4_pymtH, C_2_H_4_/MeOH	C^188^
[Cu_4_{SC(NH_2_)NHCH_2_CH=CH_2_}_6_](OTf)_4_ (280)	Cu(OTf)_2_, SC(NH_2_)NHCH_2_CH=CH_2_/C_6_H_6_, 20 min	C^189^
[{CuSC(NH_2_)_2_}_3_Cu{SC(NH_2_)_2_}_6_](NO_3_)_4_ (281)	CuNO_3_, SC(NH_2_)_2,_ HNO_3_/H_2_O	C^185^
[{CuSC(NH_2_)_2_}Cu_3_{SC(NH_2_)_2_}_6_](SO_4_)_2_ (282)	CuSO_4_, SC(NH_2_)_2_, H_2_SO_4_/H_2_O	C^190^
[{CuSC(NH_2_)_2_}(CuNO_3_)Cu_2_{SC(NH_2_)_2_}_6_](SO_4_)(NO_3_) (283)	Cu(NO_3_)_2_, SC(NH_2_)_2_/H_2_O, 80 °C to 5 °C, 5 days	C^191^
[(CuI)_4_{SC(NH_2_)NHEt}_6_] (284)	CuI, SC(NH_2_)NHEt/EtOH, 50 °C, 3 h	C^192^
[(CuI)_4_{SC(NH_2_)_2_}_6_] (285)	CuI, SC(NH_2_)_2_, KI/H_2_O, 80 °C	C^193^
[(CuCl)_4_{SC(NH_2_)NHCH_2_CH=CH_2_}_6_] (286)	CuCl_2_, Cu electrode, SC(NH_2_)NHCH_2_CH=CH_2_, HCl/EtOH, 0.2 V, 0.13 mA	N^194^
[Cu_4_{(SPPh_2_)_2_N}_3_][Cu^I^Cl_2_] (287)	1. NaN(SPPh_2_)_2_, CuCl_2_/H_2_O	J^195,196^
	2. CCl_4_, CH_2_Cl_2_	
[Cu_4_{(SPPh_2_)_2_N}_3_][BF_4_] (288)	[Cu(MeCN)_4_][BF_4_], (SPPh_2_)_2_NH/CH_2_Cl_2_, 1 h	C^197^
[Cu_4_{(SPPh_2_)_2_N}_3_]I_3_ (289)	Cu, (SPPh_2_)_2_NH·I_2_/Et_2_O, 2 days	C^198^
[Cu_4_{(SePPh_2_)_2_N}_3_][BF_4_] (290)	[Cu(MeCN)_4_][BF_4_], (SePPh_2_)_2_NH/CH_2_Cl_2_, 1 h	C^197^
[Cu_4_(O_3_N_4_)_2_](ClO_4_)_2_ (291)	Cu(ClO_4_)_2_, H_3_O_3_N_4_, Et_3_N/MeOH	C^199^
[Et_4_N]_2_[Ag_4_(SC_6_H_4_-*p*-Cl)_6_] (292)	AgNO_3_, HSC_6_H_4_-*p*-Cl, ^*n*^Bu_3_N, [Et_4_N]Cl/EtOH, MeOH, MeCN, 50 °C to 4 °C, 18 h	C^166^
[Ph_4_P]_2_[Ag_4_{*o*-(SCH_2_)_2_C_6_H_4_}_3_] (293)	AgNO_3_, Na_2_o-(SCH_2_)_2_C_6_H_4_, [Ph_4_P]Br/MeOH, 5 h	C^200^
[^*n*^Bu_4_N]_2_[Ag_4_(FcSe_2_)_3_] (294)	AgCl, Fc(SeSiMe_3_)_2_, [^*n*^Bu_4_N]Br/THF	C^201^
[Et_4_N][Br@Ag_8_(2-TBI)_12_(SO_4_)_2_][Ag_4_(2-TBI)_6_(SO_4_)_3_]_2_ (295)	Ag_2_SO_4_, 2-TBI, [Et_4_N]Br/MeCN, DMF, sonification, 120 °C, 2 days	B/L^202^
[Ph_4_P]_2_[Ag_4_(C_4_H_3_STe)_6_] (296)	1. Te, [Ph_4_P]Br, thiophene, ^*n*^BuLi/THF	C^203^
	2. AgNO_3_/DMF	
[^*n*^Pr_4_N]_2_[Ag_4_(Se_4_)_3_] (297)	AgNO_3_, Na_2_Se_5_, [^*n*^Pr_4_N]Cl/DMF	C^204^
[Ag_4_{(SePPh_2_)_2_N}_3_](OTf) (298)	Ag(OTf), K{(SePPh_2_)_2_N}/CH_2_Cl_2_, 30 min	C^205^
[(AgPPh_3_)_4_(Mbis)_3_](OTf)_4_ (299)	[Ag(OTf)(PPh_3_)], Mbis/Me_2_CO, 1 h	C^206^

a Me_2_phen = 2,9-dimethyl-1,10-phenanthroline, BIK = bis(2-methyl-imidazole-2-yl)ketone, ^*t*^Bu_2_DED = 1,1-dicarbo-*tert*-butoxy-2,2-ethylenedithiolate, tpdt = 3,4-thiophenedithiolate, α-tpdt = 2,3-thiophenedithiolate, H_4_pymtH = 3,4,5,6-tetrahydropyrimidine-2-thione, H_3_O_3_N_4_ = 1-Me-4-OH-3,4-bis(CH_2_N(CH_2_C_5_H_4_N)(CMe_2_CH_2_OH)–C_6_H_2_, Fc = ferrocenyl, 2-TBI = 2-thiobenzimidadzol, Mbis = 1,1′-methylenebis(3-methylimidazoline-2-selone).

##### 2.1.5.10 Group 12/16 adamantane-type clusters

**Fig. 10:**
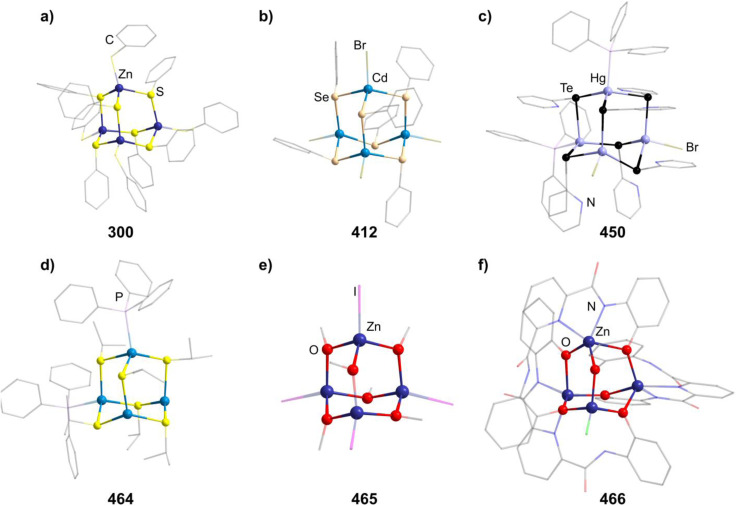
Examples of adamantane-type compounds with group 12 in the Q-position and group 16 atoms in the E-position: [Et_3_NH]_2_[(ZnSPh)_4_(SPh)_6_] (300, top left (a)), [Me_4_N]_2_[(CdBr)_4_(SPh)_6_] (412, top center (b)), [(HgPPh_3_)_2_(HgBr)_2_(Te^*o*^Py)_6_] (450, top right (c)), [Cd_2_(CdPPh_3_)_2_(S^i^Pr)_6_][ClO_4_]_2_ (464, bottom left (d)), [2.2.2]-cryptH_2_[(ZnI)_4_(MeO)_6_] (465, bottom center (e)) and [Zn_4_(POPYH)_3_Cl] (466, bottom right (f)). Hydrogen atoms and counterions, if present, are omitted for clarity.

This family of compounds has been studied systematically in regards to the influence of different ligands, elemental combinations and counter ions. Most of the studies on Zn compounds could be transferred to their cadmium and, unusually for period 6 elements, also to their Hg homologs. While the number of compounds investigated is very high, the types of compounds are not as diverse as for other combinations. With the exception of two clusters, all of them feature chalcogenolate groups in the E position. In the simplest case, this leads to anions of the type [(MER)_4_(ER)_6_]^2−^ (M = Zn, Cd; E = S, Se, 300–319, Fig. 10).

While the first such compounds were obtained from electrolysis of metal anodes in basic thiol solutions,^207,208^ a simpler method involving reactions between chalcogenolate solutions and simple non-halide metal salts at mostly room temperature has subsequently been used.^148,209–222^

In solution, Cd clusters can exchange chalcogenolates, including partial substitution with tellurium, to form mixed compounds [Cd_4_(ER)_*n*_(E′R′)_10−*n*_]^2−^ (in 320–368) by reacting them with 
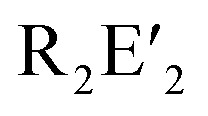
, or in equilibrium reactions with other similar clusters.^222^ The latter strategy also works to form the mixed metal compound [Me_4_N][Cd_*n*_Zn_4−*n*_(SPh)_10_] (369–371).

By utilizing a zwitter-ionic thiolate 4-(trimethylammonio)benzenethiolate (Tab), the cationic adamantane in [(MTab)_4_(Tab)_6_][PF_6_]_8_ (372–373, M = Zn, Cd) can be isolated by the above described method.^223^

The terminal chalcogenolates can be formally replaced by halides (374–433, Fig. 10). This can be done by ligand exchange reactions with PhICl_2_, Br_2_ or I_2_,^214,224^ or during cluster formation by using halide salts, which can also be used to stabilize Hg clusters including rare examples of Hg_4_Te_6_ scaffolds.^225–230^

As described for the pure chalcogenolate clusters, mixed metal adamantanes [Et_4_N]_2_[(MI)_4_(M′I)_4−*n*_(S^*n*^Pr)_6_] (434–442, M = Zn, Cd, Hg, Fig. 10) can be obtained by exchange reactions between homometallic congeners.

Asymmetric substitution at the terminal position is possible as well. Depending on the ratio and chalcogenide used, anions of the type [(MX)_*n*_(SR)_4−*n*_(SR)_6_]^2−^ (443–447, M = Zn, Cd; X = Cl, Br) can be isolated.^210,231,232^ Trying to obtain the HgI/SePh compound with a [(Ph_3_P)_2_N]^+^ countercation resulted in a charge reduced anion [Hg(HgI)_3_(SePh)_6_]^−^ (in 448) with one Hg site not carrying any ligand.^225^

To reduce the negative charge of the cluster compounds, replacement of the terminal anionic ligands used previously with neutral lewis basic ligands like phosphines or arsines was necessary. With mercury, the neutral compounds [(HgPPh_3_)_2_(HgX)_2_(Te^*o*^Py)_6_] (449–451, X = Cl, Br, I; ^*o*^Py = *ortho*-pyridyl) and [(HgPPh_3_)_2_(HgSePh)_2_(SePh)_6_] (452) with mixed terminal ligands were obtainable when using halide or acetate mercury salts.^233,234^ A complex precursor [M(L)_2_(ClO_4_)_2_] (M = Cd, Hg; L = PPh_3_, PEt_3_, AsPh_3_) in combination with M(ER)_2_ (E = S, Se) and free L leads to cationic clusters in [(ML)_4_(ER)_6_][ClO_4_]_2_ (453–464, Fig. 10).^235–237^ With certain L and R combinations, this can lead to clusters with a few terminally uncoordinated M sites, which do not, however, influence the charge.^235^

There are only two examples with oxygen in the E position. One, the methanolate cluster [2.2.2]-cryptH_2_[(ZnI)_4_(MeO)_6_] (465, [2.2.2]-crypt = 4,7,13,16,21,24-hexaoxa-1,10-diazabicyclo[8.8.8]hexacosane, Fig. 10) is obtained in a simple reaction of ZnI_2_ and [2.2.2]-crypt in MeOH in which the cryptand acts as a base.^238^

The other example, [Zn_4_(POPYH)_3_Cl] (466, POPYH_4_ = *N*,*N*′-bis(2-hydroxyphenyl)-pyridine-2,6-dicarboxamide, Fig. 10) is formed by the partially deprotonated multidentate ligand coordinating to ZnCl_2_, and comprises two different Zn sites.^239^ Three are coordinated by two oxygen and two nitrogen atoms of one ligand and one oxygen of another, while the last connects to three different ligands *via* their oxygen atoms and carries an additional terminal Cl ligand.

**Table 10:** Adamantane-type compounds with group 12 in the Q-position and group 16 atoms in the E-position^a^

Compound	Reagents/conditions	Method
[Et_3_NH]_2_[(ZnSPh)_4_(SPh)_6_] (300)	HSPh, Zn anode, Et_3_N, [Et_4_N]ClO_4_/MeCN, electrolysis or NaSPh, ZnCl_2_ [Et_3_NH]Cl/MeOH, 0 °C, 90 min	N,C^207–209^
[Me_4_N]_2_[(ZnSPh)_4_(SPh)_6_] (301)	HSPh, Zn(NO_3_)_2_, Et_3_N, [^*n*^Pr_4_N]Cl/MeOH, Me_2_CO, 3 days	C^210^
(DAMS)_2_[(ZnSPh)_4_(SPh)_6_] (302)	HSPh, Zn(NO_3_)_2_, Et_3_N, (DAMS)I/MeOH	C^211^
[Ru(2,2′-bipy)_3_][(ZnSPh)_4_(SPh)_6_] (303)	Cd(SPh)_2_, SC(NH_2_)_2_, [Ru(2,2′-bipy)_3_Cl_2_]/MeCN, H_2_O, 85 °C, 10 days	B^212^
[Et_4_N]_2_[(ZnSBn)_4_(SBn)_6_] (304)	BnSH, NaOMe, Zn(NO_3_)_2,_ [Et_4_N]_2_Cl/MeOH, 2 h	C^213^
[Ph_4_P]_2_[(ZnSBn)_4_(SBn)_6_] (305)	BnSH, NaOMe, Zn(NO_3_)_2,_ [Ph_4_P]_2_Cl/MeOH, 2 h	C^213^
[Et_3_NH][Me_4_N][(ZnSC_6_H_4_-4-Cl)_4_(SC_6_H_4_-4-Cl)_6_] (306)	HSC_6_H_4_-4-Cl, Et_3_N, Zn(NO_3_)_2_ [Me_4_N]Cl/MeOH, 0 °C, 30 min	C^209^
[Me_4_N]_2_[(ZnSC_6_H_4_-4-Cl)_4_(SC_6_H_4_-4-Cl)_6_] (307)	HSC_6_H_4_-4-Cl, NaOH, ZnCl_2_ [Me_4_N]Cl/MeOH, 0 °C, 2 h	C^209^
[Et_3_NH][(ZnSC_6_H_4_-4-Cl)_4_(SC_6_H_4_-4-Cl)_6_] (308)	HSC_6_H_4_-4-Cl, Et_3_N, Zn(NO_3_)_2_ [Et_3_NH]Cl/MeOH, 0 °C, 30 min	C^209^
[Me_4_N]_2_[(ZnSePh)_4_(SePh)_6_] (309)	Na, HSePh, Zn(NO_3_)_2_, Et_3_N, [^*n*^Pr_4_N]Cl/H_2_O, MeOH, MeCN, 60 °C	C^214,215^
[Et_3_NH][(CdSPh)_4_(SPh)_6_] (310)	HSPh, Cd anode, Et_3_N, [Et_4_N]ClO_4_/MeCN, electrolysis	N^208^
[Me_4_N]_2_[(CdSPh)_4_(SPh)_6_] (311)	HSPh, Cd(NO_3_)_2_, Et_3_N, [Me_4_N]Cl/MeOH	C^216^
[Et_4_N]_2_[(CdSPh)_4_(SPh)_6_] (312)	HSPh, CdCl_2_, Et_3_N/MeOH, H_2_O	C^148,217^
(DAMS)_2_[(CdSPh)_4_(SPh)_6_] (313)	(DAMS)I, PhSH, Et_3_N, Cd(SCN)_2_,/MeOH, 10 min	C^218^
[M(phen)_3_][(CdSPh)_4_(SPh)_6_] (314–316, M = Ru, Fe, Ni)	[Me_4_N][(CdSPh)_4_(SPh)_6_] (311), M(phen)_3_Cl_2_/MeCN, 30 min	O^219^
[Et_4_N]_2_[(CdSCy)_4_(SCy)_6_] (317)	NaSCy, CdCl_2_, [Et_3_N]Cl/EtOH, MeCN	C^220^
[Et_3_NH]_2_[(CdSC_6_H_4_-4-Me)_4_(SC_6_H_4_-4-Me)_6_] (318)	Cd[ClO_4_]_2_, SC_6_H_4_-4-Me, Et_3_N, [Me_4_N]Cl/MeOH, 1 h	C^221^
[Me_4_N][(CdSePh)_4_(SePh)_6_] (319)	NaSePh, Cd(NO_3_)_2_, [Me_4_N]Cl/MeOH, H_2_O, MeCN, 80 °C	C^215,222^
[Me_4_N][Cd_4_(SPh)_10−*n*_(SMe)_*n*_] (320–322, *n* = 8–10)	[Me_4_N][(CdSPh)_4_(SPh)_6_] (311), Me_2_S_2_/Me_2_CO	Q^222^
[Me_4_N][Cd_4_(SPh)_10−*n*_(S^*n*^Bu)_*n*_] (323–329, *n* = 4–10)	[Me_4_N][(CdSPh)_4_(SPh)_6_] (311), ^*n*^Bu_2_S_2_/Me_2_CO	Q^222^
[Me_4_N][Cd_4_(SPh)_10−*n*_(SBn)_*n*_] (330–333, *n* = 7–10)	[Me_4_N][(CdSPh)_4_(SPh)_6_] (311), Bn_2_S_2_/Me_2_CO	Q^222^
[Me_4_N][Cd_4_(SPh)_10−*n*_{S(2-C_6_H_4_Me)}_*n*_] (334–344, *n* = 0–10)	[Me_4_N][(CdSPh)_4_(SPh)_6_] (311), (2-C_6_H_4_Me)_2_S_2_/Me_2_CO	Q^222^
[Me_4_N][Cd_4_(SePh)_10−*n*_(S^*n*^Bu)_*n*_] (345–350, *n* = 5–10)	[Me_4_N][(CdSePh)_4_(SPh)_6_] (319), ^*n*^Bu_2_S_2_/Me_2_CO	R^222^
[Me_4_N][Cd_4_(SPh)_10−*n*_(TePh)_*n*_] (351–353, *n* = 8–10)	[Me_4_N][(CdSPh)_4_(SPh)_6_] (311), Ph_2_Te_2_/Me_2_CO	R^222^
[Me_4_N][Cd_4_(SePh)_10−*n*_(TePh)_*n*_] (354–357, *n* = 7–10)	[Me_4_N][(CdSePh)_4_(SePh)_6_] (319), Ph_2_Te_2_/Me_2_CO	R^222^
[Me_4_N][Cd_4_(SPh)_10−*n*_(SePh)_*n*_] (358–368, *n* = 0–10)	[Me_4_N][(CdSPh)_4_(SPh)_6_] (311), [Me_4_N][(CdSePh)_4_(SePh)_6_]/	R^222^
[Me_4_N][Cd_*x*_Zn_4−*n*_(SPh)_10_] (369–371, *n* = 2–4)	[Me_4_N][(CdSPh)_4_(SPh)_6_] (311), [Me_4_N]_2_[(ZnSPh)_4_(SPh)_6_]/Me_2_CO	R^222^
[(MTab)_4_(Tab)_6_][PF_6_]_8_ (372–373, M = Zn, Cd)	TabH[PF_6_], M(OAc)_2_/MeCN, DMF, MeOH, 70 °C, 1 h	C^223^
[Me_4_N]_2_[(ZnCl)_4_(SPh)_6_] (374)	[Me_4_N]_2_[(ZnSPh)_4_(SPh)_6_] (301), PhICl_2_/MeCN, 10 min	Q^214,224^
[Me_4_N]_2_[(ZnBr)_4_(SPh)_6_] (375)	[Me_4_N]_2_[(ZnSPh)_4_(SPh)_6_] (301), Br_2_/CCl_4_, Me_2_CO, 10 min	Q^214,224^
[Me_4_N]_2_[(ZnI)_4_(SPh)_6_] (376)	[Me_4_N]_2_[(ZnSPh)_4_(SPh)_6_] (301), I_2_/Me_2_CO	Q^214,224^
[^*n*^Bu_4_N]_2_[(ZnI)_4_(S^*n*^Pr)_6_] (377)	Zn(S^*n*^Pr)_2_, ZnI_2_, [^*n*^Bu_4_N]I/CH_2_Cl_2_	C^225^
[Et_4_N]_2_[(MX)_4_(SR)_6_] (378–406, R/M/X = ^i^Pr/Zn/Cl, Br, I; ^i^Pr/Cd/Cl, Br, I; Me/Zn/Br, I; ^*n*^Pr/Zn/I; ^*n*^Bu/Zn/I; ^*n*^Bu/Cd/I, Et/Zn/Cl, Br, I; Et/Cd/Cl, Br, I, Bn/Zn/Cl, Br, I; Bn/Cd/Cl, Br, I, ^*sec*^Bu/Zn/Cl, Br, I; ^*sec*^Bu/Cd/Cl, Br, I)	MX_2_, [Et_4_N]X, M(SR)_2_/CH_2_Cl_2_, 1 h	C^226^
[Me_4_N]_2_[(ZnCl)_4_(SePh)_6_] (407)	[Me_4_N]_2_[(ZnSePh)_4_(SePh)_6_] (309), PhICl_2_/MeCN, 10 min	Q^214^
[Me_4_N]_2_[(CdCl)_4_(SPh)_6_] (408)	[Me_4_N]_2_[(CdSPh)_4_(SPh)_6_] (309), PhICl_2_/MeCN, 10 min	Q^214^
[R_4_N]_2_[(CdCl)_4_(SePh)_6_] (409–410, R = ^*n*^Pr, ^*n*^Bu)	CdCl_2_, (cat)Cl, Cd(SPh)_2_/CH_2_Cl_2_, 1 h	C^227,228^
[^*n*^Pr_3_PH]_2_[(CdCl)_4_(SeFc)_6_] (411)	^*n*^Pr_3_P, CdCl_2_, Me_3_SiSeFc/THF, 10 min	C^229^
[Me_4_N]_2_[(CdBr)_4_(SPh)_6_] (412)	[Me_4_N]_2_[(CdSPh)_4_(SPh)_6_] (311), Br_2_/CCl_4_, Me_2_CO, 10 min	Q^214^
[Me_4_N]_2_[(CdI)_4_(SPh)_6_] (413)	[Me_4_N]_2_[(CdSPh)_4_(SPh)_6_] (311), I_2_/Me_2_CO	Q^214^
(DAMS)_2_[(CdI)_4_(SPh)_6_] (414)	(DAMS)I, PhSH, Et_3_N, Cd(NO_3_)_2_,/MeOH, 10 min	C^218^
[Et_4_N]_2_[(CdI)_4_(S^*n*^Pr)_6_] (415)	CdI_2_, [Et_4_N]I, Cd(S^*n*^Pr)_2_/CH_2_Cl_2_	C^225^
[Me_4_N]_2_[(CdCl)_4_(SePh)_6_] (416)	[Me_4_N]_2_[(CdSePh)_4_(SePh)_6_] (319), PhICl_2_/MeCN, 10 min	Q^214^
[Me_4_N]_2_[(CdBr)_4_(SePh)_6_] (417)	[Me_4_N]_2_[(CdSePh)_4_(SePh)_6_] (319), Br_2_/CCl_4_, Me_2_CO, 10 min	Q^214^
[Me_4_N]_2_[(CdI)_4_(SePh)_6_] (418)	[Me_4_N]_2_[(CdSePh)_4_(SePh)_6_] (319), I_2_/Me_2_CO	Q^214^
[Et_4_N]_2_[(HgX)_4_(S^*n*^Pr)_6_] (419–421, X = Cl, Br, I)	HgX_2_, [Et_4_N]X, Hg(S^*n*^Pr)_2_/CH_2_Cl_2_	C^225^
[Et_4_N]_2_[(HgX)_4_(SePh)_6_] (422–424, X = Cl, Br, I)	HgX_2_, [Et_4_N]X, Hg(SePh)_2_/CH_2_Cl_2_	C^225^
[Mg(CH_2_{P(O)Ph_2_}_2_)_3_][(HgX)_4_(SePh)_6_] (425–427, X = Cl, Br, I)	Hg(SePh)_2_, MgX_2_, CH_2_{P(O)Ph_2_}_2_/DMF, 1 h	C^230^
[M(CH_2_{P(O)Ph_2_}_2_)_3_][(HgBr)_4_(SePh)_6_] (428–430, M = Fe, Co, Ni)	Hg(SePh)_2_, MBr_2_, CH_2_{P(O)Ph_2_}_2_/DMF, 1 h	C^230^
[Et_4_N]_2_[(HgX)_4_(TePh)_6_] (431–433, X = Cl, Br, I)	HgX_2_, [Et_4_N]X, Hg(TePh)_2_/CH_2_Cl_2_, 30 min	C^225^
[Et_4_N]_2_[(CdI)_*n*_(ZnI)_4−*n*_(S^*n*^Pr)_6_] (434–436, *n* = 1–3)	[Et_4_N]_2_[(CdI)_4_(S^*n*^Pr)_6_] (415), [^*n*^Bu_4_N]_2_[(ZnI)_4_(S^*n*^Pr)_6_] (377)/CH_2_Cl_2_	R^225^
[Et_4_N]_2_[(HgI)_*n*_(CdI)_4−*n*_(S^*n*^Pr)_6_] (437–439, *n* = 1–3)	[Et_4_N]_2_[(HgI)_4_(S^*n*^Pr)_6_] (421), [Et_4_N]_2_[(CdI)_4_(S^*n*^Pr)_6_] (415)/CH_2_Cl_2_	R^225^
[Et_4_N]_2_[(HgI)_*n*_(ZnI)_4−*n*_(S^*n*^Pr)_6_] (440–442, *n* = 1–3)	[Et_4_N]_2_[(HgI)_4_(S^*n*^Pr)_6_] (421), [^*n*^Bu_4_N]_2_[(ZnI)_4_(S^*n*^Pr)_6_] (377)/CH_2_Cl_2_	R^225^
[Me_4_N]_2_[(ZnSPh)_2_(ZnX)_2_(SPh)_6_] (443–444, X = Cl, Br)	HSPh, Zn(NO_3_)_2_, Et_3_N, [Me_4_N]X/MeOH, Me_2_CO, 10 days	C^210^
[Me_4_N]_2_[(ZnSPh)_3_(ZnCl)(SPh)_6_] (445)	HSPh, Zn(NO_3_)_2_, Et_3_N, [^*n*^Pr_4_N]Cl/MeOH, Me_2_CO, 10 days	C^210^
[Et_3_NH]_2_[(CdCl)_3_(*p*-^*t*^Bu-C_6_H_4_SCd)(*p*-^*t*^Bu-C_6_H_4_S)_6_] (446)	*p*-^*t*^Bu-C_6_H_4_SH, Et_3_N, CdCl_2_/MeOH, 19.5 h	C^231^
[Me_4_N]_2_[(CdSPh)_3_(CdCl)(SPh)_6_] (447)	HSPh, Cd(NO_3_)_2_, NEt_3_, [Me_4_N]Cl/MeOH, 1 h	C^232^
[(Ph_3_P)_2_N]_2_[Hg(HgI)_3_(SePh)_6_] (448)	HgI_2_, [(Ph_3_P)_2_N]I, Hg(SePh)_2_/CH_2_Cl_2_	C^225^
[(HgPPh_3_)_2_(HgX)_2_(Te^*o*^Py)_6_] (449–451, X = Cl, Br, I)	^*o*^Py_2_Te_2_, Li[BH_4_], Hg(OAc)_2_, HgX_2_, PPh_3_/DMF, EtOH, THF, 2 h	C^233^
[(HgPPh_3_)_2_(HgSePh)_2_(SePh)_6_] (452)	HgBr_2_, PPh_3_, HSePh, Et_3_N/MeCN, 3 days	C^234^
[(CdPPh_3_)_4_(SPh)_6_][ClO_4_]_2_ (453)	[Cd(PPh_3_)_2_(ClO_4_)_2_], Cd(SPh)_2_, PPh_3_/CH_2_Cl_2_, 20 min	C^235^
[(CdPPh_3_)_4_(SePh)_6_][ClO_4_]_2_ (454)	[Cd(PPh_3_)_2_(ClO_4_)_2_], Cd(SePh)_2_, PPh_3_/CH_2_Cl_2_, 20 min	C^235^
[(HgPPh_3_)_4_(EPh)_6_][ClO_4_]_2_ (455–456, E = S, Se)	[Hg(PPh_3_)_2_(ClO_4_)_2_], Hg(EPh)_2_, PPh_3_/CHCl_3_, 10 min	C^236,237^
[(HgPPh_3_)_4_(SMe)_6_][ClO_4_]_2_ (457)	[Hg(PPh_3_)_2_(ClO_4_)_2_], Hg(SMe)_2_, PPh_3_/CHCl_3_, 10 min	C^236^
[(HgPPh_3_)_4_(SEt)_6_][ClO_4_]_2_ (458)	[Hg(PPh_3_)_2_(ClO_4_)_2_], Hg(SEt)_2_, PPh_3_/CH_2_Cl_2_, 10 min	C^236^
[(HgAsPh_3_)_4_(SPh)_6_][ClO_4_]_2_ (459)	[Hg(AsPh_3_)_2_(ClO_4_)_2_], Hg(SPh)_2_, AsPh_3_/CHCl_3_, 10 min	C^236^
[(HgPEt_3_)_4_(SPh)_6_][ClO_4_]_2_ (460)	[Hg(PEt_3_)_2_(ClO_4_)_2_], Hg(SPh)_2_, PEt_3_/Me_2_CO	C^236^
[(HgPEt_3_)_4_(SePh)_6_][ClO_4_]_2_ (461)	[Hg(PEt_3_)_2_(ClO_4_)_2_], Hg(SePh)_2_, PEt_3_/CHCl_3_, 10 min	C^236^
[Cd(CdPPh_3_)_3_(S^*n*^Pr)_6_][ClO_4_]_2_ (462)	[Cd(PPh_3_)_2_(ClO_4_)_2_], Cd(S^*n*^Pr)_2_, PPh_3_/CH_2_Cl_2_, 20 min	C^235^
[Cd_2_(CdPPh_3_)_2_(SR)_6_][ClO_4_]_2_ (463–464, R = Cy, ^i^Pr)	[Cd(PPh_3_)_2_(ClO_4_)_2_], Cd(SR)_2_, PPh_3_/CH_2_Cl_2_, 20 min	C^235^
[2.2.2]-cryptH_2_[(ZnI)_4_(MeO)_6_] (465)	[2.2.2]-crypt, ZnI_2_/MeOH, 1 day	C^238^
[Zn_4_(POPYH)_3_Cl] (466)	POPYH_4_, Et_3_N, ZnCl_2_/MeCN, 70 °C, 3 h	K^239^

a DAMS = *trans*-4-(4-dimethylamino-styryl)-*N*-methyl-pyridinium, bipy = bipyridine, ^*n*^Pr = normal propyl, ^sec^Bu = secondary butyl, phen = 1,10-phenanthroline, ^*o*^Py = *ortho*-pyridyl, Tab = 4-(trimethylammonio)benzenethiolate, [2.2.2]-crypt = 4,7,13,16,21,24-Hexaoxa-1,10-diazabicyclo[8.8.8]hexacosane, POPYH_4_ = *N*,*N*′-bis(2-hydroxyphenyl)-pyridine-2,6-dicarboxamide.

##### 2.1.5.11 Group 13/16 adamantane-type clusters

**Fig. 11:**
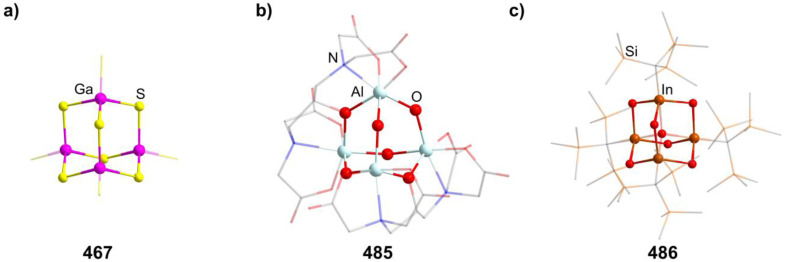
Examples of adamantane-type compounds with group 13 elements in the Q-position and group 16 atoms in the E-position: K_8_[Ga_4_S_10_] (467, left (a)), [enH_2_][Al_4_(OH)_4_(hpdta)_2_] (485, center (b)) and [(μ_4_-O){(Me_3_Si)_3_CIn}_4_(OH)_6_] (486, right (c)). Hydrogen atoms and counterions, if present, are omitted for clarity.

Some group 13 examples with Al, Ga and In are known, although no examples with Te have been observed so far. The simplest examples of group 13/16 adamantane-type structures are [Q_4_E_10_]^8−^ (467–469, Fig. 11) anionic clusters, which were the first to be realized for Ga/S, In/S and In/Se from the binary Q_2_E_3_ and K_2_E in water.^240^ The only other example of such clusters is [H_2_dap]_4_[Ga_4_Se_10_] (470, dap = 1,2-diaminopropane), also synthesized in aqueous solution, but directly from the elements and dap in solvothermal conditions.^241^

Derivatization of the cluster archetype by protonation of the terminal sulfur atoms was presented for two compounds [(InSH)_4_S_6_]^4−^ (in 471–472) with ammonium counterions, prepared by Method B.^242,243^ The addition of a larger fragment was reached in {[Ni(tepa)]_2_SO_4_}[Ni(tepa)(GaSH)_4_S_6_] (473, tepa = tetraethylenepentamine), which additionally comprises of a Ni complex coordinated by the cluster, obtained solvothermally from NiS, Ga and tepa.^241^

Formally substituting the terminal chalcogenides by neutral amine ligands yields neutral compounds [(QNR_3_)_4_S_6_]. A condensation of Me_3_N·AlH_3_ and (SiMe_3_)_2_S forms the Al congener [(AlNMe_3_)_4_S_6_] (474),^244^ while a Ga cluster [(GaNH_3_)_4_S_6_] (475) is isolated after the solvethermal reaction of Ga, S and [NMe_4_]Cl in hydrazine hydrate.^243^ A compound with a slightly larger ligand [(4-Me_2_N–C_5_H_4_NGa)_4_S_6_] (476) could be achieved in a two step synthesis *via* an intermediate [(4-Me_2_N–C_5_H_4_N)GaSH_0.64_Cl_0.36_] formed by (SiMe_3_)_2_S and the ligand decorated GaHCl_2_ species, which can then be converted to the target compound by an additional ligand.^245^

Another way to achieve neutral clusters is the partial functionalization of the chalcogenides in the E position observed in [(MI)_4_(SMe)_4_S_2_] (477–478, M = Al, Ga), prepared by solid state reactions from binary or elemental compounds.^246,247^

Hydroxo clusters of indium [(TACNIn)_4_(OH)_6_]^6+^ (479–480) were the first oxygen species reported, synthesized at room temperature by InCl_3_ and TACN in basic aqueous solution in the presence of different counterions.^248^

By utilizing a formally negative ligand, the charge reduced dication [(BuGa)_4_(OH)_6_][CHB_11_Br_6_Me_5_] (481) with a carborane counterion was obtained from hydrolysis of a low coordinate Ga complex.^249^

Another cluster type counterion is observed in [{(Me_3_Si)_3_Si}_4_Ga_4_O(OH)_5_][{(CO)_3_Fe}_3_{GaSi(SiMe_3_)_3_}_2_{GaFe(CO)_4_}] (482), which comprises a central Ga_4_O(OH)_5_ adamantane-type structure with mixed E sites decorated by hypersilyl groups (Si(SiMe_3_)_3_) leading to a monocationic cluster.^250^ It is formed by a rearrangement of [(Me_3_Si)_3_SiGaCl]_4_ in the presence of Na_2_Fe(CO)_4_·2 dioxane and NaOH.

Mixed oxo and hydroxo clusters [{(Me_3_Si)_3_CM}_4_O_2_(OH)_4_] (483–484, M = Al, Ga) can also be isolated as neutral compounds from the stepwise hydrolysis of a precursor complex [(Me_3_Si)_3_CMMe_2_], albeit in low yields.^251^

A pentadentate ligand was used to create a dianionic compound, [enH_2_][Al_4_(OH)_4_(hpdta)_2_] (485, en = ethane-1,2-diamine, Fig. 11), in which the hpdta ligands each use one oxygen moiety as a μ-bridging site in the E position while coordinating with the two N atoms and the other four oxygen positions to the Al atoms.^139^ The cluster was isolated after a simple condensation reaction between AlCl_3_ and the quintuply protonated ligand H_5_hpdta in ethane-1,2-diamine.

Lastly, a single oxo centered cluster [(μ_4_-O){(Me_3_Si)_3_CIn}_4_(OH)_6_] (486, Fig. 11) is synthesized by reacting the In complex Li[Me_3_SiInCl_3_] with Li[AlH_4_] to obtain a cyclic Li/In hydride compound [(Me_3_Si)(H)In(μ-H)Li(thf)_2_(μ-H)In(μ-H)(H)(SiMe_3_)], which will subsequently hydrolyze to the target compound.^252^

**Table 11:** Adamantane-type compounds with group 13 elements in the Q-position and group 16 atoms in the E-position^a^

Compound	Reagents/conditions	Method
K_8_[Ga_4_S_10_] (467)	Ga_2_S_3_, K_2_S/H_2_O, 90 °C, 4 h	C^240^
K_8_[In_4_S_10_] (468)	In_2_S_3_, K_2_S/H_2_O, 90 °C, 4 h	C^240^
K_8_[In_4_Se_10_] (469)	In_2_Se_3_, K_2_S/H_2_O, 90 °C, 4 h	C^240^
[H_2_dap]_4_[Ga_4_Se_10_] (470)	Ga, Se, dap, H_2_O/170 °C, 5 days	B^241^
[(C_3_H_7_)_2_NH_2_]_4_[(InSH)_4_S_6_] (471)	In, S, dipropylamine/180 °C, 5 days	B^242^
[NHMe_3_]_4_[(InSH)_4_S_6_] (472)	In, S, NMe_3_/EtOH, 140 °C, 5 days	B^243^
{[Ni(tepa)]_2_SO_4_}[Ni(tepa)(GaSH)_4_S_6_] (473)	Ga, NiS, tepa/H_2_O, 180 °C, 7 days	B^241^
[(AlNMe_3_)_4_S_6_] (474)	Me_3_N·AlH_3_, (SiMe_3_)_2_S/toluene, 110 °C, 5 days	D^244^
[(GaNH_3_)_4_S_6_] (475)	Ga, S, [NMe_4_]Cl, urea/N_2_H_4_·H_2_O, 180 °C, 8 days	B^243^
[(4-Me_2_N–C_5_H_4_NGa)_4_S_6_] (476)	1. (4-Me_2_N–C_5_H_4_N)GaHCl_2_, (SiMe_3_)_2_S/MeCN, −25 °C to RT, 29 h	D^245^
	2. 4-Me_2_N–C_5_H_4_N/MeCN, 82 °C, 8 h	
[(AlI)_4_(SMe)_4_S_2_] (477)	1. Ga, GaI_3_, AlI_3_/200 °C	A^247^
	2. Me_2_S_2_/110 °C	
[(GaI)_4_(SMe)_4_S_2_] (478)	Me_2_S_2_, Ga_2_I_4_/110 °C	A^246^
[(TACNIn)_4_(OH)_6_](ClO_4_)_6_ (479)	InBr_3_, NaOH, NaClO_4_, TACN/H_2_O, 12 h	C^248^
[(TACNIn)_4_(OH)_6_](S_2_O_6_)_3_ (480)	InBr_3_, NaOH, NaS_2_O_6_, TACN/H_2_O, 12 h	C^248^
[(BuGa)_4_(OH)_6_][CHB_11_Br_6_Me_5_] (481)	[2,6-(2,6-Mes_2_C_6_H_3_)_2_C_5_H_3_Ga^*n*^Bu][CHB_11_Br_6_Me_5_], H_2_O/C_6_D_6_, 16 h	I^249^
[{(Me_3_Si)_3_Si}_4_Ga_4_O(OH)_5_][{(CO)_3_Fe}_3_{GaSi(SiMe_3_)_3_}_2_{GaFe(CO)_4_}] (482)	[(Me_3_Si)_3_SiGaCl]_4_, Na_2_Fe(CO)_4_·2 dioxane, NaOH/Et_2_O	J^250^
[{(Me_3_Si)_3_CAl}_4_O_2_(OH)_4_] (483)	1. AlMe_2_Cl, [(Me_3_Si)_3_CLi·2 thf]/THF, hexane, 15 h	I^251^
	2. H_2_O/THF, −10 °C, 1 h	
[{(Me_3_Si)_3_CGa}_4_O_2_(OH)_4_] (484)	1. GaMe_2_Cl, [(Me_3_Si)_3_CLi·2 thf]/THF, hexane, 15 h	I^251^
	2. H_2_O/THF, 24 h, 150 °C, 4 h	
[enH_2_][Al_4_(OH)_4_(hpdta)_2_] (485)	H_5_hpdta, AlCl_3_, en/H_2_O	K^139^
[(μ_4_-O){(Me_3_Si)_3_CIn}_4_(OH)_6_] (486)	1. InCl_3_, (Me_3_Si)_3_CLi/THF, −40 °C	I^252^
	2. LiAlH_4_/THF, −78 °C	
	3. MeOH, H_2_O	

a dap = 1,2-diaminopropane, tepa = tetraethylenepentamine

##### 2.1.5.12 Group 14/16 adamantane-type clusters

The combination of group 14 and 16 elements entails the most compounds investigated until now. Most examples have been synthesized with the sulfides, selenides and, to a lesser degree, tellurides. Looking at the group 14 element, there are many examples for compounds with Si, Ge and Sn, but only a single one for a compound with Pb.

**Fig. 12:**
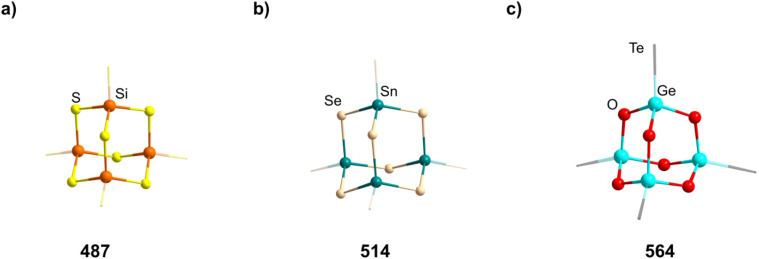
Examples of adamantane-type compounds with purely inorganic cluster anions with group 14 elements in the Q position and group 16 atoms in the E position: Na_4_[Si_4_S_10_] (487, left (a)), [18-crown-6-K]_4_[Sn_4_Se_10_] (514, center (b)) and [Mn(en)_3_]_2_[Ge_4_O_6_Te_4_] (564, right (c)). Counterions are omitted for clarity.

Two large groups of monomeric compounds can be defined: the first are purely inorganic cluster anions with a formal composition of [Q_4_E_10_]^4−^ (487–563, Fig. 12) and their derivatives. They are the analogs to previously discussed group 13 compounds like [Ga_4_S_10_]^8−^ but feature many more examples and a lower charge. They are mostly formed from the elements and/or simple binary precursors by the Methods A–C and E, resulting in regular adamantane-type anions with mostly (alkaline) metal or ammonium counterions.^253–282^ In a unique synthetic approach, it was also shown that those clusters can be made electrochemically using a Sn_2_Se_3_ cathode in a [Et_4_N]Br electrolyte solution in ethane-1,2-diamine to form [Et_4_N]_4_[Sn_4_Se_10_] (517).^283^

The known [Ge_4_E_10_] cluster compounds are often used as reactants in ion exchange reactions (Method L) to introduce a desired functionality or structural template to the compound, such as larger ammonium cations forming lamellar structures (521–538),^284–286^ organic molecules (539–546)^287–291^ or transition metal complexes with interesting optical properties(547–551).^289,292,293^ The family of clusters with metal complex counterions could also be expanded by starting from elements and binary precursors in solvothermal reactions (Method B) to not only obtain more Ge clusters (552–556),^294–297^ but also Sn congeners as well as rare earth containing examples(557–562).^298–300^ In one case, the addition of antimony to such a reaction mix of GeO_2_ and elemental sulfur led to the formation of a compound with two distinct clusters, [(Me)_2_NH_2_]_6_[Ge_2_Sb_2_S_7_][Ge_4_S_10_] (563), one adamantane-type and another ternary molecule with noradamantane like topology.^301^

Unlike the other compounds in this section, the oxo cluster compound [Mn(en)_3_]_2_[Ge_4_O_6_Te_4_] (564, Fig. 12) deviates from the strict [Q_4_E_10_]^4−^ cluster buildup and carries terminal Te groups at the Q position. It is obtained from a solvothermal reaction of Ge, Te, Mn(OAc)_2_ and [Me_4_N]I in ethane-1,2-diamine.^302^

**Table 12:** Adamantane-type compounds with purely inorganic cluster anions with group 14 elements in the Q position and group 16 atoms in the E position^a^

Compound	Reagents/conditions	Method
Na_4_[Si_4_S_10_] (487)	SiS_2_, Na_2_S/800 °C, 48 h	A^253^
Na_4_[Si_4_Se_10_] (488)	Na, Si, Se/800 °C	A^254^
K_4_[Si_4_Te_10_] (489)	K, Si, Te/350 to 400 °C, 17 h	A^255^
Na_4_[Ge_4_S_10_] (490)	GeS_2_, Na_2_S/H_2_O or GeS_2_, Na_2_S/800 °C, 48 h	A/C^253,256–259^
K_4_[Ge_4_S_10_] (491)	GeS_2_, K_2_S/H_2_O	C^256^
Rb_4_[Ge_4_S_10_] (492)	GeS_2_, Rb_2_S/H_2_O	C^256^
Cs_4_[Ge_4_S_10_] (493)	GeS_2_, Cs_2_S/H_2_O or S, Ge, CsOH/H_2_O, 150 °C, 16 h	B/C ^256,260,261^
Ba_2_[Ge_4_S_10_] (494)	GeS_2_, BaS/1250 °C	A^253^
Tl_4_[Ge_4_S_10_] (495)	Tl_2_S, GeS_2_/500 °C, 10 days	A^262^
[Me_4_N]_4_[Ge_4_S_10_] (496)	GeS_2_, [Me_4_N]HS, H_2_S/H_2_O, 150 °C, 4 days or GeS_2_, [Me_4_N]Cl, Na_2_CO_3_/H_2_O, 120 °C, 2 days	G/B^263–266^
[EtNH_3_]_3_[MeNH_3_][Ge_4_S_10_] (497)	1. GeO_2_, S, MeNH_2_/EtOH, 160 °C, 24 h	B^281^
	2. EtNH_2_/EtOH, 160 °C, 24 h	
[Li_4_(H_2_O)_16_][Ge_4_Se_10_] (498)	1. LiSe_2_, Ge, Se/heat to melt	E^282^
	2. H_2_O	
[Li_4_(thf)_12_][Ge_4_Se_10_] (499)	1. LiSe_2_, Ge, Se/heat to melt	E^282^
	2. THF	
Na_4_[Ge_4_Se_10_] (500)	Na, Ge, Se/800 °C	A^267^
K_4_[Ge_4_Se_10_] (501)	K, Ge, Se/800 °C	A^268^
Rb_4_[Ge_4_Se_10_] (502)	Rb_2_CO_3_, Ge, Se/MeOH, 190 °C, 24 h	B^269^
Cs_4_[Ge_4_Se_10_] (503)	Cs_2_CO_3_, Ge, Se/MeOH, 190 °C to RT, 4 h	B^270^
Tl_4_[Ge_4_Se_10_] (504)	Tl_2_Se, GeSe_2_/500 to 400 °C, 9 days	A^271^
[Me_4_N]_4_[Ge_4_Se_10_] (505)	Ge, Se, Me_4_N]OH/H_2_O, 150 °C, 3 days	B^272^
[(C_3_H_7_)_3_NH]_4_[Ge_4_Se_10_] (506)	Ge, Se, N(C_3_H_7_)_3_/H_2_O, 230 °C, 20 days	B^273^
[Et_4_N]_4_[Ge_4_Te_10_] (507)	1. K_2_Te, Ge, Te/heat to melt	E^274^
	2. [Et_4_N]Br/en, 3 days	
[R_4_N]_4_[Sn_4_E_10_], (508–513, R = Me, Et; E = S, Se, Te)	1. K_2_E, E, Sn/heat to melt	E^275^
	2. [R_4_N]Br/en, 100 °C, 12 h	
[18-Crown-6-K]_4_[Sn_4_Se_10_] (514)	1. K, Sn, Se/heat to melt	E^276^
	2. 18-Crown-6/THF, en, 14 days	
(K[2.2.2]-crypt)_4_[Sn_4_Se_10_] (515)	1. K, Sn, Se/heat to melt	E^277^
	2. [2.2.2]-crypt/en, NH_3_, -40 °C	
[Me_4_N]_4_[Sn_4_Se_10_] (516)	Sn, Se, [Me_4_N]OH,/H_2_O, 150 °C, 16 days	B^278^
[Et_4_N]_4_[Sn_4_Se_10_] (517)	[Et_4_N]Br, Sn_2_Se_3_ cathode, Ni anode/en, 300 μA, 5 V, 5 days	N^283^
[(CHMeEt)_2_NH_2_]_4_[Sn_4_Se_10_] (518)	Sn, Se (CHMeEt)_2_NH/H_2_O, 160 °C, 25 days	B^279^
[(C_3_H_7_)_2_NH_2_]_4_[Sn_4_Se_10_] (519)	Sn, Se, S, (C_3_H_7_)_3_N/H_2_O, 130 °C, 20 days	B^280^
[18-Crown-6-K]_4_[Sn_4_Te_10_] (520)	1. K_2_Te, Sn, Te/heat to melt	E^276^
	2. 18-Crown-6/THF, en, 28 days	
[C_*n*_H_2*n*+1_NMe_3_]_4_[Ge_4_S_10_] (521–524, *n* = 12, 14, 16, 18)	Na_4_[Ge_4_S_10_] (490), [C_*n*_H_2*n*+1_NMe_3_]Br/H_2_O, 18 h	O^284^
[C_8_H_17_NMe_3_]_4_[Ge_4_Se_10_] (525)	K_4_[Ge_4_Se_10_] (501), [C_8_H_17_NMe_3_]Br/Me_2_CO, H_2_O, 3 days	O^285^
[C_9_H_19_NMe_3_]_4_[Ge_4_Se_10_] (526)	K_4_[Ge_4_Se_10_] (501), [C_9_H_19_NMe_3_]Br/Me_2_CO, H_2_O, 45 °C, 1 day	O^285^
[C_8_H_17_NMe_2_H]_4_[Ge_4_Se_10_] (527)	K_4_[Ge_4_Se_10_] (501), [C_8_H_17_NMe_2_H]Cl/Me_2_CO, H_2_O, 40 °C, 1 day	O^285^
[C_*n*_H_2*n*+1_NMe_3_]_4_[Ge_4_Se_10_] (528–530, *n* = 10, 11, 12)	K_4_[Ge_4_Se_10_] (501), [C_*n*_H_2*n*+1_NMe_3_]Br/Me_2_CO, H_2_O, 80 °C, 1 day	O^285^
[C_*n*_H_2*n*+1_NMe_3_]_4_[Ge_4_Se_10_] (531–573, *n* = 14, 16, 18)	K_4_[Ge_4_Se_10_] (501), [C_*n*_H_2*n*+1_NMe_3_]Br/Me_2_CO, H_2_O, 120 °C, 3 days	O^285^
[(C_4_H_9_)_3_NH]_4_[Ge_4_Se_10_] (534)	K_4_[Ge_4_Se_10_] (501), (C_4_H_9_)_3_N, HCl/Me_2_CO, H_2_O, 50 °C, 3 days	O^285^
[C_*n*_H_2*n*+1_NH_3_]_4_[Ge_4_Se_10_] (535–538, *n* = 12, 14, 16, 18)	Na_4_[Ge_4_Se_10_] (500), [C_*n*_H_*n*+1_NH_3_]Cl/EtOH, H_2_O, 60 °C 2 h	O^286^
(H_2_4,4′-bipy)_2_[Ge_4_S_10_]·4,4′-bipy (539)	[Me_4_N]_4_[Ge_4_S_10_] (496), Cu(NO_3_)_2_, 4,4′-bipy/140 °C, 3 days	O^287^
[(C_*n*_H_2*n*+1_)_2_Vio]_2_[Ge_4_S_10_] (540–543, *n* = 0, 2, 3,4)	[Me_4_N]_2_[Ge_4_S_10_] (496), [(C_*n*_H_2*n*+1_)_2_Vio]/^i^PrOH, H_2_O, 3 days	O^288^
[Me_2_Vio]_2_[Ge_4_S_10_] (544)	[Me_4_N]_2_[Ge_4_S_10_] (496), [MV]I_2_/H_2_O, MeOH, DMF	O^289^
TMPyP[Ge_4_S_10_] (545)	[Me_4_N]_2_[Ge_4_S_10_] (496), TMPyP(PF_6_)_4_/MeOH, H_2_O, DMF, 80 °C, 7 days	O^290^
[DMBPE]_2_[Ge_4_S_10_] (546)	[Me_4_N]_2_[Ge_4_S_10_] (496), [DMBPE]I_2_/H_2_O	O^291^
[Ni(cyclam)]_3_[Ni(cyclam)(H_2_O)_2_][Ge_4_S_10_]_2_ (547)	[Me_4_N]_2_[Ge_4_S_10_] (496), [Ni(cyclam)](ClO_4_)_2_/MeCN, H_2_O, 3 days	O^292^
[Mn(2,2′-bipy)_2_H_2_O][Ge_4_S_10_] (548)	[Me_4_N]_2_[Ge_4_S_10_] (496), [Mn(2,2′bipy)_3_](ClO_4_)_2_/MeCN, H_2_O, 3 days	O^292^
[Fe(2,2′-bipy)_3_]_2_[Ge_4_S_10_] (549)	[Me_4_N]_2_[Ge_4_S_10_] (496), [Fe(2,2′bipy)_3_](ClO_4_)_2_/H_2_O, 1 day	O^292^
[Ni(phen)_3_]_2_[Ge_4_S_10_] (550)	[Me_4_N]_2_[Ge_4_S_10_] (496), [Ni(phen)_3_]Cl_2_/MeOH, H_2_O, 12 h	O^293^
MnTMPyP[Ge_4_S_10_] (551)	[Me_4_N]_2_[Ge_4_S_10_] (496), TMPyP(PF_6_)_4_, MnCl_2_/MeOH, H_2_O, DMF, 80 °C, 7 days	O^290^
[Ni(trien)_2_]_2_[Ge_4_S_10_] (552)	GeO_2_, NiCl_2_, S/trien, 160 °C, 5 days	B^294^
[M(dap)_3_]_4_[Ge_4_S_10_]Cl_4_ (553–554, M = Co, Ni))	GeO_2_, Sb, S, MCl_2_/dap, 170 °C, 6 days	B^295^
[Ni_2_(μ-teta)(teta)_2_][Ge_4_S_10_] (555)	GeO_2_, S, NiCl_2_, teta/H_2_O, 170 °C, 12 days	B^296,297^
[Ni(teta)_2_]_2_[Ge_4_Se_10_] (556)	GeO_2_, Se, NiCl_2_, teta/H_2_O, 170 °C, 16 days	B^296^
[Ho_2_(tepa)_2_(OH)_2_Cl_2_]_2_[Sn_4_Se_10_] (557)	SnCl_4_·H_2_O, Se, HoCl_3_/tepa,170 °C, 6 days	B^298^
[Ni(teta)(en)][Ni(teta)(hda)][Sn_4_Se_10_] (558)	Sn, Se, Ni(OAc)_2_, hda/teta, 170 °C, 5 days	B^299^
[Ln_2_(tepa)_2_(OH)_2_Cl_2_]_2_[Sn_4_Se_10_] (559–562, Ln = Y, Dy, Er, Tm)	SnCl_4_·5H_2_O, Se, LnCl_3_, Ag/tepa, 180 °C, 6 days	B^300^
[(Me)_2_NH_2_]_6_[Ge_2_Sb_2_S_7_][Ge_4_S_10_] (563)	GeO_2_, Sb, S/DMF, 160 °C, 7 days	B^301^
[Mn(en)_3_]_2_[Ge_4_O_6_Te_4_] (564)	Ge, Te, Mn(OAC)_2_, [Me_4_N]I/en, 150 °C, 80 h	B^302^

a Vio = viologen dication, TMPyP = 5,10,15,20-tetrakis(*N*-methyl-4-pyridyl)porphyrin, DMBPE = *N*,*N*′-dimethyl-1,2-bis(4-pyridinium)-ethylene, cyclam = 1,4,8,11-tetraazacyclotetradecane, trien = triethylentetramin, teta = triethylenetetramine.

**Fig. 13:**
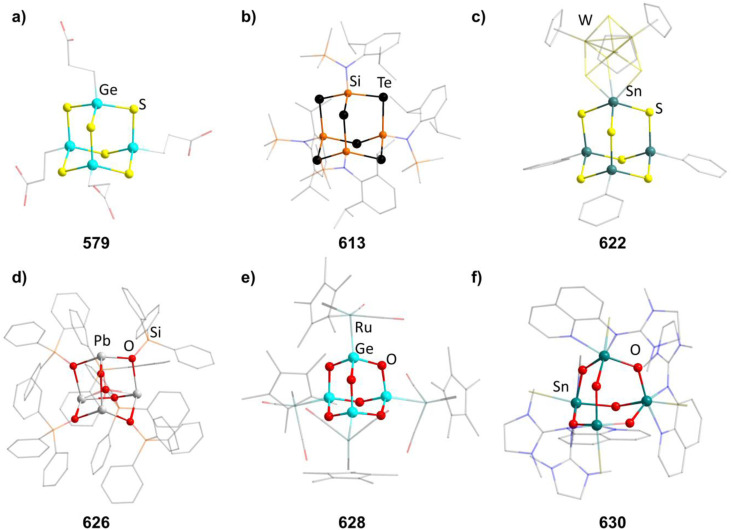
Examples of hybrid adamantane-type cluster compounds with group 14 elements in the Q position and group 16 atoms in the E position: [{HOOC(CH_2_)_2_Ge}_4_S_6_] (579, top left (a)), [(N(SiMe_3_)DippSi)_4_Te_6_] (613, top center (b)), [{(PhSn)_3_SnS_6_}{(WCp)_3_S_4_}] (622, top right (c)), [(μ_4_-O)Pb_4_(OSiPh_3_)_6_] (626, bottom left (d)), [({Cp*(CO)_2_Ru}_2_Ge)_4_O_6_] (628, bottom center (e)) and [(Sn(DMEGqu)Br)_4_O_4_(OH)_2_]Br_2_ (630, bottom right (f)). Hydrogen atoms are omitted for clarity.

The other group contains predominantly neutral clusters with mostly organic ligands of the type [(RQ)_4_E_6_]. While at first reactions were carried out using gaseous H_2_E (E = S, Se) and a group 14 halide RQX_3_,^303–305^ most hybrid materials can be obtained through route D, using a solid or liquid chalcogenide source A_2_E (A = alkaline metal, SiMe_3_; E = S, Se) to prepare 565–612 (Fig. 13).^306–322^ As some of them are sensitive to water, the (SiMe_3_)_2_E precursors are often advantageous for their solubility in organic solvents. The clusters’ structure is heavily influenced by their organic component. In some cases, this leads to an equilibrium between compounds with an adamantane like cluster core architecture and compounds featuring the previously discussed double decker type (see section 2.1.4).^306,307,323^ Especially for tin compounds, back coordinating ligands shift the equilibrium away from the adamantane-type architecture, also resulting in defect heterocubane type arrangements, while some Ge congeners can be obtained in the adamantane topology.^323^

Reactive organic groups on the adamantanes can be used as a site to introduce new functionality. But to prevent the formation of defect heterocubane or double decker type cluster during the addition of Lewis basic ligands to an adamantane core, back-coordination must be prevented by using inflexible ligands.^321^

Tellurium containing adamantanes of the [(RQ)_4_E_6_] type have not been obtained yet by Method D. However, in one example, the silicon cluster [Si_4_{N(SiMe_3_)Dipp}_4_] (Dipp = 2,6-diisopropylphenyl) can be reacted with (^*n*^Bu)_3_PTe to afford the desired [(N(SiMe_3_)DippSi)_4_Te_6_] (613, Fig. 13).^324^

In a unique oxidative addition of a Sn^II^ species N(2,6-^i^Pr_2_C_6_H_3_)(SiMe_3_)SnCl with elemental sulfur or selenium, [{N(2,6-^i^Pr_2_C_6_H_3_)(SiMe_3_)Sn}_4_E_6_] (614–615, E = S, Se) were isolated.^325^

Aside from purely organic ligands, organometallic fragments have also been used to stabilize adamantane-type clusters by the same RSnX_3_ and A_2_E method described above, either with {Cp(CO)_*x*_M} fragments (616–618)^326–328^ or ferrocenyl ligands (619–620).^329,330^

It was also possible to exchange one organic ligand in [(PhSn)_4_S_6_] with a M_3_S_4_ (M = Mo, W) fragment under retention of the adamantane framework by simple addition of [Cp(CO)_3_MCl] and (SiMe_3_)_2_S, resulting in [{(PhSn)_3_SnS_6_}{(MCp)_3_S_4_}] (621–622, Fig. 13).^331^

One case, leading to an anionic adamantane-type structure with a gold counterion, could be realized by the rearrangement of a defect heterocubane type cluster [{Me(O)CCH_2_CMe_2_Sn}_3_S_4_]Cl combined with a ligand extension to [Au(dppe)_2_][{Me(H_2_NN)CCH_2_CMe_2_Sn}_4_S_6_Cl] (623) in the presence of a gold complex.^332^

Compounds with oxygen in the E position are much rarer with only seven examples, one of which is the only known Pb containing adamantane [(μ_4_-O)Pb_4_(OSiPh_3_)_6_] (624, Fig. 13), featuring an endohedral μ_4_-O atom and silanolate μ-bridging groups.^333,334^624 was isolated after a reaction of plumbocene with Ph_3_SiOh in Et_2_O.

The stoichiometric hydrolysis of RSiCl_3_ with bulky R leads to the formation of adamantane type clusters [(RSi)_4_O_6_] (625–626, R = ^*t*^Bu, ^i^Pr), as the polymeric species are inhibited due to steric reasons.^335^

A reaction more closely related to the synthesis of the higher chalcogenide congeners is utilized for [{(Me_3_Si)_3_CSn}_4_O_6_] (627), which is made by combining (Me_3_Si)_3_CSnCl_3_ with Na_2_O.^317^

Two further examples obtained from hydrolysis are stabilized by transition metal fragments (628–629),^336,337^ with the last one being a cationic species [{Sn(DMEGqu)Br}_4_O_4_(OH)_2_]Br_2_ (630, DMEGqu = *N*-(1,3-dimethylimidazolidin2-ylidene)quinoline-8-amine) formed by SnBr_4_, DMEGqu and H_2_O and exhibiting a coordination number of 6 at the Sn center, unusual for adamantane-type structures.^338^

**Table 13:** Hybrid adamantane-type cluster compounds with group 14 elements in the Q position and group 16 atoms in the E position^a^

Compound	Reagents/conditions	Method
[(MeSi)_4_S_6_] (565)	MeSiCl_3_, H_2_S/200 °C, 12 h	F^304,305^
[(EtSi)_4_S_6_] (566)	EtSiCl_3_, H_2_S/150 °C, 3 h	F^304^
[(ThexSi)_4_S_6_] (567)	1. Li_2_S, ThexSiCl_3_/THF, 0 °C to RT, 14 days	D^306,307^
	2. Decaline, 195 °C, 24 h	
[(PhSi)_4_S_6_] (568)	PhSiCl_3_, Na_2_S/THF, 0 °C to RT, 24 h	D^310^
[(RSi)_4_S_6_] (569–570, R = 1-Np, Sty)	Na_2_S, 1-NpSiCl_3_/THF, 0 °C, 18 h	D^309^
[(MeSi)_4_Se_6_] (571)	MeSiCl_3_, H_2_Se/400 °C, 1 h	F^304^
[(EtSi)_4_Se_6_] (572)	EtSiCl_3_, H_2_Se, Al/150 °C, 3 h	F^304^
[(ThexSi)_4_Se_6_] (573)	1. Li_2_S, ThexSiCl_3_/THF, 0 °C to RT, 5 days	D^306,307^
	2. Decaline, 150 °C, 3 h	
[(PhSi)_4_Se_6_] (574)	Na_2_Se, PhSiCl_3_/THF, 0 °C, 18 h	D^308^
[(MeGe)_4_S_6_] (575)	MeGeBr_3_, H_2_S, NEt_3_/C_6_H_6_, 80 °C, 1 h	D^311,312^
[(EtGe)_4_S_6_] (576)	EtGeCl_3_, (SiH_3_)_2_S, Al_2_Cl_6_/CS_2_, 75 °C, 7 days	D^313^
[(CF_3_Ge)_4_S_6_] (577)	CF_3_GeCl_3_, (SiH_3_)_2_S, Al_2_Cl_6_/CS_2_, 80 °C, 10 days	D^313^
[(ThexGe)_4_S_6_] (578)	1. Li_2_S, ThexGeCl_3_/THF, 0 °C to RT, 24 h	D^306,307^
	2. Decaline, 195 °C, 24 h	
[{HOOC(CH_2_)_2_Ge}_4_S_6_] (579)	HOOC(CH_2_)_2_GeCl_3_, Na_2_S/Me_2_CO, H_2_O, 3 h	D^323^
[{Me(O)CCH_2_CMe_2_Ge}_4_S_6_] (580)	MeOCCH_2_CMe_2_GeCl_3_, Na_2_S/Me_2_CO, H_2_O, 4 h	D^323^
[{NC(CH_2_)_2_Ge}_4_S_6_] (581)	NC(CH_2_)_2_GeCl_3_, Na_2_S/Me_2_CO, H_2_O, 5 h	D^314^
[(PhGe)_4_S_6_] (582)	PhGeCl_3_, Na_2_S/Me_2_CO, H_2_O, 1 h	D^310^
[(CF_3_Ge)_4_Se_6_] (583)	CF_3_GeCl_3_, (SiH_3_)_2_Se, Al_2_Cl_6_/*n*-hexane, 110 °C, 4 days	D^313^
[(ThexGe)_4_Se_6_] (584)	1. Li_2_Se, ThexGeCl_3_/THF, 0 °C to RT, 24 h	D^306,307^
	2.C_6_H_6_, 80 °C, 24 h	
[{NC(CH_2_)_2_Ge}_4_Se_6_] (585)	NC(CH_2_)_2_GeCl_3_, Na_2_Se/THF, 30 h	D^314^
[(MeSn)_4_S_6_] (586)	MeSnI_3_, H_2_S, HCl/H_2_O or MeSnCl_3_, Na_2_S/Me_2_CO, H_2_O, 3 h	G/D^303,304,315,316^
[(PhSn)_4_S_6_] (587)	PhSnCl_3_, Na_2_S/Me_2_CO, H_2_O, 4 h	D^304^
[(^*n*^BuSn)_4_S_6_] (588)	^*n*^BuSnCl_3_, Na_2_S/Me_2_CO, H_2_O, 3 h	D^304,316,317^
[(^*n*^PrSn)_4_S_6_] (589)	(^*n*^PrSn)_2_O_3_, Na_2_S, HCl/H_2_O, 3 h	D^316^
[(mesSn)_4_S_6_] (590)	mesSnCl_3_, Na_2_S/H_2_O, Me_2_CO, 0 °C, 12 h	D^319^
[(1-NpSn)_4_S_6_] (591)	1-NpSnCl_3_, Na_2_S/H_2_O, Me_2_CO, 0 °C, 18 h	D^319^
[(4-MeC_6_H_4_Sn)_4_S_6_] (592)	4-MeC_6_H_4_SnCl_3_, Na_2_S/H_2_O, Me_2_CO, 0 °C, 4 h	D^319^
[(4-MeOC_6_H_4_Sn)_4_S_6_] (593)	4-MeOC_6_H_4_SnCl_3_, Na_2_S/H_2_O, Me_2_CO, 0 °C, 2 h	D^319^
[(4-FC_6_H_4_Sn)_4_S_6_] (594)	4-FC_6_H_4_SnCl_3_, Na_2_S/H_2_O, Me_2_CO, 0 °C, 14 h	D^319^
[(3-FC_6_H_4_Sn)_4_S_6_] (595)	3-FC_6_H_4_SnCl_3_, (SiMe_3_)_2_S/10 °C, 1 h	D^319^
[(C_6_F_5_Sn)_4_S_6_] (596)	C_6_F_5_SnCl_3_, (SiMe_3_)_2_S/C_6_H_6_, 10 °C, 15 min	D^319^
[{(Me_3_Si)_3_CSn}_4_S_6_] (597)	[(Me_3_Si)_3_CSnBr_3_], Na_2_S/NH_3_, 24 h	D^317^
[(StySn)_4_S_6_] (598)	PhSnCl_3_, Na_2_S/THF, 0 °C to RT, 24 h	D^320^
[(CySn)_4_S_6_] (599)	CySnCl_3_, (SiMe_3_)_2_S/toluene, 24 h	D^321^
[(BnSn)_4_S_6_] (600)	BnSnCl_3_, (SiMe_3_)_2_S/toluene, 5 min	D^321^
[{EtO_2_C(C_6_H_4_)CH_2_CH_2_Sn}_4_S_6_] (601)	EtO_2_C(C_6_H_4_)CH_2_CH_2_SnCl_3_, (SiMe_3_)_2_S/toluene, 2 h	D^321^
[(CpSn)_4_S_6_] (602)	1. SnCl_4_, NaCp/toluene, 0 °C, 5 h	D^321^
	2. (SiMe_3_)_2_S/toluene, 1 h	
[(MeSn)_4_Se_6_] (603)	MeSnBr_3_, NaHSe, Na[BH_4_]/H_2_O, 1 h	D^318^
[(^*n*^BuSn)_4_Se_6_] (604)	Na_2_Se, ^*n*^BuSnCl_3_/NH_3_, −33 °C, 5 h	D^317^
[{(Me_3_Si)_3_CSn}_4_Se_6_] (605)	[(Me_3_Si)_3_CSnBr_3_], Na_2_Se/NH_3_, 24 h	D^317^
[(^i^PrSn)_4_Se_6_] (606)	^i^PrSnCl_3_, Na_2_S/H_2_O, Me_2_CO, 0 °C, 18 h	D^322^
[(PhSn)_4_Se_6_] (607)	PhSnCl_3_, (SiMe_3_)_2_Se/toluene, 5 min	D^321^
[(BnSn)_4_Se_6_] (608)	BnSnCl_3_, (SiMe_3_)_2_Se/toluene, 5 min	D^321^
[EtO_2_C(C_6_H_4_)CH_2_CH_2_Sn)_4_Se_6_] (609)	EtO_2_C(C_6_H_4_)CH_2_CH_2_SnCl_3_, (SiMe_3_)_2_Se/toluene, 16 h	D^321^
[(CpSn)_4_Se_6_] (610)	1. SnCl_4_, NaCp/toluene, 0 °C, 5 h	D^321^
	2. (SiMe_3_)_2_S/toluene, 5 min	
[(CySn)_4_Se_6_] (611)	CySnCl_3_, (SiMe_3_)_2_Se/toluene, 1 h	D^321^
[{Me(PhNHN)CCH_2_CMe_2_Ge}_4_S_6_] (612)	[(MeOCCH_2_CMe_2_Ge)_4_S_6_], H_2_NNHPh/CH_2_Cl_2_, 3 h	Q^323^
[(N(SiMe_3_)DippSi)_4_Te_6_] (613)	[Si_4_{N(SiMe_3_)Dipp}_4_], (^*n*^Bu)_3_PTe/toluene, 110 °C, 2 h	J^324^
[{N(2,6-^i^Pr_2_C_6_H_3_)(SiMe_3_)Sn}_4_E_6_] (614–615, E = S, Se)	N(2,6-^i^Pr_2_C_6_H_3_)(SiMe_3_)SnCl, E/THF, 18 h	C^325^
[({Cp(CO)_2_Fe}Sn)_4_Se_6_] (616)	[{Cp(CO)_2_Fe}_2_SnCl_2_], (SiMe_3_)_2_Se/THF	D^326^
[({Cp(CO)_3_Mo}Sn)_4_Te_6_] (617)	[{Cp(CO)_3_Mo}SnCl_3_], (SiMe_3_)_2_Te/THF, −78 to −18 °C, 19 days	D^327^
[({Cp(CO)Fe}_2_Sn)_4_S_6_] (618)	[{Cp(CO)_2_Fe}SnCl_3_], (Bu_3_Sn)_2_S/toluene, 12 h	D^328^
[(FcSn)_4_S_6_] (619)	FcSnCl_3_, Na_2_S/THF, 0 °C, 31 h	D^329^
[(FcSn)_4_Se_6_] (620)	FcSnCl_3_, K_2_Se/THF, 48 h	D^330^
[{(PhSn)_3_SnS_6_}{(MCp)_3_S_4_}] (621–622, M = Mo, W)	[(PhSn)_4_S_6_] (587), [M(CO)_3_CpCl], (Me_3_Si)_2_S/THF, 15 h	R^331^
[Au(dppe)_2_][{Me(H_2_NN)CCH_2_CMe_2_Sn}_4_S_6_Cl] (623)	1. [{Me(O)CCH_2_CMe_2_Sn}_3_S_4_]Cl, AuCl, dppe, (Me_3_Si)_2_S,/CH_2_Cl_2_, 17 h	R^332^
	2. PhNHNH_2_/CH_2_Cl_2_, 45 min	
[(μ_4_-O)Pb_4_(OSiPh_3_)_6_] (624)	Ph_3_SiOH, [Pbcp_2_]/Et_2_O, 35 °C, 30, min	D^333,334^
[(^*t*^BuSi)_4_O_6_] (625)	^*t*^BuSiCl_3_, H_2_O/Et_2_O, 24 h	I^335^
[(^i^PrSi)_4_O_6_] (626)	^i^PrSiCl_3_, H_2_O/Et_2_O, −80 °C to RT, 2 days	I^335^
[{(Me_3_Si)_3_CSn}_4_O_6_] (627)	[(Me_3_Si)_3_CSnBr_3_], Na_2_O/NH_3_, THF, −78 °C, 6 h	D^317^
[({Cp*(CO)_2_Ru}_2_Ge)_4_O_6_] (628)	1. [Cp*RuCO(GeCl_2_)]_2_, K/THF, 48 h	I^336^
	2. O_2_	
[{(CO)_5_WGe}_4_O_2_(OH)_4_] (629)	1. 2-Methoxybenzyl alcohol, Ge[N(SiMe_3_)_2_]_2_/Et_2_O, 30 min	I^337^
	2. [W(CO)_5_(thf)]/THF, 12 h	
	3. H_2_O in pentane	
[(Sn(DMEGqu)Br)_4_O_4_(OH)_2_]Br_2_ (630)	SnBr_4_, 3,5-ditert-butyl-*o*-benzoquinone, DMEGqu/THF, H_2_O	I^338^

a Thex = 1,1,2-trimethylpropyl, Np = naphthyl, Sty = *para*-styryl, Cy = cyclohexyl, Cp = cyclopentadienyl, Dipp = 2,6-diisopropylphenyl, DMEGqu = *N*-(1,3-dimethylimidazolidin2-ylidene)quinoline-8-amine.

##### 2.1.5.13 Group 15/16 adamantane-type clusters

The simplest adamantanes with the combination of 15/16 elements are P_4_O_10_, P_4_O_6_, P_4_S_10,_ P_4_Se_10_ or As_4_O_10_. They are often used as precursors for further derivatives.

Simple derivatization reactions on [P_4_O_6_] can be carried out by adding terminal chalcogenide groups to the P moieties, oxidizing them from their +III to a +V state. A straightforward method is the thermal oxidation reaction in the presence of trace amount of water to form [P_4_O_7_] (631).^339–342^ Ligand exchange reactions using [P_4_S_10_] or [P_4_Se_10_] can be used with [P_4_O_6_] to obtain the series [(P_4_O_6_S_*x*_] (632–635, *x* = 1–4) and [(P_4_O_6_Se_*x*_] (636–638, *x* = 1–3) with the four fold substituted selenium compound not being achieved due to the lower reactivity of the reagent, which would make temperatures above the decomposition point necessary.^343,344^ By employing this strategy and starting from 632, a mixed S/Se compound [(P_4_O_6_SSe] (639) is accessible as well.^343^ [(SP)_4_O_6_] (635) can also be obtained by reacting [P_4_O_6_] with elemental sulfur.^345–347^ Repeating the reactions with [P_4_O_7_] gives the corresponding mixed terminated adamantane-type structures [P_4_O_7_S_*x*_] (640–642, *x* = 1–3) and [P_4_O_7_Se] (643), with impurities of [P_4_O_8_] (644) and [P_4_O_8_S_*x*_] (645–646, *x* = 1–2) being found in the sulfur containing reaction mixture.^343,348,349^

P_4_O_6_ could also be used as a non-chelating tetradentate ligand to coordinate to Ni(CO)_4_ in a solventless reaction at room temperature.^350–352^ Depending on the ratio used, the complexes [P_4_O_6_{Ni(CO)_3_}_*x*_] (647–650, *x* = 1–4) or [(P_4_O_6_)_*x*_Ni(CO)_4−*x*_] (651–652, *x* = 2–3) could be obtained if one reactant is given in excess. Using a stoichiometric ratio, the formation of coordination polymers has been reported. Reactions with the iron carbonyl [Fe(CO)_5_] can similarly be carried out, but proceed much slower and at higher temperatures (653–656).^351,353^

**Fig. 14:**
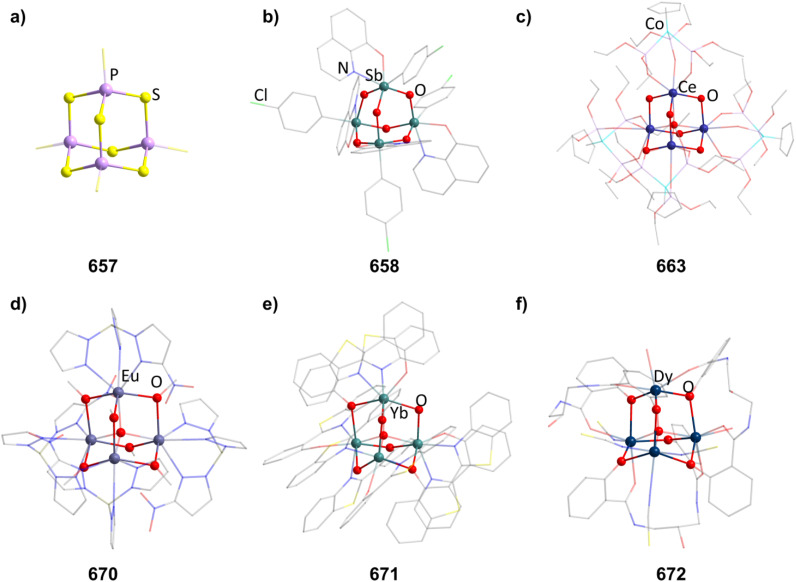
Examples of adamantane-type with group 15 and lanthanide elements in the Q position and group 16 atoms in the E position: [P_4_S_10_] (657, top left), [{(8-HQ)(*p*-Cl-C_6_H_4_)Sb}_4_O_6_] (658, top center (a)), [(μ_4_-O){Ce(L_OEt_)}_4_O_4_(OH)_2_] (663, top right (b)), [(μ_4_-O){Eu(3-NO_2_Tp)}_4_(μ_2_-OMe)_6_] (670, bottom left (c)), [(μ_4_-O){(SON)Yb}_4_(SON)_4_(OH)_2_] (671, bottom center (d)) and [(μ_4_-O)Dy_4_(HL)_3_(SCN)_4_(H_2_O)_2_] (672, bottom right (e)). Hydrogen atoms are omitted for clarity.

[P_4_S_10_] (657, Fig. 14) is most easily obtained from the elements using Method A, though many methods are available.^354–356^

Arylstibonic acids, RSbO_3_H_2_, can be used as precursors for adamantane-type structures with six coordinated Sb sites (658–661, Fig. 14) in combination with N,O-chelating ligands which trigger the rearrangement at elevated temperatures.^357^ A similar compound can also be achieved by treating the C,P-coordinated Sb complex (dpan)SbCl_4_ (dpan = 6-diphenylphosphinoacenaphth-5-yl) with a basic aqueous solution, yielding [{(dpan)(OH)Sb}_4_O_6_] (662).^358^

##### 2.1.5.14 Lanthanide/group 16 adamantane-type clusters

Lanthanide atoms occupying positions within the adamantane-type scaffolds are only known in combination with oxygen in the E position for a number of oxygen centered compounds. In similarity to clusters with hydrogen (see section 2.1.1), related compounds derived from the adamantane-type architecture, in which some atoms in the E positions are formally replaced by two oxygen bridges are also known, but will not be further discussed here.^359–365^ In either case, the lanthanides prefer higher coordination numbers, often resulting in multiple or multidentate ligands.

[(μ_4_-O){Ce(L_OEt_)}_4_O_4_(OH)_2_] (663, Fig. 14) was the first example of such a compound, featuring the tripodal ligand L_OEt_ = [Co(η^5^-C_5_H_5_){P(O)(OEt)_2_}_3_]^−^^366^ It was realized by the addition of [Et_4_N]OH to [L_OEt_Ce(NO_3_)_3_], which led to a mix of oxo and hydroxy bridges. It is possible to treat this compound with H_2_O_2_, which will result in exchanging the oxo bridges with η^2^-O_2_ units in the Q position (664).

The series of clusters [(μ_4_-O){M(3-NO_2_Tp)}_4_(μ_2_-OMe)_6_] (665–670, M = Pr–Tb; 3-NO_2_Tp = 3-nitrotrispyrazolylborate, Fig. 14) also comprises a tripodal ligand on each metal center, but methoxy groups in the E position.^367^ The reaction path also involved the formation of the monomeric metal complex by addition of MCl_3_ to [Bu_4_N][3-NO_2_Tp] in the presence of methanol.

Another study resulted in a compound in which most oxygen atoms are part of a bridging ligand directly connected to the metal centers.^368^ [(μ_4_-O){(SON)Yb}_4_(SON)_4_(OH)_2_ (671, SON = (benzothiazole-2-yl)phenolate, Fig. 14) contains SON ligands with two different connecting modes: chelating a single Yb site or connecting two such atoms *via* one of its oxygens and two E positions.

Two clusters, [(μ_4_-O)M_4_(HL)_3_(SCN)_4_(H_2_O)_2_] (672–673, M = Dy, Eu, Fig. 14), were constructed by arranging the metal atoms stemming from M(SCN)_3_ around two polydentate ligands 2-hydroxy-*N*-[2-hydroxy-3-[(2hydroxybenzoyl)amino]propyl]benzamide (H_3_L), which comprise all oxygen atoms in the E position.^369^

**Table 14:** Adamantane-type with group 15 and lanthanide elements in Q position and group 16 atoms in the E position^a^

Compound	Reagents/conditions	Method
[P_4_O_7_] (631)	P_4_O_6_, H_2_O/diglyme, 140 °C	P^339–342^
[(P_4_O_6_S_*x*_] (632–635, *x* = 1–4)	P_4_O_6_, [P_4_S_10_]/PhMe, 110 °C	P^343^
[(SP)_4_O_6_] (635)	P_4_O_6_, S/160 °C	P^345–347^
[(P_4_O_6_Se_*x*_] (636–638, *x* = 1–3)	P_4_O_6_, [P_4_Se_10_]/PhMe, 110 °C	P^343,344^
[(P_4_O_6_SSe] (639)	[P_4_O_6_S] (632), [P_4_Se_10_]/PhMe, 110 °C	P^343^
[P_4_O_7_S_*x*_] (640–642, *x* = 1–3)	[P_4_O_7_] (631), [P_4_S_10_]/PhMe, 110 °C	P^343,348^
[P_4_O_7_Se] (643)	[P_4_O_7_] (631), [P_4_Se_10_]/PhMe, 110 °C	P^343,349^
[P_4_O_8_] (644)	[P_4_O_7_] (631), [P_4_S_10_]/PhMe, 110 °C	P^343^
[P_4_O_8_S_*x*_] (645–646, *x* = 1–2)	[P_4_O_7_] (631), [P_4_S_10_]/PhMe, 110 °C	P^343^
[P_4_O_6_{Ni(CO)_3_}_*x*_] (647–650, *x* = 1–4)	P_4_O_6_, Ni(CO)_4_/10 min	P^350–352^
[(P_4_O_6_)_*x*_Ni(CO)_4−*x*_] (651–652, *x* = 2–3)	P_4_O_6_, Ni(CO)_4_/10 min	P^350,351^
[P_4_O_6_{Fe(CO)_4_}_*x*_] (653–656, *x* = 1–4)	P_4_O_6_, [Fe(CO)_5_]/103 °C, 24 h	P^351,353^
[P_4_S_10_] (657)	P, S,/100 °C	A^354–356^
[{(8-HQ)(*p*-X-C_6_H_4_)Sb}_4_O_6_] (658–659, X = Cl, Br)	*p*-X-C_6_H_4_SbO_3_H_2_, 8-HQ/toluene, 110 °C, 6 h	C^357^
[{(H_2_naphpz)(*p*-X-C_6_H_4_)Sb}_4_O_6_] (660–661, X = Cl, Br)	*p*-X-C_6_H_4_SbO_3_H_2_, H_2_naphpz/toluene, 110 °C, 6 h	C^357^
[{(dpan)(OH)Sb}_4_O_6_] (662)	dpanSbCl_4_, NaOH/H_2_O, Et_2_O, 18 h	I^358^
[(μ_4_-O){Ce(L_OEt_)}_4_O_4_(OH)_2_] (663)	[Et_4_N]OH, [CeL_OEt_(NO_3_)_3_]/MeCN, 1 h	C^366^
[(μ_4_-O){Ce(L_OEt_)}_4_(O_2_)_4_(OH)_2_] (664)	[(μ_4_-O){Ce(L_OEt_)}_4_O_4_(OH)_2_] (663), H_2_O_2_/MeCN, 1 h	I^366^
[(μ_4_-O){M(3-NO_2_Tp)}_4_(μ_2_-OMe)_6_] (665–666, M = Gd, Tb)	MCl_3_·H_2_O, [Bu_4_N][3-NO_2_Tp]/MeOH, 3 days	C^367^
[(μ_4_-O){M(3-NO_2_Tp)}_4_(μ_2_-OMe)_6_] (667–670, M = Pr, Nd, Sm, Eu)	MCl_3_·H_2_O, [Bu_4_N][3-NO_2_Tp]/MeOH, 2–4 weeks	C^367^
[(μ_4_-O){(SON)Yb}_4_(SON)_4_(OH)_2_] (671)	Yb[N(SiMe_3_)_2_], HBT/DME, H_2_O, 30 °C, 1 h	I/K^368^
[(μ_4_-O)M_4_(HL)_3_(SCN)_4_(H_2_O)_2_] (672–673, M = Dy, Eu)	H_3_L, Et_3_N, M(SCN)_3_·6H_2_O/MeOH, MeCN, 100 °C, 2 days	K^369^

a 8-HQ = 8-hydroxyquinoline, H_2_naphpz = 2-[1*H*-pyrazol-5(3)-yl]naphthalene-1-ol, dpan = 6-diphenylphosphinoacenaphth-5-yl, L_OEt_ = [Co(η^5^-C_5_H_5_){P(O)(OEt)_2_}_3_]^−^, 3-NO_2_Tp = 3-nitrotrispyrazolylborate, SON = (benzothiazole-2-yl)phenolate, HBT = 2-(2-hydroxyphenyl)benzothiazole, H_3_L = 2-hydroxy-*N*-[2-hydroxy-3-[(2hydroxybenzoyl)amino]propyl]benzamide.

#### 2.1.6 Q/group 17 adamantane-type clusters

Group 17 elements only occur in the E position in adamantane-like structures and mainly in (oxygen centered) copper clusters, although there are a few examples outside of this elemental combination, which will be discussed first. While the compounds with the higher congeners Cl, Br and I comprise no further, or only one, ligand at the Q atom, all examples for species with F carry three ligands to expand the coordination sphere on the Q atom to six. Nearly all compounds are produced from elements or simple binary compounds under addition of an appropriate counterion, which is often important for the formation of an adamantane-type scaffold over other structural motifs.

A study showed the formation of simple anionic [Be_4_Cl_10_]^2−^ compounds (in 674–678) with various cations in solid state reactions of BeCl_2_ and chlorides.^370^

**Fig. 15:**
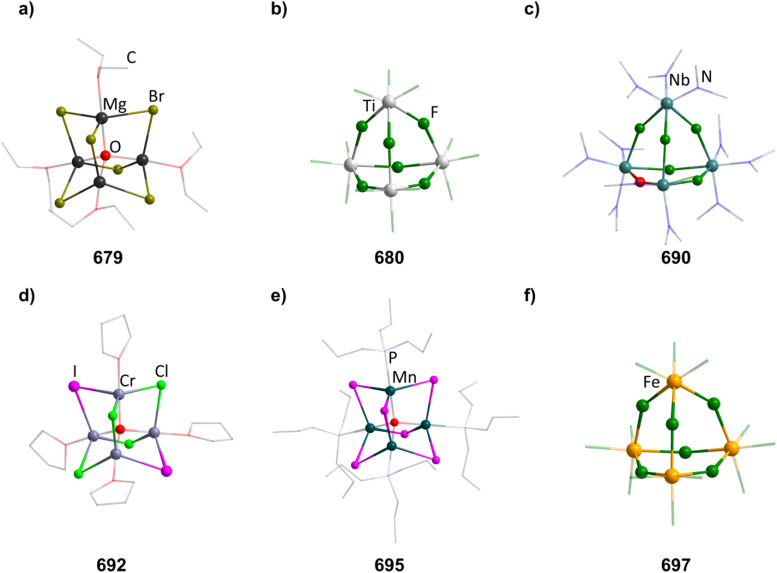
Examples of adamantane-type clusters with group 2 and 4–8 atoms in the Q position and group 17 atoms in the E position: [μ_4_-O{Mg(Et_2_O)}_4_Br_6_] (679, top left), [{Nb)NMe_2_)_3_}_4_{Nb)NMe_2_)_2_}F_5_O]Cl_2_ (680, top center (a)), [{Nb)NMe_2_)_3_}_4_{Nb)NMe_2_)_2_}F_5_O]Cl_2_ (690, top right (b)), [μ_4_-O{Cr(thf)}_4_Cl_4_I_2_] (692, bottom left (c)), [μ_4_-O{Mn(P^*n*^Pr_3_)}_4_Cl_6_] (695, bottom center (d)) and [H8-HQ]_6_[(FeF_3_)_4_F_6_] (697, bottom right (e)). Hydrogen atoms and counterions, if present, are omitted for clarity.

There is an oxygen centered example of a magnesium adamantane-type cluster [μ_4_-O{Mg(Et_2_O)}_4_Br_6_] (679, Fig. 15) prepared by directly reacting the Grignard reagent PhMgBr with O_2_ in ether.^371,372^

Titanium mostly forms adamantane-type clusters of the composition [(TiF_3_)_4_F_6_]^2−^ (680–685, Fig. 15). All of them are formed from TiF_4_ in the presence of an appropriate counterion complex, such as crown ether coordinated alkaline metals, ammonium or phosphonium cations.^373–375^ These reactions can be carried out in conventional solvents like MeCN or in liquid HF.

In the presence of a macrocyclic arene during the formation of the adamantane, coordination to two Ti moieties under elimination of two fluorines at each position was observed, leading to [(TiCl_3_)_2_(Ti_2_{da6aH_2_(H_2_)})F_6_] (686, da6aH_6_ = *p*-methyl-dimethyldiazacalix[6]areneH_6_).^376^ Another formal, but this time complete, exchange of the terminal fluorine atoms by chlorine was observed for [C_4_mim]_2_[(TiCl_3_)_4_F_6_] (687, C_4_mim = 1-butyl-3-methylimidazolium) obtained after an ionothermal reaction of TiCl_4_ under decomposition of the [BF_4_] counterion of the ionic liquid.^377^

The cage compound [{Nb)NMe_2_)_3_}_4_F_6_]Cl_2_ (688) is obtainable by a synthesis using NbF_5_ and Me_3_SiNMe_2_ in chloroform and toluene.^378^ While the anion is exchanged by Br in CH_2_Br_2_ (689), dissolving the cluster in H_2_O exchanges one of the F atoms in the E position with an O atom and eliminates a ligand to form [{Nb(NMe_2_)_3_}_4_{Nb(NMe_2_)_2_}F_5_O]Cl (690, Fig. 15).

A Cr compound [μ_4_-O{Cr(thf)}_4_Cl_6_] (691) with a central oxygen and coordinated solvent molecule very similar to the Mg species 679 was obtained from CrCl_2_, ^*n*^BuLi and LiOH·H_2_O in THF.^379^

Two derivatives with both Cl and I sites in the E position [μ_4_-O{Cr(solv)}_4_Cl_4_I_2_] (692–693, solv = THF, tetrahydropyran (thp), Fig. 15), could be found in small quantities while trying to synthesize the methylidine complexes [Cr_3_Cl_3_(μ-Cl)_3_(μ_3_-CH)(solv)_6_].^380^

A tungsten congener in oxidation state V+ features an anionic fluorine scaffold in [Cp_2_WCl_2_]_2_[(WF_3_)_4_F_6_] (694), resulting from the comproportionation reaction of WF_6_ and [Cp_2_WCl_2_].^381^

The Mn analogs [μ_4_-O{Mn(PR_3_)}_4_Cl_6_] (695–696, R_3_ = ^*n*^Pr_3_, PhMe_2_, Fig. 15) were prepared by bubbling O_2_ through an anhydrous solution of [MnI_2_(PR_3_)].^382,383^

Another fluorine cluster [H8-HQ]_6_[(FeF_3_)_4_F_6_] (8-HQ = 8-hydroxyquinoline, 697, Fig. 15) was isolated after a solvothermal reaction of FeF_2_, FeF_3_ and 8-HQ in the presence of HF.^384^

**Table 15:** Adamantane-type clusters with group 2 and 4–8 atoms in Q position and group 17 atoms in E position^a^

Compound	Reagents/conditions	Method
[H_4_N]_2_[Be_4_Cl_10_] (674)	BeCl_2_, NH_4_Cl/400–230 °C	A^370^
Cs_2_[Be_4_Cl_10_] (675)	BeCl_2_, CsCl/400–230 °C	A^370^
Rb_2_[Be_4_Cl_10_] (676)	BeCl_2_, RbCl/400–230 °C	A^370^
K_2_[Be_4_Cl_10_] (677)	BeCl_2_, KCl/400–230 °C	A^370^
Tl_2_[Be_4_Cl_10_] (678)	BeCl_2_, TlCl/400–230 °C	A^370^
[μ_4_-O{Mg(Et_2_O)}_4_Br_6_] (679)	BrMgPh, O_2_/Et_2_O	G^371,372^
[TiF_2_(15-crown-5)][(TiF_3_)_4_F_6_] (680)	TiF_4_, 15-crown-5/MeCN	C^373^
[*o*-C_6_H_4_(PPh_2_H)_2_][(TiF_3_)_4_F_6_] (681)	TiF_4_, *o*-C_6_H_4_(PPh_2_)_2_/MeCN, CH_2_Cl_2_, 1 h	C^374^
[*o*-C_6_H_4_(AsMe_2_H)_2_][(TiF_3_)_4_F_6_] (682)	TiF_4_, *o*-C_6_H_4_(AsMe_2_)_2_/MeCN, CH_2_Cl_2_, 1 h	C^374^
[H^i^PrS(CH_2_)_2_S^i^PrH][(TiF_3_)_4_F_6_] (683)	TiF_4_, ^i^PrS(CH_2_)_2_S^i^Pr/MeCN, CH_2_Cl_2_, 1 h	C^374^
[Me_4_N]_2_[(TiF_3_)_4_F_6_] (684)	TiF_4_, [Me_4_N]F/HF, −196 K to RT	F^375^
[Ph_4_P]_2_[(TiF_3_)_4_F_6_] (685)	TiF_4_, [Ph_4_P]F/HF, −196 K to RT	F^375^
[(TiCl_3_)_2_(Ti2{da6aH_2_(H_2_)})F_6_] (686)	TiF_4_, *p*-methyl-dimethyldiazacalix[6]areneH_6_/toluene, 110 °C	C^376^
[C_4_mim]_2_[(TiCl_3_)_4_F_6_] (687)	TiCl_4_, [C_4_mim][BF_4_]/70 °C	B^377^
[{Nb)NMe_2_)_3_}_4_F_6_]Cl_2_ (688)	NbF_5_, Me_3_SiNMe_2_/toluene, CHCl_3_	C^378^
[{Nb)NMe_2_)_3_}_4_F_6_]Br_2_ (689)	[{Nb(NMe_2_)_3_}_4_F_6_]Cl_2_ (688)/CH_2_Br_2_	O^378^
[{Nb)NMe_2_)_3_}_4_{Nb)NMe_2_)_2_}F_5_O]Cl_2_ (690)	[{Nb(NMe_2_)_3_}_4_F_6_]Cl_2_ (688)/H_2_O	I^378^
[μ_4_-O{Cr(thf)}_4_Cl_6_] (691)	LiOH·H_2_O, ^*n*^BuLi, CrCl_2_/THF, hexane	C^379^
[μ_4_-O{Cr(thf)}_4_Cl_4_I_2_] (692)	[Cr_3_Cl_3_(μ-Cl)_3_(μ_3_-CH)(thf)_6_], benzaldehyde/THF	J^380^
[μ_4_-O{Cr(thp)}_4_Cl_4_I_2_] (693)	CrCl_2_, CHI_3_/THP, −35 °C to RT, 19 h	C^380^
[Cp_2_WCl_2_]_2_[(WF_3_)_4_F_6_] (694)	[Cp_2_WCl_2_], WF_6_/SO_2_	C^381^
[μ_4_-O{Mn(P^*n*^Pr_3_)}_4_Cl_6_] (695)	[MnI_2_(P^*n*^Pr_3_)], O_2_/*n*-pentane	F^382^
[μ_4_-O{Mn(P^*n*^PhMe_2_)}_4_Cl_6_] (696)	[MnI_2_(P^*n*^PhMe_2_)], O_2_/*n*-pentane	F^383^
[H8-HQ]_6_[(FeF_3_)_4_F_6_] (697)	FeF_2_, FeF_3_, 8-HQ, HF/H_2_O, EtOH, 120 °C, 72 h	B^384^

a C_4_mim = 1-butyl-3-methylimidazolium, da6aH_6_ = *p*-methyl-dimethyldiazacalix[6]areneH_6_.

##### 2.1.6.1 Group 11/17 adamantane-type clusters with a central μ_4_-O atom

Compounds with copper form by far the biggest group of this combination. The vast majority of compounds with Cl and Br in the E position comprise a central oxygen atom and will be discussed first.

**Fig. 16:**
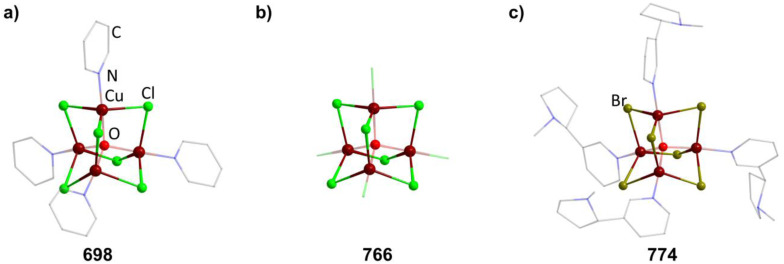
Examples of adamantane-type clusters with a central μ_4_-oxygen atom, Cu in the Q position and group 17 atoms in the E position: [μ_4_-O{Cu(Py)}_4_Cl_6_] (698, left (a)), [Me_4_N]_4_[μ_4_-O(CuCl)_4_Cl_6_] (766, center (b)) and [μ_4_-O{Cu(nicotine)}_4_Br_6_] (774, right (c)). Hydrogen atoms and counterions, if present, are omitted for clarity.

The first compounds discovered were the neutral Cu^II^ complexes of the type [μ_4_-O{Cu(L)}_4_Cl_6_] (698–758, Fig. 16) with L being a neutral ligand. They were isolated after an addition of simple CuCl_*x*_ to L in the presence of ambient air, hydroxide or CuO.^385–436^ In some of those cases, the oxygen source could not be determined and is most likely a H_2_O or O_2_ impurity in the reaction, or stems from decomposition of the solvent. A deviating synthetic strategy uses oligomeric [LCuCl]_*x*_ complexes already containing the desired ligand, which rearrange to the desired product.^437–439^ The clusters [μ_4_-O{Cu(solv)}_4_Cl_6_] (707 and 737, solv = MeCN, MeOH) can also be used in ligand exchange reactions to generate different compounds with more Lewis-basic ligands (748–749).^394,440^ A unique approach was taken in the formation of [μ_4_-O{Cu(Amt)}_4_Cl_6_] (758, Amt = 1,3-diamino-1,2,2-trimethylcyclopentane), which is formed after the ligand in [Cu(α-CgPAmtHMe)(Cl)][BF_4_] (CgP = 1,3,5,7-tetramethyl-2,4,6-trioxa-8-phosphatricyclo[3.3.1.1]-decane) decomposes after addition of KHMDS.^441^

Heterogenous substitution is possible as well in cases where multiple coordinating molecules are present (759–765).^401,442–444^

Anionic clusters can be generated when not all chloride atoms are substituted by a ligand during the reaction.^416,436^ When no coordinating ligand is present, tetraanions [μ_4_-O(CuCl)_4_Cl_6_]^4−^ (in 766–772, Fig. 16) can be isolated readily with different counterions.^445–451^

While not as extensively studied, the Br homologs [μ_4_-O{Cu(L)}_4_Br_6_] (773–779, Fig. 16)^394,432,452–455^ were found to be achievable in a similar way by using the appropriate CuBr_*x*_ salts.

The mixed cluster [μ_4_-O{Cu(L)}_4_Cl_6−*n*_Br_*n*_] (780–807) with *n* = 0–6 are available from using both CuBr_2_ and CuCl_2_ during the formation reaction, or by ligand exchange from [μ_4_-O{Cu(MeOH)}_4_Cl_6−*n*_Br_*n*_] (780–786).^456,457^

**Table 16:** Adamantane-type clusters with a central μ_4_-oxygen atom, Cu in the Q position and group 17 atoms in the E position^a^

Compound	Reagents/conditions	Method
[μ_4_-O{Cu(Py)}_4_Cl_6_] (698)	CuCl_2_, NaOH/Py, 2 days	C^385^
[μ_4_-O{Cu(2-methylpyridine)}_4_Cl_6_] (699)	CuCl_2_, 2-methylpyridine/MeOH, 65 °C, 24 h	C^387^
[μ_4_-O{Cu(OPPh_3_)}_4_Cl_6_] (700)	CuCl_2_, CuO, OPPh_3_/MeNO_2_, 100 °C, 3 h	C^386^
[μ_4_-O{Cu(3-quinuclidinone)}_4_Cl_6_] (701)	CuCl_2_·2H_2_O, 3-quinuclidinone, MeONa/MeOH, 65 °C, 15 min	C^388^
[μ_4_-O{Cu(HMTA)}_4_Cl_6_] (702)	CuCl_2_·H_2_O, HMTA/Me_2_CO	C^389^
[μ_4_-O{Cu(OSR_2_)}_4_Cl_6_] (703–704, R = Et, ^*n*^Pr)	CuCl_2_·2H_2_O, OSR_2_/Me_2_CO, 24 h	C^390^
[μ_4_-O{Cu(*N*-Methylimidazole)}_4_Cl_6_] (705)	CuCl_2_·2H_2_O, *N*-methylimidazole/MeOH	C^391,392^
[μ_4_-O{Cu(dmso)}_4_Cl_6_] (706)	Cu/CCl_4_, DMSO	C^393,394^
[μ_4_-O{Cu(MeCN)}_4_Cl_6_] (707)	CuCl_2_·2H_2_O, HBDA/MeCN, 82 °C	C^394,395^
[μ_4_-O{Cu(1,2-dimethylimidazole)}_4_Cl_6_] (708)	CuCl_2_·2H_2_O, 1,2-dimethylimidazole/EtOH, MeOH	C^396^
[μ_4_-O{Cu(nictonie)}_4_Cl_6_] (709)	CuCl_2_·2H_2_O, nicotine/H_2_O, Me_2_CO	C^397^
[μ_4_-O{Cu(3,4-dimethyl-5-phenylpyrazole)}_4_Cl_6_] (710)	CuCl_2_·2H_2_O, 3,4-dimethyl-5-phenylpyrazole/EtOH	C^398^
[μ_4_-O{Cu(*N*,*N*-dimethylaminomethylferrocene)}_4_Cl_6_] (711)	CuCl, *N*,*N*-dimethylaminomethylferrocene, O_2_/CH_2_Cl_2_, 20 min	C^399^
[μ_4_-O{Cu(7-azaindol)}_4_Cl_6_] (712)	CuCl_2_·2H_2_O, 7-azaindol/MeOH, 65 °C, 15 min	C^400^
[μ_4_-O{Cu(Me_2_NH)}_4_Cl_6_] (713)	Cu/Me_2_NH·HCl, DMF, 50 °C, 30 min	C^401^
[μ_4_-O{Cu(cpz)}_4_Cl_6_] (714)	CuCl_2_·2H_2_O, cpz/EtOH	C^402^
[μ_4_-O{Cu(1-(4-picolylpyrrolidin-2-on)}_4_Cl_6_] (715)	CuCl_2_·2H_2_O, 1-(4-picolyl)pyrrolidin-2-on/MeOH	C^403^
[μ_4_-O{Cu(morpholine)}_4_Cl_6_] (716)	CuCl, morpholine, Cl_3_CCOOMe/MeCN, H_2_O, 30 min	C^404,405^
[μ_4_-O{Cu(Ph_2_SNH)}_4_Cl_6_] (717)	CuCl_2_·2H_2_O, Ph_2_SNH, air/MeCN, 1 day	C^406^
[μ_4_-O{Cu(Imidazole)}_4_Cl_6_] (718)	CuCl_2_·2H_2_O, imidazole/MeOH	C^407^
[μ_4_-O{Cu(thf)}_4_Cl_6_] (729)	CuCl_2_·2H_2_O/THF	C^408^
[μ_4_-O{Cu(2-methyl-2-thiazoline)}_4_Cl_6_] (720)	CuCl_2_·2H_2_O, 2-methyl-2-thiazoline/MeOH	C^409^
[μ_4_-O{Cu(2-ethylpyrazine)}_4_Cl_6_] (721)	CuCl, 2-ethylpyrazine, air/MeCN, 2 days	C^410^
[μ_4_-O(Cu{1-(1-Isoquinolyl)benzotriazole})_4_Cl_6_] (722)	CuCl_2_·2H_2_O, 1-(1-isoquinolyl)benzotriazole/MeOH, CHCl_3_, 1 day	C^411^
[μ_4_-O{Cu(3-mesitylpyrazole)}_4_Cl_6_] (723)	CuCl_2_·2H_2_O, 3-mesitylpyrazole, NaOH/MeOH, 18 h	C^412^
[μ_4_-O{Cu(3-benzyl-benzimidazole)}_4_Cl_6_] (724)	CuSO_4_·5H_2_O, benzimidazole, benzlychloride/Py, 120 °C, 36 h	B^413^
[μ_4_-O{Cu(2-ethyltetrazole)}_4_Cl_6_] (725)	CuCl_2_·2H_2_O, 2-ethyltetrazole/MeOH, 1 h	C^414^
[μ_4_-O{Cu(1-Methylbenzotriazole)}_4_Cl_6_] (726)	CuCl_2_·2H_2_O, 1-methylbenzotriazole, CuO/MeOH, 65 °C, 1 h	C^415^
[μ_4_-O{Cu(pyridine *N*-oxide)}_4_Cl_6_] (727)	CuCl_2_·2H_2_O, pyridine N-oxide/MeOH, 45 min	C^416^
[μ_4_-O{Cu(2-Methylimidazole)}_4_Cl_6_] (728)	CuCl_2_·2H_2_O, 2-methylimidazole/MeOH, 45 min	C^416^
[μ_4_-O(Cu{OP(NH^*t*^Bu)_3_})_4_Cl_6_] (729)	CuCl_2_·2H_2_O, OP(NH^*t*^Bu)_3_/hexane, 80 °C, 38 h	C^417^
[μ_4_-O{Cu(3,5-dimethylpyrazole)}_4_Cl_6_] (730)	CuCl_2_·2H_2_O, acetylacetone, benzohydrazide/EtOH, 8 h	C^418,419^
[μ_4_-O{Cu(1,4-dioxane)}_4_Cl_6_] (731)	CuCl_2_·2H_2_O, 1,4-dioxane, benzoylhydrazine/MeOH, CH_2_Cl_2_, 30 min	C^420^
[μ_4_-O{Cu(1-ethyl-5-nitro-1,2,3-triazole)}_4_Cl_6_] (732)	CuCl_2_·2H_2_O, 1-ethyl-5-nitro-1,2,3-triazole/EtOH, 78 °C, 1 h	C^421^
[μ_4_-O{Cu(3-hydroxyethylpyridine)}_4_Cl_6_] (733)	CuCl_2_·2H_2_O, 3-hydroxyethylpyridine/MeOH	C^422^
[μ_4_-O{Cu(Quinuclidine)}_4_Cl_6_] (734)	CuCl, quinuclidine, air/MeCN, 82 °C, 30 min	C^423^
[μ_4_-O(Cu{1-(pyridin-2-ylmethyl)-1H-benzimidazole})_4_Cl_6_] (735)	CuCl_2_·6H_2_O, 1-(pyridin-2-ylmethyl)-1H-benzimidazole, air/MeCN, H_2_O	C^424^
[μ_4_-O{Cu(benzylamine)}_4_Cl_6_][Cu(benzylamine)_2_Cl_2_] (736)	CuCl_2_·2H_2_O, benzylamine/MeOH, 10 min	C^394^
[μ_4_-O{Cu(MeOH)}_4_Cl_6_] (737)	CuCl_2_·2H_2_O, CuO/MeOH, 65 °C, 2 h	C^394^
[μ_4_-O{Cu(Pz^iPr2^H)}_4_Cl_6_] (738)	CuCl_2_·2H_2_O, Pz^iPr2^H, sodium parafluorobenzoate/MeOH, 4 h	C^425^
[μ_4_-O{Cu(DASO)}_4_Cl_6_] (739)	CuCl, DASO, air/allyl chloride, 3 h	C^426^
[μ_4_-O{Cu(4-dimethylaminopyridine)}_4_Cl_6_] (740)	CuCl_2_·2H_2_O, 4-dimethylaminopyridine, 2,2,6,6-tetramethylpiperidinyl-1-oxyl, BnOH/MeOH, CH_2_Cl_2_, 10 min	C^427^
[μ_4_-O{Cu(phenethylamine)}_4_Cl_6_]·[Cu(phenethylamine)_2_Cl_2_] (741)	CuCl_2_·2H_2_O, phenethylamine/MeOH, 10 min	C^428^
[μ_4_-O{Cu(*N*,*N*-dimethylbenzylamine)}_4_Cl_6_] (742)	CuCl_2_·2H_2_O, *N*,*N*-dimethylbenzylamine/MeOH, 10 min	C^428^
[μ_4_-O{Cu(cyclohexanemethylamine)}_4_Cl_6_]·1,5[Cu(cyclohexanemethylamine)_2_Cl_2_] (743)	CuCl_2_·2H_2_O, cyclohexanemethylamine/MeOH, 10 min	C^428^
[μ_4_-O{Cu(pyrazole)}_4_Cl_6_] (744)	CuCl_2_·2H_2_O, pyrazole/MeOH, 65 °C, 2 h	C^455^
[μ_4_-O{Cu(dimethyl acetamide)}_4_Cl_6_] (745)	CuCl_2_·2H_2_O, dimethyl acetamide/1,4-dioxane, 50 °C, 24 h	C^429^
[μ_4_-O{Cu(1-vinylimidazole)}_4_Cl_6_] (746)	CuCl_2_·2H_2_O, 1-vinylimidazole/MeOH, H_2_O, 60 °C, 2 days	C^430^
[μ_4_-O{Cu(metronidazole)}_4_Cl_6_] (747)	CuCl, metronidazole, air/MeOH	C^431^
[μ_4_-O{Cu(NCNMe_2_)}_4_Cl_6_] (748)	CuCl_2_·2H_2_O/NCNMe_2_	C^432^
[μ_4_-O{Cu(4-(phenylethynyl)pyridine)}_4_Cl_6_] (749)	CuCl, 4-(phenylethynyl)pyridine, air/CH_2_Cl_2_, 24 h	C^433^
[μ_4_-O{Cu(pyridine-3-carbaldehyde)}_4_Cl_6_] (750)	CuCl_2_·2H_2_O, pyridine-3-carbaldehyde/MeOH, CH_2_Cl_2_, 70 °C, 6 days	B^434^
[μ_4_-O{Cu(2-ethylpyridine)}_4_Cl_6_] (751)	CuCl_2_·2H_2_O, 2-ethylpyridine, air/MeOH, 50 °C, 1 h	C^435^
[μ_4_-O(Cu{N-(α-4-picolyl)piperidine})_4_Cl_6_] (752)	CuCl_2_·2H_2_O, *N*-(α-4-picolyl)piperidine/MeCN	C^436^
[μ_4_-O{Cu(OPEt_3_)}_4_Cl_6_] (753)	[PEt_3_CuCl]_4_/CCl_4_, CH_2_Cl_2_, 4 days	C^437^
[μ_4_-O{Cu(DENC)}_4_Cl_6_] (754)	[{(DENC)CuCl}_4_O_2_]/MeOH, CH_2_Cl_2_	J^438^
[μ_4_-O{Cu(benzimidazol)}_4_Cl_6_] (755)	[Cu_2_Cl_3_(benzimidazol)_5_]Cl/EtOH	J^439^
[μ_4_-O{Cu(dmf)}_4_Cl_6_] (756)	[μ_4_-O{Cu(MeOH)}_4_Cl_6_] (737)/DMF	Q^394^
[μ_4_-O{Cu(3-nonyl-8-fluoroimidazo[1,5-*a*]pyridine)}_4_Cl_6_] (757)	[μ_4_-O{Cu(MeCN)}_4_Cl_6_] (707), 3-nonyl-8-fluoroimidazo[1,5-*a*]pyridine/MeCN, 100 °C, 10 min	Q^440^
[μ_4_-O{Cu(Amt)}_4_Cl_6_] (758)	[Cu(α-CgPAmtHMe)(Cl)][BF_4_], KHMDS/THF	J^441^
[μ_4_-O{Cu(nmp)}_3_(CuOH_2_)Cl_6_] (759)	CuCl, O_2_/nmp, H_2_O	F^442^
[μ_4_-O{Cu(Me_2_NH)}_3_{Cu(dmso)}Cl_6_] (760)	Cu/Me_2_NH·HCl, DMSO, 50 °C, 2 h	C^401^
[μ_4_-O{Cu(Me_2_NH)}_2_{Cu(MeOH)}_2_Cl_6_] (761)	Cu/Me_2_NH·HCl, MeOH, 50 °C, 30 min	C^401^
[μ_4_-O{Cu(thf)}_3_(CuOH_2_)Cl_6_]_2_[μ_4_-O{Cu(thf)}_4_Cl_6_]_2_ (762)	CuCl_2_·2H_2_O, tetra-μ-acetato-κ^8^O:O^*t*^-dicopper(ii) dehydrate/THF, 24 h	C^443^
[μ_4_-O{Cu(urea)}_3_{Cu(thf)}Cl_6_] (763)	[μ_4_-O{Cu(MeOH)}_4_Cl_6_] (737), urea/THF, 2 h	Q^444^
[4-phenylimidazolium][μ_4_-O{Cu(4-phenylimidazole)}_3_{CuCl}Cl_6_] (764)	CuCl_2_·2H_2_O, 4-phenylimidazole/MeOH, 45 min	C^416^
[μ_4_-O(Cu{N-(α-4-picolyl)morpholine})_2_(Cu{N-(α-4-picolyl)morpholinium})(CuCl)Cl_6_] (765)	CuCl_2_·2H_2_O, *N*-(α-4-picolyl)morpholine/MeCN, H_2_O	C^436^
[Me_4_N]_4_[μ_4_-O(CuCl)_4_Cl_6_] (766)	CuCl_2_, CuO, [Me_4_N]Cl/MeOH, 65 °C, 24 h	C^445^
[teedH_2_]_2_[μ_4_-O(CuCl)_4_Cl_6_] (767)	CuCl_2_, teed/EtOH	C^446^
[Et_2_NH_2_]_4_[μ_4_-O(CuCl)_4_Cl_6_] (768)	CuCl_2_, [Et_2_NH_2_]Cl/MeOH, 65 °C, 24 h	C^447^
(C_11_H_24_C_12_N_4_O_2_)_2_[μ_4_-O(CuCl)_4_Cl_6_] (769)	CuCl_2_, *N*,*N*′-bis[2-(dimethylamino)-ethyl]propanediamide/CHCl_3_, H_2_O	C^448^
[BMIm]_4_[μ_4_-O(CuCl)_4_Cl_6_] (770)	CuCl_2_·2H_2_O, [BMIm]Cl, O_2_, 2,3,6-trimethylphenol/^*n*^BuOH, 60 °C	C^449^
[H_2_BPBACy]_2_[μ_4_-O(CuCl)_4_Cl_6_] (771)	CuCl_2_·2H_2_O, BPBACy/MeNO_2_, MeOH	C^450^
[choline]_4_[μ_4_-O(CuCl)_4_Cl_6_] (772)	CuCl_2_·2H_2_O, air/choline chloride	C^451^
[μ_4_-O{Cu(Py)}_4_Br_6_] (773)	CuBr_2_, CuO, Py/H_2_O, 100 °C	C^452^
[μ_4_-O{Cu(nicotine)}_4_Br_6_] (774)	CuBr, 4-cyanopyridine, nicotine/DMF, 40 min	C^458^
[μ_4_-O{Cu(2-bromo-1-methyl-imidazole)}_4_Br_6_] (775)	CuBr, 2-mercapto-1-methyl-imidazoline, air/MeCN, CHCl_3_	C^453^
[μ_4_-O{Cu(clotrimazole)}_4_Br_6_] (776)	CuBr_2_, clotrimazole/EtOH, 78 °C, 4 h	C^454^
[μ_4_-O{Cu(benzylamine)}_4_Br_6_][Cu(benzylamine)_2_Br_2_] (777)	CuBr_2_, benzylamine/MeOH, 10 min	C^394^
[μ_4_-O{Cu(3,5-dimethyl-4-bromo-pyrazole)}_4_Br_6_] (778)	CuBr_2_, acetylacetone, benzohydrazide/EtOH, 8 h	C^455^
[μ_4_-O{Cu(NCNMe_2_)}_4_Br_6_] (779)	CuBr_2_/NCNMe_2_	C^432^
[μ_4_-O{Cu(MeOH)}_4_Cl_6−*n*_Br_*n*_] (780–786)	CuCl_2_·2H_2_O, CuBr_2_, CuO/MeOH, 60 °C, 4 h	C^456^
[μ_4_-O{Cu(morpholine)}_4_Cl_6−*n*_Br_*n*_] (787–793, *n* = 0–6)	[μ_4_-O{Cu(MeOH)}_4_Cl_6−*n*_Br_*n*_] (780–786), morpholine/MeOH, 60 °C, 6 h	Q^456^
[μ_4_-O{Cu(piperidine)}_4_Cl_6−*n*_Br_*n*_] (794–800, *n* = 0–6)	[μ_4_-O{Cu(MeOH)}_4_Cl_6−*n*_Br_*n*_] (780–786), piperidine/MeOH, 60 °C, 6 h	Q^456^
[μ_4_-O{Cu(OPPh_3_)}_4_Cl_6−*n*_Br_*n*_] (801–807, *n* = 0–6)	[μ_4_-O{Cu(MeOH)}_4_Cl_6−*n*_Br_*n*_] (780–786), OPPh_3_/MeOH, 60 °C, 6 h	Q^456,457^

a HMTA = hexamethylentetramine, HBDA = hexakis(trimethylsilyl)benzdiamidine, cpz = 2-chloro-10-(3-dimethylaminopropyl(phenothiazine), DENC = *N*,*N*-diethylnicotinamide, Pz^iPr2^H = 3,5-diisopropylpyrazole, DASO = diallyl sulfoxide, Amt = 1,3-diamino-1,2,2-trimethylcyclopentane, CgP = 1,3,5,7-tetramethyl-2,4,6-trioxa-8-phosphatricyclo[3.3.1.1]-decane, nmp = *N*-methyl-2-pyrrolidinone, teed = *N*,*N*,*N*′,*N*′-tetraethylethylenediamine, BPBACy = bis(1-propylbenzimidazol-2-yl)-trans-1,2-cyclohexane.

##### 2.1.6.2 Group 11/17 adamantane-type clusters without central μ_4_-O atom

Unlinke the many oxygen centered chloride adamantane-type structures, there is only one example for an oxygen free species besides a binary copper complex, namely in [H_2_dpipa]_3_[Cu_4_Cl_6_][Cu_2_Cl_6_] (808, dpipa = *N*,*N*′-dimethylpiperazine), obtained from dissolving elemental Cu in HCl together with dpipa and treating it solvothermally in aqueous solution at 120 °C degree for 24 h.^459^

The analogous Br cluster [Cu_4_Br_6_]^2−^ (in 809–816) is found in combination with different ammonium, phosphonium and a Mg complex counterions, always available through a reaction of CuBr with the corresponding complex bromide.^460–464^ One such cluster (806) was also found in a side reaction during the catalytical C–C bond formation between allyl bromide and a (C_6_F_5_)^−^ ligand from a mixed Cu/Al complex.^465^ The congener of the only known Cl species discussed before [H_2_dpipa]_3_[Cu_4_Br_6_][Cu_2_Br_6_] (815) is synthesized in an analogous way by exchanging HCl with HBr.

Even larger complexes can be found in the compound [Ti_12_(μ_3_-O)_14_(O^i^Pr)_18_][Cu_4_Br_6_] (816), in which a polyoxotitanium cluster formed alongside the adamantane when treating CuBr with [Ti(O^i^Pr)_4_] under solvothermal conditions.^466,467^

There is only one example of a compound with a [Cu_4_Br_6_] inorganic core carrying terminal ligands: [{Cu(Hdabco)}_4_Br_6_](HCOO)_2_ (817, dabco = 1,4-diazabicyclo[2.2.2]octane). It is isolated from CuBr and dabco, and contains [{Cu(Hdabco)}_4_Br_6_]^2+^ cations forming loose networks by hydrogen bonding between the cluster units.^468^

**Fig. 17:**
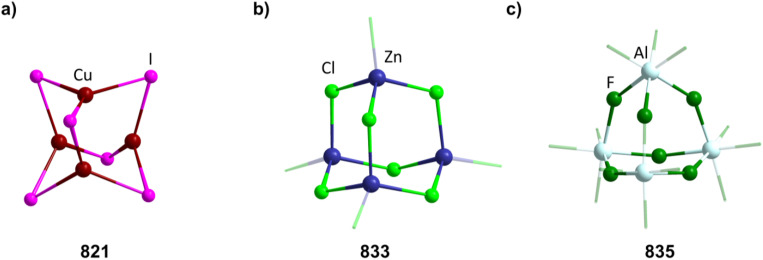
Examples of adamantane-type clusters without a central μ_4_-oxygen atom, group 11–13 elements in the Q position and group 17 atoms in the E position: [(C_7_H_16_)_4_N]_2_[Cu_4_I_6_] (821, left (a)), [{Cp*NbClO}_3_][(Cp*Nb)_3_Cl_2_O_3_OH][(ZnCl)_4_Cl_6_] (833, center (b)) and [H_3_dien]_2_[Al_4_F_18_] (835, right (c)). Counterions are omitted for clarity.

Synthethic strategies for the preparation of [Cu_4_I_6_]^2−^ (in 818–832, Fig. 17) are generally the same as for the bromide compounds. Simple species with ammonium, arsonium or phosphonium are isolated after reactions of CuI, or alternatively Cu and I_2_, with an appropriate complex salt (818–823).^469–473^ Another type of counterion often used are alkaline metal complexes with multidentate ligands such as crown ethers (824–827).^474–476^ They are accessible through iodine salts of Cu and the alkaline metal used, if a polyether of the appropriate size is present. [Cu_4_I_6_]^2−^, similar to its Br congener, is also present as a counterion with other complexes of interest. It is found either as the sole anion or together with [Cu_2_I_4_] in compounds with phosphine Mn complexes, depending on the phophine used (828–829).^477^ Reaction conditions apart from the nature of the ligand stay the same: MnI_2_ and CuI are reacted with R(PPh_2_O)_2_. Similarly, (BPPIP)[{(BPPIP)Cu_2_I_3_}_2_][Cu_4_I_6_] (830, BPPIP = Bis-triphenylphosphonio-isophosphindolide) comprises an additional phosphine coordinated linear Cu_4_I_6_ complex besides the adamantane.^478^ This formation of multiple Cu/I complexes in one compound is also observed for K[K(12-crown-4)]_6_[Cu_4_I_6_][Cu_8_I_13_] (831), prepared according to the strategy described for other ether complex species.^479^ This showcases the importance of the nature of the counterion for the structural motif of the cluster ion.

Lastly, the Cu/I-adamantane motif is observed as a counterion to a three dimensionally extended metal organic framework [Co(tib)_2_][Cu_4_I_6_] (832, tib = 1,3,5-tris(1-imidazolyl)benzene) after a reaction of CoO, CuI and tib according to Method B.^480^

##### 2.1.6.3 Group 11/12 adamantane-type clusters

A very complex compound [{Cp*NbClO}_3_][(Cp*Nb)_3_Cl_2_O_3_OH][(ZnCl)_4_Cl_6_] (833, Fig. 17) featuring two cationic Nb clusters and [(ZnCl)_4_Cl_6_]^2−^ was observed when reducing the bimetallic trigonal bipyramidal complex [(Cp*NbCl_2_)_2_ClO(OH)] with Zn in the presence of ZnO.^481^ It was also found as the counterion in [(Cp*TaCl)_3_O_3_(OH)_2_][(ZnCl)_4_Cl_6_] (834), obtained from a similar reaction of Zn, O_2_ and [(Cp*TaCl_2_)_2_Cl_2_O].^482^

##### 2.1.6.4 Group 11/13 adamantane-type clusters

In group 13, [Al_4_F_18_]^6−^ (in 835–837, Fig. 17) with varying organic countercations are obtained by solvothermal methods using microwave heating from Al(OH)_3_ and HF.^483–485^ In these compounds, each Al site carries three terminal fluorine ligands.

**Table 17:** Adamantane-type clusters without a central μ_4_-oxygen atom, group 11–13 elements in the Q position and group 17 atoms in the E position^a^

Compound	Reagents/conditions	Method
[H_2_dpipa]_3_[Cu_4_Cl_6_][Cu_2_Cl_6_] (808)	Cu, dpipa, HCl/H_2_O, 180 °C, 24 h	B^459^
[^i^Pr_4_N]_2_[Cu_4_Br_6_] (809)	CuBr, [^i^Pr_4_N]Br,/EtOH	C^460,461^
[^*n*^BuNPh_3_]_2_[Cu_4_Br_6_] (810)	CuBr, [^*n*^BuNPh_3_]Br/EtOH	C^462^
[N(PPh_3_)_2_]_2_[Cu_4_Br_6_] (811)	CuBr, [N(PPh_3_)_2_]Br/EtOH, heat	C^463^
[^*t*^Bu_3_NMe]_2_[Cu_4_Br_6_] (812)	CuBr, [^*n*^Bu_3_NMe]Br/^i^PrOH, 100 °C, 30 min	C^461^
[Mg(thf)_6_][Cu_4_Br_6_] (813)	CuBr, MgBr_2_/THF, 50 °C, 18 h	C^464^
[(Poxim)_2_AlBr][Cu_4_Br_6_] (814)	[Al(C_6_F_5_)_3_(toluene)_0.5_], CuO^*t*^Bu, poxim, allyl bromide, C_14_H_30_/toluene, −30 °C to 80 °C, 7 h	U^465^
(H_2_dpipa)_3_[Cu_4_Br_6_][Cu_2_Br_6_] (815)	Cu, HBr, dpipa/180 °C, 24 h	B^459,467^
[Ti_12_(μ_3_-O)_14_(O^i^Pr)_18_][Cu_4_Br_6_] (816)	CuBr, [Ti(O^i^Pr)_4_]/^i^PrOH, 80 °C, 3 days	B^466^
[{Cu(Hdabco)}_4_Br_6_](HCOO)_2_ (817)	CuBr, dabco/DMF, H_2_O, 85 °C, 72 h	C^468^
[MePPh_3_]_2_[Cu_4_I_6_] (818)	CuI, [MePPh_3_]I CuI/MeNO_2_, EtOH, heat	C^469,470^
[MeAsPh_3_]_2_[Cu_4_I_6_] (819)	CuI, [MePPh_3_]I/MeNO_2_, EtOH, heat	C^469^
[Ph_4_P]_2_[Cu_4_I_6_] (820)	Cu, I_2_, [Ph_4_P]I/Me_2_CO, 56 °C	C^471^
[(C_7_H_16_)_4_N]_2_[Cu_4_I_6_] (821)	Cu, I_2_, [(C_7_H_16_)_4_N]/hydroxyacetone, heat	C^472^
[O{P(pyr)_3_}_2_][Cu_4_I_6_] (822)	CuI, KI, ClP(pyr)_3_/MeCN, 90 °C, 1 day	B^473^
[KN{(CH_2_)_2_O(CH_2_)_2_OMe}_3_]_2_[Cu_4_I_6_] (823)	CuI, KI, N{(CH_2_)_2_O(CH_2_)_2_OMe}_3_	C^474^
[Li(benzo-15-crown-5)H_2_O]_2_(benzo-15-crown-5)[Cu_4_I_6_] (824)	CuI, LiI, benzo-15-crown-5, ascorbic acid/H_2_O, Me_2_CO, reflux, 4 h	C^475^
[Cs(benzo-15-crown-5)]_2_[Cu_4_I_6_] (825)	CuI, CsI, benzo-15-crown-5, ascorbic acid/H_2_O, Me_2_CO, reflux, 2 h	C^475^
[Na(18-crown-6)H_2_O]_2_(H_2_O)[Cu_4_I_6_] (826)	CuI, NaI, 18-crown-6, ascorbic acid/H_2_O, Me_2_CO, reflux, 6 h	C^475^
[Rb(18-crown-6)]_2_(MeCN)[Cu_4_I_6_] (827)	RbI, Cu, [NH_4_]I, 18-crown-6/MeCN, 60 °C, 28 h	C^476^
[Mn(tdpmO_3_)_2_][Cu_4_I_6_] (828)	CuI, MnI_2_, tdpmO_3_/MeCN, 30 min	C^477^
[Mn(dppbO_2_)_3_]_2_[Cu_4_I_6_][Cu_2_I_4_] (829)	CuI, MnI_2_, dppbO_2_/MeCN, 1 h	C^477^
(BPPIP)[{(BPPIP)Cu_2_I_3_}_2_][Cu_4_I_6_] (830)	(BPPIP)I, CuI/CH_2_Cl_2_, MeOH	C^478^
K[K(12-crown-4)]_6_[Cu_4_I_6_][Cu_8_I_13_] (831)	CuI, KI, 12-crown-4/H_2_O, Me_2_CO	C^479^
[Co(tib)_2_][Cu_4_I_6_] (832)	CoO, CuI, tib, KI, HI/MeOH, 145 °C, 7 days	B^480^
[{Cp*NbClO}_3_][(Cp*Nb)_3_Cl_2_O_3_OH][(ZnCl)_4_Cl_6_] (833)	[(Cp*NbCl_2_)_2_ClO(OH], Zn, ZnO/CH_2_Cl_2_	U^481^
[(Cp*TaCl)_3_O_3_(OH)_2_][(ZnCl)_4_Cl_6_] (834)	[(Cp*TaCl_2_)_2_Cl_2_O] Zn, O_2_/CH_2_Cl_2_	U^482^
[H_3_*dien*]_2_[Al_4_F_18_] (835)	Al(OH)_3_, dien, HF/EtOH, 190 °C microwave heating, 1 h	B^483^
[H_3_*tren*]_2_[Al_4_F_18_]·3.5H_2_O (836)	Al(OH)_3_, tren, HF/EtOH, 190 °C microwave heating, 1 h	B^484^
(H_3_O)_2_[H*gua*]_16_[Al_4_F_18_]_3_ H_2_O (837)	Al(OH)_3_, HguaCl, HF/EtOH, 190 °C microwave heating, 1 h	B^485^

a dpipa = *N*,*N*′-dimethylpiperazine, PoxIm = *N*-phenyl-*N*′-{bis(tertbutyl)phosphinoxide}-imidazolylidene, dabco = 1,4-diazabicyclo[2.2.2]octane, tib = 1,3,5-tris(1-imidazolyl)benzene, pyr = pyrrolidine, dppbO_2_ = 1,2-bis(diephenlyphospineoxide) benzol, tdpmO_3_ = tris(diephenlyphospineoxide) methan, BPPIP = bis-triphenylphosphonio-isophosphindolide, THP = tetrahydropyran, tren = tris(2-ethylamino)amine, gua = guanidine.

#### 2.1.7 Q/transition metal adamantane-type clusters

Some uncommon examples of transition metals in the E position can be found in the literature, two of them of clusters with a group 15 element in the Q positon, but mostly with group 16 elements occupying that site. The metals in the E position belong to the transition metals of group 10–12. The structure of the adamantane can vary in its degree of distortion depending on the elements used, as well as the ligands and the possible presence of a central atom. They were often observed by serendipity or as by products for other target compounds. This is reflected in the synthetic methods not following a trend and differing from cluster to cluster.

**Fig. 18:**
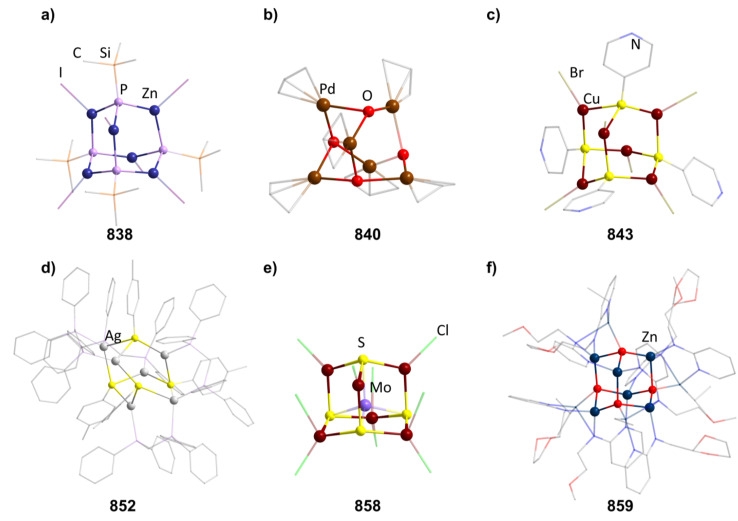
Examples of adamantane-type cluster with transition metal atoms in the E position: [^*n*^Bu_4_N]_2_[(PSiMe_3_)_4_(ZnI)_6_(thf)_2_] (838, top left (a)), [(Pd{(η^3^-C_3_H_5_)}_4_(OH)_6_]CF_3_SO_3_ (840, top center (b)), [(4-SC_5_H_4_NH)_4_(CuBr)_6_] (843, top right (c)), [(SC_6_H_4_Me-p)_4_Ag_6_{(Ph_2_P)_2_Me}_4_][PF_6_]_2_ (852, bottom left (d)), [NMe_4_]_5_[(μ_4_-Mo)S_4_(CuCI)_3_(CuCI_2_)_3_] (858, bottom center(e)) and [O_4_(anpy)_8_Zn_6_(ZnEt)_4_] (859, bottom right (f)). Hydrogen atoms and counterions, if present, are omitted for clarity.

The cyclic Q/Zn complexes [ZnI_2_{Q(SiMe_3_)_3_}]_2_ (Q = P, As) can be prompted to rearrange at elevated temperature when offered a proper cation to form the anionic adamantane-type structures [(QSiMe_3_)_4_(ZnI)_6_(thf)_2_] (838–839, Fig. 18).^486,487^

Hydrolysis of a π-allyl Pd complex with an additional chelating and sterically hindered bispidine ligand leads to the formation of a cationic hydroxo cluster [(Pd{(η^3^-C_3_H_5_)}_4_(OH)_6_]^2+^ (in 840, Fig. 18).^488^

Simple addition reactions can be used to react a preformed Cu complex of a S_2_N_2_-tetradentate ligand with CuCl to form [Cu(bme*daco)}_2_(CuCl)_4_] (841, bme*daco = bis(*N*,*N*′-2-mercapto-2-methylpropyl)1,5-diazocyclooctane).^489^

In a redox reaction of Cu^II^Cl_2_ with KI and *para*-4-mercaptopyridine, a poylmeric Cu^I^_3_I_4_ formed as the main product next to an adamantane-type cluster [(4-SC_5_H_4_NH)_4_(CuCl)_6_] (842) of neutral pyridine-4-thione and Cu^I^Cl.^490^ The corresponding bromide compound (843; Fig. 18) could be obtained after cleaving the S–S bond in 4,4′-bipyridyldisulfide at higher temperatures and reacting with CuBr.^491^

Two isomers of [Cu_6_(phen)_4_(SPh)_4_Cl_2_] (844–845) with differing positions of the chlorine atoms in the cluster scaffold were found after a simple condensation of PhSH with CuO^*t*^Bu under the addition of phenanthroline.^492^ The chlorine found in the compound is suspected to stem from decomposition of the solvent CH_2_Cl_2_.

[NEt_3_]X (X = Cl, Br) was found to break up polymeric [CuSPh]_*n*_ to initiate a rearrangement to [(NEt_4_]_4_[(SPh)_4_(CuX)_6_] (846–847).^170^ This cluster could be prompted to reversibly invert its Q and E positions and form [Et_4_N]_2_[Cu_4_(SPh)_6_] (256) with an excess of [HNEt_3_][SPh], as described before.

Extreme levels of structural distortion are seen in compounds with cationic cluster molecules of the type [(ER)_4_M_6_{(Ph_2_P)_2_R}_4_]^2+^ (848–857, E = S, Se, M = Cu, Ag, Fig. 18), which are made by combination of dimeric complexes [M_2_{(Ph_2_P)_2_R}_2_(MeCN)_2_]^2+^ with phosphine ligand bridged metal centers rearranging around RE^−^ units,^493–495^ or the reaction of polymeric [AgER]_*n*_ with the phosphine ligand.^496^

In one case, an adamantane-type structure could be built around a central [MoS_4_] fragment by coordination of the tetrahedral [NMe_4_]_2_[MoS_4_] with CuCl to isolate crystals of [NMe_4_]_5_[(μ_4_-Mo)S_4_(CuCI)_3_(CuCI_2_)_3_] (858, Fig. 18).^497^

A [Zn_10_O_4_] oxo adamantane is found at the centre of [O_4_(anpy)_8_Zn_6_(ZnEt)_4_] (859, anpy = anilido-pyridinate, Fig. 18). It comprises four terminal ZnEt and six bridging Zn units, which are interconnected by eight bidentate organic ligands. It is obtained from the hydrolysis of ZnEt_2_ in the presence of the templating anilido-pyridinate.^498^

**Table 18:** Adamantane-type cluster with transition metal atoms in the E position^a^

Compound	Reagents/conditions	Method
[^*n*^Bu_4_N]_2_[(PSiMe_3_)_4_(ZnI)_6_(thf)_2_] (838)	[ZnI_2_{P(SiMe_3_)_3_}]_2_, [^*n*^Bu_4_N]_2_I/THF, 24 h	J^486^
[^*n*^Bu_4_P]_2_[(AsSiMe_3_)_4_(ZnI)_6_(thf)_2_] (839)	[ZnI_2_{As(SiMe_3_)_3_}]_2_, [^*n*^Bu_4_P]_2_I/THF, 24 h	J^487^
[(Pd{(η^3^-C_3_H_5_)}_4_(OH)_6_]CF_3_SO_3_ (840)	[(Bdpman)Pd(η^3^-C_3_H_5_)]CF_3_SO_3_/H_2_O, pentane	I^488^
[{Cu(bme*daco)}_2_(CuCl)_4_] (841)	(bme*daco)Cu, CuCl/MeCN	K^489^
[(4-SC_5_H_4_NH)_4_(CuCl)_6_] (842)	CuCl_2_ HS-4-C_5_H_4_N, KI/EtOH, 160 °C, 60 h	B^490^
[(4-SC_5_H_4_NH)_4_(CuBr)_6_] (843)	CuBr, 4,4′-bipyridyldisulfide/EtOH, 120 °C, 3 days	B^491^
[Cu_6_(phen)_4_(SPh)_4_Cl_2_] (844–845)	PhSH, phen, CuO^*t*^Bu/THF, CH_2_Cl_2_, 18 h	C^492^
[(NEt_4_]_4_[(SPh)_4_(CuX)_6_] (846–847, X = Cl, Br)	[CuSPh]_*n*_, [NEt_3_]X/DMF, 10 min	J^170^
[(SePh)_4_Cu_6_{(Ph_2_P)_2_R}_4_][BF_4_]_2_ (848–849, R=CH_2_, NH)	HSePh, NEt_3_ [Cu_2_{(Ph_2_P)_2_R}_2_(MeCN)_2_][BF_4_]_2_/THF, Me_2_CO, 12 h	J^493^
[(ER)_4_Ag_6_{(Ph_2_P)_2_Me}_4_][PF_6_]_2_ (850–853, ER = SPh, SC_6_H_4_Me-p, SePh, SeC_6_H_4_Cl-p)	NaER, [Ag_2_{(Ph_2_P)_2_Me}_2_(MeCN)_2_][PF_6_]_2_/CH_2_Cl_2_, 12 h	J^494^
[(SC_6_H_4_(NH_2_)-m)_4_Ag_6_{(Ph_2_P)_2_NH}_4_][BF_4_]_2_ (854)	NaSC_6_H_4_(NH_2_)-m, [Ag_2_{(Ph_2_P)_2_MNH}_2_][BF_4_]_2_/MeCN, CH_2_Cl_2_ 12 h	J^495^
[(SC_6_H_4_Me-p)_4_Ag_6_{(Ph_2_P)_2_Me}_4_][PF_6_]_2_ (855)	[AgSC_6_H_4_Me-p]_*n*_, dppm, [NH_4_][PF_6_]/CH_2_Cl_2_, 4 h	J^496^
[(SR)_4_Ag_6_{(Ph_2_P)_2_Me}_4_][ClO_4_]_2_ (856–857, R=C_6_H_4_Me-p, 2-Np)	[AgSR]_*n*_, dppm, LiClO_4_/CH_2_Cl_2_, 3 h	J^496^
[NMe_4_]_5_[(μ_4_-Mo)S_4_(CuCI)_3_(CuCI_2_)_3_] (858)	[NMe_4_]_2_[MoS_4_], CuCl/MeCN, 1 h	C^497^
[O_4_(anpy)_8_Zn_6_(ZnEt)_4_] (859)	ZnEt_2_, anpy,/H_2_O	I/K^498^

a bme*daco = bis(*N*,*N*′-2-mercapto-2-methylpropyl)1,5-diazocyclooctane, bdpman = *N*,*N*′-bis(diphenylmethyl)-3,7-diazabicyclo[3.3.1]nonane.

#### 2.1.8 Adamantane-type clusters with mixed elements in Q and E positions

Adamantane-type structures comprising elements from different groups in E and Q positions are rare but have been realized in a variety of examples. Most often, a stepwise buildup approach is used, in which different elements are first linked in small molecules, which can then reassemble into the desired adamantane framework.

**Fig. 19:**
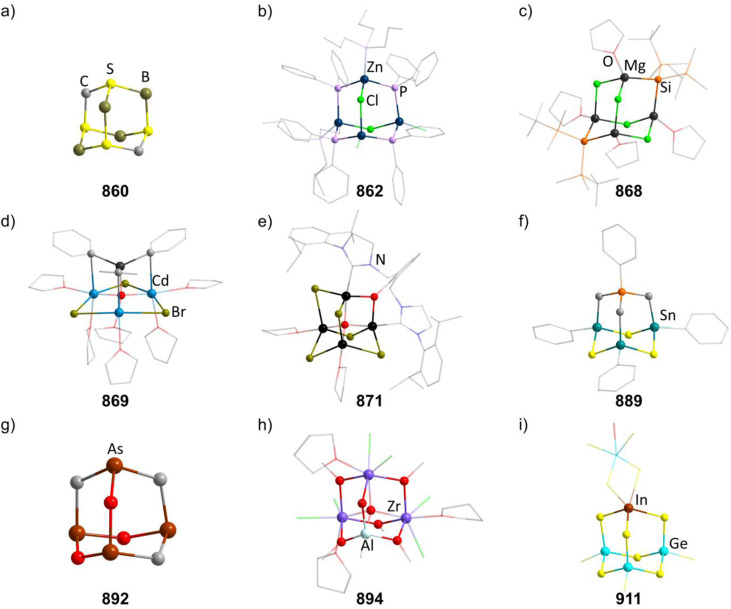
Examples of adamantane-type clusters with elements from different groups in equivalent positions.: [S_4_(CH_2_)_2_(BH_2_)_4_] (860, top left (a)), [(ZnCl)_2_(ZnP^*n*^Pr_3_)_2_(PPh_2_)_4_Cl_2_] (862, top center (b)), [{(thf)Mg}_4_{Si(SiMe_2_^*t*^Bu)_2_}_2_Cl_4_] (868, top right (c)), [μ_4_-O{(thf)_2_Ca}_3_MgPh_3_Br_3_] (869, middle left (d)), [{(1-C{NDippCH_2_CH_2_N})_2_(CH_2_)_2_PhO}Mg_2_(Mg(thf)}_2_Br_4_] (871, middle center (e)), [PhSi{CH_2_Sn(S)Ph}_3_] (889, middle right (f)), [As_4_(CH_2_)_3_O_3_] (892, bottom left (g)), [μ_4_-O(AlMe){(thf)Cl_2_Zr}_3_(OMe)_6_] (894, bottom center (h)) and [H_3_TAEA]_2_[InGe_4_S_11_(SH)_2_(OH)] (911, bottom right (i)). Hydrogen atoms and counterions, if present, are omitted for clarity.

The earliest example of such a reported compound was the cage compound [S_4_(CH_2_)_2_(BH_2_)_4_] (860, Fig. 19), which is obtained by using THF-BH_3_ gas with the binary synthon and solvent CS_2_.^499^ Exchanging the borane for NaB_3_H_8_ leads to a slightly different reactivity, with only one intact CS_2_ unit in the product [S_4_(CH_2_)(BH_2_)_5_] (861).^500^

A mixture of Cl and P in the E position results from a stochiometric condensation reaction between four MCl_2_ (M = Zn, Cd) and four Ph_2_PSiMe_3_ molecules under elimination of ClSiMe_3_ in the presence of P^*n*^Pr_3_ (^*n*^Pr = normal propy). The resulting [(MCl)_2_(MP^*n*^Pr_3_)_2_(PPh_2_)_4_Cl_2_] (862–863, Fig. 19) features two formally retained MCl_2_ fragments bridged by PPh_2_ units.^501^ The Zn compound was also synthesised with varying terminal phosphine ligands (864–866).^502^

A preformed complex dimer [(SiMe_3_)_3_PZnI)I]_2_ was observed to form the adamantane-type [^*n*^Bu_4_N]_2_[(CdI)_4_{P(SiMe_3_)_3_}_2_I_4_] (867) after addition of [^*n*^Bu_4_N]I, which also comprises mixed P and halide E sites, albeit with inverse ratios.^486^ This is formally achieved by a dimerization under elimination of two equivalents of (Me_3_Si)_3_PI.

A similar dimer with a four membered ring-structure [(thf)_2_Mg{Si(SiMe_2_^*t*^Bu)_2_}]_2_ was rearranged under formal chlorination by ^*t*^BuMgCl·2MgCl_2_ to form the adamantane-type dimer [{Mg(thf)}_4_{Si(SiMe_2_^*t*^Bu)_2_}_2_Cl_4_] (868, Fig. 19).^503^

In the preparation of a calcium cuprate, using a CuPh precursor with residual MgBr_2_ from the Grignard reaction carried out in its synthesis can lead to a formal adduct of MgBr_2_ to the Ca complex, leading to [μ_4_-O{(thf)_2_Ca}_3_MgPh_3_Br_3_] (869, Fig. 19) with the central oxygen atom stemming from decomposition of THF.^504^ In this compound, three phenyl groups and three bromides occupy the E positions.

MgBr_2_ can also be used in a reaction with a tridentate carbene-ligand-stabilized adduct of lithium hexamethyldisilazide [{1-C(NDippCH_2_CH_2_N)}_2_(CH_2_)_2_PhOLi_2_N(SiMe_3_)_2_], leading to the substitution of the lithium azide with two MgBr fragments.^505^ As additional products, a symmetric and asymmetric adamantane-type cluster with endohedral μ_4_-O atoms were found. The symmetrical compound, [{(1-C{NDippCH_2_CH_2_N})_2_(CH_2_)_2_PhO}_2_Mg_4_Br_4_] (870), can be understood as a dimer of the carbene stabilized Mg complex, while the asymetrical example, [{(1-C{NDippCH_2_CH_2_N})_2_(CH_2_)_2_PhO}Mg_2_(Mg(thf)}_2_Br_4_] (871, Fig. 19), has lost one ligand and saturates the Mg moieties with THF.

In group 14/16 adamantane clusters, the group 16 elements in E position can be replaced by isoelectronic CR_2_ fragments. Corresponding compounds can be accessed from carbon-bridged fragments, which are connected by intermolecular or intramolecular condensation reactions with the desired group 16 precursor. For tin compounds, this was first shown for a series 
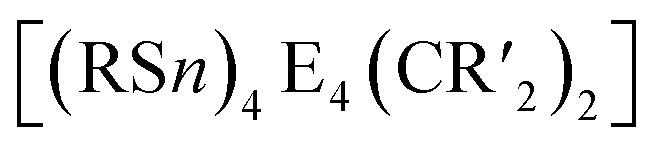
 (872–876, R = Ph, CH_2_SiMe_3_, R′ = H, E = S; R = Ph, R′ = H, E = Se, Te; R = R′ = Me, E = Se), originating from an 
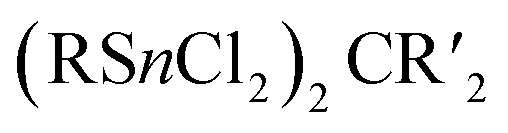
 precursor reacted with Na_2_E or (^*t*^Bu_2_SnE)_2_.^506^ Those compounds were also found to undergo exchange reactions, forming either a cluster with mixed organic ligands, [(PhSn)_2_(Me_3_SiCH_2_Sn)_2_S_4_(CH_2_)_2_] (877), by mixing 872 and 873 or clusters with mixed E sites, [(PhSn)_4_S_4−*x*_Se_*x*_(CH_2_)_2_] (878–881) or [(PhSn)_4_Se_4−*x*_Te_*x*_(CH_2_)_2_] (882–885), by mixing 872 with 874 or 874 with 875, respectively. Note that there are two possible isomers for the *x* = 2 case.

An analogous oxo-cluster, [{(Me_3_Si)_2_CH_2_Sn}_4_O_4_(CMe_2_)_2_] (886), was isolated after exposure of {(Me_3_Si)_2_CH_2_SnCl_2_}_2_CMe_2_ to a NaOH solution.^507^

When using a tetrameric precursor RSi(CH_2_SnPhX_2_)_3_ (X = Cl, I) in reactions with a chalcogenide source, the mixed-element clusters [MeSi{CH_2_Sn(E)Ph}_3_] (887–888, E = S, Se) and [PhSi{CH_2_Sn(E)Ph}_3_] (889–891, E = S, Se, Te; Fig. 19) were realized, with three instead of two E positions being occupied by CH_2_ and also mixed Si and Sn positions.^508,509^

Another reaction to mixed adamantane-type structures from preformed precursors is the synthesis of arsenicin A [As_4_(CH_2_)_3_O_3_] (892, Fig. 19) from the linear CH_2_(AsPhCH_2_AsPh_3_)_2_, which is isolated as a racemic mixture after treatment with HI to halogenate all the As positions and a subsequent hydrolysis with aqueous ammonia.^510^

A hydride cluster [(Cp*Ru)_3_H_5_] can coordinate the primary silane ^*t*^BuSiH_3_ in a μ_3_-η^2^:η^2^:η^2^ mode under H_2_ elimination to form the compound [(Cp*Ru)_3_(^*t*^BuSi)H_6_] (893) with an adamantane-type scaffold.^511^ Hydrogen atoms can be abstracted to transform the multi center bonds into a simpler Ru–Si contact.

Another method to obtain such mixed adamantane-type structures is the substitution of one atom in an already synthesized cluster. One Zr atom in the previously described cluster [μ_4_-O{(thf)Cl_2_Zr}_4_(OMe)_6_] was treated with AlMe_3_ to incorporate a AlMe site in the Q position of the compound [μ_4_-O(AlMe){(thf)Cl_2_Zr}_4_(OMe)_6_] (894, Fig. 19).^512^

Multiple chalcogenolate clusters comprising transition metals of different groups, and in one study Ga, could be isolated. The earliest study achieves this for [Me_4_N][(MSPh)_*n*_(M′SPh)_4−*n*_(SPh)_6_] (895–899, M/M′ = Fe/Co, Fe/Zn, Fe/Cd, Co/Zn, Co/Cd) by exchange between the homometallic clusters, as has been described before in this review.^148^ Similar compounds [Me_3_NBn]_2_[(FeCl)_3_Cu(E^i^Pr)_6_] (900–901, E = S, Se) and [^*n*^Pr_3_N(CH_2_)_6_N^*n*^Pr_3_][(FeBr)_3_Cu(SePh)_6_] (902) with Fe and Cu in the Q positions could also be obtained from a dimeric homometallic precursor complex [Me_3_NBn]_2_[(Fe_2_(E^i^Pr)_6_] by addition of FeCl_2_ and CuCl or from a mixture of CuBr, Fe(OAc)_2_ and PhSeSiMe_3_, and under addition of [^*n*^Pr_3_N(CH_2_)_6_N^*n*^Pr_3_] counterions in the second case.^513–515^

As discussed for the Cu/Te cluster 254 before, there are examples for μ_3_-group 11 atoms located at the center of a M_3_Te_3_ six membered ring. In the following compounds, the Q position opposing this μ_3_ metal is occupied by an element from a different group. The first examples of this architecture are [(μ_3_-M)(CdPPh_3_)(MPPh_3_)_3_(TePh)_3_(μ_3_-TePh)_3_] (903–904, M = Cu, Ag), prepared from NaTePh, MCl and CdCl_2_ in the presence of PPh_3_.^516^ The Zn congener [(μ_3_-Cu)(ZnP^i^Pr_3_)(CuP^i^Pr_3_)_3_(TePh)_3_(μ_3_-TePh)_3_] (905) was later isolated by a more complex synthetic route starting from a tetranuclear cluster precursor [(P^i^Pr_3_)_3_(CuTePh)_4_] which was reacted in a stepwise manner with ZnEt_2_, P^i^Pr_3_ and PhTeSiMe_3_.^517^ The same motif could also be stabilized for compounds with the main group metal Ga, [μ_3_-Cu{Cu(PR_3_)_3_}_3_(GaMe)(EPh)_6_] (906–909, E = Se, R = Me, Et, Et_2_^i^Pr; E = Te, R = Et).^174^ They are obtained after reacting the complexes [(PR_3_)_5_(CuEPh)_6_] with chalcogenidolates and a GaMe source.

A mixed W/Ti oxygen adamantane-type structure [(W(O)O^i^Pr)_2_{Ti(O^i^Pr)_2_}_2_(O)_4_(bdmap)_2_] (910, Hbdmap = 1,3-bis-(dimethylamino)-propan-2-ol) was obtained after a reaction of the complex [W(O)(O^i^Pr)_3_(bdmap)] with Hbdmap and Ti(O^i^Pr)_4_, followed by a hydrolysis in a H_2_O/^i^PrOH mixture.^122^

There are also examples of mixed adamantanes accessible directly from the elements and simple binary compounds if the correct additives and conditions are used. [H_3_TAEA]_2_[InGe_4_S_11_(SH)_2_(OH)] (911, TAEA = tris(2-aminoethyl)amine, Fig. 19) is obtained solvothermally from In(NO_3_) and GeO_2_.^518^ Its structure can be understood as a [Ge_4_S_10_]^4−^ adamantane-type in which one GeS unit is substituted by an InS_2_GeOH(SH)_2_ fragment.

An example of two different transition metals in the E position is obtained when using a Ni complex instead of a copper complex in the reaction to give [Cu(bme*daco)}_2_(CuCl)_4_] (912), leading to the mixed derivative [Ni(bme*daco)}_2_(CuCl)_4_].^489^

**Table 19:** Adamantane-type clusters with elements from different groups in equivalent positions^a^

Compound	Reagents/conditions	Method
[S_4_(CH_2_)_2_(BH_2_)_4_] (860)	THF-BH_3_,/CS_2_, 50 °C, 3 h	G^499^
[S_4_(CH_2_)(BH_2_)_5_] (861)	NaB_3_H_8_/CS_2_, 75 °C, 5 h	B^500^
[(MCl)_2_(MP^*n*^Pr_3_)_2_(PPh_2_)_4_Cl_2_] (862–863, M = Zn, Cd)	MCl_2_, P^*n*^Pr_3_, Ph_2_PSiMe_3_/THF, 12 h	C^501^
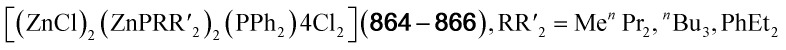	ZnCl_2_, 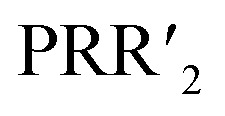 , Ph_2_PSiMe_3_/THF, 12 h	C^502^
[^*n*^Bu_4_N]_2_[(CdI)_4_{P(SiMe_3_)_3_}_2_I_4_] (867)	[CdI_2_{P(SiMe_3_)_3_}]_2_, [^*n*^Bu_4_N]_2_I/THF, 24 h	J^486^
[{(thf)Mg}_4_{Si(SiMe_2_^*t*^Bu)_2_}_2_Cl_4_] (868)	[(thf)_2_Mg{Si(SiMe_3_^*t*^Bu)_2_}]_2_, ^*t*^BuMgCl·2 MgCl_2_/THF, C_6_H_6_, 0 °C	J^503^
[μ_4_-O{(thf)_2_Ca}_3_MgPh_3_Br_3_] (869)	MgBr_2_, CuPh, Ca/THF, −78 °C to RT, 20 h	C^504^
[{(1-C{NDippCH_2_CH_2_N})_2_(CH_2_)_2_PhO}_2_Mg_4_Br_4_] (870)	[{1-C(NDippCH_2_CH_2_N)}_2_(CH_2_)_2_PhOLi_2_N(SiMe_3_)_2_], MgBr_2_/THF, 12 h	K^505^
[{(1-C{NDippCH_2_CH_2_N})_2_(CH_2_)_2_PhO}Mg_2_(Mg(thf)}_2_Br_4_] (871)	[{1-C(NDippCH_2_CH_2_N)}_2_(CH_2_)_2_PhOLi_2_N(SiMe_3_)_2_], MgBr_2_/THF, 12 h	K^505^
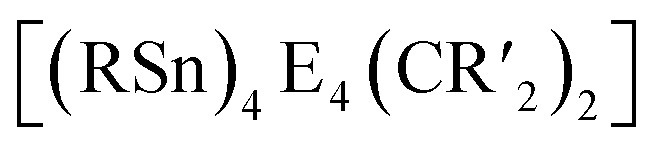 (872–876, R = Ph, CH_2_SiMe_3_, R′ = H, E = S; R = ph, R′=H, E = Se, Te; R = R′ = Me, E = Se)	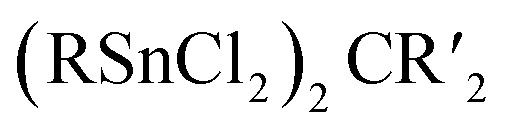 , Na_2_E/Me_2_CO, H_2_O, 0 °C to RT, 18 h	K^506^
[(PhSn)_2_(Me_3_SiCH_2_Sn)_2_S_4_(CH_2_)_2_] (877)	[(PhSn)_4_S_4_(CH_2_)_2_] (872), [(Me_3_SiCH_2_Sn)_4_S_4_(CH_2_)_2_] (873)/CH_2_Cl_2_, 2 days	R^506^
[(PhSn)_4_S_4−*x*_Se_*x*_(CH_2_)_2_] (878–881)	[(PhSn)_4_S_4_(CH_2_)_2_] (872), [(PhSn)_4_Se_4_(CH_2_)_2_] (874)/CH_2_Cl_2_	R^506^
[(PhSn)_4_Se_4−*x*_Te_*x*_(CH_2_)_2_] (882–885)	[(PhSn)_4_Se_4_(CH_2_)_2_] (874), [(PhSn)_4_Te_4_(CH_2_)_2_] (875)/CH_2_Cl_2_	R^506^
[{(Me_3_Si)_2_CH_2_Sn}_4_O_4_(CMe_2_)_2_] (886)	{(Me_3_Si)_2_CH_2_SnCl_2_}_2_CMe_2_, NaOH/H_2_O, PhMe, 80 °C, 18 h	K^507^
[MeSi{CH_2_Sn(S)Ph}_3_] (887–888, E = S, Se)	PhSi(CH_2_SnPhI_2_)_3_, Na_2_S/Me_2_CO, MeOH, H_2_O	K^508^
[PhSi{CH_2_Sn(S)Ph}_3_] (889–891, E = S, Se, Te)	PhSi(CH_2_SnPhCl_2_)_3_, (SiMe_3_)_2_E/toluene, 24 h	K^509^
[As_4_(CH_2_)_3_O_3_] (892)	1. CH_2_(AsPhCH_2_AsPh_3_)_2_, HI/CH_2_Cl_2_	I^510^
	2. NH_3_, H_2_O/THF	
[(Cp*Ru)_3_(^*t*^BuSi)H_6_] (893)	[(Cp*Ru)_3_H_5_], ^*t*^BuSiH_3_/hexane, 5 min	C^511^
[μ_4_-O(AlMe){(thf)Cl_2_Zr}_3_(OMe)_6_] (894)	[μ_4_-O{(thf)Cl_2_Zr}_4_(OMe)_6_], AlMe_3_/THF, PhMe, 12 h	R^512^
[Me_4_N][(MSPh)_*n*_(M′SPh)_4−*n*_(SPh)_6_] (895–899, M/M′ = Fe/Co, Fe/Zn, Fe/Cd, Co/Zn, Co/Cd)	[Me_4_N][(MSPh)_4_(SPh)_6_], [Me_4_N][(M′SPh)_4_ (SPh)_6_]/MeCN	C^148^
[Me_3_NBn]_2_[(FeCl)_3_Cu(S^i^Pr)_6_] (900)	[Me_3_NBn]_2_[(Fe_2_(S^i^Pr)_6_], FeCl_2_, CuCl/THF, 2 days, 70 °C	K^513^
[Me_3_NBn]_2_[(FeCl)_3_Cu(Se^i^Pr)_6_] (901)	[Me_3_NBn]_2_[(Fe_2_(Se^i^Pr)_6_], FeCl_2_, CuCl/MeCN, 1 day, 70 °C	J^514^
[^*n*^Pr_3_N(CH_2_)_6_N^*n*^Pr_3_][(FeBr)_3_Cu(SePh)_6_] (902)	CuBr, Fe(OAc)_2_, PhSeSiMe_3_, [^*n*^Pr_3_N(CH_2_)_6_N^*n*^Pr_3_]Br_2_/MeCN	C^515^
[(μ_3_-Cu)(CdPPh_3_)(CuPPh_3_)_3_(TePh)_3_(μ_3_-TePh)_3_] (903)	NaTePh, CuCl, CdCl_2_, PPh_3_/THF, 2 h	C^516^
[(μ_3_-Ag)(CdPPh_3_)(AgPPh_3_)_3_(TePh)_3_(μ_3_-TePh)_3_] (904)	NaTePh, AgCl, CdCl_2_, PPh_3_/THF, 3 h	C^516^
[(μ_3_-Cu)(ZnP^i^Pr_3_)(CuP^i^Pr_3_)_3_(TePh)_3_(μ_3_-TePh)_3_] (905)	[(P^i^Pr_3_)_3_(CuTePh)_4_], ZnEt_2_, P^i^Pr_3_, PhTeSiMe_3_/^*n*^Hep, EtOH, 2 h, 0 °C to RT	C^517^
[(μ_3_-Cu){Cu(PR_3_)_3_}_3_(GaMe)(SePh)_3_(μ_3_-SePh)_3_] ((906–907, R = Me, Et_2_^i^Pr)	1. CuOAc, PR_3_, Me_3_SiSePh/THF	J^174^
	2. Me_3_SiSePh, [Me_2_GaSePh]_*n*_/THF	
[(μ_3_-Cu){Cu(PEt_3_)_3_}_3_(GaMe)(SePh)_3_(μ_3_-SePh)_3_] (908)	[(PEt_3_)_5_(CuSePh)_6_], Me_3_SiSePh, [Me_2_GaSePh]_*n*_/THF	J^174^
[(μ_3_-Cu){Cu(PEt_3_)_3_}_3_(GaMe)(TePh)_3_(μ_3_-TePh)_3_] (909)	[(PEt_3_)_5_(CuTePh)_6_], Me_3_SiTePh, Me_3_Ga·OEt_2_/THF	J^174^
[(W(O)O^i^Pr)_2_{Ti(O^i^Pr)_2_}_2_(O)_4_(bdmap)_2_] (910)	[W(O)(O^i^Pr)_3_(bdmap)], Hbdmap, Ti(O^i^Pr)_4_/PhMe, H_2_O, HO^i^Pr, 2 days, 110 °C to 0 °C	C/I^122^
[H_3_TAEA]_2_[InGe_4_S_11_(SH)_2_(OH)] (911)	In(NO_3_), GeO_2_, TAEA/(CH_2_OH)_2_, ^*n*^BuNH_2_, (CH_2_SH)_2_, 170 °C, 5 days	B^518^
[{Ni(bme*daco)}_2_(CuCl)_4_] (912)	(bme*daco)Ni, CuCl/MeCN	K^489^

a Hbdmap = 1,3-bis-(dimethylamino)-propan-2-ol, ^*n*^Hep = normal heptane, TAEA = tris(2-aminoethyl)amine.

#### 2.1.30 Compounds with extended adamantane-type structure

By formal addition of metal atoms on the outside of an adamantane-type cage, as opposed to endohedral addition as in case of some previously discussed compounds, larger clusters could be obtained while still maintaining an adamantane core structure. As the addition of many atoms leads to totally new structural motifs, only some examples with the addition of only a few atoms and a clear adamantane core will be discussed.

A formal addition of a Cu(PR_3_)_2_ unit to a [μ_3_-Cu(CuPR_3_)_3_Cu(EPh)_6_] (E = Se, Te) core, a structural motif observed in the previously discussed compound 261,^175^ leads to the neutral clusters [μ_3_-Cu(CuPR_3_)_3_{Cu(PR_3_)_2_}Cu(EPh)_6_] (913–915).^519,520^ The synthesis does not deviate much from the one for the anionic cluster. As in all cases, a Cu salt is reacted with PR_3_ and PhESiMe_3_, with the resulting compound depending only on the exact chalcogenide or PR_3_ used.

**Fig. 20:**
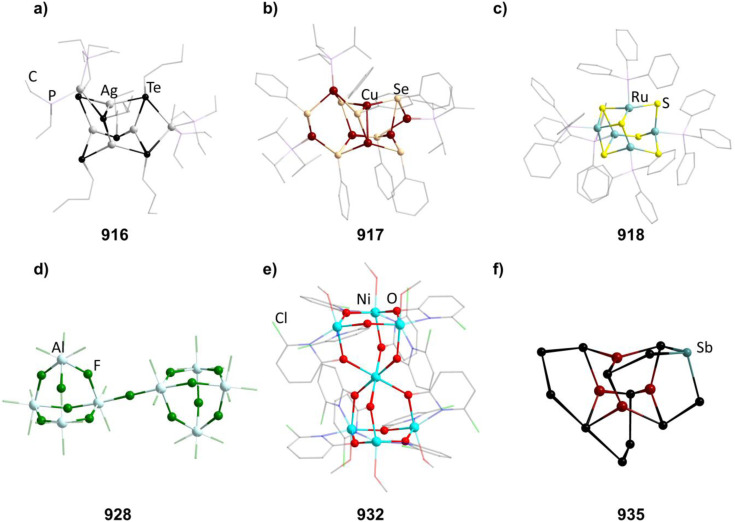
Examples of compounds with an extended adamantane-type structure: [Ag_4_{Ag(PEt_3_)_2_}_2_(Te^*n*^Bu)_6_] (916, top left (a)), [Cu_4_(CuP^i^Pr_3_)_3_(SePh)_7_] (917, top center (b)), [μ_3_-(RuPPh_3_)(RuPPh_3_)_4_S_6_] (918, top right (c)), [H_3_tren]_4_[(Al_4_F_17_)_2_F]OH (928, bottom left (d)), [Ni{Ni(chp)_2_MeOH}_6_]Cl_2_ (932, bottom center (e)) and [Et_4_N]_3_[Cu_4_Sb(Te_7_)(Te_2_)_2_Te] (935, bottom right (f)). Hydrogen atoms and counterions, if present, are omitted for clarity.

A related compound featuring silver atoms [Ag_4_{Ag(PEt_3_)_2_}_2_(Te^*n*^Bu)_6_] (916, Fig. 20) could be isolated using an analogous route.^521^ Here, both additional Ag(PEt_3_)_2_ units coordinate on the outside of the cluster, bridging two Te atoms each.

[Cu_4_(CuP^i^Pr_3_)_3_(SePh)_7_] (917, Fig. 20) is an example of a larger expansion of a [Cu_4_(SePh)_6_] central adamantane. In this case, by addition of a μ-CuP^i^Pr_3_ connecting two selenium atoms of the central scaffold and an additional μ_3_-(CuP^i^Pr_3_)_2_SePh bridge between three other selenolates, an increase of the coordination number of all but one selenium atoms to 4 is achieved.^520^

Another structural motif of expanded adamantanes is achieved by formally capping one face of the octahedron formed by the six E atoms with an additional metal fragment. In contrast to some other molecules, we have discussed featuring μ_3_ Cu or Ag atoms in the center of a six membered ring of the adamantane-type scaffold, these metal moieties carry additional ligands and are located below the plane of the Q_3_E_3_-ring, which causes a greater deviation from planarity as opposed to a more planar arrangement when compared to an uncoordinated adamantane-type structure. This effect can be observed in [μ_3_-(RuPPh_3_)(RuPPh_3_)_4_S_6_] (918, Fig. 20) when compared to the non-coordinated 235 discussed beforehand.^152^ The extended compound is obtained by reacting S(SiMe_3_)_2_ with [Ru(PPh_3_)_3_Cl_2_] in hot THF, as opposed to using NaSH as a sulfur precursor, which leads to less oxidized metal centers.

This architecture has also been explored for two clusters [μ_3_-(FeCl)(VPEt_3_)(FePEt_3_)_3_S_6_] (919–920, M = V, Mo) capped by a FeCl unit.^522,523^ They are also obtained by using S(SiMe_3_)_2_ and a mixture of the metal complexes [M(thf)_3_Cl_3_] and [Fe(PEt_3_)_2_Cl_2_]. Sodium thiolates can be used to replace the chlorine atom at the added site by a SR group (921–925).

When NaS_2_ is used instead, the cluster will dimerize to [μ_3_-{(VPEt_3_)(FePEt_3_)_3_FeS_6_}_2_S] (926), comprising two adamantane-type cages connected by a μ-S bridge.^524^ A more distorted example of this dimer buildup is [μ_3_-(HgSPh)(AgPPh_3_)_3_Hg(SPh)_6_}_2_S] (927), in which the metal atoms on both sites of an Ag_3_S_3_ ring are Hg atoms.^525^ This is achieved by forming NaSPh *in situ* and reacting it with HgO and [Ag(PPh_3_)_2_]NO_3_.

This μ-bridged adamantane topology is also present in two further examples. One is [H_3_tren]_4_[(Al_4_F_17_)_2_F]OH (928, tren = tris(2-ethylamino)amine, Fig. 20), in which two [Al_4_F_18_] clusters are condensed by a μ-F.^484^ It is observed when adjusting the compound ratios in the synthesis of monomeric 836 (see section 2.1.6). The other is [{P_4_(NMe)_6_}_2_CuCl]_2_ (929) which was isolated as a side product when reacting an excess of [P_4_(NMe)_6_] with CuCl besides further polymeric products, that will be discussed in the next section.^526^

A different type of dimer could first be observed in the compounds [H_2_Ta(tdci)_2_]CI_3_ (tdci = 1,3,5-trideoxy-1,2,5-tris(dimethylamino)-*cis*-inositol, Hchp = 6-chloro-2-hydroxypyridine, 930) and [H_11_Ta_7_O_12_(tdci)_6_] (931), in which two adamantane-type clusters are condensed by one atom in the Q position.^527^ The first is an organometallic compound, in which a central Ta is trigonal prismatically coordinated by six oxygen atoms and the adamantane-type scaffolds are completed by hydrocarbons. This is achieved by coordinating tdci to TaCl_5_ in methanol. The second compound is obtained after hydrolysis of the first, and features two condensed Ta_4_O_6_ subunits decorated by tdci ligands on the three non-condensed Ta sites, which in turn resemble an organometallic adamantane-type structure. Thus, this compound could also be described as comprising 8 condensed adamantane-type scaffolds.

A further compound with the same dimer architechture is [Ni{Ni(chp)_2_MeOH}_6_]Cl_2_ (932, Hchp = 6-chloro-2-hydroxypyridine, Fig. 20), made at 130 °C under inert conditions by addition of Ni(OH)_2_ and Hchp.^528^ This compound is notable due to the fact that there are no monomeric group 10/16 adamantane-type structures at all.

Apart from examples with oxygen, there is an Al/F dimer [(C_2_H_4_NH_3_)_3_NH]_2_·(H_3_O)·[Al_7_F_30_] (933), formally made up of [Al_4_F_18_] clusters condensed by an Al site.^529^ It is obtained by solvothermal conversion from Al_2_O_3_ with HF.

By formally condensing two adamantanes at a face between a Q and two connected E atoms instead of just by one Q atom, a new structural motif is achievable. This was realized for [{(SiMe)_3_(CH2)_4_}_2_Si(CH)_2_] (934), which is formed by two [(SiMe)_4_(CH_2_)_6_] molecules condensed *via* one face.^530,531^ The presence of this compound was confirmed after heating SiMe_4_ at 700 °C.

A clear adamantane-type cluster Cu_4_Te_6_ core is also present in the cluster [Et_4_N]_3_[Cu_4_Sb(Te_7_)(Te_2_)_2_Te] (935, Fig. 20). However, the Te sites are mostly part of oligotellurides.^532^ One Cu atom coordinates to three sites of a linear Te_7_, all of which also coordinate to the three other Cu atoms which form the typical six membered ring opposed to the first copper together with a single Te and two Te_2_ units. Lastly these three Te fragments coordinate a Sb atom below the six membered ring. It was obtained by the extraction of the alloy KCuSbTe_3_, prepared from K_2_Te, Cu, Sb_2_Te_3_ and Te with ethane-1,2-diamine.

**Table 20:** Compounds with an extended adamantane-type structure^a^

Compound	Reagents/conditions	Method
[μ_3_-Cu(CuPEtPh_2_)_3_{Cu(PEtPh_2_)_2_}Cu(TePh)_6_] (913)	CuCl, PEtPh_2_, Te(Ph)SiMe_3_/THF:Et_2_O, RT	C^519^
[μ_3_-Cu(CuPEt_3_)_3_{Cu(PEt_3_)_2_}Cu(SePh)_6_] (914)	CuOAc, Et_3_P, PhSeSiMe_3_/toluene, RT, 12 h	C^520^
[μ_3_-Cu(CuPEt_3_)_3_{Cu(PEt_3_)_2_}Cu(TePh)_6_] (915)	CuOAc, Et_3_P, PhTeSiMe_3_/Et_2_O, 0 °C, 2 h	C^520^
[Ag_4_{Ag(PEt_3_)_2_}_2_(Te^*n*^Bu)_6_] (916)	^*n*^BuTeSiMe_3_, AgCl–PEt_3_/pentane, −40 °C	C^521^
[Cu_4_(CuP^i^Pr_3_)_3_(SePh)_7_] (917)	CuOAc, ^i^Pr_3_P, PhSeSiMe_3_/THF, RT, 1 h	C^520^
[μ_3_-(RuPPh_3_)(RuPPh_3_)_4_S_6_] (918)	[Ru(PPH_3_)_3_Cl_2_], S(SiMe_3_)_2_/MeCN, 85 °C, 6.5 h	C^152^
[μ_3_-(FeCl)(MoPEt_3_)(FePEt_3_)_3_S_6_] (919)	[Mo(thf)_3_Cl_3_], S(SiMe_3_)_2_, [Fe(PEt_3_)_2_Cl_2_]/THF, 50 °C, 4 h	D^522,523^
[μ_3_-(FeCl)(VPEt_3_)(FePEt_3_)_3_S_6_] (920)	[V(thf)_3_Cl_3_], S(SiMe_3_)_2_, [Fe(PEt_3_)_2_Cl_2_]/THF, RT	D^522^
[μ_3_-(FeSPh)(VPEt_3_)(FePEt_3_)_3_S_6_] (921)	[μ_3_-(FeCl)(VPEt_3_)(FePEt_3_)_3_S_6_] (920), NaSPh/THF, MeCN, RT	Q^523^
[μ_3_-(FeSPh)(MoPEt_3_)(FePEt_3_)_3_S_6_] (922)	[μ_3_-(FeCl)(MoPEt_3_)(FePEt_3_)_3_S_6_] (919), NaSPh/THF, MeCN, RT, 30 min	Q^523^
[μ_3_-(FeSEt)(VPEt_3_)(FePEt_3_)_3_S_6_] (923)	[μ_3_-(FeCl)(VPEt_3_)(FePEt_3_)_3_S_6_] (920), NaSEt/THF, MeCN, RT	Q^523^
[μ_3_-(FeSEt)(MoPEt_3_)(FePEt_3_)_3_S_6_] (924)	[μ_3_-(FeCl)(MoPEt_3_)(FePEt_3_)_3_S_6_] (919), NaSEt/THF, MeCN, RT, 30 min	Q^523^
[μ_3_-(FeS-*p*-C_6_H_4_OMe)(MoPEt_3_)(FePEt_3_)_3_S_6_] (925)	[μ_3_-(FeCl)(MoPEt_3_)(FePEt_3_)_3_S_6_] (919), NaS-*p*-C_6_H_4_OMe/THF, MeCN, RT, 30 min	Q^523^
[μ_3_-(VPEt_3_)(FePEt_3_)_3_FeS_6_}_2_S] (926)	[μ_3_-(FeCl)(VPEt_3_)(FePEt_3_)_3_S_6_] (920), Li_2_S/MeCN, RT, overnight	Q^524^
[μ_3_-(HgSPh)(AgPPh_3_)_3_Hg(SPh)_6_}_2_S] (927)	Na, HgO, PhSH, [Ag(PPh_3_)_2_]NO_3_/MeOH, CHCl_3_, 3 h	C^525^
[H_3_*tren*]_4_[(Al_4_F_17_)_2_F]OH (928)	Al(OH)_3_, tren, HF/EtOH, 190 °C microwave heating, 1 h	B^484^
[{P_4_(NMe)_6_}_2_CuCl]_2_ (929)	[P_4_(NMe)_6_], CuCl/MeCN, 2 days	T^526^
[H_2_Ta(tdci)_2_]CI_3_ (930)	TaCl_5_, tdci/MeOH	J^527^
[H_11_Ta_7_O_12_(tdci)_6_] (931)	[H_2_Ta(tdci)_2_]CI_3_/H_2_O	I^527^
[Ni{Ni(chp)_2_MeOH}_6_]Cl_2_ (932)	Ni(OH)_2_, Hchp/130 °C	C^528^
{[C_2_H_4_NH_3_)_3_NH]}_2_·(H_3_O)·[Al_7_F_30_] (933)	Al_2_O_3_, HF, tris(2-aminoethyl)amine/EtOH, 200 °C, 96 h	B^529^
[Si_7_C_16_H_36_] (934)	SiMe_4_/700 °C	A^530,531^
[Et_4_N]_3_[Cu_4_Sb(Te_7_)(Te_2_)_2_Te] (935)	1. K_2_Te, Cu, Sb_2_Te_3_, Te/heat to melt	E^532^
	2. [Et_4_N]Br/en	

a tdci = 1,3,5-trideoxy-1,2,5-tris(dimethylamino)-*cis*-inositol, Hchp = 6-chloro-2-hydroxypyridine.

#### 2.1.31 Adamantane-type scaffolds in polymeric structures

Apart from molecular species, networks consisting of linked adamantanes of the type [Q_4_E_10_] can be formed, either by linking previously existing isolated clusters, or by the synthesis of simpler precursors. To limit the scope, we will only discuss corner condensed species as well as those connected by additional linker molecules. This can lead to zeolite like compounds. Such species have been found for the groups E = 15, 16 and 17.

**Fig. 21:**
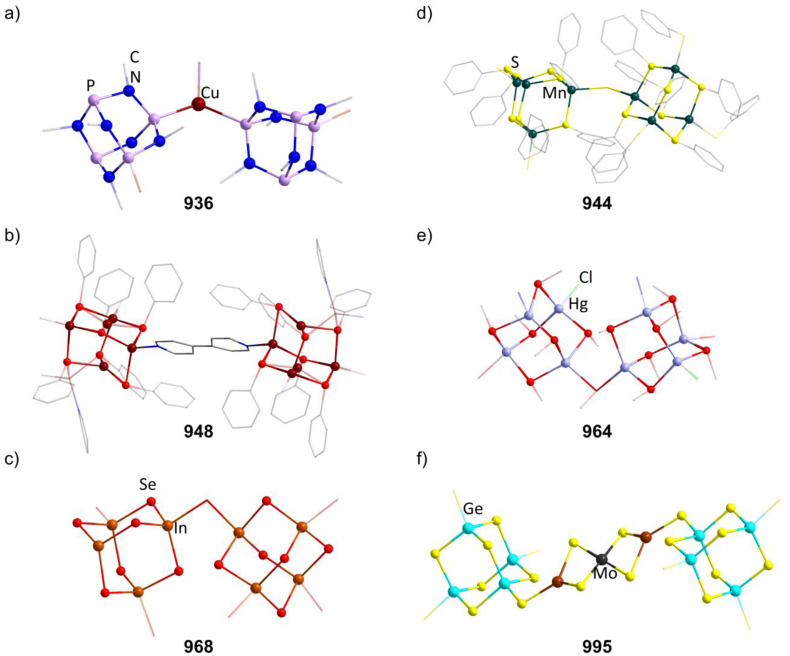
Examples of networks of type 2 supertetrahedra: [{P_4_(NMe)_6_}CuI]_*n*_ (936, top left), [Mn_4_(SPh)_8_]_*n*_ (944, top right (a)), [(μ_3_-Cu)Cu_4_(SePh)_6_(CuPPh_3_)_3_(4,4′-bipy)]_*n*_ (948, middle left (b)), [Hg_4_(PhSe)_7_ClPy]_*n*_ (964, middle right (c)), (C_5_H_5_NH_2_)_24_[In_28_Se_54_(H_2_O)_4_] (968, bottom left (d)) and {[Me_4_N]_4_[(Ge_4_S_10_)Cu_4_Mo_2_S_8_]}_*n*_ (995, bottom right (f)). Hydrogen atoms and counterions, if present, are omitted for clarity.

Linking of the previously discussed P/N adamantane-type structures [P_4_(NR)_6_] (936–938, R = Me, Et, Bn, Fig. 21) can be achieved by the addition CuI to form one dimensional chains of [{P_4_(NR)_6_}CuI]_*n*_ (R = Me, Et) with μ-bridging CuI moieties or [{P_4_(NMe)_6_}(CuI)_2_(MeCN)_2_]_*n*_ comprising linking CuI and MeCN four membered rings.^81,533^ Similarly, reactions of [P_4_(NMe)_6_] with CuCl lead to a three dimensional network [{P_4_(NMe)_6_}_2_(CuCl)_3_(MeCN)_2_] (939) or a ladder like one dimensional polymer [{P_4_(NMe)_6_}(CuCl)_2_]_*n*_ (940) depending on the cluster to CuCl ratio.^526^ Ligands on [(PNSiMe_3_)_4_(NMe)_6_] can be exchanged for TiCl_3_ or *p*-^*n*^BuPhPCl_2_, which polymerize to form extended networks that could not yet be structurally characterized (941–942).^95^

A previously discussed Cr/O adamantane-type cluster with hpdta ligands can be obtained as Ba linked chains in [Ba(OH_2_)_5_{Cr_4_(OH)_4_(hpdta)_2_}]_*n*_ (943) by adding BaCl_2_ to the initial reaction mixture without ethane-1,2-diamine.^119^ This leads to two parallel cluster strands connected *via* interactions between Ba ions and the organic ligand.

The only known group 7 example is the thiolate network [Mn_4_(SPh)_8_]_*n*_ (944, Fig. 21), in which all metal centers coordinate to the next cluster *via* a bridging thiolate, a composition often observed for transition metal chalcogenolates.^534^ It is isolated after a reaction of [Mn{N(SiMe_3_)_2_}_2_]_2_ with HSPh in THF at low temperatures.

A layered network of [{Cu_4_{SC(NH_2_)_2_}_6_}_2_{SC(NH_2_)_2_}_3_]_*n*_(SO_4_)_4_ (945) can be observed from dissolving Cu_2_SO_4_ in sulfuric acid in the presence of thiourea.^187^ Only three copper atoms per cluster carry a thiourea ligand forming the cluster sheets, while the last one only forms bonds within the adamantane-type scaffold.

Utilizing a multidentate thiolate ligand 4,5-dimercapto-1,3-dithiole-2-thionato (dmit) in a reaction with [Cu(MeCN)_4_][ClO_4_] an ammonium or pyridinium counterion form the dimeric anion in [{Cu_4_(dmit)_3_}_2_]_*n*_^2−^ (in 946–947), which is made up of layers facilitated by further Cu–S and S–S interactions.

The structural motif of a μ_3_-Cu coordinating a six membered ring in an adamantane-type structure has been discussed several times before. Such a motif can also be found in a one dimensional zigzag chain polymer [(μ_3_-Cu)Cu_4_(SePh)_6_(CuPPh_3_)_3_(4,4′-bipy)]_*n*_ (948, Fig. 21), in which such adamantanes are connected by 4,4′-bipy(CuPPh_3_)_2_ units to two Se atoms in the E position and another 4,4′-bipy terminally connected to an adamantane Cu moity.^535^ It forms from Cu(MeCOO), PhSeSiMe_3_, PPh_3_ and 4,4′-bipy.

Another dmit linked layered cluster exists in {[Et_4_N][(Ag_4_(dmit)_3_]dmf}_*n*_ (949), in which a porous architecture filled with both cations and solvents is formed.

Linear chains of [Zn_4_(SPh)_6_] adamantane-type clusters are found in a series of compounds [(Zn_4_(SPh)_8_ROH]_*n*_ (950–953, R = Me, Et, ^*n*^Pr, ^*n*^Bu) obtained from ZnCO_3_ and HSPh reacted in alcoholic solutions.^536^ Two opposing Zn atoms carry SPh ligands bridging to the next cluster to form the polymer, while the others carry a terminal alcohol or SPh ligand respectively which form hydrogen bonds to extend the structure to loose layers. There are a couple of related one dimensional adamantane-type clusters connected *via* organic ligands [(Zn_4_(SPh)_8_L]_*n*_ (954–955, L = *trans*-1,2-bis(4-pyridyl)ethylene, 4,4′-bipy).^537^ The first one forms a zigzag chain, while the second exhibits a helical buildup. They both are obtained from solvothermal reactions in water with HSPh, Zn(MeCOO)_2_ and the appropriate ligand.

In the cadmium thiolate cluster network [(Cd_4_(SPh)_8_]_*n*_ (956), all Cd moieties carry bridging thiolate ligands to form a three dimensional architechture with helical arrangement of the adamantane-type clusters similar to that in cristobalite.^538^ This is obtained by reacting Ca(NO_3_)_2_ with HSPh and NEt_3_ in ethanol. An alternative reaction strategy is the poylmerisation of 447 in THF/MeCN at elevated temperatures.^232^ The same structural motif, albeit in a different crystallographic space group, is found for the *para*-fluorinated species [(Cd_4_(SC_6_H_4_F-4)_8_]_*n*_ (957).^539^

When exchanging the fluorine with Br or Me groups, the structure of the product varies significantly. The methylated species also forms a three-dimensional network, but arranges in cyclic groups of 4, 6 or 8 clusters, resulting in a porous zeolite like buildup. The bromide congener forms a layered structure [{Cd_6_(SC_6_H_4_Br-4)_15_}(CdSC_6_H_4_Br-4){Cd(dmf)_3_}]_*n*_ (958) and incorporates solvent molecules in its buildup. This leads to two distinct clusters, each with three cadmium atoms linking to the next cluster *via* briding thiolate units, but also each with one terminally coordinated metal center, either by a thiolate or three dmf molecules. The chlorinated species has been synthesized as well, but could not be elucidated crystallographically due to a fast decomposition of the crystals.

956 can be partially decomposed to chains of [(Cd_4_(SPh)_8_PPh_3_]_*n*_ (959) by the addition of PPh_3_.^232^

The higher homolog [(Cd_4_(SePh)_8_]_*n*_ (960) is isostructural to its thiolate compound and prepared in the same manner by creating the selenolate and reacting with a Cd salt, CdCl_2_ in this case.^540^

Using two Cd sources, (PhSe)_2_Cd and CdX (Cl, Br), and a coordinating ligand, PR_3_, to stabilize intermediates leads to one dimensional chains of [Cd_4_(PhSe)_7_X(PR_3_)]_*n*_ (961–962), where two Cd atoms in each cluster connect to the next *via* PhSe bridges and the others carry a PPh_3_ or X ligand.^541,542^

Four isostructural Hg species [Hg_4_(PhSe)7(X)solv]_*n*_ (963–966, Fig. 21) with different (pseudo)halides (X) and solvent ligands were obtained by changing to the appropriate salt, solvent and stabilizing ligand.^543–545^ This chemistry could also be translated to a Te congener [Hg_4_(PhTe)_7_IPy]_*n*_ (967), although in this case, (PhTe)_2_Hg was used alongside CdI_2_ instead of the mercury halide, which did not lead to the inclusion of Cd into the final compound.^546^

For the group 13/16 combination, some In/Se networks are known. (C_5_H_5_NH_2_)_24_[In_28_Se_54_(H_2_O)_4_] (968, Fig. 21), formed by the elements and piperidine in aqueous solution through Method B, features a three dimensional structure of corner condensed adamantane-type clusters with some indium sites coordinated by water molecules.^547^ These positions can be partially substituted by Bi atoms when adding Bi(NO_3_)_3_ to the reaction mixture, leading to a doped structure (969). Other linking modes are obtained for the three dimensional network [μ_3_-Se_4_]_3.27_[In_49.88_Se_95.92_](C_5_H_12_N)_26.0_·(C_2_H_8_N)_42.4_ (970), in which [In_4_Se_10_] clusters are linked by μ_3_ Se and [InSe_4_] fragments, a structure obtained from the elements in piperidine solvothermally.^548^ Adding 1,4-dioxane and 3,5-dimethylpyridine to the mixture changes the outcome to [In_4_Se_10_]·(C_7_H_16_N)_1.8_·(C_2_H_8_N)_2.2_ (971), comprising μ-Se_3_ linkers between the individual clusters.

In group 14/16 adamantane-type clusters, extended structures are produced mainly by adding transition metal complexes to ammonium or alkaline metal salts of [Q_4_E_10_] clusters (972–994).^260,263,272,275,281,549–552^ By utilizing two different transition metal complexes during the synthesis, a more complex Cu_2_MS_6_ (M = Mo, W) linker between Ge/S adamantane-type compounds was obtained to form the MOF-like {[Me_4_N]_4_[(Ge_4_S_10_)Cu_4_M_2_S_8_]}_*n*_ (995–996, Fig. 21).^553^ The concept could also be used to introduce another group 14 element, here tin, to Ge/S adamantanes by adding SnCl_2_ to K+ or Cs+ salts of [Ge_4_S_10_]^4−^ in ionic liquids, with the exact outcome dependent on the ionic liquid used (997–999).^554,555^ In one case, {[BMIm]_2_[Ge_4_Se_9_]}_*n*_ (997), this approach did not lead to the incorporation of Sn into the structure.^554^

In another case, utilizing the functional ligand in 579, manganese complexes were used to form a coordination polymer forming a layered structure [Mn_2_{(OOCC_2_H_4_Ge)_4_S_6_}(MeOH)(dmf)_2_]_*n*_ (1000) by using the transition metal as a linker between the acid moieties.^556^

Most other transition metal linked adamantanes are isolated after reactions of simple binary or elemental precursors in solution (1001–1005)^549,557–560^ or, in one case, the solid state,^561^ which results in clusters linked by disordered Cu_0.44_Ge_0.56_S_4_ sites (in 1006).

Two examples also showcase the possibility of creating manganese linked adamantane-type structures by Method E, the extraction of a solid created from a melt of simple precursors (1007–1008).^275,282^ This led to the only example of a tellurium adamantane in network structures (1007).

Pure group 14/16 structures can also be obtained, one of them containing the same polymeric chain [Ge_4_Se_9_]^2−^ previously discussed as the surprising outcome of a reaction of a Ge/S adamantane with SnCl_2_. In this case, the compound {[Pr_2_NH_2_][PrEtNH_2_][Ge_4_S_9_]}_*n*_ (1009) could be isolated from a solvothermal reaction of GeS_2_ and [Pr_2_NH_2_]Cl in the presence of NaHCO_3_.^562^

The other example {[Me_4_N]_2_[OSn_5_Se_10_]}_*n*_ (1010) consists of a corner condensed oxygen centered [μ_4_-OSn_4_Se_10_] adamantane-type structure synthesized solvothermally from the elements and [Me_4_N]OH.^278^

Aside from ionic or ligand decorated networks, partial acidic decomposition of 497 led to a novel modification of GeS_2_, δ-GeS_2_ (1011), with corner condensed Ge_4_S_10_ adamantanes, which can be derived from two interpenetrating cristobalite-like structures of γ-GeS_2_.^266^

**Table 21:** Networks of type 2 supertetrahedra^a^

Compound	Reagents/conditions	Method
[{P_4_(NMe)_6_}CuI]_*n*_ (936)	[P_4_(NMe)_6_] (131), CuI/MeCN, 2 days	T^533^
[{P_4_(NEt)_6_}CuI]_*n*_ (937)	[P_4_(NEt)_6_] (132), CuI/MeCN, 2 days	T^81^
[{P_4_(NMe)_6_}(CuI)_2_(MeCN)_2_]_*n*_ (938)	[P_4_(NBn)_6_] (133), CuI/MeCN, 3 days	T^81^
[{P_4_(NMe)_6_}_2_(CuCl)_3_(MeCN)_2_]_*n*_ (939)	[P_4_(NMe)_6_] (131), CuCl/MeCN, 90 min	T^526^
[{P_4_(NMe)_6_}(CuCl)_2_]_*n*_ (940)	[P_4_(NMe)_6_] (131), CuCl/MeCN, 2 days	T^526^
[{PN(*p*-^*n*^BuPhP)_0.5_}_4_(NMe)_6_]_*n*_ (941)	[(PNSiMe_3_)_4_(NMe)_6_] (148), *p*-^*n*^BuPhPCl_2_/THF, 90 °C, 5 days	T^95^
[{PN(TiCl_2_)_0.5_}_4_(NMe)_6_]_*n*_ (942)	[(PNSiMe_3_)_4_(NMe)_6_] (148), TiCl_4_/MeCN, 100 °C, 4 days	T^95^
[Ba(H_2_O)_5_][Cr_4_(OH)_4_(hpdta)_2_] (943)	H_5_hpdta, CrCl_3_, BaCl_2_/H_2_O, 85 °C, 20 h	J^119^
[Mn_4_(SPh)_8_]_*n*_ (944)	[Mn{N(SiMe_3_)_2_}_2_]_2_, HSPh/THF, 0 °C, 2 h	C^534^
[{Cu_4_{SC(NH_2_)_2_}_6_}_2_{SC(NH_2_)_2_}_3_]_*n*_(SO_4_)_4_ (945)	CuSO_4_, SC(NH_2_)_2_, H_2_SO_4_/H_2_O, 80 °C	C^187^
[*N*-methylpyridinium]_2_[{Cu_4_(dmit)_3_}_2_]_*n*_ (946)	[Cu(MeCN)_4_][ClO_4_], Na_2_dmit, [*N*-methylpyridinium]I/MeOH	C^563^
[^*n*^Bu_4_N]_2_[{Cu_4_(dmit)_3_}_2_]_*n*_ (947)	[Cu(MeCN)_4_][ClO_4_], Na_2_dmit, [^*n*^Bu_4_N]Br/MeOH	C^563^
[(μ_3_-Cu)Cu_4_(SePh)_6_(CuPPh_3_)_3_(4,4′-bipy)]_*n*_ (948)	Cu(MeCOO), PhSeSiMe_3_, PPh_3_, 4,4′-bipy/DME, 4 h	C^535^
{[Et_4_N][(Ag_4_(dmit)_3_]dmf}_*n*_ (949)	AgNO_3_, H_2_dmit, Na, [Et_4_N]Br, NH_3_/MeOH, DMF	C^564^
[(Zn_4_(SPh)_8_MeOH]_*n*_ (950)	HSPh, ZnCO_3_/MeOH, 55 °C, 5 days	C^536^
[(Zn_4_(SPh)_8_EtOH]_*n*_ (951)	HSPh, ZnCO_3_/EtOH, 78 °C, 2 h	C^536^
[(Zn_4_(SPh)_8_^*n*^PrOH]_*n*_ (952)	HSPh, ZnCO_3_/MeOH, ^*n*^PrOH, 10 to 97 °C, 5 h	C^536^
[(Zn_4_(SPh)_8_^*n*^BuOH]_*n*_ (953)	HSPh, ZnCO_3_/MeOH, ^*n*^BuOH, 10 to 117 °C, 5 h	C^536^
[(Zn_4_(SPh)_8_(*trans*-1,2-bis(4-pyridyl)ethylene)]_*n*_ (954)	HSPh, Zn(MeCOO)_2_, *trans*-1,2-bis(4-pyridyl)ethylene/H_2_O, 165 °C, 5 days	B^537^
[(Zn_4_(SPh)_8_(4,4′-bipy)]_*n*_ (955)	HSPh, Zn(MeCOO)_2_, 4,4′-bipy/H_2_O, 165 °C, 5 days	B^537^
[(Cd_4_(SPh)_8_]_*n*_ (956)	Cd(NO_3_)_2_, HSPh, NEt_3_/EtOH, DMF or [Me_4_N]_2_[(CdSPh)_3_(CdCl)(SPh)_6_] (447)/MeCN, H_2_O, 100 °C	C/T^232,538^
[(Cd_4_(SC_6_H_4_F-4)_8_]_*n*_ (957)	Cd(NO_3_)_2_, HSC_6_H_4_F-4, NEt_3_/EtOH, DMF	C^539^
[{Cd_6_(SC_6_H_4_Br-4)_15_}(CdSC_6_H_4_Br-4){Cd(dmf)_3_}]_*n*_ (958)	Cd(NO_3_)_2_, HSC_6_H_4_Br-4, NEt_3_/EtOH, DMF	C^539^
[(Cd_4_(SPh)_8_PPh_3_]_*n*_ (959)	[(Cd_4_(SPh)_8_]_*n*_ (939), PPh_3_/THF, DMF	I^232^
[(Cd_4_(SePh)_8_]_*n*_ (960)	CdCl_2_, HSePh, NaOH/MeOH, H_2_O	C^540^
[Cd_4_(PhSe)_7_X(PPh_3_)]_*n*_ (961–962, X = Cl, Br)	(PhSe)_2_Cd, CdX_2_, PPh_3_/MeOH, 130 °C, 1 h	B^541,542^
[Hg_4_(PhSe)_7_BrPy]_*n*_ (963)	(PhSe)_2_Hg, HgBr_2_, 1,3-bis(4-nitrophenyl)triazene/THF, Py, 100 min	C^543^
[Hg_4_(PhSe)_7_ClPy]_*n*_ (964)	(PhSe)_2_Hg, HgCl_2_, PPh_3_/THF, Py, 5 h	C^544^
[Hg_4_(PhSe)_7_Br(dmf)]_*n*_ (965)	(PhSe)_2_Hg, HgI_2_, 4,4′-bipy/DMF, 5 h	C^544^
[Hg_4_(PhSe)_7_(SCN)Py]_*n*_ (966)	(PhSe)_2_Hg, Hg(SCN)_2_,/MeOH, THF, Py, 1 h	C^545^
[Hg_4_(PhTe)_7_IPy]_*n*_ (967)	(PhTe)_2_Hg, CdI_2_/THF, Py, 90 min	C^546^
(C_5_H_5_NH_2_)_24_[In_28_Se_54_(H_2_O)_4_] (968)	In, Se, piperidine/H_2_O, 170 °C, 7 days	B^547^
(C_5_H_5_NH_2_)_24_[In_28−*x*_Bi_*x*_Se_54_(H_2_O)_4_] (969)	In, Se, Bi(NO_3_)_3_, piperidine/H_2_O, 170 °C, 7 days	B^547^
[μ_3_-Se_4_]_3.27_[In_49.88_Se_95.92_](C_5_H_12_N)_26.0_·(C_2_H_8_N)_42.4_ (970)	Se, In, piperidine/DMF, 170 °C, 5 days	B^548^
[In_4_Se_10_](C_7_H_16_N)_1.8_ (C_2_H_8_N)_2.2_ (971)	Se, In, 1,4-dioxane, 3,5-dimethylpyridine/DMF, 170 °C, 7 days	B^548^
{[Me_4_N]_2_[MnGe_4_S_10_]}_*n*_ (972)	[Me_4_N]_4_[Ge_4_S_10_] (496), Mn(Me_2_CO)_2_/H_2_O, 24 h or GeS_2_, Mn(Me_2_CO)_2_, [Me_4_N]Cl, NaHCO_3_/H_2_O, 120 °C, 2 days	B/T^263,549,550^
{[Me_4_N]_2_[FeGe_4_S_10_]}_*n*_ (973)	[Me_4_N]_4_[Ge_4_S_10_] (496), Fe(Me_2_CO)_2_/H_2_O, 24 h or GeS_2_, FeCO_3_, [Me_4_N]Cl, [H_4_N]HCO_3_/H_2_O, 220 °C, 2 days	B/T^260,549,557^
{[Me_4_N]_2_[CoGe_4_S_10_]}_*n*_ (974)	[Me_4_N]_4_[Ge_4_S_10_] (496), Co(Me_2_CO)_2_/H_2_O, 24 h	T^549^
{[Me_4_N]_2_[ZnGe_4_S_10_]}_*n*_ (975)	[Me_4_N]_4_[Ge_4_S_10_] (496), Zn(Me_2_CO)_2_/H_2_O, 24 h	T^549^
{[Me_4_N]_2_[Ag_2_Ge_4_S_10_]}_*n*_ (976)	[Me_4_N]_2_[Ge_4_S_10_] (496), Na_2_S_2_O_3_, Ag_2_NO_3_/H_2_O, 16 h	T^551^
{Cs_2_[FeGe_4_S_10_]}_*n*_ (977)	Cs_4_[Ge_4_S_10_] (493), FeSO_4_/H_2_O	T^260^
{[Me_4_N]_2_[Cu_2_Ge_4_S_10_]}_*n*_ (978)	[Me_4_N]_2_[Ge_4_S_10_] (496), NaBr, CuCl/H_2_O, MeCN, 16 h	T^551^
{[C_*n*_H_2*n*+1_NC_5_H_5_]_2_[Pt_2_Ge_4_S_10_]}_*n*_ (979–984, *n* = 12, 14, 16, 18, 20, 22)	[Me_4_N]_2_[Ge_4_S_10_] (496), [C_*n*_H_2*n*+1_NC_5_H_5_]Br, K_2_[PtCl_4_]/formamide, 80 °C, 18 h	T^552^
{[(CH_3_CH_2_)_4_N]_3_[AgGe_4_S_10_]}_*n*_ (985)	[EtNH_3_]_3_[MeNH_3_][Ge_4_S_10_] (497), AgOAc, [(Et)_4_N]Br, methylurea/130 °C, 24 h	T^281^
{[(Et)_4_N]_3_[CuGe_4_S_10_]}_*n*_ (986)	[EtNH_3_]_3_[MeNH_3_][Ge_4_S_10_] (497), CuCl, [(Et)_4_N]Br, methylurea/130 °C, 24 h	T^281^
{[Me_4_N]_2_[MnGe_4_Se_10_]}_*n*_ (987)	[Me_4_N]_4_[Ge_4_Se_10_] (505), Mn(OAc)_2_/H_2_O	T^272^
{[Me_4_N]_2_[FeGe_4_Se_10_]}_*n*_ (988)	[Me_4_N]_4_[Ge_4_Se_10_] (505), FeSO_4_/H_2_O	T^272^
{[C_16_H_33_NC_5_H_5_]_2_[Pt_2_Ge_4_Se_10_]}_*n*_ (989)	[Me_4_N]_2_[Ge_4_Se_10_] (505), [C_16_H_33_NC_5_H_5_]Br, K_2_[PtCl_4_]/Formamide, 80 °C, 18 h	T^552^
{[C_16_H_33_NC_5_H_5_]_2_[Pt_2_Sn_4_Se_10_]}_*n*_ (990)	[Me_4_N]_2_[Sn_4_Se_10_] (516), [C_16_H_33_NC_5_H_5_]Br, K_2_[PtCl_4_]/Formamide, 80 °C, 18 h	T^552^
{[Me_4_N]_2_[MSn_4_Se_10_]}_*n*_ (991–993, M = Fe, Co, Mn)	[Me_4_N]_2_[Sn_4_Se_10_] (561), MCl_2_/H_2_O	T^275^
{[Me_4_N]_2_[ZnSn_4_Se_10_]}_*n*_ (994)	[Me_4_N]_2_[Sn_4_Se_10_] (561), ZnCl_2_, Na_4_EDTA/H_2_O	T^275^
{[Me_4_N]_4_[(Ge_4_S_10_)Cu_4_M_2_S_8_]}_*n*_ (995–996, M = Mo, W)	[Me_4_N]_4_[Ge_4_S_10_] (496), [Me_4_N]_2_[MS_4_], Cu(OAc)_2_/BuOH, H_2_O, DMF, 100 °C, 3 days	T^553^
{[BMIm]_2_[Ge_4_Se_9_]}_*n*_ (997)	K_4_[Ge_4_Se_10_] (501), SnCl_2_ 2,6-dimethylmorpholine/[BMIm][BF_4_], 150 °C, 3 days	T^554^
{[BMMIm]_2_[Ge_4_SnSe_10_]}_*n*_ (998)	K_4_[Ge_4_Se_10_] (501), SnCl_2_ 2,6-dimethylmorpholine/[BMMIm][BF_4_], 150 °C, 3 days	T^554^
{(BMIm)_2_[Sn^II^(Ge^IV^_4_Se_10_)]}_*n*_ (999)	Cs_4_[Ge_4_Se_10_] (503), SnCl_2_, 2,6-dimethylmorpholine, [Pt@Bi_10_][AlBr_4_]_4_/(BMIm)Cl, (BMIm)[BF_4_], 120 °C, 4 days	T^555^
[Mn_2_{(OOCC_2_H_4_Ge)_4_S_6_}(MeOH)(dmf)_2_]_*n*_ (1000)	[{HOOC(CH_2_)_2_Ge}_4_S_6_] (579), MnCl_2_/MeOH, DMF, 100 °C, 24 h	T^556^
{[Me_4_N]_2_[Mn_0.86_Co_0.14_Ge_4_S_10_]}_*n*_ (1001)	GeS_2_, [Me_4_N]HCO_3_, [Me_4_N]OH, Mn(Me_2_CO)_2_, Co(Me_2_CO)_2_, H_2_S/H_2_O, EtOH, 78 to 150 °C, 3 days	G^549^
{[Me_4_N]_2_[CdGe_4_S_10_]}_*n*_ (1002)	GeS_2_, CdSO_4_, [Me_4_N]Cl, [H_4_N]HCO_3_/H_2_O, 220 °C, 2 days	B^557^
{(H_2_dabco)[MnGe_4_S_10_]}_*n*_ (1003)	GeS_2_, MnCl_2_, dabco/H_2_O, CO_2_, 120 °C, 7 days	B^558^
{(H_2_dabco)(H_3_O)[AgGe_4_S_10_]}_*n*_ (1004)	GeS_2_, Ag(OAc), dabco/H_2_O, 130 °C, 2 days	B^559^
{[Et_4_N]_2_[Cu_2_Ge_4_Se_10_]}_*n*_ (1005)	GeS_2_, Cu(OAc), [Me_4_N]HCO_3_/H_2_O, 150 °C, 1 day	B^560^
{[Me_4_N]_6_[(Cu_0.44_Ge_0.56_S_2.23_)_4_(Ge_4_S_8_)_3_]}_*n*_ (1006)	GeS_2_, [Me_4_N]HCO_3_, Cu(OAc)_2_, [Me_4_N]Cl/150 °C, 24 h	A^561^
{[Me_4_N]_2_[MnGe_4_Te_10_]}_*n*_ (1007)	1. K_2_Te, Te, Ge/heat to melt	E^275^
	2. [Me_4_N]Br, MnCl_2_/en, 100 °C, 12 h	
{[Li_4_(H_2_O)_8_][MnGe_4_Se_10_]}_*n*_ (1008)	1. LiSe_2_, Ge, Se/heat to melt	E^282^
	2. MnCl_2_/MeOH, H_2_O, 24 h	
{[Pr_2_NH_2_][PrEtNH_2_][Ge_4_S_9_]}_*n*_ (1009)	GeS_2_, [Pr_2_NH_2_]Cl, NaHCO_3_/H_2_O, 125 °C, 24 h	B^562^
{[Me_4_N]_2_[OSn_5_Se_10_]}_*n*_ (1010)	Sn, Se, [Me_4_N]OH,/H_2_O, 150 °C, 16 days	B^278^
δ-GeS_2_ (1011)	[Me_4_N]_4_[Ge_4_S_10_] (496), HCl/H_2_O, 50 °C, 24 h	I^266^

a dmit = 4,5-dimercapto-1,3-dithiole-2-thionato, EDTA = ethylenediamine-tetraacetate, BMMIm = 1-butyl-2,3-dimethyl-imidazolium.

### 2.2 1,3,5,7-Tetrasubstituted adamantane derivatives

**Scheme 4:**
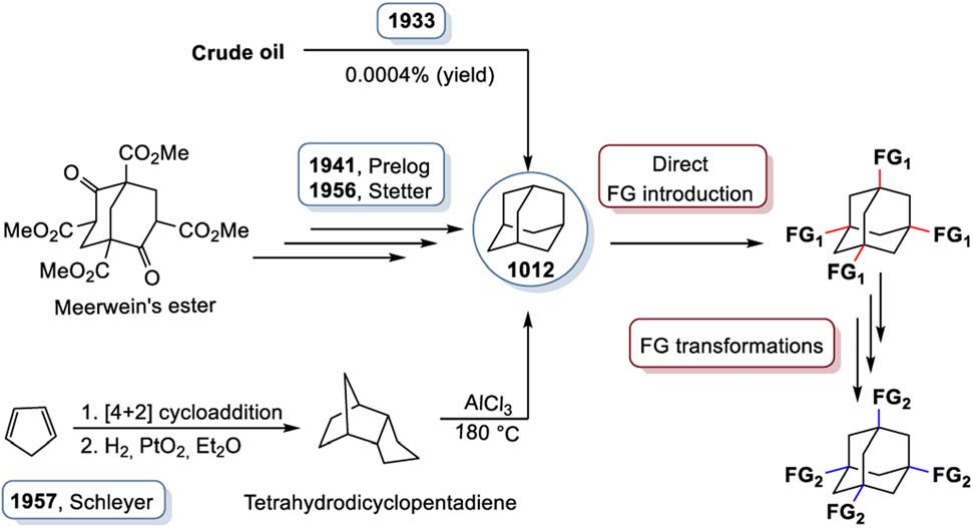
Synthesis and functionalization of tetra-substituted adamantane.

In 1941, the first synthesis of adamantane (1012), the smallest so-called diamondoid,^565,566^ was achieved by Prelog, yielding 1.5% from Meerwein's ester through a series of conventional enolate alkylation techniques, Wolff–Kishner reductions, and a final double decarboxylation step (Scheme 4).^567^ Subsequent refinements by Stetter increased the yield to 6.5%, but the method remained intricate, involving multiple stages for the removal of functional groups used in adamantane synthesis.^568^ In 1957, Paul von Ragué Schleyer introduced a groundbreaking Lewis acid-promoted rearrangement of tetrahydrodicyclopentadiene, offering an alternative pathway to adamantane synthesis. This isomerization method significantly enhanced the yield by approximately 40%.^569^

These sections center on the synthesis of 1,3,5,7-tetra-substituted 1012 and explores its applications in advancing nonlinear optical properties.^570,571^ In these sections, we employ two distinct approaches. The first approach concentrates on directly incorporating functional groups onto the adamantane core. The second approach delves into functional group transformations, commencing from 1,3,5,7-tetra-substituted adamantane as the starting point.

#### 2.2.1 Direct functional group introduction

Adamantane, a tricyclic hydrocarbon comprised of interconnected chair cyclohexane rings, exhibits remarkable symmetry and inherent resistance to direct modification due to the absence of functional groups. Nevertheless, there exist several approaches to introduce functional groups into adamantane.^572,573^ One such method involves reactions of adamantane with electrophiles such as bromine (Br_2_). Subsequently, nucleophilic substitutions enlarge the spectrum of possible substituents; these reactions must exclusively procede through an S_*N*_1 mechanism. Hence, this mechanism involves the reaction of tertiary adamantyl cations with nucleophiles. Notably, the nucleophilic C–H-bond substitution 1012 can be accomplished directly with strong acids such as hydrochloric acid (HCl) and hydrobromic acid (HBr).

In general, these conditions facilitate the abstraction of hydride ions while also serving as sources of nucleophilic species.

**Scheme 5:**
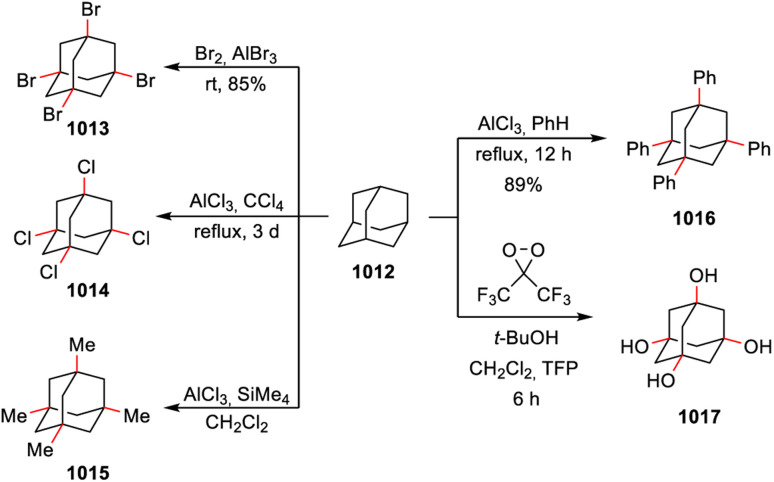
Direct tetra-functionalization of adamantane.

Direct bromination of 1012 leads to the formation of only 1-bromo adamantane.^574,575^ However, the presence of Friedel–Crafts-type catalysts like AlCl_3_ and AlBr_3_ allows for the gradual replacement of more tertiary C–H bonds with bromine. The successful synthesis of 1,3,5,7-tetrabromoadamantane (1013) has been achieved by utilizing AlCl_3_ and Br_2_ at 150 °C (Scheme 5).^576,577^ Note that the use of larger amounts of AlCl_3_ leads to the generation of not only 1013 but also small amounts of 1-chloro-3,5,7-tribromoadamantane in around 12% yield. In addition, synthesis of 1013 has been achieved in the presence of AlBr_3_ under sealed tube conditions at 150 °C. This approach avoids halogen exchange during the synthesis of 1013 by utilizing aluminum tribromide.^578^ The use of two equivalents of AlBr_3_ resulted in the clean formation of 1013 with 85% yield at room temperature.^579^ The established one-step method to synthesize 1,3,5,7-tetrachloroadamantane (1014) proceeds by refluxing adamantane in CCl_4_ in the presence of AlCl_3_ (Scheme 5).^580^

Direct methylation of 1012 with tetramethylsilane as the methylation agent and a Friedel–Crafts catalyst has been explored and optimized for the synthesis of 1,3,5,7-tetramethyladamantane (1015, Scheme 5). With the introduction of four methyl groups in the presence of AlCl_3_, adamantane underwent fourfold methylation of all bridgehead carbons.^581^

As is well known, Friedel–Crafts alkylations can generate mixtures of substitution products, and the selective introduction of aryl groups at the 1,3,5,7-positions of 1012 requires precise control of reaction conditions.

In 1968, Stetter and Krause employed a two-step process to add phenyl groups to the adamantane molecule, resulting in the synthesis of 1,3,5,7-tetraphenyladamantane (1016, Scheme 5). Initially, they brominated adamantane using molecular bromine (Br_2_). Subsequently, in the presence of AlCl_3_ and benzene, phenyl groups were introduced *via* Friedel–Crafts alkylation.^582^

In 1972, Newman utilized the Friedel–Crafts catalyst along with *tert*-butyl bromide to synthesize 1016 from 1-bromoadamantane. This method allowed for selective Friedel–Crafts phenylation under controlled reaction conditions, resulting in the clean formation of 1016.^583^

Alternatively, 1016 was synthesized from adamantane under refluxing conditions, utilizing a catalytic amount of AlCl_3_. The reaction proceeded overnight giving a yield of 89%.^584^ Furthermore, 1012 can be directly converted into 1,3,5,7-tetrahydroxyadamantane (1017) under remarkably mild conditions, employing an excess of methyl(trifluoromethyl)dioxirane in solution (Scheme 5).^585^

**Scheme 6:**
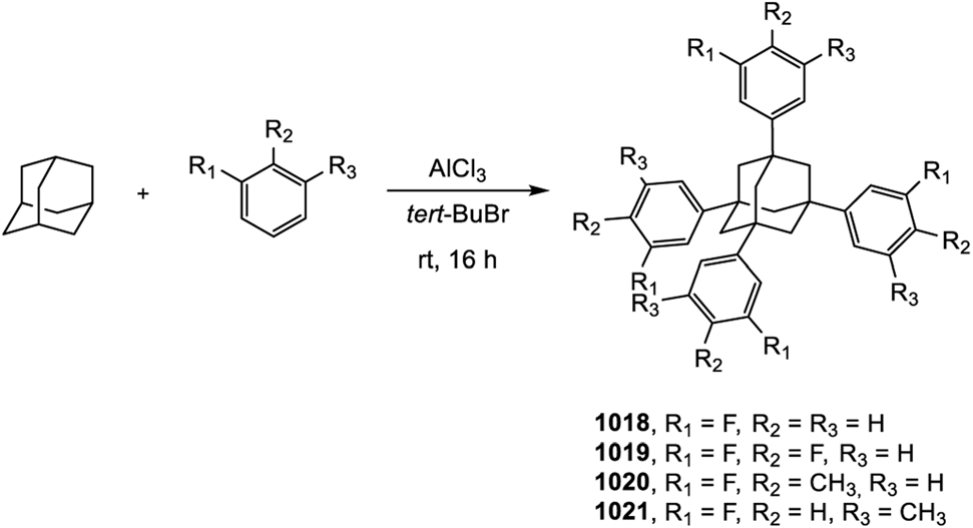
Direct meta-substituted tetra-functionalization of adamantane.

Recently, we reported a new meta-selective adamantane tetraarylation using substituted benzenes. This Friedel–Crafts-type reaction yields a large amount of all-*meta*-tetrafluorophenyl adamantane derivatives (1018–1021) in the presence of *tert*-BuBr as the additive and AlCl_3_ as the catalyst (Scheme 6).^586^

#### 2.2.2 Functional group transformations

Functional group transformations in organic synthesis are a fundamental and essential aspect of modern organic chemistry.^587,588^ Functional groups attached to adamantane derivatives can be modified to create a wide range of compounds with tailored properties. This field of study is at the core of organic synthesis, and plays a crucial role in designing and preparing complex adamantane derivatives for various applications, including materials science,^589–591^ pharmaceuticals,^592,593^ and agrochemicals.^594^ Functional group transformations involve converting one functional group into another while preserving the overall molecular structure, such as the adamantane core.^565,569,595–597^ Developing efficient and selective methods for functional group transformations encompasses a wide array of chemical reactions. These reactions can include substitution,^598–601^ addition,^602^ and elimination reactions,^603–605^ among others. They are applicable to various functional groups, including halides, alcohols, ketones, and carboxylic acids, among others. The choice of transformation method depends on the specific functional group and the desired product.

**Scheme 7:**
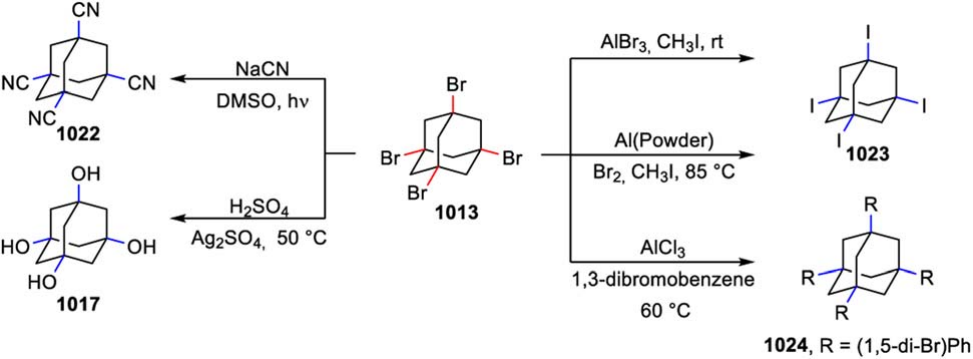
Functional Group transformations from 1,3,5,7-tetrabromoadamantane.

The conversion of 1,3,5,7-tetracyanoadamantane (1022, Scheme 7) from 1013 was achieved through a nucleophilic radical substitution reaction. Interestingly, no reaction occurred in the dark. However, upon photolysis with sodium cyanide in DMSO in a quartz vessel using a Rayonet reactor, a mixture was obtained where 1022 was the dominant product.^579^ The synthesis of 1,3,5,7-tetraiodoadamantane (1023) did not proceed directly from 1012. Initially, a bromination reaction was conducted to substitute hydrogen atoms on the adamantane bridgeheads with bromine atoms. This process involves halogen exchange in the presence of methyliodide, aluminum powder, and bromine, carried out at 80–85 °C for 45 min, as illustrated in Scheme 7.^606^ An improved procedure for 1023 involves the use of methyliodide and AlBr_3_, resulting in a yield of 91% (Scheme 7).^579^

The direct method to prepare 1,3,5,7-tetrahydroxyadamantane (1017) from 1012 utilizes a strong oxidation reagent such as dioxiranes, which poses a risk of explosion during their preparation (see Scheme 5). Target compound 1017 can be prepared conveniently from 1013 in the presence of concentrated H_2_SO_4_ and Ag_2_SO_4_.^582^ Exhaustive Soxhlet extraction improved the yield, increasing it to 98% compared to the 84% reported in the literature.^607^ Starting from 1013, the synthesis of 1,3,5,7-tetrakis(1,3-dibromophenyl)adamantane (1024) can be accomplished with 1,3-dibromobenzene and AlCl_3_ (Scheme 7).^608^

**Scheme 8:**
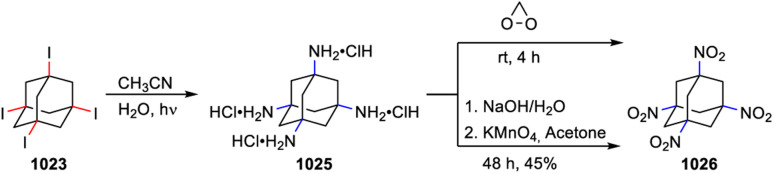
Synthesis of 1,3,5,7-tetranitroadamantane.

The nitration of 1012 with concentrated nitric acid in glacial acetic acid at elevated temperatures has been previously reported to yield 1-nitro-, 1,3-dinitro-, and 1,3,5-trinitroadamantanes, albeit in moderate to low yields.^609^ When adamantanes are subjected to nitration with nitrogen dioxide at elevated temperatures, the primary products are typically 1-nitro and 1,3-dinitro derivatives. Similarly, the photochemical reaction of N_2_O_5_ with 1012 primarily results in mononitration. Note that while the oxidation of *tert*-alkyl amines to their corresponding nitro compounds is a standard method, it has not been widely used in the past to prepare compounds containing more than two nitro groups. In a noteworthy synthesis, Sollot and Gilbert reported the hydrolysis of 1,3,5,7-tetraaminoadamantane·tetrahydrochloride (1025) to obtain the free tetraamine, which was subsequently oxidized using permanganate to yield the desired 1,3,5,7-tetranitrodamantane (1026) with a yield of 45% (Scheme 8).^576^Additionally, the powerful oxidizing agent dimethyldioxirane was employed to synthesize 1026, achieving an impressive yield of 91%.^606^

**Scheme 9:**
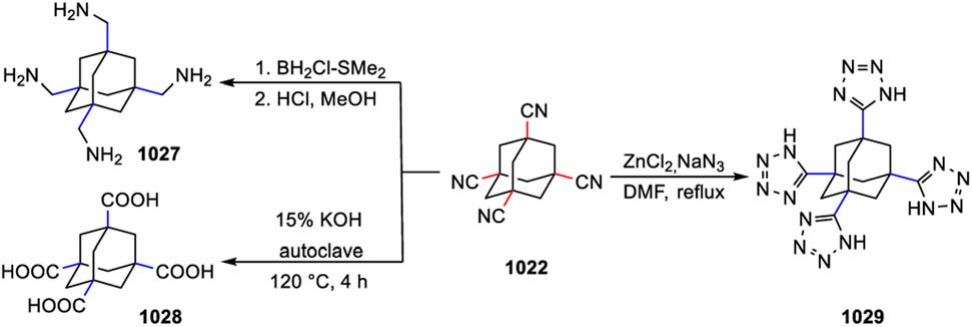
Functional Group transformations from 1,3,5,7-tetracynoadamantane.

The reduction of 1022 was accomplished using monochloroborane-methyl sulfide in refluxing THF. Subsequent reaction with dry methanolic HCl resulted in the formation of 1,3,5,7-tetrakis(aminomethyl)adamantane tetrahydrochloride with an impressive yield of 98%. To obtain the 1,3,5,7-tetrakis(aminomethyl)adamantane (1027), deprotonation of an aqueous solution with NaOH was performed (Scheme 9).^579^ Additionally, hydrolysis of 1022 led to 1,3,5,7-tetracarboxylic acid adamantane (1028). This method serves as an excellent alternative for preparing 1028, reducing the number of synthetic steps compared to those reported by others (Scheme 9).^610^

The use of ‘click chemistry,’ specifically tetrazole formation through the cycloaddition of azides to nitriles in the presence of ZnCl_2_, offers an especially cost-effective route to obtain tetrakis-tetrazole derivatives of adamantane. When applied to 1022, this process exhibited slightly slower kinetics compared to aromatic or unhindered aliphatic nitriles but ultimately yielded 1,3,5,7-tetrakis(tetrazol-5-yl)adamantane (1029, Scheme 9). The reaction can be conducted in DMF under reflux conditions for 48 h or at 175 °C in an autoclave within 6 h. The former conditions provide a purer product.^611^

**Scheme 10:**
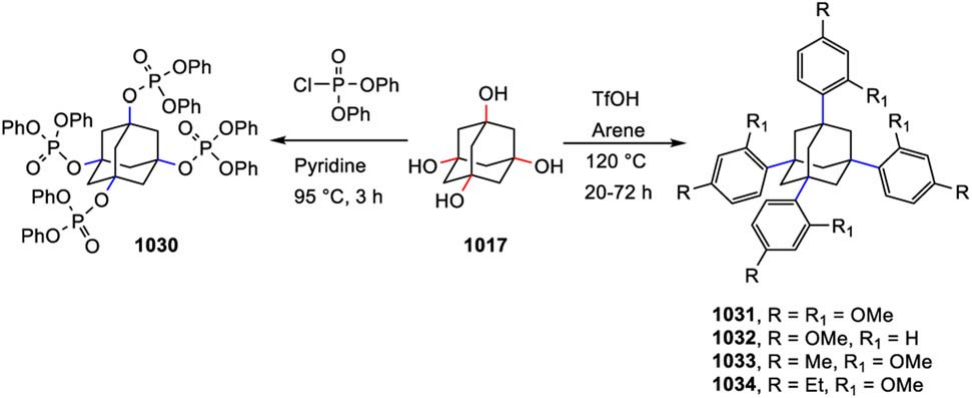
Functional group transformations from 1,3,5,7-tetrahydroxyadamantane.

The synthesis of 1,3,5,7-tetra(diphenylphosphate)adamantane (1030) with a yield of 62% can be achieved by reacting 1,3,5,7-tetrahydroxyadamantane (1017) with diphenyl chlorophosphate under controlled conditions (Scheme 10).^612^ Introduction of electron-rich arenes (substituted anisoles) to 1,3,5,7-tetrahydroxyadamantane through Friedel–Crafts alkylation results in symmetrical tetraaryladamantanes with yields ranging from 20–41% (1031–1034, Scheme 10). This alkylation process requires strong Brønsted acids, such as tosylic acid (TfOH), and elevated temperatures. The study reports that weaker acids or lower temperatures are ineffective, leading to low reactivity and consequently very low yields.^607,613–615^

In this context, various functional groups were introduced onto the phenyl rings of 1016 through electrophilic substitution at the *para*-positions of the phenyl moieties, as outlined in Table 22. The direct functionalization of 1016 through electrophilic aromatic substitution can serve as a good starting point for synthetic modifications, enabling access to a wide array of functional groups (R) attached to the aromatic moieties of 1,3,5,7-tetraaryladamantanes (R = Br, I, SO_2_Cl, NO_2_, COCH_3_, and CHO).

**Table 22:** 1,3,5,7-Tetraphenyladamantane postfunctionalization (1016)

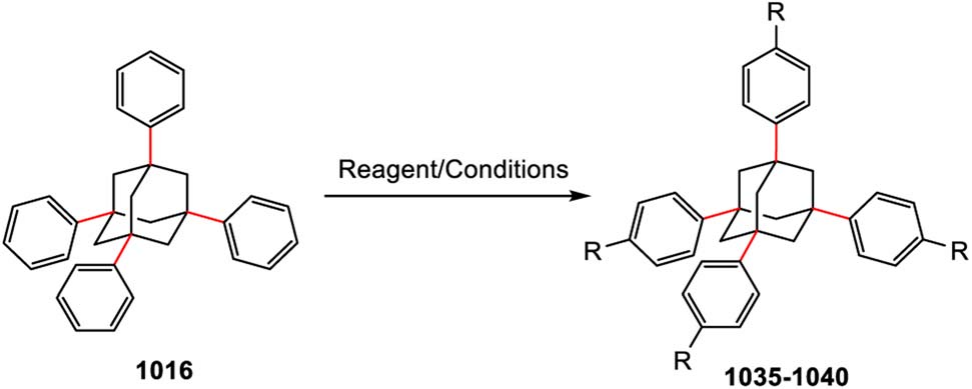		
R =	Reagents/conditions	Ref.
Br (1035)	Br_2_/CHCl_3,_ −78 °C, 12 h, 60%	616
Br (1035)	Fe, Br_2_/50 °C, 12 h, 94%	617
Br (1035)	Fe, Br_2_/70 °C, 7 h, 36%	618
I (1036)	PhI(CH_3_CO_2_)_2_, I_2_/CHCl_3_, 12 h, 80%	619
SO_2_Cl (1037)	HSO_3_Cl/1.6 h, 57%	620
NO_2_ (1038)	HNO_3_, Ac_2_O, AcOH/30 min, 35%	621 and 622
COCH_3_ (1039)	AlCl_3_, CHCOCl/16 h, 68%	626
CHO (1040)	TiCl_4_, CH_3_OCHCl_2_/CH_2_Cl_2_, −10 °C to rt, 12 h, 84%	^623^

The bromination of 1016 in liquid bromine selectively occurs at the *para*-position, yielding 1,3,5,7-tetrakis(4-bromophenyl)adamantane (1035, Table 22) with 60% yield, without the need for additional catalysts.^616^ However, when bromination of 1016 is conducted in the presence of Fe, 1035 is obtained in a significantly improved yield of 94%,^617^ which further increases to 96% at elevated temperatures.^618^ The iodination of 1016, using PhI(OCOCF_3_)_2_ in a chloroform solution of iodine, leads to the formation of 1,3,5,7-tetrakis(4-iodophenyl)adamantane (1036, Table 22).^619^ The sulfonation of 1016 using chlorosulfuric acid efficiently produces 1,3,5,7-tetra(phenyl-4-sulfonyl chloride)adamantane (1037) with a yield of 57% (Table 22).^620^ Starting material 1016 was subjected to nitration in fuming nitric acid at −15 °C for 30 min, yielding 1038 in low yield (Table 22). The degree of nitration can be controlled by adjusting the reaction time.^621,622^ The Friedel–Crafts acetylation of 1016 results in the formation of 1039 with good yield (Table 22). Additionally, 1,3,5,7-tetrakis-4-formylphenyladamantane (1039)^626^ was synthesized using a modified patented procedure involving the titanium tetrachloride-promoted formylation of 1040 (Table 22).^623^

The synthesis of 1041 was achieved by reacting 1036 with NaOMe and Cu(i)Br in dry MeOH/DMF, yielding a 47% yield. Similarly, the reaction of 1,3,5,7-tetrakis(4-bromophenyl)adamantane (1035) with NaOMe and Cu(i)Br in dry MeOH/DMF results in the formation of 1041 with a yield of 52% (tableref>/tableref>).^614^

**Table 23:** 1,3,5,7-Tetrakis(4-iodophenyl)adamantane (1036) postfunctionalization^a^

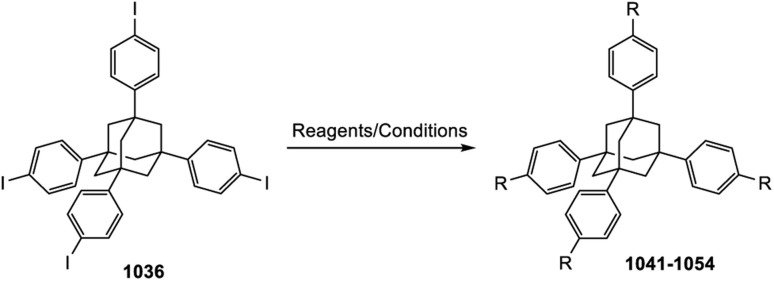		
R =	Reagents/conditions	Ref.
OMe (1041)	Cu(i)Br, NaOMe/MeOH, DMF, 100 °C, 12 h, 47–52%	587, 622
CN (1042)	CuCN/DMF, 160 °C, 16 h, 82%	625
PO(OEt)_2_ (1043)	1. HPO(OEt)_2_, PdCl_2_(PPh_3_)_2_, Et_3_N/PhH, 80 °C	627
	2. HCl/reflux, 76%	
Pyrrole (1044)	R–H, CuI, K_2_CO_3_, *N*,*N*-dimethylglycine/DMSO, 120 °C, 21–42%	628
Carbazole (1045)		
Imidazole (1046) benzimidazole (1047) phenylimidazole (1048)	R–H, CuI, K_2_CO_3_, *N*,*N*-dimethylglycine/DMSO, 120 °C, 5 days, 35–43%	628
2-CH_3_-imidazole (1049)	R–H, CuI, K_2_CO_3_, *N*,*N*-dimethylglycine/DMSO, 120 °C, 5 days, 41%	629
N(4-OMePh)_2_ (1050)	R–H, Pd(OAc)_2_, ^*t*^BuOK, ^*t*^Bu_3_P/toluene, 140 °C, 48 h, 72%	630
Ph (1051)	R–B(OH)_2_, Pd(PPh_3_)_4_, NaOEt/EtOH, PhH, 80–100 °C, 24 h, 35–45%	619 and 632
Ethynyl (1052)	1. Me_3_Si-ethynyl, Et_3_N, [PdCl_2_(PPh_3_)_2_], CuBr/80 °C, 72 h	621
	2. KF/CH_3_OH, 50 °C, 12 h, 74%	
Ethynyl (1052)	Me_3_Si-ethynyl, Pd(PPh_3_)_2_Cl_2_, CuI, Et_3_N, KF/MeOH, 5 days, 81%	621 and 631
I(OAc)_2_ (1053)	MCPBA/CH_2_Cl_2_, AcOH, rt, 12 h, 97%	633
Stilbenyl (1054)	Styrene, Pd(OAc)_2_, K_2_CO_3_, ^*n*^Bu_4_NBr, DMA/105 °C, 24%	634

a MCPBA = *m*-chloroperbenzoic acid.

The synthesis of 1,3,5,7-tetrakis(4-cyanophenyl)adamantane (1042) commenced with 1036, using the Rosenmund–von Braun reaction. Typically, in the literature, ethane-1,2-diamine is used to eliminate the nitrile–copper cyanide complexes and is followed by nitrile extraction to obtain the desired product.^592,624^ However, in this particular case, ethane-1,2-diamine proved to be inefficient, and the use of an excess of aqueous KCN was found to be more effective in the synthesis of 1042 (Table 23).^625^

1,3,5,7-Tetrakis(4-phosphonatophenyl)adamantane (1043), was synthesized through a two-step process but without isolating the intermediate. First, a palladium-catalyzed P–C coupling reaction between 1036 and diethylphosphite was carried out. Subsequently, the resulting phosphonic acid diethyl ester was subjected to acidic hydrolysis to obtain 1043 (Table 23).^627^

A copper(i)-catalyzed coupling reaction was employed to synthesize various derivatives of 1044–1049 (Table 23). This reaction involved the use of pyrrole, carbazole, imidazole, benzimidazole, phenylimidazole, and 2-CH_3_-imidazole as reactants. The reaction took place in the presence of *N*,*N*-dimethylglycine and DMSO at a temperature of 120 °C for a duration of 5 days.^628,629^

The synthesis of 4,4′,4′′,4′′′–(adamantane-1,3,5,7-tetrayl)tetrakis(*N,N*-bis(4-methoxyphenyl)aniline) (1050) was achieved by combining bis(4-methoxyphenyl)amine and 1036 in solution in the presence of Pd(OAc)_2_, *t*-Bu_3_P, and *t*-BuOK (Table 23).^630^ The reaction of 1036 with phenyl boronic acid under Suzuki coupling conditions yielded compound 1051 (Table 23). This compound is soluble in CHCl_3_, making it easy to purify and characterize.^619^ It can readily be converted to 1,3,5,7-tetrakis(4-trimethylsilyl-ethynylphenyl)adamantane by reacting it with Et_3_N, trimethylsilylacetylene, Pd(PPh_3_)_2_Cl_2_, and CuI in toluene. The crude product can then be deprotected to give 1,3,5,7-tetrakis(4-ethynylphenyl)adamantane (1052) in a yield of 74% (Table 23).^621^ This product was also prepared by a palladium/copper co-catalytic system for coupling 1036 with Me_3_Si-ethynyl in the presence of Et_3_N and DMSO.^621,631^

Oxidation of 1036 by conventional methods with peracetic acid (30% H_2_O_2_ and acetic anhydride), sodium perborate (NaBO_3_·*n*H_2_O) in acetic acid, or sodium periodate (NaIO_4_) unexpectedly gave 1,3,5,7-tetrakis[4-(diacetoxyiodo)phenyl]adamantane (1053) in low yield, accompanied by poorly soluble and unidentifiable polymeric products (Table 23). After further investigations, it was finally possible to synthesize 1053 in 97% yield by using MCPBA in CH_2_Cl_2_/AcOH (1 : 1) under dilute conditions.^633^ The synthesis of 1,3,5,7-tetrakis(4-stilbenylphenyl)adamantane (1054) is readily achieved by reacting compound 1036 with excess styrene under palladium-mediated Heck coupling conditions, resulting in an 86% yield. However, when starting with 1,3,5,7-tetrakis(4-bromophenyl)adamantane (1035) under analogous conditions, consistently lower yields were obtained.^634^

## 3. Optical properties: linear optical phenomena and photocurrent conversion

Compounds with an adamantane-type scaffold have most commonly been investigated for their luminescence properties over the years. Especially molecules with group 16 or group 17 elements in the E position have been the focus of such investigations, but other emissive examples have been reported as well.

Compounds containing the highly distorted group 11 chalcogenide adamantane cations [(ER)_4_M_6_{(Ph_2_P)_2_R}_4_]^2+^ (848–853, 855–857) have been investigated systematically for their photoluminescence behavior.^493,494,496^ While the emission of the copper complexes [(SePh)_4_Cu_6_{(Ph_2_P)_2_R}_4_][BF_4_]_2_ (848–849, R = CH_2_, NH) in solution was of low intensity when compared to other related copper clusters, the long lifetime of the excited state suggests a spin-forbidden transition likely stemming from a ligand-to-metal charge transfer between the PhSe fragments and the Cu centers.^493^ Most silver congeners only feature a significant luminescence at low temperatures of about 77 K in the solid state, which consists of a single emission peak for [(ER)_4_Ag_6_{(Ph_2_P)_2_Me}_4_][PF_6_]_2_ (850–853, ER = SPh, SC_6_H_4_Me-p, SePh, SeC_6_H_4_Cl-p) at around 700 nm. With a rising electron richness of the ER fragments from 850 to 853, the signal shifts to higher energies (746 nm to 666 nm). This was attributed to the influence of the π-acceptor ability of the ER moiety, which affects the orbital splitting of the bonding and antibonding orbitals.^494^ In contrast, compounds 856–857 show a double emission at 77 K, while 855 is non-emissive.^496^ An explanation of the different behaviors of the silver homologs is still elusive.

The Cu-thiolate adamantane moieties in [(NEt_4_]_4_[(SPh)_4_(CuX)_6_] (846–847, X = Cl, Br) show a more symmetrical buildup and a strong photoluminescence with a single emission at around 560 nm.^170^

[Et_4_N]_2_[Cu_4_(SPh)_6_] (256), comprising an inverted adamantane-type core as compared to 846 and 847, shows a much weaker photoluminescence when being excited at 350 nm, but exhibits a dual emission at 436 nm and 573 nm, which is attributed to ligand-to-metal charge transfer or transitions between the metal centers, respectively, which has not been possible for the previously discussed compounds due to their larger Cu⋯Cu distances.^170^

As part of a study on differently sized Cd-selenolate supertetrahedra, the photoluminescence of [^*n*^Pr_4_N]_2_[(CdCl)_4_(SePh)_6_] (409) was discussed.^228^ Significant emission is only detected at temperatures below ∼50 K and is attributed to forbidden transitions. Other adamantane-type thiolate clusters featuring ammonium counterions, like [Me_4_N]_2_[(CdSPh)_4_(SPh)_6_] (311) and [Et_3_NH]_2_[(CdSC_6_H_4_-4-Me)_4_(SC_6_H_4_-4-Me)_6_] (318), exhibit photoluminescence at room temperature, with low to moderate intensity.^219,221^

Group 12 chalcogenolate adamantane anions also have been subject to studies in combination with the chromophore cation DAMS. The clusters were found to affect the intramolecular charge transfer and reduce quenching.^211^

The cadmium cluster (DAMS)_2_[(CdSPh)_4_(SPh)_6_] (313) and its iodine derivative (DAMS)_2_[(CdI)_4_(SPh)_6_] (414) have been proven to show significant photoluminescence at room temperature.^218^ While (DAMS)I already exhibits an emission under similar conditions, 313 and especially 414 do so much more intensely, albeit slightly blue-shifted. Additionally, two-photon pumped lasing spectra revealed nonlinear optical properties for both compounds.

The first OLED constructed from such a compound comprises (DAMS)_2_[(ZnSPh)_4_(SPh)_6_] (302), which follows the trend of a more intense and slightly blue-shifted emission as compared to the pure chromophore.^211^ The device produces a narrow red emission at 630 nm with a full width at half heights of the measured peak of 80 nm.

Subsequently, combinations of adamantane anions and different chromophore cations were explored. In [Ru(phen)_3_][(CdSPh)_4_(SPh)_6_] (314), the fluorescence enhancing findings made for DAMS compounds could be repeated in a titration study, where an increase of the fluorescence intensity could be observed when adding cluster anions to a solution of the ruthenium complex.^219^ This effect reaches a plateau at a 1 : 1 ratio, which is in accordance with an anion–cation charge transfer indicated by both spectroscopic findings and theoretical studies.

The cluster-dye composite [Ru(2,2′-bipy)_3_][(ZnSPh)_4_(SPh)_6_] (303) was investigated for its photocurrent conversion behavior, which can be enhanced by substituting the adamantane type cluster with larger supertetrahedra.^212^

**Fig. 22:**
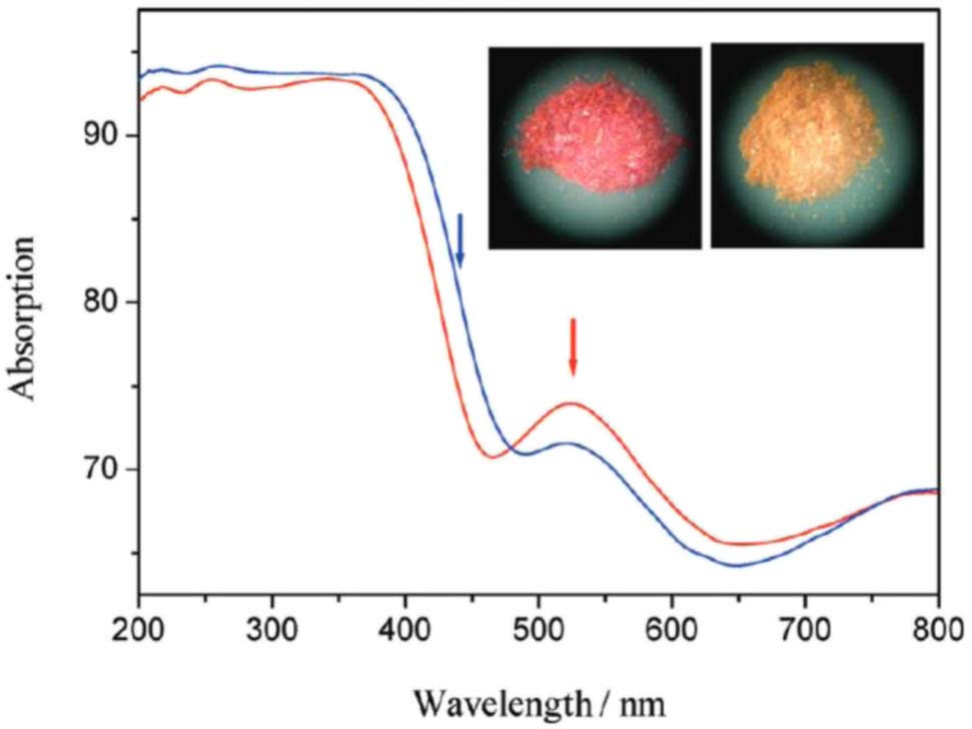
Solvatochromicity in [Ni(phen)_3_]_2_[Ge_4_S_10_] (550), depending on the inclusion of H_2_O (red curve) or MeOH (blue curve) into the crystal lattice. (reproduced from ref. 293 with permission from ACS)

The earlier UV-vis measurements on group 14/16 adamantane anions without organic ligands revealed the intra molecular transitions to be responsible for the absorption behavior, with no or only negligible contributions of the counterions in case of ammonium cations.^279,281,284,286^ The optical properties of the adamantane-type cluster remain dominant even in the presence of some transition metal complexes, as long as no charge transfer between them is possible.^295,296,298,300^ Therefore, such charge transfer pathways have to be present to influence the band gap more significantly, as has been seen in [Ni(trien)_2_]_2_[Ge_4_S_10_] (552).^288–291,294^ In the case of [Me_2_Vio]_2_[Ge_4_S_10_] (544), fluorescence can be detected distinctly red-shifted from the fluorescence of [Me_2_Vio]I_2_ by the charge transfer between the cation and anion. The cluster compound also shows solid-state solvatochromicity, depending on the inclusion of water or MeOH into the crystal structure, and it is photoelectrically active.^289^ A similar behavior in regard to fluorescence and photocurrent was found for [DMBPE]_2_[Ge_4_S_10_] (546) and for the compounds [(C_*n*_H_2*n*+1_)_2_Vio]_2_[Ge_4_S_10_] (540–543, *n* = 0, 2, 3,4).^288,291^ By utilizing a porphyrin derivative as counterion, like in the fluorescing species TMPyP[Ge_4_S_10_] (545), even larger photocurrents could be measured.^290^ Lastly, there are cases, in which the anion plays nearly no role in the transitions at the band gap, such as in [Ni_2_(μ-teta)(teta)_2_][Ge_4_S_10_] (555), in which the photoluminescence does not deviate much from the one found for the amine hydrochloride.^293,297,299^ Similar to 544, [Ni(phen)_3_]_2_[Ge_4_S_10_] (550) additionally features the ability to reversibly change its color depending on the inclusion of water or MeOH in its crystal structure (Fig. 22).^293^

For [Ni(teta)(en)][Ni(teta)(hda)][Sn_4_Se_10_] (558) moderately weak photocurrent photoelectric conversion was observed.^299^

An extensive study of lanthanide complexes of the 3-NO_2_Tp ligand features adamantane type clusters in [(μ_4_-O){M(3-NO_2_Tp)}_4_(μ_2_-OMe)_6_] (665–670), with the photoluminescence behavior depending heavily on the lanthanide center.^367^

The Yb cluster 671 also shows photoluminescence when excited at 405 nm with an emission pattern in the near infrared region, as typical for Yb^3+^ complexes.^368^

The mixed-metal compounds [(μ_3_-M)(CdPPh_3_)(MPPh_3_)_3_(TePh)_3_(μ_3_-TePh)_3_] (903–904, M = Cu, Ag) show strong photoluminescence at around 480 nm when excited at 350 nm. This was assigned to transitions between the coinage metal and its ligands, rather then involving the Cd centers.^516^ The spectrum of 903 shows an additional shoulder attributed to intracopper ds/dp transitions owing to the small Cu⋯Cu distances.

Adamantane-type clusters with a group 17 element in E position are the second group that were heavily investigated for their optical properties, chiefly among them the copper halide anions [Cu_4_E_6_]^2−^. The first bromine congener investigated was [{Cu(Hdabco)}_4_Br_6_](HCOO)_2_ (817), although a cationic one due to the ligands at the Cu sites. It showed strong photoluminescence with a yellow emission at 556 nm.^468^ Thermochromic photoluminescence can be observed for (H_2_dpipa)_3_[Cu_4_Br_6_][Cu_2_Br_6_] (815), with different bromido cuprate anions in its structure, and its Cl homolog, which was extensively studied by DFT calculations.^459,467^ The luminescence of [^i^Pr_4_N]_2_[Cu_4_Br_6_] (809) and [^*t*^Bu_3_NMe]_2_[Cu_4_Br_6_] (812) was compared to other copper bromide compounds, and 809 was found to feature the most brilliant red-orange emission, which was utilized to manufacture a white-light emitting LED in conjunction with two other commercial phosphors.^461^

The combination of bromido cuprate anions, [Cu_4_Br_6_]^2−^ among them, and a polyoxotitanium cluster in a series of compounds including [Ti_12_(μ_3_-O)_14_(O^i^Pr)_18_][Cu_4_Br_6_] (816) showed a vast dependency of the absorption spectra on the anion and the resulting supersalt structure.^466^

**Fig. 23:**
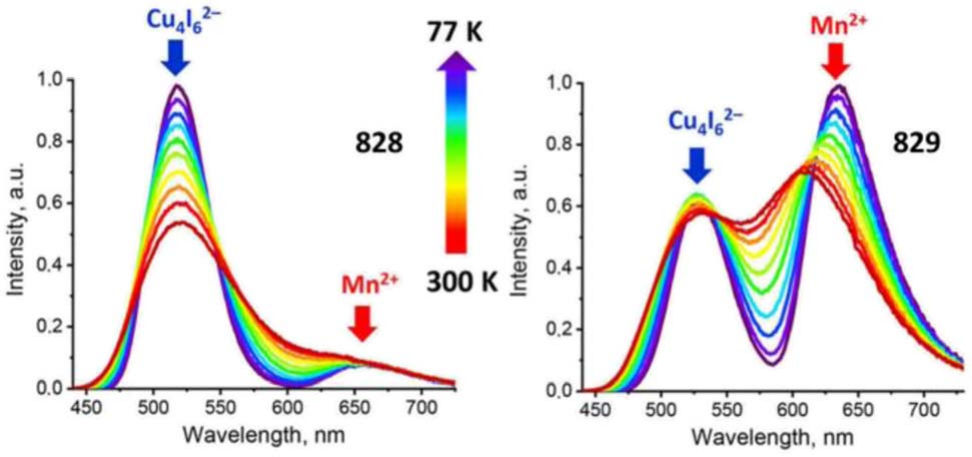
Photoluminescence at different temperatures of 828 (left, reproduced from ref. 477 with permission from ACS) and 829 (right, reproduced from ref. 477 with permission from ACS).

Also [Cu_4_I_6_]^2−^-containing compounds have been investigated for their luminescence properties in several studies.^471,472,474,476,477^ [Mn(tdpmO_3_)_2_][Cu_4_I_6_] (828) and [Mn(dppbO_2_)_3_]_2_[Cu_4_I_6_][Cu_2_I_4_] (829) are part of a series of dual-emission compounds with both the cation and anion showing a distinct emission (Fig. 23).^477^ When grinding crystals of 829, a triboluminescence originating from the same centers as the photoluminescence is detectable.

The band gap in compounds with a [μ_4_-O(CuR)_4_Cl_6_] inorganic core is determined by ligand to metal charge transfer and therefore heavily influenced by the ligand used, but such compounds do not show luminescence as opposed to their anionic relatives.^390,403,415^

## 4. Optical properties: nonlinear white-light generation (WLG) and second-harmonic generation (SHG)

The huge compositional variety of adamantane based clusters offers the possibility to finely tune the optical properties for a multitude of applications. Obviously, the fundamental element of all optical properties is defined by the HOMO–LUMO gap, in the case of adamantane (1012), this gap is ∼6.49 eV.^635^ As a consequence, the absorption onset and corresponding photoluminescence is in the ultra-violet (UV) spectral range.^635^ An advantage of this large HOMO–LUMO gap is a very high photostability of adamantane, since it is virtually unaffected by light in the visible spectral range.^636^ At the same time, the large HOMO–LUMO gap makes it difficult to make use of pure adamantane in optoelectronic applications. However, by functionalization on both the Q and E site, the HOMO–LUMO gap can easily be tuned. Functionalization schemes include fluorination, addition of alkali-metals or introduction of very simple or complex organic ligands, only to name a few.^637–640^ Even with these rather simple functionalization schemes, it is already possible to tune the HOMO–LUMO gap and thus the optical properties of fully organic adamantanes to cover virtually the whole visible spectral range.

An even larger space for tuning the optical properties opens up when stepping away from the fully organic adamantane by alternating the composition on the Q- and E-site too.

**Fig. 24:**
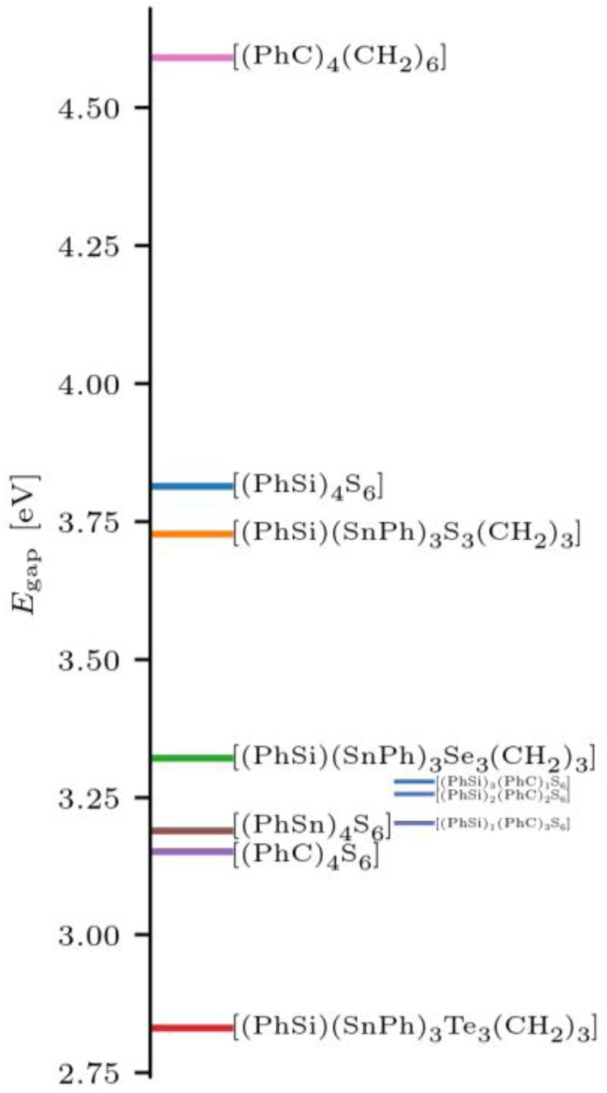
HOMO–LUMO energy gaps calculated within the density functional theory in the independent particle approximation for selected adamantane-based tetraphenyl clusters.

In the last decade, several hundreds of adamantane based clusters have been investigated theoretically.^571^ All the structures with adamantane like cores and the same ligand field show a variation in the HOMO–LUMO gaps in a range of about Δ*E* ≈ 2 eV. This is demonstrated in Fig. 24 for clusters with homogeneous phenyl ligands (tetraphenyl clusters) and different cluster cores. It should be noted that Fig. 24 is not exhaustive and only features a relatively small sample of possible modifications of the core which have been investigated recently.

The highest HOMO–LUMO gap is predicted (DFT calculations in the IPA) for the prototypical AdPh_4_ cluster (1016, *E*_g_ ≈ 4.65 eV). Modifications of the adamantane core (inorganic and/or heterogeneous composition) lower this value up to about 2 eV, with a minimum value calculated for the heterogeneous and inorganic [PhSi{CH_2_Sn(Te)Ph}_3_] (891) cluster. Interestingly, a fine tuning of the HOMO–LUMO gap value can be achieved by the gradual modification of the cluster core, as shown exemplarily by the stepwise transition from the inorganic [(PhSi)_4_S_6_] cluster (568) to the organic [(PhC)_4_S_6_] cluster.^641^ The first substitution largely modifies the HOMO–LUMO-gap, due to an abrupt modification of the bond lengths in the core. For the subsequent modifications, a quite gradual change is seen (as shown in Fig. 24). The fine tuning of the HOMO–LUMO gap is also expected for the stepwise transition between the other cores and has consequences for the manipulation of the optical response.

**Fig. 25:**
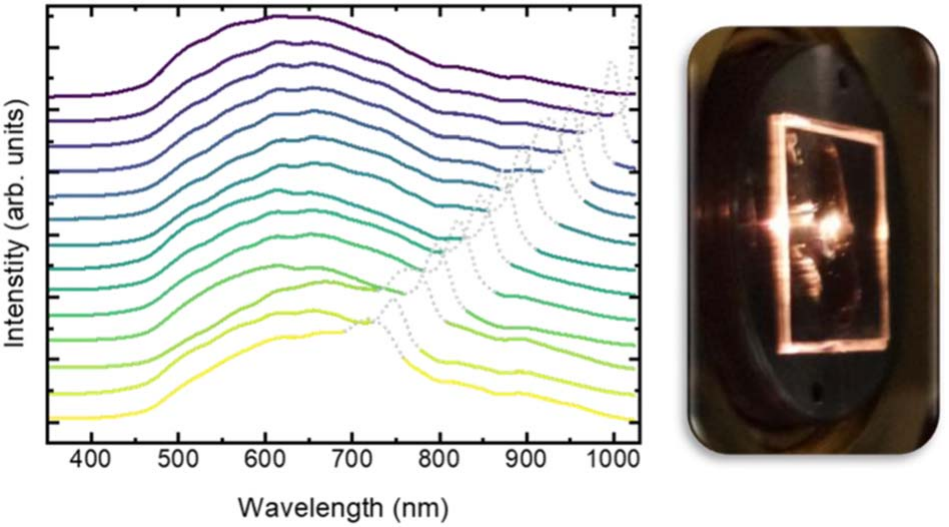
Broad white-light emission of [(StySn)_4_S_6_] (598), shown in differently colored lines for various excitation wavelength (indicated by the grey dotted part of the spectra) in the range of 700–1050 nm (left). Photograph of the emission of [(StySn)_4_S_6_] sandwiched between two glass slides and excited with 800 nm laser light (reproduced from ref. 320 with permission from AAAS).

In addition to tuneability of the linear optical properties, it was found that a large number of cluster molecules of adamantane-type cluster compounds with composition [(RQ)_4_E_6_] presented in Table 13, with group 14 elements in the Q position and those of group 16 in the E-position, show strong nonlinear optical properties when they are irradiated by a simple near-infrared (NIR) low-power laser diode.^310,320^ This nonlinear response manifests itself in the emission of light covering virtually the whole visible spectrum from ∼400 nm to 800 nm (see Fig. 25 for emission spectra under various excitation energies with excitation region marked by dotted grey line). Because of the broad spectral-range, we will refer to the process as white-light emission, although due to the spectral intensity distribution the appearance to the human-eye is warm-white (see Fig. 25).

As it has been demonstrated that the nonlinear optical response of the clusters can be generally interpreted in terms of multiphoton processes,^309,642,643^ tuning the HOMO–LUMO gap makes it possible to tune the nonlinear response too, *e.g.*, the frequency dependence of the main SHG and THG peaks. It is worth mentioning here that SHG can be observed on these materials even for compounds that crystallize in centrosymmetric space groups, which usually is an exclusion rule, as only crystals lacking inversion symmetry can produce bulk SHG. However, the SHG in such samples appears to be due to surface effects and/or defects in the crystal. With the SHG being very effective, the contribution of the surfaces of the (micro- or nanosized) crystals is sufficiently high to observe SHG in most cases.

**Fig. 26:**
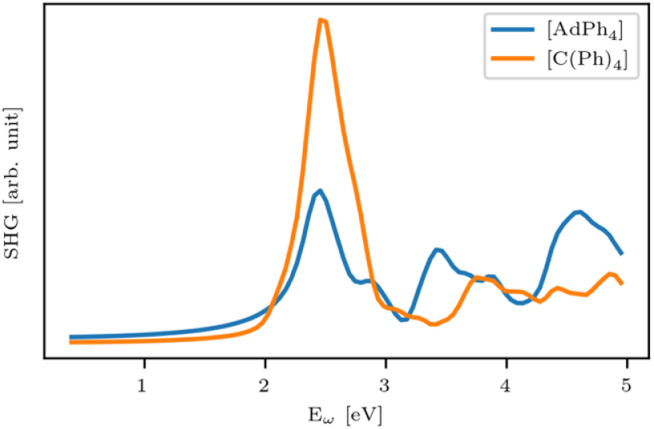
Second-harmonic generation (average of all tensor components, absolute values) calculated as a function of the incident laser wavelength for the isolated [AdPh_4_] cluster (1016) (blue line) and the [CPh_4_] cluster (orange line) within the independent particle approximation at the DFT equilibrium geometry.^646^

The optical response of a wide class of adamantane-based clusters and cluster aggregates has been calculated from first principles in recent years.^309,571,642–645^ The calculations show that all adamantane-based clusters are characterized by a nonlinear optical response with the same structure, as long as they have the same ligand field. For example, a prominent peak above 2 eV followed by a dip and a second peak is common to all adamantane-based cores with phenyl ligands. This suggests that the nonlinear optical response is dominantly defined by the ligand structures and originates only to a minor extent in the core region. This is demonstrated exemplarily in Fig. 26, where we compare the SHG signal of AdPh_4_ (1016) with that calculated on the same footing for CPh_4_.^646^

The structure of the second order nonlinear response is basically the same for the two clusters, although CPh_4_ has no adamantane core at all. This, however, does not mean that the cluster core does not affect the linear and nonlinear optical properties. As we will discuss in the following, the geometry and the chemistry of the core have an impact on the magnitude of the nonlinearities and, to a lesser extent, to their spectral position. Thus, modifications of the cluster core offer the possibility to manipulate the optical response towards desired energies and intensities. Yet, the main characteristics are preserved as long as the ligand structures are maintained.

**Fig. 27:**
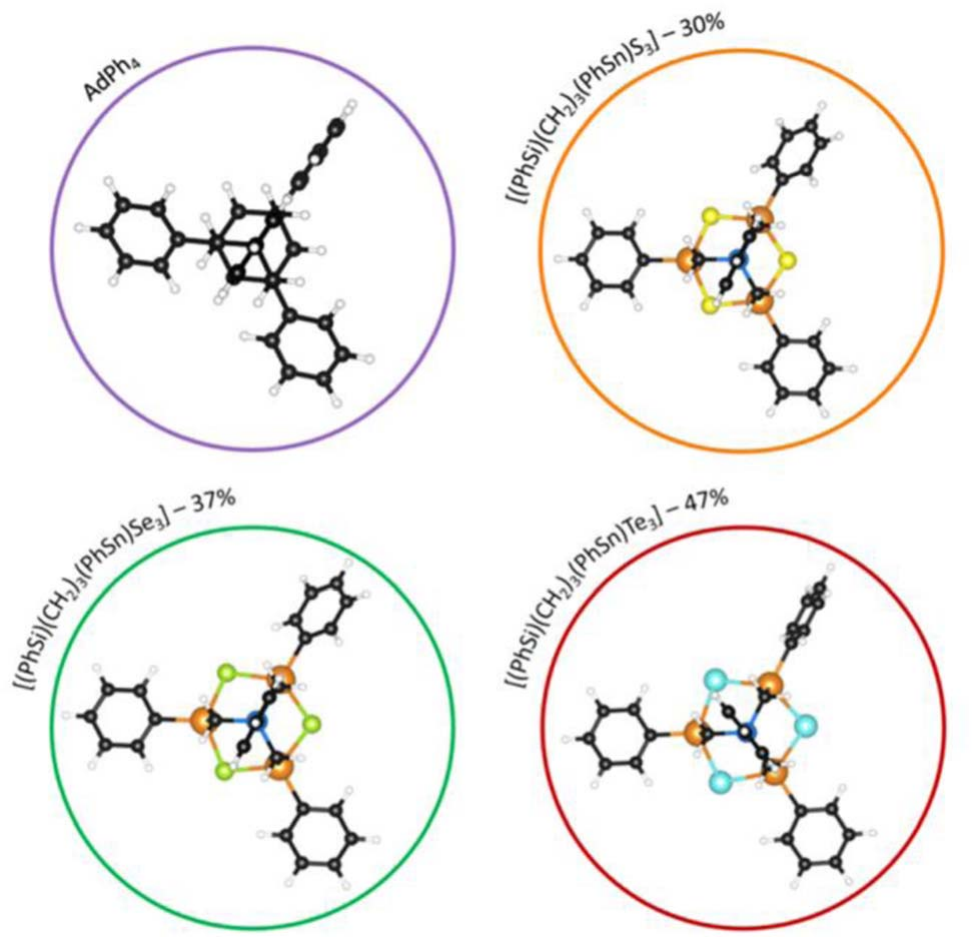
Atomic structure calculated within DFT in the independent particle approximation for AdPh_4_ (1016, top left) and different tetraphenyl clusters with modified cluster cores. The circle color corresponds to the respective line color of the second order optical response shown in Fig. 28.^642,644^

**Fig. 28:**
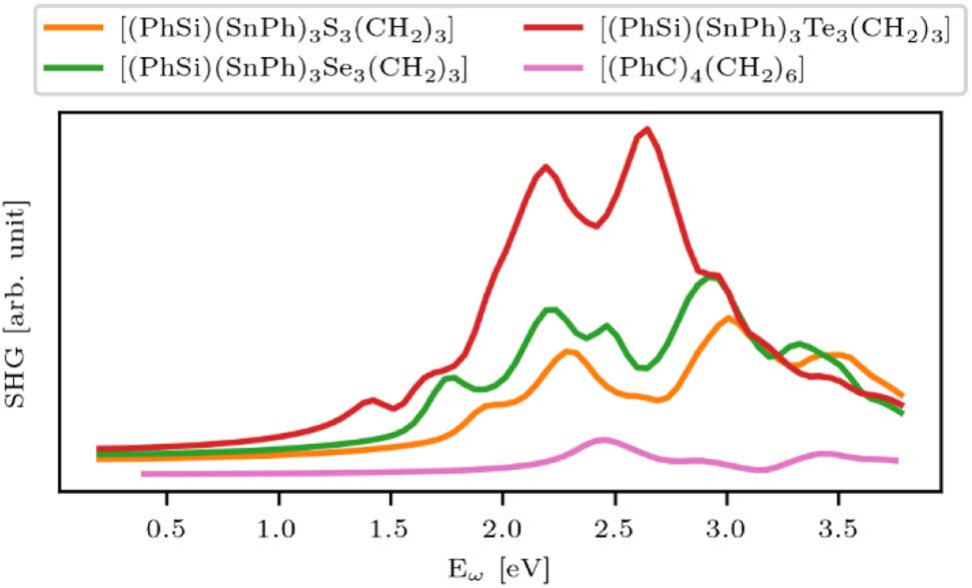
Second-harmonic generation per cluster (the average of all tensor components is shown) calculated within DFT in the independent particle approximation for different tetraphenyl clusters with modified cluster cores.^642,644^

Although the nonlinear optical response originates in and is dominated by the ligand field, as previously discussed, the optical nonlinearities are enhanced by disorder and structural asymmetry in the cluster core. This is particularly true for molecules featuring a heterogeneous core composition.^642^ This is clearly shown with clusters [PhSi{CH_2_Sn(E)Ph}_3_] (889–891, E = S, Se, Te) as a model system. The cluster structure is shown in Fig. 27, along with the distortion in the core, quantified by the ratio between the largest and shortest bond length. The core symmetry is greatly reduced from S to Te, while the optical susceptibilities are correspondingly enhanced, as displayed in Fig. 28. The AdPh_4_ cluster features a regular core and has the lowest SHG coefficients. This effect is quite remarkable, as a range of intensities spanning an entire magnitude of SHG responses can be achieved with these structures. Noticeably, the form of the second order optical response typical for tetraphenyl compounds (displaying a main peak above 2 eV, followed by a dip and a second peak) is maintained for all variations of the core.

Although a generally accepted theory of the observed white-light generation is still missing, it is known that higher order nonlinear effects like supercontinuum generation involve the whole catalogue of nonlinear-optical effects, which add up to produce emission with an extremely broad spectrum.^646–648^ These nonlinear effects include self- and cross-phase modulation, four-wave mixing, and many others, for which an exhaustive theory is still missing. Nevertheless, it is reasonable to assume that a more or less high nonlinear optical activity is a prerequisite for the mentioned effects leading to white-light emission. In this respect, the theoretical studies performed in the last years allowed for an (at least partial) understanding of the optical response of the white-light emitters and revealed several interesting aspects. As a general feature, all clusters showing WLE are characterized by strong optical nonlinearities of second and third order, at least as strong as that of the crystalline ferroelectrics.^309,571,642–646,650,651^ The optical nonlinearities feature high peaks and low dips at which the optical coefficients are almost quenched, as seen by the example in Fig. 28 for the prototypical AdPh_4_.

**Fig. 29:**
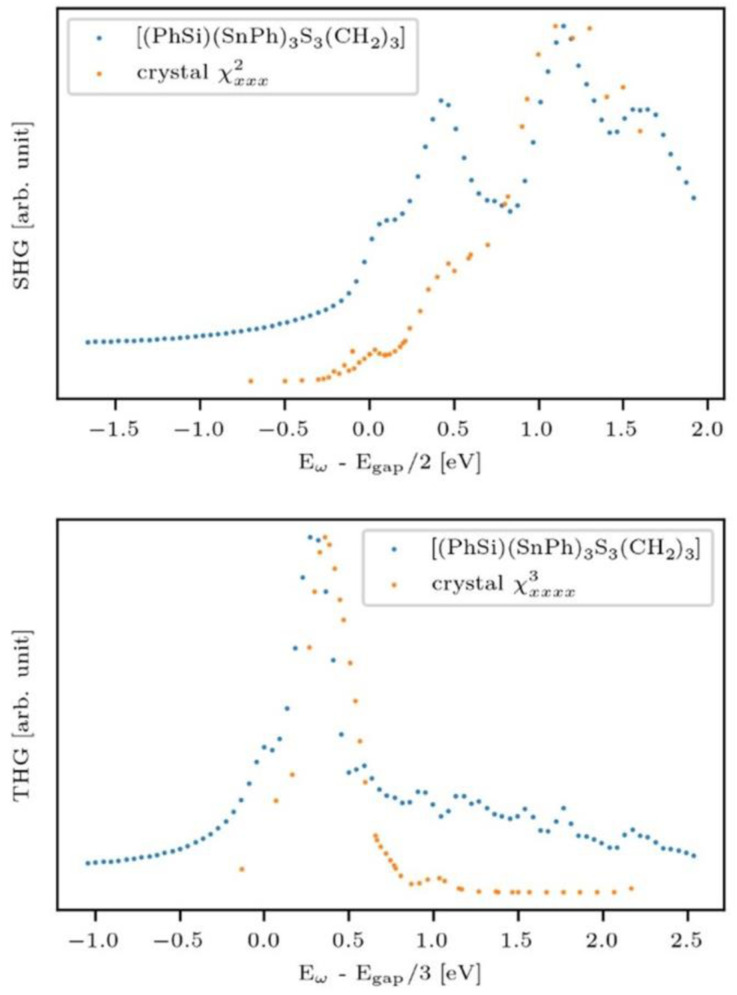
Second-harmonic generation (top, *χ*^(2)^_*xxx*_ component) and third harmonic generation (bottom, *χ*^(3)^_*xxxx*_ component) coefficients (absolute values) calculated as a function of the incident laser wavelength for the isolated [PhSi{CH_2_Sn(S)Ph}_3_] cluster (blue dots) and the corresponding crystal (orange dots) within the independent particle approximation at the DFT equilibrium geometry. Intensities are scaled by the respective maximum for each structure, and energies are shifted relative to the energy gap.^642,644^

The optical response is thus strongly dependent on the incident photon energy, with most compounds showing a maximum of the SHG coefficients above 2 eV and a THG maximum just below 2 eV (see *e.g.*, Fig. 29). In general, the white-light emission efficiency is expected to depend on the exciting wavelength.^648^ However, this does not seem to be the case for the adamantane-type cluster molecules.^320,571^ This might be related to the fact that in the adamantane-type cluster molecules, white-light emission is achieved by an excitation in a generally non-resonant region of the nonlinear optical spectrum (1.1–1.3 eV), however, where the onset of the optical nonlinearities is already pronounced. As this spectral region is followed by a steep gradient of the nonlinear optical susceptibilities, the white-light emission efficiency might be further increased, provided it correlates (as currently assumed) with the optical nonlinearities.

## 5. Materials properties (crystalline/amorphous, glass formation): comprehension and prediction

A common trend identified in multiple structures with adamantane and adamantane-like cores is the inheritance of both the linear and the nonlinear optical response from the parent molecules to dimers and the crystal structures.^642,644^ This is admittedly shown in Fig. 29 for the [PhSi{CH_2_Sn(S)Ph}_3_] molecule. However, a similar trend has been demonstrated also for many other adamantane-based clusters such as [(PhSi)_4_S_6_] and [(NpSi)_4_S_6_].^309,571^Fig. 29 shows that all spectral features of the isolated molecules can be found in the optical response of the crystals, although the spectral weights are somewhat redistributed. This similarity offers the possibility to roughly estimate, *e.g.*, the second and third order optical nonlinearity by the knowledge of the corresponding single molecule. This is a great advantage, in particular in theoretical studies, due to the computationally less extensive investigations of the single clusters as compared to molecular crystals.

It has been discussed in many publications that an amorphous structure is a prerequisite for white-light generation.^652^ Although the connection between the habitus of the aggregate and its nonlinear optical answer is not completely understood, atomistic calculations show a clear relation between aggregate symmetry and intensity of the nonlinear optical response. We demonstrate this connection employing the prototypical adamantane-type cluster [AdPh_4_] as a model system.

**Fig. 30:**
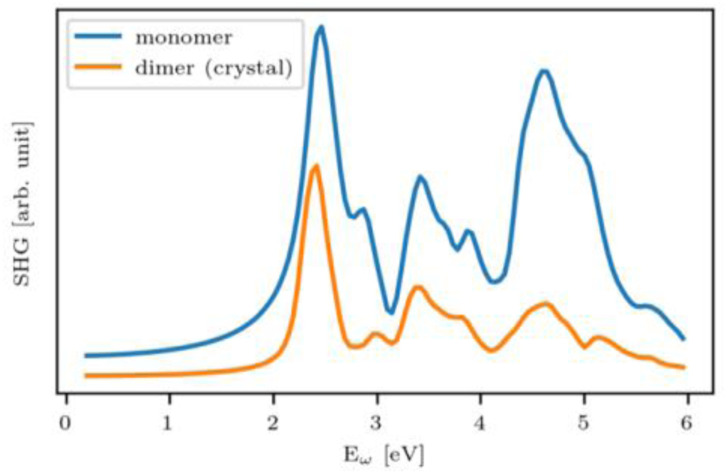
Second-harmonic generation per cluster (the average of the absolute value of all tensor components is shown) calculated within the independent particle approximation as a function of the incident laser wavelength for the isolated [AdPh_4_] cluster with the DFT equilibrium geometry (blue line) and with the geometry of a cluster dimer cut out from a [AdPh_4_] crystal (orange line).^644^

Depending on the environment, the geometry and the symmetry of the cluster undergo slight modifications and so does the nonlinear optical response, quantified, in this example, by the average of all the components of the SHG tensor. [AdPh_4_] belongs to the space group *P*4̄2*n*. This group lacks centrosymmetry, so that SHG generation is expected. Indeed, the isolated cluster in its calculated equilibrium geometry is characterized by a second-order optical response as shown by the blue line in Fig. 30. In aggregates such as [AdPh_4_] crystals, the material can be thought of as a periodic repetition of [AdPh_4_] dimers.^644^ The dimers are arranged in a manner that is still not centrosymmetric, however, the deviation from the centrosymmetry is lower than in the case of the isolated clusters. The SHG response per molecule, shown in Fig. 30 (orange line) features all the spectral signatures predicted for the isolated cluster, however, with a lower intensity.

**Fig. 31:**
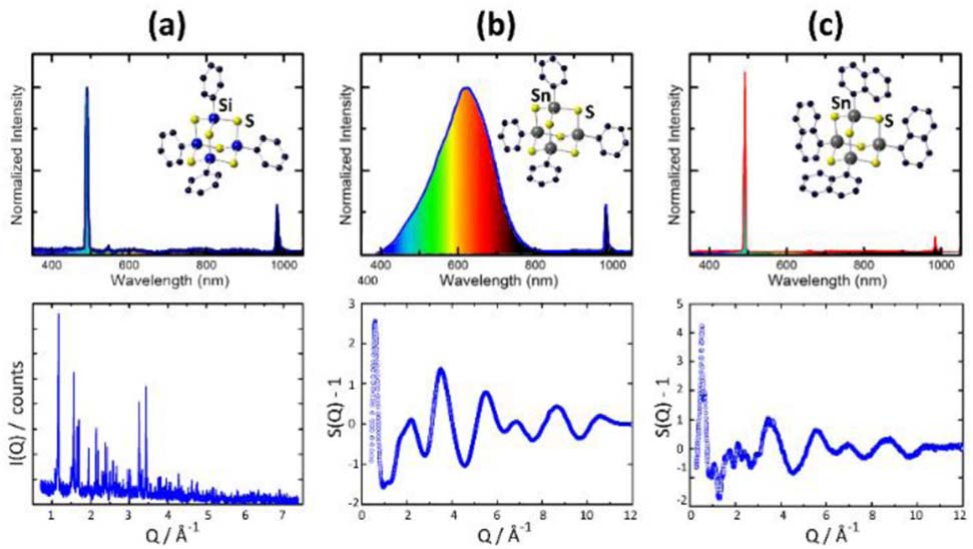
NLO responses from the clusters 568 (a) and 587 (b) (top). The driving excitation is visible at 979 nm (1.265 eV) in each spectrum. The 2nd-harmonics of (a) is seen at 489.5 nm (2.53 eV), while (b) depicts a broad white spectrum. The respective X-ray patterns are also shown below indicating that 568, the SHG-material, is clearly crystalline while the WLG material 587 shows a typical structure factor S(Q) of an amorphous solid. (c) shows the NLO response of cluster 591 indicating SHG, although the X-ray structure factor clearly designates an amorphous solid (reproduced from ref. 657 with permission from the authors under CC BY 4.0).

Artificially modifying the structure towards centrosymmetry further lowers the SHG coefficients. The same behavior is observed for other optically nonlinear molecular clusters. Differences in the symmetry of monomer and dimer structures can also greatly influence the magnitude of the optical response, yet maintaining its overall form. Especially the differences of the nonlinear optics of free-standing dimers compared to their crystal counterparts show that the habitus of the material offers a path to tuning the symmetry and thus the nonlinear optical properties. WLG is only observed in samples with amorphous morphology. The crystalline members of the class of adamantane-based cluster compounds show a different nonlinear optical response, generally SHG originating from the bulk (in the case of crystals lacking inversion symmetry) or from surfaces or interfaces and defects (in the case of centrosymmetric crystals).^649^ This is shown in Fig. 31 for the crystalline compound [(PhSi)_4_S_6_] (568) (a) and for the amorphous material [(PhSn)_4_S_6_] (587) (b) as examples. Both clusters are provided with the same organic phenyl ligands and differ just by the exchange of Si atoms by Sn atoms in the adamantane shaped cluster cores. The solid with the {SiS} cluster core (568) shows the typical powder diffractogram of a crystal (Fig. 31a). When irradiated by an optical laser line with wavelength 979 nm, it reacts with an intense second-harmonic at 489 nm. In contrast, the X-ray diffractogram obtained from the solid of the corresponding {SnS} cluster (587) yields a pattern typical for a completely disordered material like a glass, not comprising any information about structural long-range order between the molecules (Fig. 31b). However, the optical response to NIR irradiation at 979 nm is now found to be a broad white emission that spans the entire visible portion of the white-light spectrum.

Meanwhile, a large number of solids of this type could be synthesized showing this correlation between morphology and optical behavior.^571^ While all crystalline materials have been identified as materials for second-harmonic generation (SHG), it however turned out that among the amorphous materials, it is only the vast majority responding by white-light generators (WLG). A few apparently amorphous clusters whose diffractograms resemble those of glasses, with pronounced structural disorder at the molecular level, nevertheless exhibit SHG upon NIR irradiation. The optical response and X-ray diffractogram of one such example, the {SnS} cluster decorated with naphthyl ligands, [(NpSn)_4_S_6_] (591), are shown in Fig. 31c. Although the X-ray diffractogram of (591) does not show typical Bragg peaks but rather resembles the typical structure factor S(Q) for a glass, the optical response when irradiated with the NIR line at 979 nm is found to be SHG.

In trying to understand this behavior, two questions come to mind: What controls the solidification of these cluster molecules into either an amorphous or a crystalline state, and why is pronounced microscopic disorder a prerequisite for a nonlinear response in the form of WLG? In order to answer these questions, it is compulsory to first obtain precise knowledge of the interactions between the clusters, which however requires detailed knowledge of their electronic structure as a function of composition. Then, it must be further understood how the molecular and electronic structure is altered when the clusters aggregate into either a crystalline or amorphous condensed phase. This is a prerequisite for finding approaches from the observed differences that may lead to an understanding of the different optical behavior of these clusters in the two different solid-state morphologies. For this purpose, several optical studies were performed on different cluster systems.^653^ Furthermore, the electronic structures of many clusters from this group with different organic ligands and {QE} cluster cores were calculated, as well as interaction energies between the clusters for simple arrangements of two to four clusters as simple models to distinguish between different contributions to the interactions.^309,644^ Also, experimental investigations were accomplished to explore the mutual orientation of the clusters in the amorphous solid.^654–657^ It showed up thereby however, that the control of the morphology is complex and an assignment amorphous/WLG, crystalline/SHG is oversimplified. It was however found from EXAFS experiments^658^ that the molecular structure of adamantane-like cluster materials showing SHG (586 and 591) was close to that found from quantum chemical calculations,^659^ while those showing WLG (587 and 602) considerably deviated from these structures.

More detailed studies carried out on the WLG compound 587 and the SHG compound 591 using X-ray diffraction combined with molecular Reverse Monte Carlo (m-RMC) simulations further revealed that this observation results from significantly distorted cluster cores in the compounds showing WLG. The distortions originate from sulfur atoms moving out of their equilibrium positions, towards other sulfur atoms of neighboring molecules. This may result from strong interactions between the cores of the individual clusters, as was also suggested by quantum chemical calculations,^309,660^ where smaller cluster aggregates of two to four molecules were assumed as simple models for a disordered phase. Indeed, the mutual cluster orientations predicted there for the WLG compound 587 were also found in the experimental X-ray/RMC-studies^657^ supporting the theoretical findings. Similar experimental studies on the SHG compound 591 revealed no evidence for distorted cluster cores. The mutual molecular orientation of the clusters in the solid state also suggested stronger intermolecular interactions *via* the organic ligands, which also supports the quantum chemical calculations made earlier. In addition, electron microscopy could reveal sections of nanocrystallinity, which may additionally explain the findings by ordered parts of the material.

The observation of additional cluster core distortions in the material exhibiting strong WLG compound 587, further highlights the point made earlier that a clear relationship exists between structural and molecular symmetry and intensity of the nonlinear optical response.

## 6. Conclusion and outlook

The number of compounds based on adamantane-type core architechture is vast, and the elemental compositions of the cluster cores as well as the variety of the substituents is extremely diverse. While this naturally leads to different chemical reactivities and unique electronic structures, a number of them share luminescence phenomena. Only recently, strong second-harmonic generation as well as more uncommon nonlinear optical properties in the form of directed white-light emission have been observed, which seem to be a consequence of the unique adamantane type architecture – with its relatively dense electronic structure and its inherently missing inversion symmetry – as well as of the compounds' arrangement within the solid material.^571^ Assuming that these phenomena could in theory be observable for other adamantane-type cluster compounds, we have summarized all classes of inorganic and a selection of organic admantanes in this survey. Most of these compounds have not been fully investigated for their physical properties so far, but we suggest that doing so could be a great chance to gain understanding on the optical phenomena in this class of compounds and its potential use. The plethora of different compositions and structural peculiarities is an excellent basis for future investigations into this field, which could become a library of compounds that fulfils any desired properties in regards of wavelength, bandwidth, intensity, and directionality in combination of convenient synthetic access, robustness, and processability of the material.

## Abbreviations

IDipp: 1,3-Bis(2,6-diisopropylphenyl)imidazole-2-ylidene
HMDS: 1,1,1,3,3,3-Hexamethyldisilazide
TACNMe: 1,4,7-Trimethyl-1,4,7-triazacyclononane
Bn: Benzyl
Cp*: Pentamethylcyclopentadienyl
DMAP: Dimethylamine borane
BAr^F^: [B[3,5-(CF_3_)_2_C_6_H_3_]
^*n*^Bu: Normal butyl
^*t*^Bu: Tertiary butyl
Diglyme: Bis(2-methoxyethyl) ether
DMPyr: 1,1-Dimethylpyrrolidinium
OTf: O_3_SCF_3_
cod: 1,5-Cyclooctadiene
OAc: Acetate
acac: Acetylacetonate
BMIm: 1-Butyl-3-methyl-imidazolium
NTf_2_: Bistrifluoridomethansulfonimide
TMEDA: Tetramethylethylenediamine
^i^Pr: Isopropyl
DME: 1,2-Dimethoxyethane
Ar^Me6^: C_6_H_3_-2,6(C_6_H_2_-2,4,6-Me_3_)_2_
mes: 2,4,6-Me_3_-C_6_H_2_
Tp^Me2^: Tri(3,5 dimethylpyrazolyl)borate)
TACN: 1,4,7-Triazacyclononane
DMSO: Dimethyl sulfoxide
Cp^*x*Ph^: C_5_Me_4_Ph
OHF: 1,2,3,4,5,6,7,8-Octahydrofluorenyl
dmae: *N*,*N*-Dimethylaminoethanolate
TFA: Trifluoacetic acid
H_4_cit: Citric acid
H_2_DBcat: 3,5-Di-*tert*-butylcatechol
HBO: 2-(2′-Hydroxyphenyl)benzoxazole
tach: 1,3,5-Triaminocyclohexane
en: Ethylendiamine
H_5_hpdta: Hydroxypropanediaminotetraacetic acid
HIPAP: *N*-(*tert*-Butyl)-3-((3,5-di-*tert*-butyl-2-hydroxybenzylidene)amino)-propanamide
tdmap: OC(CH_2_NMe_2_)_3_
S-Phoz: 2-(4′,4′-Dimethyloxazoline-2′-yl)thiophenolate
bpea: *N*,*N*-Bis(2-pyridylmethyl)ethylamine

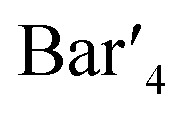

[3,5-(CF_3_)_2_C_6_H_3_]_4_B]^−^
dien: Diethylenetriamine
Medien: *N*′-Methyldiethylenetriamine
R-ida: *N*-(*R*)Iminodiacetate
^*C*^Pe: Cyclopentane
tame: *tert*-Amyl methyl ether
Htphpn: *N*,*N*,*N*′,*N*′-Tetra-(2-methylpyridyl)-2-hydroxypropanediamine
pz: Pyrazolyl
H_2_BMAP: 2-[bis(2-mercaptoethyl)aminomethyl]pyridine
Py: Pyridine
H_5_HMeXCG: *N*,*N*′-(2-Hydroxy-5-methyl-1,3-xylylene)bis(*N*-(carboxymethyl)glycine)
H_5_HPhXCG: *N*,*N*′-(2-Hydroxy-5-phenyl-1,3-xylylene)bis(*N*-(carboxymethyl)glycine)
Dma: *N*,*N*-Dimethylacetamid
Hbpbp: 2,6-Bis((*N*,*N*′-bis-(2-picolyl)amino)methyl)-4-*tert*-butylphenol
{(TACN)CH_2_}_2_CHOH: 1,3-Bis(1,4,7-triaza-1-cyclononyl)-2-hydroxypropane
N-Et-HPTB: *N*,*N*,*N*′,*N*′-Tetrakis(2-(1-ethylbenzimidazolyl))-2-hydroxy-1,3-diaminopropane
dppoe: 1,2-Bis(diphenylphosphine oxide)ethane
dppe: 1,2-Bis(diphenylphosphino)ethane
H_3_HMPM: 2,6-Bis[{{(1-hydroxy-2-methylpropan-2-yl)(pyridine-2-ylmethyl)}amino}methyl]-4-methylphenol
Me_2_phen: 2,9-Dimethyl-1,10-phenanthroline
BIK: Bis(2-methyl-imidazole-2-yl)ketone
^*t*^Bu_2_DED: 1,1-Dicarbo-*tert*-butoxy-2,2-ethylenedithiolate
tpdt: 3,4-Thiophenedithiolate
α-tpdt: 2,3-Thiophenedithiolate
H_4_pymtH: 3,4,5,6-Tetrahydropyrimidine-2-thione
H_3_O3N4: 1-Me-4-OH-3,4-bis(CH_2_N(CH_2_C_5_H_4_N)(CMe_2_CH_2_OH)–C_6_H_2_
Fc: Ferrocenyl
2-TBI: 2-Thiobenzimidadzol
Mbis: 1,1′-Methylenebis(3-methylimidazoline-2-selone)
DAMS: *Trans*-4-(4-dimethylamino-styryl)-*N*-methyl-pyridinium
bipy: Bipyridine
^*n*^Pr: Normal propyl
^sec^Bu: Secondary butyl
phen: 1,10-Phenanthroline
^*o*^Py: *ortho*-Pyridyl
Tab: 4-(Trimethylammonio)benzenethiolate
[2.2.2]-crypt: 4,7,13,16,21,24-Hexaoxa-1,10-diazabicyclo[8.8.8]hexacosane
POPYH_4_: *N*,*N*′-Bis(2-hydroxyphenyl)-pyridine-2,6-dicarboxamide
dap: 1,2-Diaminopropane
tepa: Tetraethylenepentamine
Vio: Viologen dication
TMPyP: 5,10,15,20-Tetrakis(*N*-methyl-4-pyridyl)porphyrin
DMBPE: *N*,*N*′-Dimethyl-1,2-bis(4-pyridinium)-ethylene
Cyclam: 1,4,8,11-Tetraazacyclotetradecane
Trien: Triethylentetramin
Teta: Triethylenetetramine
Thex: 1,1,2-Trimethylpropyl
Np: Naphthyl
Sty: *para*-Styryl
Cy: Cyclohexyl
Cp: Cyclopentadienyl
Dipp: 2,6-Diisopropylphenyl
DMEGqu: *N*-(1,3-Dimethylimidazolidin2-ylidene)quinoline-8-amine
8-HQ: 8-Hydroxyquinoline
H_2_naphpz: 2-[1*H*-Pyrazol-5(3)-yl]naphthalene-1-ol
dpan: 6-Diphenylphosphinoacenaphth-5-yl
L_OEt_: [Co(η^5^-C_5_H_5_){P(O)(OEt)_2_}_3_]^−^
3-NO_2_Tp: 3-Nitrotrispyrazolylborate
SON: (Benzothiazole-2-yl)phenolate
HBT: 2-(2-Hydroxyphenyl)benzothiazole
H_3_L: 2-Hydroxy-*N*-[2-hydroxy-3-[(2hydroxybenzoyl)amino]propyl]benzamide
C_4_mim: 1-Butyl-3-methylimidazolium
da6aH_6_: *p*-Methyl-dimethyldiazacalix[6]areneH_6_
HMTA: Hexamethylentetramine
HBDA: Hexakis(trimethylsilyl)benzdiamidine
cpz: 2-Chloro-10-(3-dimethylaminopropyl(phenothiazine)
DENC: *N*,*N*-Diethylnicotinamide
Pz^iPr2^H: 3,5-Diisopropylpyrazole
DASO: Diallyl sulfoxide
Amt: 1,3-Diamino-1,2,2-trimethylcyclopentane
CgP: 1,3,5,7-Tetramethyl-2,4,6-trioxa-8-phosphatricyclo[3.3.1.1]-decane
nmp: *N*-Methyl-2-pyrrolidinone
teed: *N*,*N*,*N*′,*N*′-Tetraethylethylenediamine
BPBACy: Bis(1-propylbenzimidazol-2-yl)-trans-1,2-cyclohexane
dpipa: *N*,*N*′-Dimethylpiperazine
PoxIm: *N*-Phenyl-*N*′-{bis(tertbutyl)phosphinoxide}-imidazolylidene
Dabco: 1,4-Diazabicyclo[2.2.2]octane
tib: 1,3,5-Tris(1-imidazolyl)benzene
pyr: Pyrrolidine
dppbO_2_: 1,2-bis(diephenlyphospineoxide) benzol
tdpmO_3_: tris(diephenlyphospineoxide) methan
BPPIP: Bis-triphenylphosphonio-isophosphindolide
THP: Tetrahydropyran
tren: Tris(2-ethylamino)amine
gua: Guanidine
bme*daco: Bis(*N*,*N*′-2-mercapto-2-methylpropyl)1,5-diazocyclooctane
Bdpman: *N*,*N*′-Bis(diphenylmethyl)-3,7-diazabicyclo[3.3.1]nonane
Hbdmap: 1,3-Bis-(dimethylamino)-propan-2-ol
^*n*^Hep: Normal heptane
TAEA: Tris(2-aminoethyl)amine
tdci: 1,3,5-Trideoxy-1,2,5-tris(dimethylamino)-*cis*-inositol
Hchp: 6-Chloro-2-hydroxypyridine
dmit: 4,5-Dimercapto-1,3-dithiole-2-thionato
EDTA: Ethylenediamine-tetraacetate
BMMIm: 1-Butyl-2,3-dimethyl-imidazolium
MCPBA: *m*-Chloroperbenzoic acid

## Author contributions

All athors agreed on the concept of the article and co-wrote the paper.

## Conflicts of interest

There are no conflicts to declare.
